# High Target Homology Does Not Guarantee Inhibition: Aminothiazoles Emerge as Inhibitors of *Plasmodium falciparum*

**DOI:** 10.1021/acsinfecdis.3c00670

**Published:** 2024-02-17

**Authors:** Sandra Johannsen, Robin M. Gierse, Arne Krüger, Rachel L. Edwards, Vittoria Nanna, Anna Fontana, Di Zhu, Tiziana Masini, Lais Pessanha de Carvalho, Mael Poizat, Bart Kieftenbelt, Dana M. Hodge, Sophie Alvarez, Daan Bunt, Antoine Lacour, Atanaz Shams, Kamila Anna Meissner, Edmarcia Elisa de Souza, Melloney Dröge, Bernard van Vliet, Jack den Hartog, Michael C. Hutter, Jana Held, Audrey R. Odom John, Carsten Wrenger, Anna K. H. Hirsch

**Affiliations:** † Helmholtz Institute for Pharmaceutical Research Saarland (HIPS) − Helmholtz Centre for Infection Research (HZI), Campus Building E8.1, Saarbrücken 66123, Germany; ‡ Department of Pharmacy, Saarland University, Campus Building E8.1, Saarbrücken 66123, Germany; § Stratingh Institute for Chemistry, University of Groningen, Nijenborgh 7, Groningen 9747 AG, The Netherlands; ∥ Unit for Drug Discovery, Department of Parasitology, Institute of Biomedical Sciences, University of São Paulo, Av. Prof. Lineu Prestes 1374, São Paulo-SP 05508-000, Brazil; ⊥ Department of Pediatrics, Washington University School of Medicine, Saint Louis, Missouri 63110, United States; # Institute of Tropical Medicine, University of Tübingen, Wilhelmstraße 27, Tübingen 72074, Germany; ∇ Symeres, Kadijk 3, Groningen 9747 AT, The Netherlands; ○ Department of Pediatrics, Children’s Hospital of Philadelphia, Perelman School of Medicine, University of Pennsylvania, Philadelphia, Pennsylvania 19104, United States; ◆ Proteomics & Metabolomics Facility, Center for Biotechnology, Department of Agronomy and Horticulture, University of Nebraska-Lincoln, Lincoln, Nebraska 68588, United States; ¶ Center for Bioinformatics, Saarland University, Campus Building E2.1, Saarbrücken 66123, Germany; β German Center for Infection Research (DZIF), Partner Site Tübingen, Tübingen 72074, Germany; α Centre de Recherches Médicales de Lambaréné (CERMEL), B.P. 242 Lambaréné, Gabon

**Keywords:** MEP pathway, malaria, *Plasmodium falciparum*, DXPS, Polypharmacology Browser

## Abstract

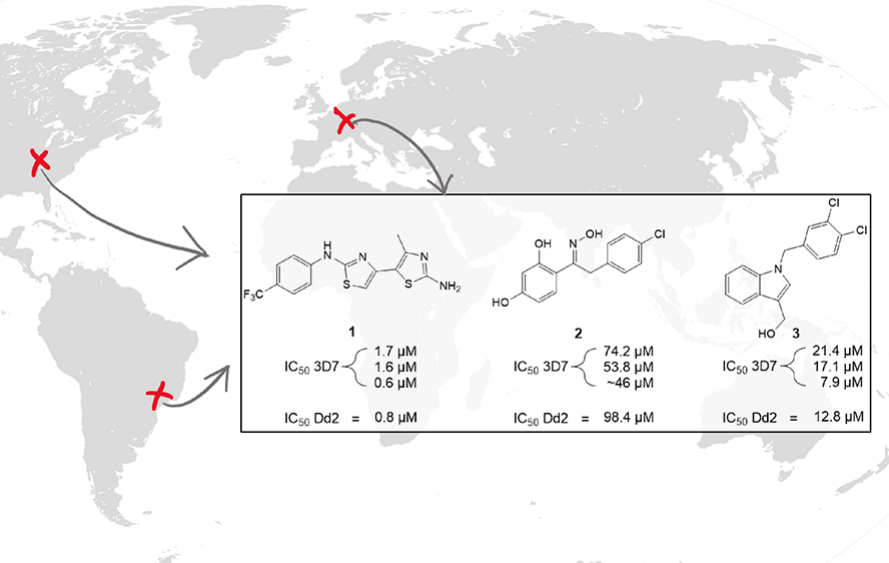

In this study, we identified three novel compound classes with potent activity against *Plasmodium falciparum*, the most dangerous human malarial parasite. Resistance of this pathogen to known drugs is increasing, and compounds with different modes of action are urgently needed. One promising drug target is the enzyme 1-deoxy-d-xylulose-5-phosphate synthase (DXPS) of the methylerythritol 4-phosphate (MEP) pathway for which we have previously identified three active compound classes against *Mycobacterium tuberculosis*. The close structural similarities of the active sites of the DXPS enzymes of *P. falciparum* and *M. tuberculosis* prompted investigation of their antiparasitic action, all classes display good cell-based activity. Through structure–activity relationship studies, we increased their antimalarial potency and two classes also show good metabolic stability and low toxicity against human liver cells. The most active compound **1** inhibits the growth of blood-stage *P. falciparum* with an IC_50_ of 600 nM. The results from three different methods for target validation of compound **1** suggest no engagement of DXPS. All inhibitor classes are active against chloroquine-resistant strains, confirming a new mode of action that has to be further investigated.

Malaria remains one of the major diseases with a high impact on health and welfare worldwide, especially in subtropical regions. In 2020, the World Health Organization (WHO) reported an estimated number of 627,000 deaths worldwide.^1^ Among the six known human malaria parasites, *Plasmodium falciparum* is responsible for the majority of deaths. To treat uncomplicated *P. falciparum* malaria, artemisinin-based combination therapies (ACTs) are recommended but the potent artemisinin derivatives must be partnered with a second drug due to their short half-life. Currently, six different ACTs are in use but decreasing potency of artemisinin derivatives displayed by a delayed clearance phenotype is widespread in Southeast Asia, together with resistances to the partner drugs in this combinations, is threatening the efficacy of these treatments.^2^ Therefore, finding new compounds with novel modes of action is of great importance.

A promising pool of targets is the methylerythritol 4-phosphate (MEP) pathway that is utilized by many human pathogens, such as *P. falciparum* and *Mycobacterium tuberculosis* (Scheme 1). The final products of the MEP pathway are isopentenyl diphosphate (IDP) and dimethylallyl diphosphate (DMADP), two precursors for the biosynthesis of isoprenoids. In malaria parasites, the MEP pathway is located in the apicoplast, a plastid-like organelle of prokaryotic origin. Removing this organelle showed its crucial role for cell survival but also that addition of IDP or DMADP rescues the parasites. This result demonstrated the significance of the MEP pathway in *P. falciparum* and its validity as a drug target.^3^ Furthermore, since humans utilize a completely different pathway for isoprenoid biosynthesis, the parasite enzymes can be targeted without causing side effects on the host.^4−6^

**Scheme 1:**
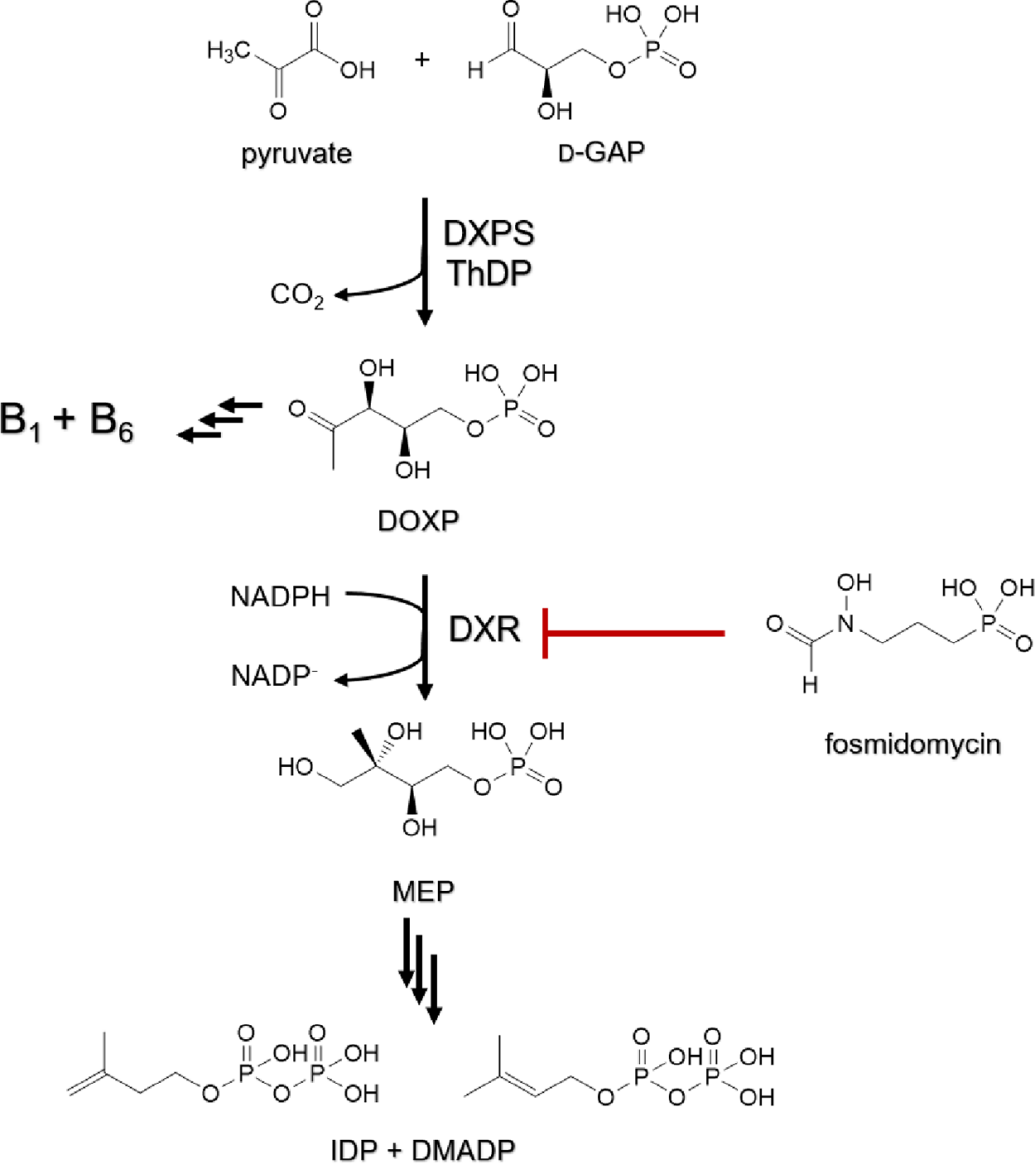
Illustration of the MEP Pathway, Highlighting the Important Branch Point Enzyme 1-Deoxy-D-xylulose 5-Phosphate Synthase d-GAP = glyceraldehyde 3-phosphate, DXPS = 1-deoxy-d-xylulose-5-phosphate synthase, B_1_ = thiamine, B_6_ = pyridoxine, DOXP = 1-deoxy-d-xylulose 5-phosphate, DXR = 1-deoxy-d-xylulose 5-phosphate reductoisomerase, MEP = 2-*C*-methylerythritol 4-phosphate, IDP = isopentenyl diphosphate, DMADP = dimethylallyl diphosphate.

The identification of fosmidomycin as a potent inhibitor of the enzyme 1-deoxy-d-xylulose 5-phosphate reductoisomerase (DXR), which is investigated in several clinical trials, nicely demonstrates the effectiveness of MEP pathway inhibitors (Scheme 1).^7^ Fosmidomycin mimics the substrate of DXR, 1-deoxy-d-xylulose 5-phosphate (DOXP), and it was shown that the hydroxamate group and the phosphonate group are essential for binding.^8^ The high polarity of fosmidomycin however greatly limits its application and leaves little room for modifications of the original structure. Its promising inhibition profile validates the effectiveness of targeting the MEP pathway, and more research is urgently needed to expand the pool of potent inhibitors. Particularly, 1-deoxy-d-xylulose-5-phosphate synthase (DXPS) attracted our attention. The rate-limiting, first enzyme in the pathway catalyzes the condensation of pyruvate and glyceraldehyde 3-phosphate (d-GAP) and concomitant decarboxylation with thiamine diphosphate (ThDP) as a cofactor. A unique advantage over the other MEP enzymes is that by targeting DXPS, both the production of isoprenoid precursors and the biosynthesis of the vitamins B_1_ and B_6_ are inhibited effectively.^9−11^

In our previous efforts to identify inhibitors of *M. tuberculosis* (*Mt*)DXPS, we found three promising compound classes.^12^ Superposition of the crystal structure of *Mt*DXPS (Protein Data Bank-ID (PDB): 7A9H) and a homology model of *P. falciparum* (*Pf*)DXPS showed a similar structure with several loops in *Pf*DXPS that are not present in *Mt*DXPS (Figure S1). However, a closer look at the active site revealed high conservation of the essential amino acids between *Mt*DXPS and *Pf*DXPS (Figure S2), which suggested that active compounds against *Mt*DXPS may be effective against *Pf*DXPS.^13,14^

## Results and Discussion

### Structure–Activity Relationship (SAR) of Hit Compounds

**Figure 1:**
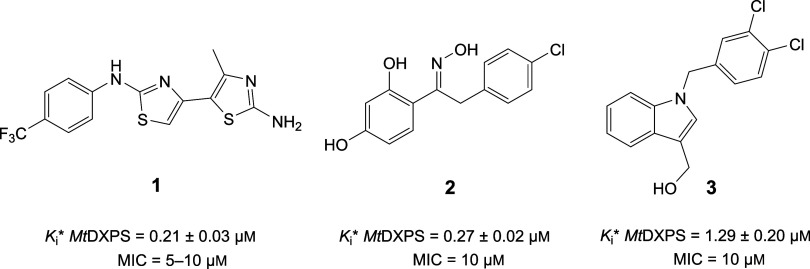
Ligand-based virtual screening hits (**1**, **2**, **3**) were tested against *M. tuberculosis* DXPS. Minimum inhibitory concentrations (MICs) were determined against the *Mycobacterium tuberculosis* H37Rv strain.

In our previous work, we used ligand-based virtual screening (LBVS) as a powerful tool to identify new inhibitors based on known reference compounds for the target of interest.^12^ LBVS relies solely on the use of descriptors of molecular structures and properties to compare various molecules and does not require crystallographic data.^15^ As there were no suitable known inhibitors that could directly initialize the LBVS campaign against *Mt*DXPS, we used pseudoinhibitors as initial ligands (we combined the concepts of pseudoreceptors (receptor structure designed based on structure of true inhibitors) and pseudoligands (virtual inhibitors that have not been tested against the target) to develop pseudoinhibitors (inhibitors of a close homologue with no target activity are used to define key anchor points and pharmacophores that overlap with the target of interest as starting points for LBVS)), with validation of key pharmacophores on the homologue *Deinococcus radiodurans* (*Dr*)DXPS, a model enzyme for *Mt*DXPS. We selected three compounds from a previous project, one ThDP analogue and two inhibitors from a *de novo* fragment design campaign.^16^ Their 3D shape was generated and compared to all commercially available compounds from the Princeton database.^17^ After each round of LBVS, all compounds were tested on *Dr*DXPS, as well as *Mt*DXPS. We identified three promising, structurally diverse hit classes. The most active compound in each class inhibited *Mt*DXPS in a slow-, tight-binding pattern, with submicromolar Morrison inhibition constants (*K*_i_*) between 0.2 and 1.3 μM and showed promising minimum inhibitory concentrations (MICs) of 5–10 μM against *M. tuberculosis* (Figure 1).

**Figure 2:**
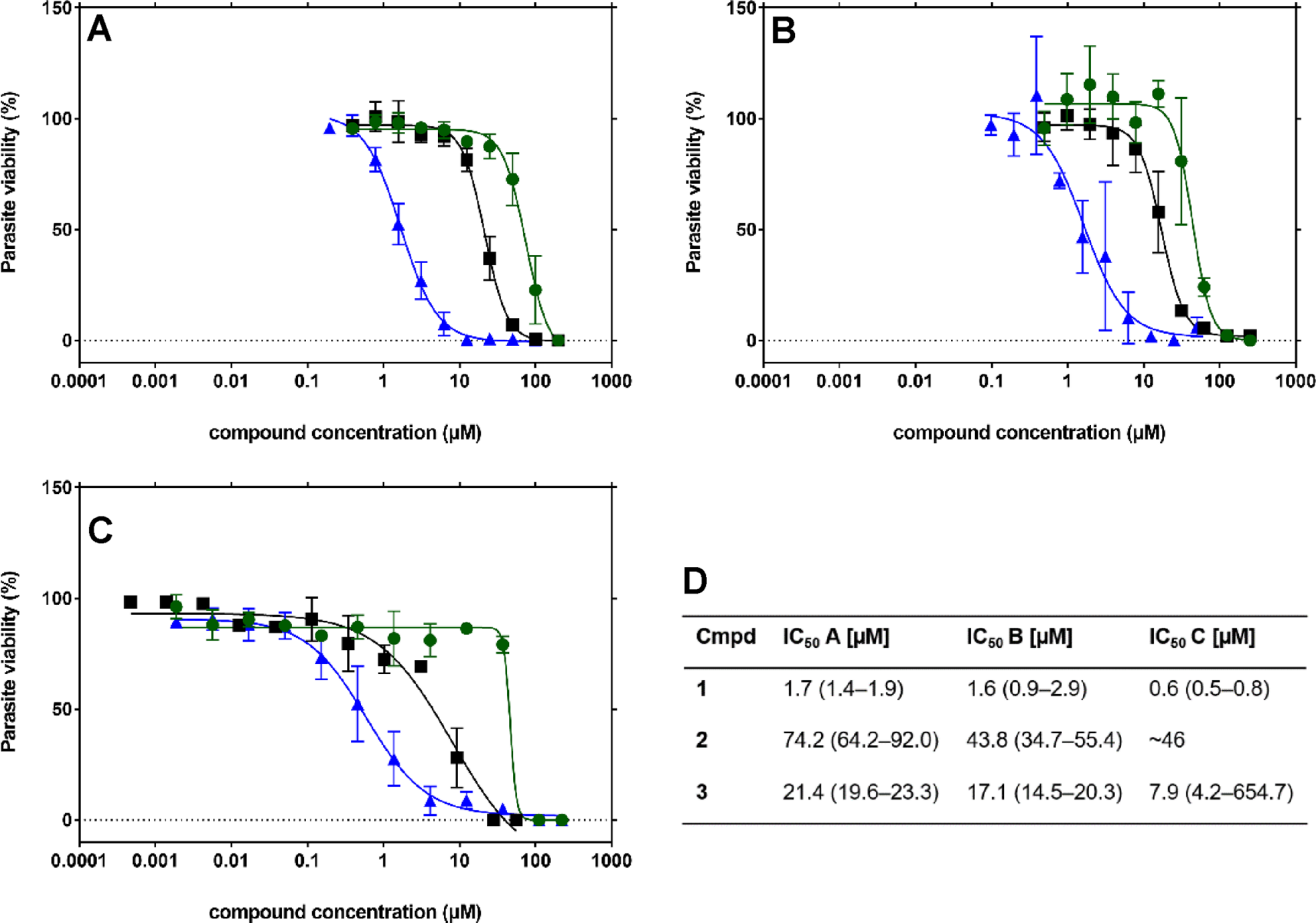
Whole-cell antiplasmodial activity of DXPS compounds against *Plasmodium falciparum* 3D7 from three different laboratories and assays; following (A) method I, (B) method II, and (C) method III. IC_50_ curves of **1** (blue triangles), **2** (green circles), and **3** (black squares). All data were averaged from two to four independent experiments conducted in duplicate or triplicate and is shown including SD (error bars). (D) For IC_50_ determination, data were analyzed using nonlinear regression of the log-dose–response curves and interpolated from the sigmoidal curve. 95% CI is displayed as error measure. Please note that in Table 1, Table 2, and 3 the inhibition values from method III differ slightly as they have been calculated with a different program (see Methods section). As they are within the 95% CI, we have decided not to recalculate the values.

Despite ongoing efforts, we have no *Pf*DXPS enzyme for on-target testing available and, therefore, the antimalarial activity was evaluated against cultured blood-stage *P. falciparum* 3D7. Several compounds were tested in three different laboratories on three continents under varying experimental conditions, but in all cases, the data were similar (methods I–III). Compound **1** shows the most notable variation with a half-maximal inhibitory concentration (IC_50_) between 0.6 and 1.7 μM (Figure 2). Compounds **2** and **3** vary between 44 and 74 and 8–21 μM, respectively, which gives us great confidence in our results. All compounds were additionally tested on NF54 (method II), a chloroquine-sensitive strain, and on Dd2 (method III), a chloroquine-resistant strain (Table S6). The differences in inhibition were small, indicating a different mode of action than chloroquine for all three compound classes, suitable for treatment of chloroquine-resistant strains. To the best of our knowledge, compounds **1**–**3** represent promising hits as novel antimalarials.

We synthesized several derivatives of all three classes to see if we could improve potency and achieve favorable cytotoxicity and metabolic stability properties. Here, we only discuss the measurements on 3D7 that were performed with all compounds in more detail (using method III).

#### Oximes

**Scheme 2:**
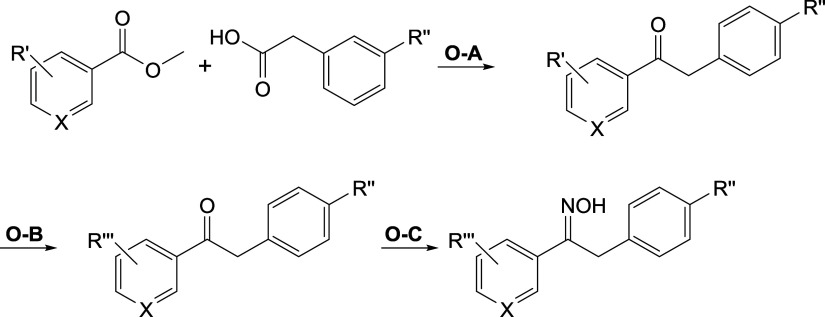
General Reaction Scheme for the Synthesis of Oximes **O-A**: sodium *bis*(trimethylsilyl)amide (4.0 equiv), dimethylformamide, −10 °C, 3–72 h. **O-B**: BBr_3_ (10.0 equiv), dichloromethane, 25 °C, 5 h. **O-C**: KOAc (3.0 equiv), [NH_3_OH]Cl (1.5 equiv), reflux, 2 h. R′ = OMe or H. R″ = Cl. R‴ = OMe or H or OH. X = C or N.

The synthesis of the oximes followed general procedures **O-A** to **O-C** or a selection thereof (Scheme 2). First, a methyl benzoate with methoxy group(s) as R′ and a phenylacetic acid were condensated to form ketone intermediates with sodium *bis*(trimethylsilyl)amide. When necessary, the methoxy groups were reduced using procedure **O-B** to form the free hydroxyl groups with boron tribromide. The last step was the oxime formation with potassium acetate and hydroxylammonium chloride. If alternative routes were taken, the corresponding synthetic schemes can be found in Schemes S1 to S4.

In derivatives for compound **2**, we maintained the two aromatic rings, connected by a two-carbon linker, but changed the substituents on the rings as well as the oxime functionality (Table 1). To improve solubility, we replaced the chlorine with an amino group (compounds **4**, **5**, and **6**). While these modifications improve solubility 2-fold to ∼200 μM (Table S1) for compounds **4** and **5,** the activity for all three compounds is lost. Replacing the Western aromatic ring with a 2-methoxy pyridine (compounds **7***E* and **7***Z*) or a 2-hydroxy pyridine (although in water the 2-pyridone is probably the dominant tautomer) neither improves the activity for the *E*- nor the *Z*-isomer (compounds **8***E* and **8***Z*). Methylation of one of the hydroxyl groups (compound **9**) increases the activity 2-fold (47 μM). Compound **10**, where in comparison to **2** a NH_2_– is replacing a hydroxyl group, is the most active oxime against *P. falciparum* with an IC_50_ value of 38 ± 2 μM. Although we increased the activity 2-fold with compound **10**, replacing the oxime moiety with an imine (**11**) or a hydrazone (**12**) improves activity. Replacing the oxime with an alcohol group (**13**) leads to a ten-fold increase (IC_50_ = 10 ± 2 μM) in comparison to the parent compound **2**.

**Table 1:**
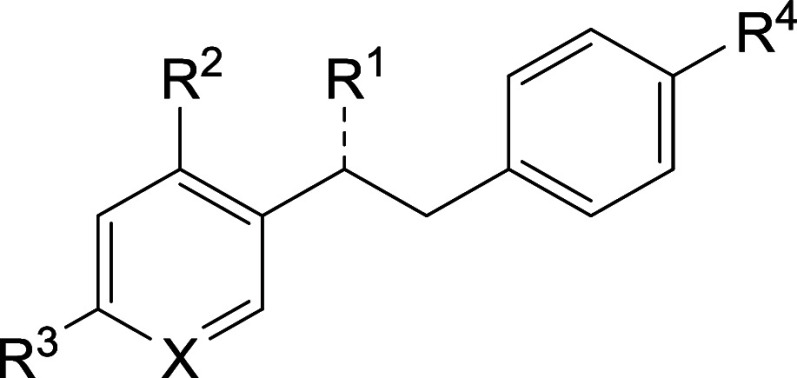
Inhibition Data for All Oxime Derivatives^a^

**ID**	**R**^**1**^	**R**^**2**^	**R**^**3**^	**R**^**4**^	**X**	**IC**_**50**_**[μM]**
**4**	=NOH	OMe	OH	NH_2_	C	>111
**5**	=NOH	OH	OH	NH_2_	C	>111
**6**	=NOH	OMe	OMe	NH_2_	C	>111
**7***Z*	=NOH	H	OMe	Cl	N	>111
**8***E*	=NOH	H	OH	Cl	N	>111
**8***Z*	=NOH	H	OH	Cl	N	>28
**2**	**=NOH**	**OH**	**OH**	**Cl**	**C**	**94 ± 8**
**9**	=NOH	OH	OMe	Cl	C	47 ± 15
**7***E*	=NOH	H	OMe	Cl	N	44 ± 10
**10**	=NOH	OH	NH_2_	Cl	C	38 ± 11
**11**	=NOMe	OH	OH	Cl	C	28 ± 9
**12**	=NNH_2_	OH	OH	Cl	C	16 ± 0
**13**	**–OH**	**OH**	**OH**	**Cl**	**C**	**10 ± 2**

a IC_50_ measured against *P. falciparum* 3D7 (see Supplementary Information method III). The original hit is compound **2** and best derivative **13** (in bold).

#### Indoles

**Scheme 3:**
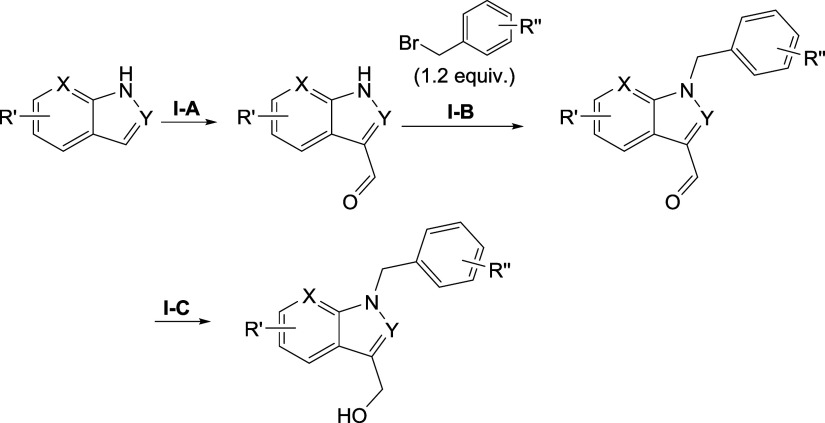
General Reaction Scheme for the Synthesis of Indole Derivatives **I-A**: POCl_3_ (1.3 equiv), dimethylformamide, 80 °C, 15 min. **I-B**: NaH (1.8 equiv), dimethylformamide, 0 °C – 25 °C, 16 h. **I-C**: NaBH_4_ (3.2 equiv), MeOH, 25 °C, 1 h. R′ = R^3^ in Table 2. R″ = H or Cl. X and Y = N or C.

The synthesis of the indole derivatives followed general procedures **I-A** to **I-C** or a selection thereof (Scheme 3). 1*H*-Indoles were substituted with POCl_3_ to from 1*H*-indole-3-carbaldehydes. In general procedure **I-B**, bromobenzenes were attached to form benzyl-substituted intermediates that were reduced to the final products using procedure **I-C** with NaBH_4_. If alternative routes were taken, the corresponding synthetic schemes can be found in Schemes S5 and S6.

Given the promising activity of indole **3**, we additionally explored different substitution patterns to gain insight into the SARs (Table 2). The most prominent difference is observed after removal of one methylene group. Replacing the C7-carbon in the indole with a nitrogen (**14**) leads to a loss of activity. The same can be observed when attaching the phenyl ring directly to the nitrogen of the indole (compound **15**). Also, a methoxy substituent in this position is not tolerated (**16**) and a nitrile substituent in position 6 (**17**) results in a two-fold decrease in comparison to the original hit **3**. Replacing the core indole with an indazole (**18**) or inserting a methylene group in the R^1^-residue (**19**) does not result in significant changes. Removing the chloro-substituents gives a first interesting difference. If only the 3-chloro substituent is removed (**20**), the activity stays the same, but removal of the 4-chloro substituent (**24**) or removal of both substituents (**25**) leads to a two-fold increase in activity. Interestingly, the complete removal or only removal of the hydroxyl group of the R^1^-substituent leads to IC_50_ values of ∼8 μM (**28**) and ∼10 μM (**26**), respectively. We have shown previously that this group is essential for binding to *Mt*DXPS, which suggests a different mode of inhibition in *P. falciparum*.^12^ Mostly, substituents on the indole core are tolerated. While a 7-methoxy substituent is not tolerated as mentioned before, the electron-withdrawing 7-chloro group increases the activity to 13 μM (**23**). A methoxy substituent in position 4 is favorable (**31**), but moving it to position 5 (**21**) or removing one chlorine of the R^2^-group (**22**) reduces the activity again. A 5-nitro-substituent (**29**) or 5-bromo- (**30**) results in IC_50_ values below 10 μM, as do a 6-fluoro (**27**) and 5-fluoro (**32**) substituent; only a fluorine in four-position affords an activity close to the nanomolar range (**33**). Since many compounds show an activity between 2 and 20 μM, these subtle changes do not have a substantial impact. To make a more significant change, we tested a bulky substituent in the R^1^-position and determined an IC_50_ value of 800 ± 200 nM for compound **34**.

**Table 2:**
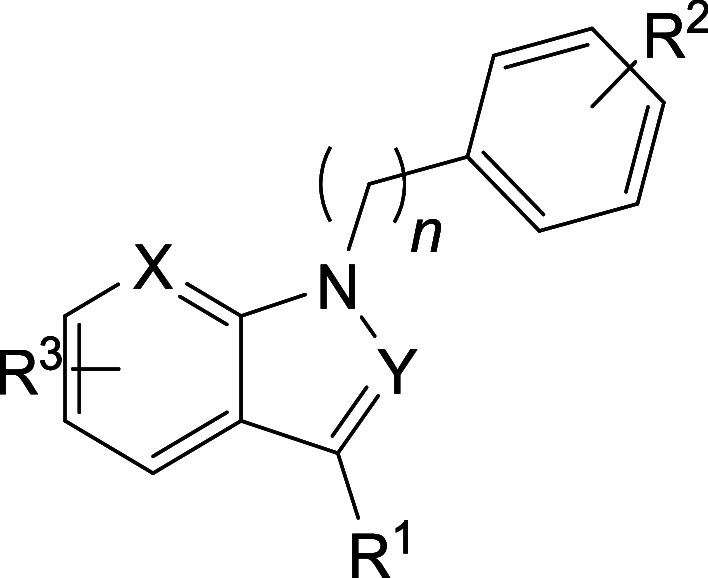

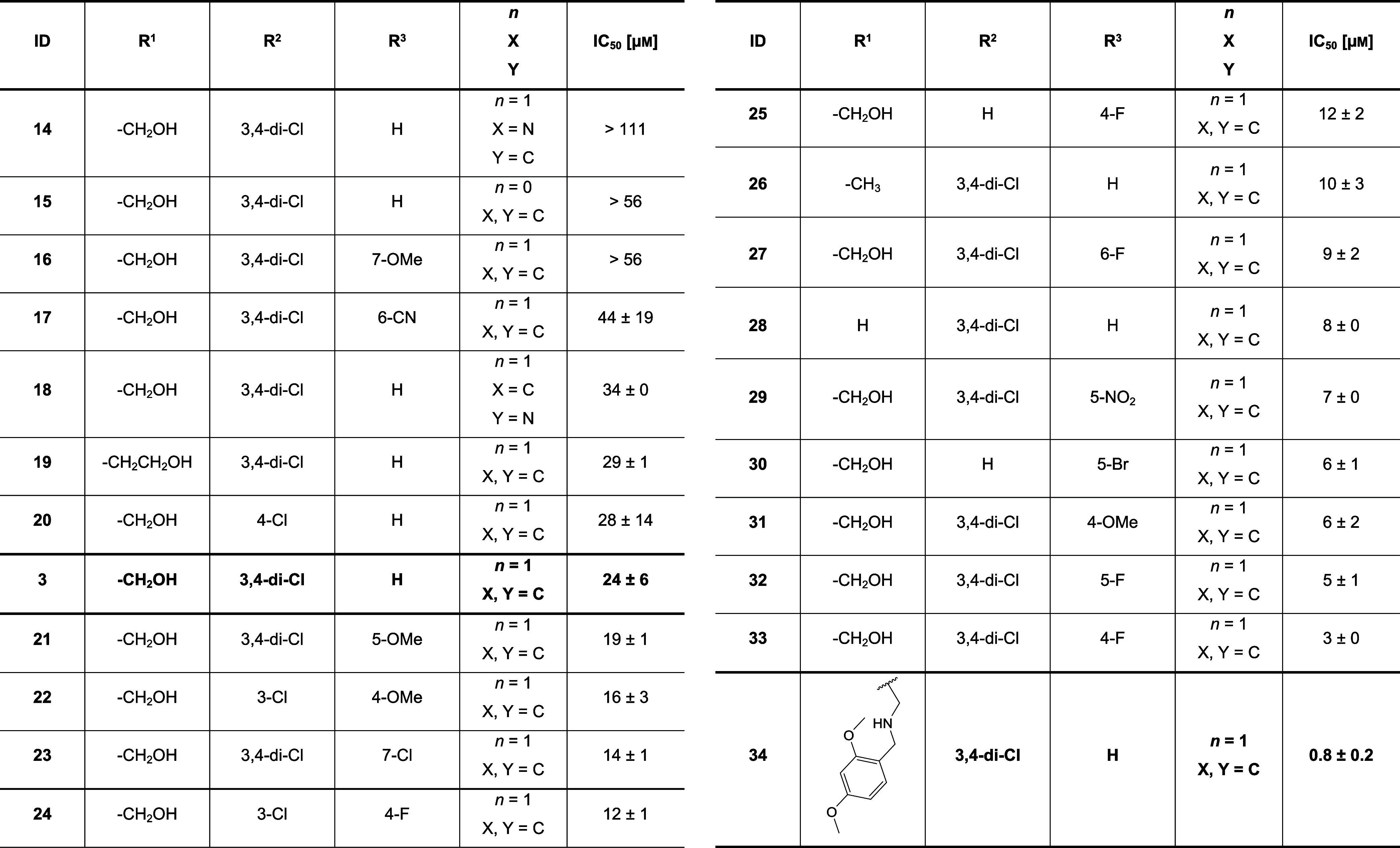
Inhibition Data for All Indole Derivatives^a^

a IC_50_ values measured against *Plasmodium falciparum* 3D7 (see the Supplementary Information method III). Original hit is compound **3** and best derivative **34** (in bold).

#### Aminothiazoles

**Scheme 4:**
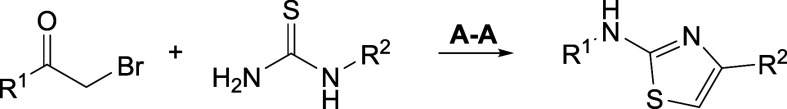
General Reaction Scheme for the Synthesis of Aminothiazoles **A-A**: diisopropylethylamine (1.1 equiv), ethanol, 25 °C, 72 h.

The synthesis of the indole derivatives followed general procedure **A-A**. A mixture of α-bromoketones and substituted thioureas was stirred in ethanol with diisopropylethylamine to form the final aminothiazoles (Scheme 4).

For the most promising class, the aminothiazoles, we investigated many derivatives with substantial changes on both ends of the molecule (Table 3). We did not observe a severe reduction in activity, which indicates that the central core is essential. Changing the position of the CF_3_ group on the left side of the original molecule **1** results in a lower activity for 3-CF_3_-aminothiazole **45** (3 μM) and 2-CF_3_-aminothiazole **35** (43 μM). Modification of the right part of the molecule to a 2-pyridyl ring affords single-digit micromolar activities when the left part of the molecule is either a phenyl ring with 2,5-dimethyl- (**43**), a 3,4-dimethyl- (**46**), or a 3-chloro-2-methyl- substitution (**44**). A 4-pyridyl ring does not show the same trend, and the activity drops to 34 μM (**36**). A 2,4-dihydroxyphenyl ring (**37**, **38**) on the right side of the molecules is not tolerated. Utilizing a 3,4-dihydroxyphenyl on the right side and a 3,4-dimethylphenyl on the left side, however, results in an IC_50_ value of 4 μM (**42**). When keeping the second aminothiazole ring on the right side, modifications on the left ring with 3-methoxy- (**39**) or 3-chloro-2-methyl-substituents (**40**) do not improve the activity. Simply replacing the NH_2_ group on the right side with a methyl group, the activity drops 10-fold to ∼10 μM (**41**), which shows the importance of this group. Another promising compound, **47**, with a 4-chloro-pyridine on the left side of the molecule, has a similar potency as **1** (IC_50_ = 1 μM). Overall, the original hit compound **1** was the most active with an IC_50_ of 600 nM.

**Table 3:**
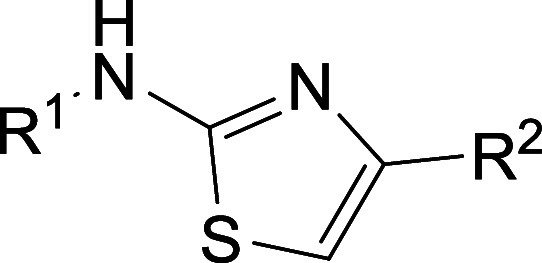

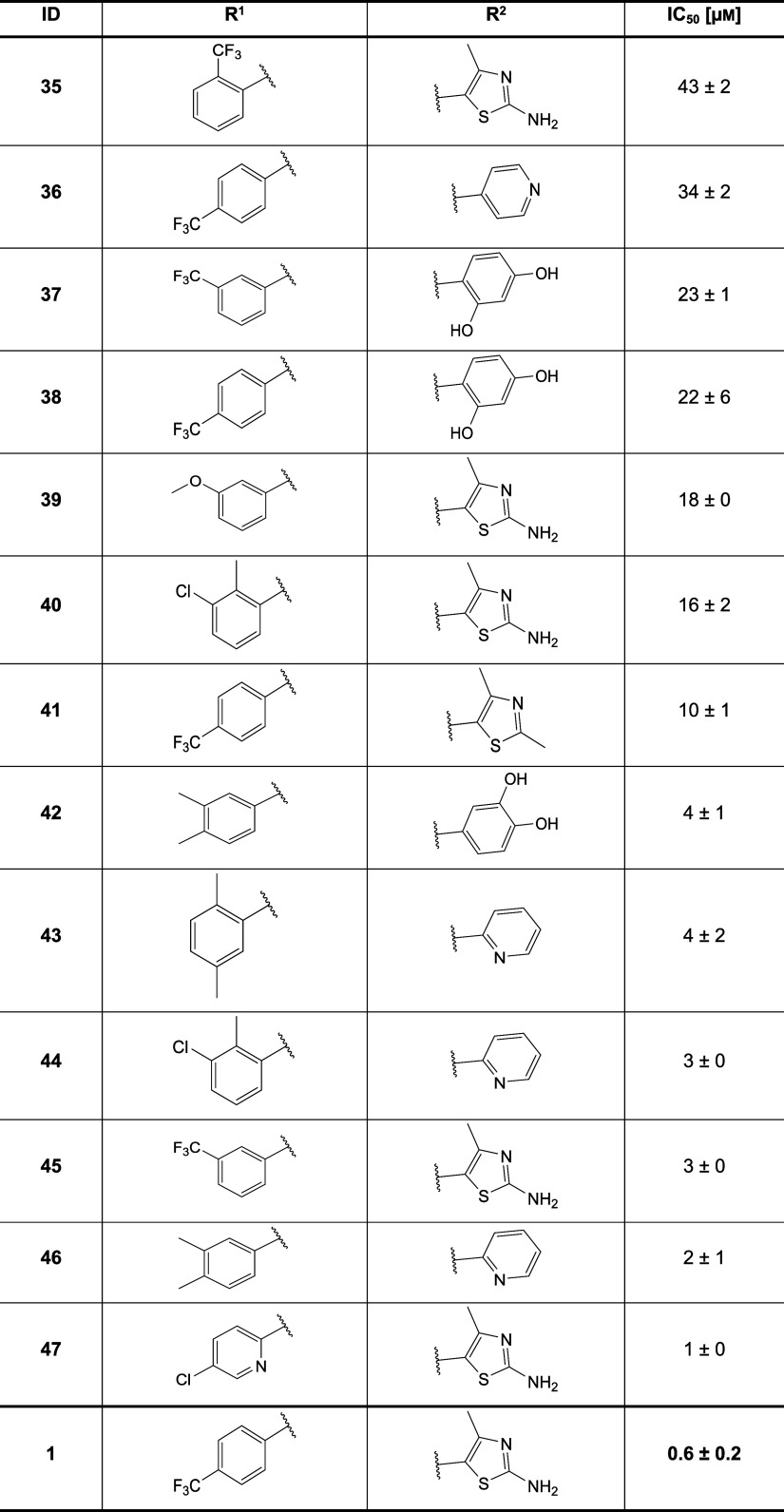
Inhibition Data for All Aminothiazole Derivatives^a^

a IC_50_ values measured against *Plasmodium falciparum* 3D7 (see the Supplementary Information method III). The original hit is compound **1** (in bold).

### Cytotoxicity and Metabolic Stability of Selected Compounds

The promising results in culture motivated us to thoroughly investigate the properties of all three hit classes further. We tested them for their respective cytotoxicity and metabolic stability (Table 4). Indole **3** exhibits an IC_50_ at 23.6 ± 6.4 μM against 3D7. Evaluating its cytotoxicity against human hepatocytes (Hep G2) results in a lower IC_50_ value of 0.8 ± 0.2 μM, suggesting that the compound lacks specificity against asexual parasites. The two most active indole derivatives on *P. falciparum*, **33** and **34**, are equally potent against Hep G2 cells (0.79 ± 0.37 μM and 90% inhibition at 10 μM, respectively). When investigating the metabolic stability, we found that indole **3** has a half-life of only 10 min in human liver S9 fractions. More synthetic work is needed to balance these properties. While oxime **2** is active against *P. falciparum* (IC_50_ = 93.8 ± 8.0 μM), it is similarly potent against Hep G2 (>50 μM). The best oxime hit **10** is two-fold more active against *P. falciparum* but equipotent against Hep G2 cells. After removing the oxime functionality and replacing it with a hydroxyl group, we found that compound **13** not only is the most active oxime derivative (IC_50_ = 9.9 ± 1.7 μM) but also showed no cytotoxicity towards Hep G2 cells at 100 μM. With the synthetic modifications, we improved the metabolic stability from 28 min for compound **2** to 98 min for alcohol **13**. We therefore excluded the indole and oxime class from further investigations, but compound **13** emerged as a new potent inhibitor class for *P. falciparum* with an improved cytotoxicity and metabolic stability profile.

**Table 4:** Summary of *P. falciparum* Activity, Cytotoxicity (Activity against Hep G2 in μM or % Inhibition), and Metabolic Stability in Human Liver S9 Fraction (Half-Life in min) of Selected Compounds from All Three Hit Classes^a^

**compound**	**IC**_**50**_*P. falciparum***3D7 (μM)**	**IC**_**50**_**Hep G2**	*t*_**1/2**_**(min)**
**1**	0.6 ± 0.2	>50 μM	>240
**2**	94 ± 8	>50 μM	29 ± 0.7
**10**	38 ± 11	50–100 μM	56 ± 0.4
**13**	10 ± 2	>100 μM	98 ± 10
**3**	24 ± 6	0.8 ± 0.2 μM	10 ± 0.4
**33**	2 ± 0	0.79 ± 0.37 μM	n.d.
**34**	0.8 ± 0.2	at 10 μM 90 ± 2 % inhibition	n.d.

a n.d. = not determined.

Compound **1** exhibits a promising IC_50_ value (IC_50_ = 0.6 ± 0.2 μM) in addition to a weaker inhibition of Hep G2 with 54.0 ± 2.4 μM. It is also the most stable compound in the human liver S9 fraction (>240 min) that we tested. As such, aminothiazole **1** is a potent hit and inhibitor of *P. falciparum* growth in culture. It will be further optimized against *P. falciparum* and *M. tuberculosis* in parallel.

### Docking Analysis of Most Potent Aminothiazoles

In an effort to understand the binding mode of selected compounds, we docked them into the AlphaFold-predicted structure of *Pf*DXPS. The results for the oxime and indole class can be found in the Supporting Information (Figures S3–S7).

**Figure 3:**
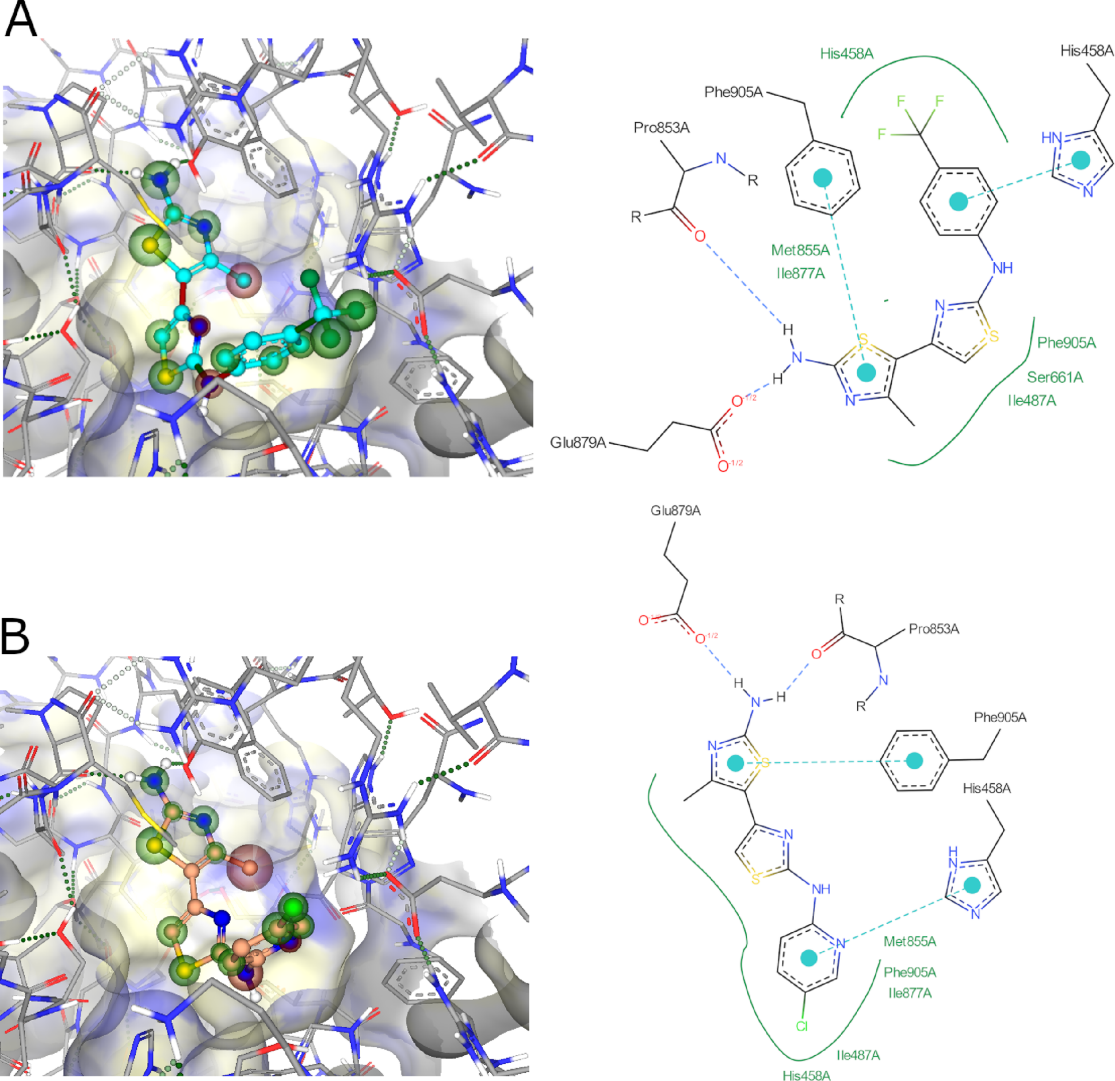
(A) Binding mode and docking pose diagram of compound **1**. (B) Binding mode and docking pose diagram of compound **47**. Protein surface representation is clipped for clarity. Green and red spheres represent positive or negative contribution to the predicted affinity, respectively.

In the aminothiazole series, compound **1** was predicted to bind in the ThDP site, forming H-bonds with Glu879 and Pro853 (Figure 3). Similar interactions were observed for compound **47**, with lower predicted affinity matching experimental results. The CF_3_ group in compound **1** is predicted to play a significant role in binding affinity compared to the chlorine in compound **47**.

### Attempt at Target Validation

We have strong reason to believe that DXPS is the main target of all hit compounds in *M. tuberculosis*.^12^ To confirm *Pf*DXPS as a target in *P. falciparum*, we chose to further investigate compound **1**, since it showed the most promising activity and the docking results were supporting our hypothesis. One well-established method is a rescue assay with IDP, the product of the MEP pathway. It has been shown that the addition of IDP to blood-stage *Plasmodium* spp. rescued parasite survival after treatment with fosmidomycin (FSM), which inhibits the second enzyme in the MEP pathway, as well as survival of apicoplast-minus *Plasmodium* spp.^3^ We determined the growth inhibition of blood-stage *P. falciparum* with selected compounds, including the most active compound **1**, in the presence and absence of 125 μM IDP. This assay has never been performed with a DXPS inhibitor, but we expected a rescue effect if DXPS was the main target of compound **1**.

While, as expected, the antiparasitic activity of FSM was rescued by IDP addition, compound **1** still inhibited *P. falciparum* growth under IDP supplementation. Similar results were observed for two other active aminothiazole derivatives, **46** and **47**, and the indole derivative **33** (Figures S7 and S8 and Table S3). Together, these data suggest that our compounds inhibit additional pathways within *P. falciparum* apart from the MEP pathway or does not inhibit DXPS.

In order to elucidate whether **1** interacts with DXPS, we profiled the cellular concentration of MEP pathway intermediates by LC-MS. In this experiment, we analyzed the concentration changes of MEP pathway metabolites in the presence and absence of **1** in *P. falciparum* and *Escherichia coli*. If a compound inhibits the MEP pathway, a reduced concentration of all metabolites downstream of the inhibited enzyme is expected. In *E. coli*, however, all downstream metabolites of DXPS are increased upon inhibitor treatment, while the pyruvate concentration drops (Figure S9). This behavior has not previously been reported, but it suggests an influence on the pathway that requires further investigation. In *P. falciparum*, we observe no difference in metabolite concentrations but a reduction in pyruvate levels (Figure S10 and Table S4). This decrease is consistent with a reduction in tricarboxylic acid (TCA) cycle metabolites (Figure S11 and Table S4), but since pyruvate is tied to many other metabolic pathways, we could not determine the reason for the decrease. Although these results did not confirm our hypothesis, they indicated that a different mode of action in *P. falciparum* and *E. coli* is responsible for the anti-infective activities of **1** in culture.

To address the ambiguous result from the previous assays, we screened the three original LBVS hits against transgenic parasites overexpressing thiamine pyrophosphokinase (*Pf*TPK) and *Pf*DXPS (DOXP cell line) and compared the results to the MOCK cell line that contained only the transfected vector backbone. All three hit compounds are ThDP-competitive inhibitors, as we have shown previously.^12^ Therefore, it is possible that the compounds indiscriminately bind to *Pf*TPK as well as *Pf*DXPS. In case of inhibition, we would expect a higher IC_50_ value for the overexpressing cell lines than for the MOCK cell line, but a statistical difference (Table S5) was not detected for either *Pf*DXPS (Figure S12) or *Pf*TPK (Figure S13). While these results leave the compound’s main target unclear, we can exclude *Pf*TPK as an off-target.

### PolyPharmacology Browser (PPB) Analysis to Identify Alternative Targets

Despite extensive efforts to experimentally verify DXPS as the molecular target, the results were not conclusive. To extend our knowledge of other possible targets and off-target proteins, we turned to an *in silico* approach using the PPB to search for other potential target enzymes. The PPB search engine employs a similarity-based approach following the idea that similar compounds should target the same proteins.^18,19^ Several methods are used in parallel to calculate molecular fingerprints of a query compound, which are then used to search the open-access bioactivity database ChEMBL.^20^ The search output contains compounds similar to the query molecule, associated with their biological activity. We manually analyzed the results, looking for alternative targets of our compounds. The results for the analysis of the oxime and indole classes can be found in the Supporting Information.

#### Aminothiazoles

For all 15 queried aminothiazole molecules, we could find related compounds that are reported to target *P. falciparum*. The majority of the reported activities however are based on cell-based assays without an assigned molecular target. Only the compounds with a known target are further analyzed below.

**Figure 4:**
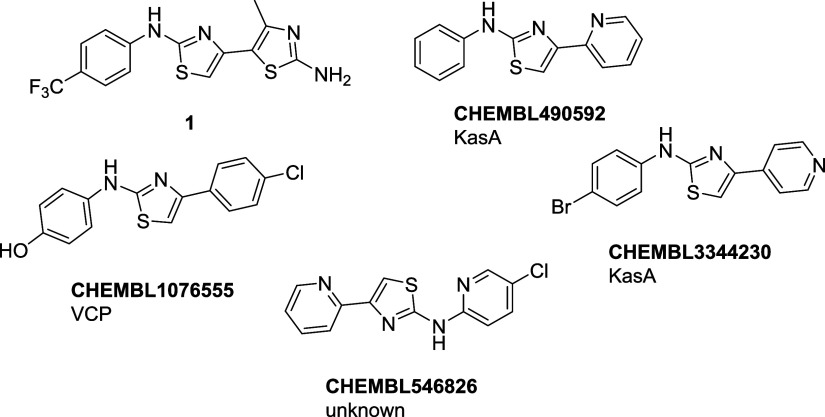
Aminothiazole hit compound **1** and similar compounds we found during the PolyPharmacology Browser search, with their reported target. KasA = β-ketoacyl ACP synthase; VCP = valosin-containing protein.

With the aminothiazole **CHEMBL490592** (Figure 4), we found a compound similar to our herein described aminothiazole class. It is part of a different group of aminothiazole-based inhibitors with activity against *M. tuberculosis*.^21,12^ In a previous study, we could also observe antitubercular activity for our group of aminothiazoles. In 2014, Makam and Kannan reported that a possible target of 2-aminothiazoles in *M. tuberculosis* is the enzyme β-ketoacyl ACP synthase (KasA).^22^ The KasA protein is part of the FAS-II pathway and involved in the biosynthesis of mycolic acid, an essential cell wall component. It is also present and active in *P. falciparum*, but it was shown that the blood stage of *P. falciparum* does not require the FAS-II pathway for proliferation.^16−18,23−25^ Therefore, inhibition of KasA cannot explain the activity we observed in the blood-stage assays. However, it could be beneficial for a new antimalarial drug to inhibit KasA as a second target.^18^

In addition, a series of aminothiazoles have been developed as antileishmanial agents, a protozoan parasite, despite no identified target protein.^26^ Based on the structural similarity of their reported best hit **CHEMBL546826** to our aminothiazoles, it seems likely that KasA may be the target, which is also present in *Leishmania* spp.

We identified compound **CHEMBL1076555** (Figure 4), which was included in a series of anti-cancer agents targeting the valosin-containing protein (VCP).^27^ Furthermore, the endoplasmic reticulum-assisted degradation (ERAD) is also gaining attention as a target against protozoan pathogens, as they have only a very small network for their protein quality control system.^28^ One of these members is VCP, which might be targeted by the reported aminothiazoles.^29^ As a consequence of this pared-down network of protein degradation, *P. falciparum* is highly susceptible to ERAD inhibitors, a VCP-knockout strain that is not viable. Although VCP is also present in mammals (often termed p97), it was shown that selectivity toward VCP from *P. falciparum* can be achieved.^28,30^ Testing our hits as well as the previously reported VCP inhibitors against *P. falciparum* might reveal VCP as an additional target, which could explain the inhibitory activity we observed in the blood-stage assays.^27^

#### Human Off-Targets

During the search for antimicrobial targets, we found mammalian enzymes that are affected by compounds similar to our hits, including enzymes of the eicosanoid metabolism, the membrane protein KDR kinase and RNA polymerase II (Table S7).^31−33^ These early data are beneficial, as we now have the possibility to develop and test our hits for selectivity for the bacterial over the human targets. Furthermore, if we can confirm activity against human enzymes, it is possible that some derivatives could be repurposed. For example, we report three compounds (**3**, **15**, **20**) that are identical to derivatives of the oncrasin-1 inhibitor of the RNA polymerase II, which is discussed as a new class of anticancer drugs.^33^

## Conclusions

We showed that our previously identified small-molecule inhibitors against *M. tuberculosis* DXPS from an LBVS campaign are also active against several *P. falciparum* strains. We successfully improved the activity of two of our three initial hit classes. Oxime **2** was improved 2.5-fold from 94 to 38 μM by replacing one hydroxyl group with an amino group (compound **10**). By removing the oxime, we also identified several new compounds that require further investigation. Imine **11**, hydrazone **12**, and alcohol **13** are promising alternative functionalities to oxime **2** with low cytotoxicity and improved metabolic stability in the case of compound **13**. The best hit of the indole class, compound **3** with an IC_50_ value of 24 μM, was improved 30-fold to 0.8 μM by adding a bulky substituent on the hydroxyl group (**34**). We discontinued this class due to the toxicity issues. Overall, we identified the aminothiazole **1** as a promising compound against *P. falciparum* with good activity, selectivity, and excellent metabolic stability.

Using compound **1**, we investigated *Pf*DXPS as a potential target. First, docking studies show binding in the active site of *Pf*DXPS. However, IDP failed to rescue growth after treatment. LC-MS analysis of metabolites downstream of DXPS following inhibition in both *P. falciparum* and *E. coli* was ambiguous. In *P. falciparum*, we observe a reduction in pyruvate levels that is consistent with the downregulation of TCA-cycle metabolites indicating complex effects on parasite metabolism. To further investigate potential interaction with *Pf*DXPS, we used genetically modified parasites overexpressing the enzyme. Here, we cannot observe any significant difference between the MOCK and the overexpressing cell lines. Although the two homologues *Mt*DXPS and *Pf*DXPS are highly similar, DXPS is not the target of compound **1** in *P. falciparum*.

The validation of molecular targets is notoriously difficult. Using the PPB search engine, we identified two potential targets for the aminothiazole hit class, the enzymes KasA and VCP. The KasA enzyme is part of the FAS-II pathway. However, as FAS-II inhibition is not essential for proliferation in the *P. falciparum* blood stage, it cannot explain the observed asexual growth inhibition suggesting additional targets. Whether our hits are dual inhibitors will need to be determined experimentally in future studies. Antimalarial drugs on the market such as artemisinin and chloroquine also have multiple targets, which makes them highly potent, but emerging resistance increases the need for alternative treatments.^34,35^ We showed that the discussed compounds, in particular the aminothiazoles, effectively kill the chloroquine-resistant *P. falciparum* strain Dd2, which makes them suitable candidates for further investigation as alternative drugs or combination partners with existing therapeutic agents.

## Methods

### General Procedures

NMR experiments were run on a Bruker Avance NEO 500 MHz (^1^H at 500.0 MHz; ^13^C at 126.0 MHz; ^19^F-NMR at 470 MHz), equipped with a Prodigy CryoProbe. Spectra were acquired at 298 K, using deuterated dimethyl sulfoxide ((CD_3_)_2_SO, ^1^H: 2.50 ppm, ^13^C: 39.52 ppm), deuterated methanol (CD_3_OD, ^1^H: 3.31 ppm, ^13^C: 49.00 ppm), or deuterated chloroform (CDCl_3_, ^1^H: 7.26 ppm, ^13^C: 77.16 ppm) as solvents. Chemical shifts for ^1^H and ^13^C spectra were recorded in parts per million (ppm) using the residual nondeuterated solvent as the internal standard. Coupling constants (*J*) are given in Hertz (Hz). Data are reported as follows: chemical shift, multiplicity (s = singlet, d = doublet, t = triplet, q = quartet, m = multiplet, br = broad, and combinations of these) coupling constants and integration. Mass spectrometry was performed on a Spectra Systems MSQ LC-MS system (Thermo Fisher, Dreieich, Germany). Purification of the final products was performed using preparative HPLC (Dionex UltiMate 3000 UHPLC+ focused, Thermo Scientific) on a reversed-phase column (C18 column, 5 μM, Macherey-Nagel, Germany), or flash chromatography was performed using the automated flash chromatography system CombiFlash Rf+ (Teledyne Isco, Lincoln, Nebraska, USA) equipped with RediSepRF silica columns (Axel Semrau, Sprockhövel, Germany). High-resolution mass spectra (HRMS) of final products were determined by LC-MS/MS using the Thermo Scientific Q Exactive Focus Orbitrap LC-MS/MS system. The purity of the final compounds was determined using the Dionex UltiMate 3000 HPLC system (Thermo Fisher Scientific). Chromatographic separation was performed on an EC 150/2 NUCLEODUR C18 Pyramid (3 μm particle size) analytical column (Macherey-Nagel). The mobile phase consisted of solvent A (water containing 0.1% formic acid) and solvent B (acetonitrile containing 0.1% formic acid) with a flow rate of 0.25 mL/min. All final compounds had a purity >95%. Yields refer to analytically pure compounds and have not been optimized. All chemicals were purchased at Sigma-Aldrich or comparable commercial suppliers and used without further purification.

#### General Remarks about the Analysis

Full characterization is provided for final compounds that have not been published before or have been published in different NMR solvents. All compounds that were used in our original LBVS work as well as in this manuscript are from the same synthetic batch. The synthesis and characterization of oximes **2**, **9**, **10**, and **12** of indoles **3**, **16**, **17**, **20**, and **32** and of aminothiazoles **1**, **35**, **37**, **45**, and **47** were previously reported by us or others.^12,33^ The identity of intermediates was determined by ^1^H NMR, ^13^C NMR, and ^19^F-NMR if applicable. The ^13^C NMR signals are doublets in the case of six carbons in the F-substituted aromatic ring in **27**, seven carbons including −**C**CH_2_OH for **24**, and eight carbons including −**CC**H_2_OH for **33** and **25**. Many indole HR-ESI-MS measurements give [M-18], due to fragmentation during ionization. The identity of *E*/*Z* isomers was determined by 1D NOESY NMR for compounds *E*-**7** and *Z*-**7**. All other compounds with isomers were assigned based on carbon shifts. *E*-oxime C7-shift (ppm) > *Z*-oxime C7-shift (ppm). All compounds are >95% pure by HPLC analysis.

##### Synthesis and Characterization of Oximes

2-(4-((*tert*-Butoxycarbonyl)amino)phenyl)acetic acid (**48**) was synthesized following a literature procedure, and all data were consistent with the reported values.^36^

##### Condensation (Procedure **O-A**)

Synthesis of the protected ketone intermediate followed a previously reported procedure.^37^ To a Schlenk flask, methyl benzoate and phenylacetic acid (1.0 equiv) were added and dissolved in dry dimethylformamide (DMF) under nitrogen. The yellow solution was cooled to −10 °C, and then sodium *bis*(trimethylsilyl)amide (2 M in tetrahydrofuran (THF), 4.0 equiv) was added dropwise under stirring. After full conversion of the starting material (3–72 h) monitored by thin layer chromatography (TLC) or LC-MS, the reaction was terminated by adding saturated aqueous NH_4_Cl solution and concentrated *in vacuo* to remove DMF. Subsequently, the residue was extracted with ethyl acetate (3×), and the combined organic layers were washed with saturated aqueous NaCl solution. The organic layer was dried over Na_2_SO_4_, filtered, and concentrated *in vacuo*.

##### Deprotection (Procedure **O-B**)

The deprotection reaction followed a previously reported procedure.^38^ To a solution of the condensation product from procedure **O-A** in dry dichloromethane (9 mL) under nitrogen, boron tribromide was added (1 M in dichloromethane, 12.0 equiv) under stirring at 25 °C. After 5 h, a saturated aqueous Na_2_CO_3_ solution was added to the solution, which was extracted with dichloromethane. The organic layer was washed with water (2×), and then dried over Na_2_SO_4_, filtered, and concentrated *in vacuo*.

##### Oxime Formation (Procedure **O-C**)

Oximation followed a previously reported procedure.^39^ To a solution of the deprotected product in methanol, potassium acetate (3.0 equiv) and hydroxylamine hydrochloride (1.5 equiv) were subsequently added under stirring. The light-yellow suspension was refluxed for 2 h. Subsequently, water was added to the mixture. The organic layer was washed with saturated aqueous NaCl solution, dried over Na_2_SO_4_, filtered, and concentrated *in vacuo*.

##### *tert*-Butyl (4-(2-(2,4-Dimethoxyphenyl)-2-oxoethyl)phenyl)carbamate (**49**)

Compound **49** was synthesized following procedure **O-A**, using **48** (650 mg, 2.6 mmol, 1.0 equiv), methyl-2,4-dimethoxybenzoate (508 mg, 2.6 mmol, 1.0 equiv), and sodium *bis*(trimethylsilyl)amide (10.4 mL, 10.4 mmol, 3.0 equiv) in dry DMF (20 mL). After 72 h, no full conversion was achieved, so it was decided to terminate the reaction. Flash column chromatography (petroleum benzine/ethyl acetate 2:1 + 1% acetic acid) afforded a mixture of the product and starting material **48**. To remove the acid, the mixture was dissolved in ethyl acetate and washed with saturated aqueous NaHCO_3_ solution (6×). The product (106 mg, 11% yield) was obtained as a white solid. ^1^H NMR (500 MHz, CD_3_OD): δ (ppm) = 7.69 (d, *J* = 8.57 Hz, 1H), 7.29 (d, *J* = 8.37 Hz, 2H), 7.08 (d, *J* = 8.57 Hz, 2H), 6.56 (m, 2H), 4.19 (s, 2H), 3.91 (s, 3H), 3.85 (s, 3H), 1.50 (s, 9H). ^13^C NMR (126 MHz, CD_3_OD): δ (ppm) = 200.8, 166.6, 162.4, 155.4, 139.0, 133.8, 131.2, 130.9, 121.5, 119.8, 106.9, 99.2, 56.1, 56.0, 50.1, 28.70. HR-ESI-MS: calculated for C_21_H_26_NO_5_ [*M*+H]^+^ 372.1805, found 372.1806.

##### 2-(4-Aminophenyl)-1-(4-hydroxy-2-methoxyphenyl)ethan-1-one (**50**)

Following procedure **O-B** using **49** (90 mg, 0.24 mmol, 1.0 equiv) and boron tribromide (1.2 mL, 1.2 mmol, 5.0 equiv) in dry dichloromethane (2 mL), compound **50** (28 mg, 45% yield) was obtained as a white solid after flash column chromatography (CH_2_Cl_2_/CH_3_OH 19:1). Compound **51** was purified as a side product (4 mg, 7% yield). ^1^H NMR (500 MHz, CD_3_OD): δ (ppm) = 7.90 (d, *J* = 8.99 Hz, 1H), 7.03 (d, *J* = 8.50 Hz, 2H), 6.68 (d, *J* = 8.50 Hz, 2H), 6.47 (dd, *J* = 2.50, 8.99 Hz, 1H), 6.41 (d, *J* = 2.50 Hz, 1H), 4.09 (s, 2H), 3.82 (s, 3H). ^13^C NMR (126 MHz, CD_3_OD): δ (ppm) = 204.9, 167.7, 166.8, 147.5, 134.1, 131.0, 125.7, 116.9, 114.2, 108.3, 101.9, 56.1, 45.1. HR-ESI-MS: calculated for C_15_H_16_NO_2_ [*M*+H]^+^ 258.1125, found 258.1123.

##### 2-(4-Aminophenyl)-1-(2,4-dihydroxyphenyl)ethan-1-one (**51**)

To achieve full deprotection in one step, compound **49** (22 mg, 0.06 mmol, 1.0 equiv) was heated to 110 °C in the microwave (5 min, 15 W) with pyridine hydrochloride (1 mL). The reaction was diluted with saturated aqueous Na_2_SO_4_ solution, and this aqueous solution was extracted with ethyl acetate (3 × 5 mL). The combined organic layers were dried over Na_2_SO_4_, filtered, and concentrated *in vacuo*. The residue was coevaporated with toluene (3 × 10 mL) to remove residual pyridine. The product (6 mg, 40% yield) was obtained as a white solid. ^1^H NMR (500 MHz, (CD_3_)_2_SO): δ (ppm) = 12.67 (s, 1H), 10.60 (s, 1H), 7.90 (d, *J* = 8.89 Hz, 1H), 6.92 (m, 2H), 6.49 (m, 2H), 6.36 (dd, *J* = 2.31, 8.89 Hz, 1H), 6.23 (d, *J* = 2.31 Hz, 1H), 4.95 (s, 2H), 4.02 (s, 2H). ^13^C NMR (126 MHz, (CD_3_)_2_SO): δ (ppm) = 203.1, 164.9, 164.8, 147.3, 133.7, 129.7, 121.8, 114.0, 111.9, 108.2, 102.5, 43.4. HR-ESI-MS: calculated for C_14_H_14_NO_3_ [*M*+H]^+^ 244.0968, found 244.0969.

##### (*E*)-2-(4-Aminophenyl)-1-(4-hydroxy-2-methoxyphenyl)ethan-1-one oxime (**4**)

Following procedure **O-C**, using compound **50** (20 mg, 0.07 mmol, 1.0 equiv), potassium acetate (7 mg, 0.21 mmol, 3.0 equiv), and hydroxylamine hydrochloride (7 mg, 0.1 mmol, 1.5 equiv), the oxime (20 mg, 100% yield) was afforded as a white solid and not purified further. ^1^H NMR (500 MHz, (CD_3_)_2_SO): δ (ppm) = 11.96 (s, 1H), 11.44 (s, 1H), 7.39 (d, *J* = 9.43 Hz, 1H), 6.90 (d, *J* = 8.41 Hz, 2H), 6.45 (m, 2H), 6.40 (m, 2H), 4.88 (s, 2H), 3.99 (s, 2H), 3.71 (s, 3H). ^13^C NMR (126 MHz, (CD_3_)_2_SO): δ (ppm) = 160.7, 159.7, 159.4, 146.9, 129.4, 128.9, 123.4, 114.1, 111.3, 105.4, 101.5, 55.1, 28.9. HR-ESI-MS: calculated for C_15_H_17_N_2_O_3_ [*M*+H]^+^ 273.1234, found 273.1232.

##### (*E*)-2-(4-Aminophenyl)-1-(2,4-dihydroxyphenyl)ethan-1-one oxime (**5**)

Following procedure **O-C** using compound **51** (6 mg, 0.03 mmol, 1.0 equiv), potassium acetate (7 mg, 0.07 mmol, 3.0 equiv), and hydroxylamine hydrochloride (3 mg, 0.04 mmol, 3.0 equiv), the oxime (5 mg, 77% yield) was afforded as a white solid after purification by flash column chromatography (CH_2_Cl_2_/CH_3_OH, 3% CH_3_OH). ^1^H NMR (500 MHz, (CD_3_)_2_SO): δ (ppm) = 11.85 (s, 1H), 11.31 (s, 1H), 9.68 (s, 1H), 7.28 (d, *J* = 8.65 Hz, 1H), 6.90 (d, *J* = 8.40 Hz, 2H), 6.44 (m, 2H), 6.23 (dd, *J* = 2.43, 8.65 Hz, 1H), 6.21 (d, *J* = 2.43 Hz, 1H), 4.87 (s, 2H), 3.95 (s, 2H). ^13^C NMR (126 MHz, (CD_3_)_2_SO): δ (ppm) = 159.9, 159.4, 159.2, 146.9, 129.4, 128.9, 123.5, 114.0, 110.0, 106.8, 102.9, 54.9. HR-ESI-MS: calculated for C_14_H_15_N_2_O_3_ [*M*+H]^+^ 259.1077, found 259.1075.

##### 1-(2,4-Dimethoxyphenyl)-2-(4-nitrophenyl)ethan-1-one (**52**)

Following a published procedure, 2-(4-aminophenyl)acetic acid (420 mg, 3.0 mmol) was dissolved with 1,3-dimethoxybenzene (450 mg, 3.0 mmol, 1.0 equiv) in dichloroethane (6 mL).^40^ Polyphosphoric acid (7 g) was added, and the reaction stirred at 85 °C for 3 h. After full conversion, the mixture was cooled to 0 °C and carefully basified with ammonia. The resulting solution was extracted with ethyl acetate (3 × 50 mL—the pH has to be over 7), and the combined organic layers were dried over Na_2_SO_4_, filtered, and concentrated *in vacuo*. Purification by flash column chromatography (CH_2_Cl_2_/ethyl acetate +1% NH_3_, gradient from 0 to 40% ethyl acetate) afforded the product (200 mg, 15%) as a yellow solid. ^1^H NMR (500 MHz, CD_3_OD): δ (ppm) = 7.66 (d, *J* = 8.67 Hz, 1H), 6.92 (d, *J* = 2.83 Hz, 2H), 6.64 (d, *J* = 2.83 Hz, 2H), 6.57 (d, *J* = 2.28 Hz, 1H), 6.54 (dd, *J* = 2.28, 8.67 Hz, 1H), 4.11 (s, 2H), 3.90 (s, 3H), 3.84 (s, 3H). ^13^C NMR (126 MHz, CD_3_OD): δ = 201.6, 166.4, 162.3, 147.0, 133.8, 131.2, 126.5, 121.7, 116.7, 106.8, 99.2, 56.1, 56.0, 50.0. HR-ESI-MS: calculated for C_16_H_18_NO_3_ [*M*+H]^+^ 272.1281, found 272.1281.

##### (*E*)-2-(4-Aminophenyl)-1-(2,4-dimethoxyphenyl)ethan-1-one oxime (**6**)

Following procedure **O-C**, compound **52** (125 mg, 0.5 mmol, 1.0 equiv), potassium acetate (136 mg, 1.5 mmol, 3.0 equiv), and hydroxylamine hydrochloride (48 mg, 0.7 mmol, 1.5 equiv) were dissolved in methanol (10 mL), and the oxime was formed. Purification by preparative HPLC (H_2_O/CH_3_CN + 0.1% formic acid, gradient 5% to 100% CH_3_CN) afforded product **6** (35 mg, 27%) as a white solid. ^1^H NMR (500 MHz, (CD_3_)_2_SO): δ (ppm) = 3.72 (s, 3H), 3.76 (s, 3H), 3.81 (s, 2H), 4.78 (s, 2H), 6.35 (d, *J* = 8.40 Hz, 2H), 6.39 (dd, *J* = 2.35, 8.36 Hz, 1H), 6.51 (d, *J* = 2.30 Hz, 1H), 6.67 (d, *J* = 8.35 Hz, 2H), 6.88 (d, *J* = 8.30 Hz, 1H), 10.90 (s, 1H). ^13^C NMR (126 MHz, (CD_3_)_2_SO): δ (ppm) = 160.7, 158.1, 157.0, 146.5, 131.0, 129.3, 124.1, 118.8, 113.8, 104.5, 98.4, 55.4, 55.2, 32.6. HR-ESI-MS: calculated for C_16_H_19_N_2_O_3_ [*M*+H]^+^ 287.1390, found 287.1384.

##### 2-(4-Chlorophenyl)-1-(6-methoxypyridin-3-yl)ethan-1-one (**53**)

Following procedure **O-A**, using commercially available methyl 6-methoxynicotinate (1.0 g, 5.9 mmol, 1.0 equiv) and 4-chlorophenylacetic acid (1.0 g, 5.9 mmol, 1.0 equiv), the pure product (1.1 g, 72%) was obtained after flash column chromatography (petroleum benzine/ethyl acetate, gradient from 0 to 100% ethyl acetate) as a white solid. ^1^H NMR (500 MHz, CDCl_3_): δ (ppm) = 8.76 (s, 1H), 8.08 (d, *J* = 8.80 Hz, 2H), 7.23 (m, 2H), 7.12 (m, 2H), 6.72 (d, *J* = 8.80 Hz, 1H), 4.12 (s, 2H), 3.93 (s, 3H). ^13^C NMR (126 MHz, CDCl_3_): δ (ppm) = 195.0, 167.0, 149.7, 138.7, 133.2, 132.7, 130.9, 129.1, 126.2, 111.6, 54.3, 44.8. HR-ESI-MS: calculated for C_14_H_13_ClNO_2_ [*M*+H]^+^ 262.0629 (^35^Cl), 264.0600 (^37^Cl), found 262.0609 (100%), 264.0577 (30%).

##### 2-(4-Chlorophenyl)-1-(6-methoxypyridin-3-yl)ethan-1-one oxime (*E*-**7** and *Z*-**7**)

Starting from compound **53** (500 mg, 1.9 mmol, 1.0 equiv) following procedure **O-C**, using potassium acetate (563 mg, 5.8 mmol, 3.0 equiv) and hydroxylamine hydrochloride (200 mg, 2.7 mmol, 1.5 equiv) in methanol (33 mL), the products (*E*-**7**: 370 mg, 70%, *Z*-**7**: 45 mg, 9%) were obtained after flash column chromatography (cyclohexane/ethyl acetate, gradient 0 to 30% ethyl acetate) as white solids. Note: 1D-NOESY experiments were performed on both compounds, irradiating the oxime-hydroxy proton. The isomers could be identified unambiguously. *E*-**7**: ^1^H NMR (500 MHz, CD_3_OD): δ (ppm) = 8.30 (dd, *J* = 0.51, 2.49 Hz, 1H), 7.96 (dd, *J* = 2.49, 8.79 Hz, 1H), 7.23 (s, 4H), 6.74 (dd, *J* = 0.51, 8.79 Hz, 1H), 4.16 (s, 2H), 3.88 (s, 3H). ^13^C NMR (126 MHz, CD_3_OD): δ (ppm) = 165.8, 154.4, 145.9, 138.2, 137.2, 133.1, 131.3, 129.6, 126.7, 111.5, 54.2, 31.2. 1D-NOESY (500 MHz, (CD_3_)_2_SO): δ (ppm) = 11.56 (irradiation point), 8.40, 7.99, 7.31, 7.24, 6.80, 4.14. HR-ESI-MS: calculated for C_14_H_14_ClN_2_O_2_ [*M*+H]^+^ 277.0738 (^35^Cl), 279.0709 (^37^Cl), found 277.0738 (100%), 279.0705 (30%). *Z*-**7**: ^1^H NMR (500 MHz, (CD_3_)_2_SO): δ (ppm) = 11.09 (s, 1H), 8.36 (d, *J* = 2.32 Hz, 1H), 7.90 (dd, *J* = 2.32, 8.71 Hz, 1H), 7.30 (d, *J* = 8.40 Hz, 2H), 7.20 (d, *J* = 8.40 Hz, 2H), 6.78 (d, *J* = 8.71 Hz, 1H), 3.89 (s, 2H), 3.82 (s, 3H). ^13^C NMR (126 MHz, (CD_3_)_2_SO): δ (ppm) = 162.9, 151.0, 147.1, 139.6, 136.7, 131.0, 130.6, 128.4, 122.1, 109.7, 53.2, 40.0. 1D-NOESY (500 MHz, (CD_3_)_2_SO): δ (ppm) = 11.09 (irradiation point), 8.36, 7.90, 7.20, 6.78, 3.89. HR-ESI-MS: calculated for C_14_H_14_ClN_2_O_2_ [*M*+H]^+^ 277.0738 (^35^Cl), 279.0709 (^37^Cl), found 277.0740 (100%), 279.0709 (30%).

##### 2-(4-Chlorophenyl)-1-(6-hydroxypyridin-3-yl)ethan-1-one (**54**)

Compound **53** (0.2 g, 0.38 mmol), LiCl (0.16 g, 1.9 mmol, 5.0 equiv), and *p*-toluenesulfonic acid (0.33 g, 1.9 mmol, 5.0 equiv) were dissolved in dry DMF (15 mL) and heated to 150 °C for 24 h. The reaction was diluted with H_2_O (40 mL), and the mixture was extracted with ethyl acetate (3 × 30 mL). The combined organic layers were washed with saturated aqueous NaCl solution (30 mL), dried over MgSO_4_, filtered, and concentrated *in vacuo*. Purification by flash column chromatography (petroleum benzine/ethyl acetate +1% acetic acid, gradient from 0 or 100% ethyl acetate) afforded the product (54 mg, 29%) as a yellow solid. Note: The deprotection as described in procedure **O-B** does not yield any of the desired product. ^1^H NMR (500 MHz, (CD_3_)_2_SO): δ (ppm) = 12.25 (s, 1H), 8.38 (d, *J* = 2.56 Hz, 1H), 7.88 (dd, *J* = 2.74, 9.70 Hz, 1H), 7.37 (d, *J* = 2.80 Hz, 2H), 7.25 (d, *J* = 8.39 Hz, 2H), 6.38 (d, *J* = 9.67 Hz, 1H), 4.21 (s, 2H). ^13^C NMR (126 MHz, (CD_3_)_2_SO): δ (ppm) = 193.1, 162.5, 141.6, 138.5, 134.4, 131.7, 131.3, 128.2, 119.8, 116.0, 42.7. HR-ESI-MS: calculated for C_13_H_11_ClNO_2_ [*M*+H]^+^ 248.0473 (^35^Cl), 250.0443 (^37^Cl), found 248.0472 (100%), 250.0440 (30%).

##### 2-(4-Chlorophenyl)-1-(6-hydroxypyridin-3-yl)ethan-1-one oxime (*E*-**8** and *Z*-**8**)

Starting from compound **54** (40 mg, 0.2 mmol, 1.0 equiv) following procedure **O-C**, using potassium acetate (48 mg, 0.5 mmol, 3.0 equiv) and hydroxylamine hydrochloride (16 mg, 0.2 mmol, 1.5 equiv), the products (*E*-**8**: 10 mg, 24%, *Z*-**8**: 3 mg, 8%) were afforded after preparative HPLC (H_2_O/CH_3_CN + 1% formic acid, gradient from 5% to 100% CH_3_CN) as white solids. **8***E*: ^1^H NMR (500 MHz, (CD_3_)_2_SO): δ (ppm) = 11.71 (s, 1H), 11.36 (s, 1H), 7.83 (dd, *J* = 2.55, 9.75 Hz, 1H), 7.53 (d, *J* = 2.55 Hz, 1H), 7.33 (d, *J* = 8.42 Hz, 2H), 7.22 (d, *J* = 8.42 Hz, 2H), 6.33 (d, *J* = 9.75 Hz, 1H), 4.01 (s, 2H). ^13^C NMR (126 MHz, (CD_3_)_2_SO): δ (ppm) = 162.1, 151.4, 137.9, 136.3, 133.6, 130.8, 130.2, 128.5, 120.4, 113.8, 28.7. HR-ESI-MS: calculated for C_13_H_12_ClN_2_O_2_ [*M*+H]^+^ 263.0582 (^35^Cl), 265.0552 (^37^Cl), found 263.0581 (100%), 265.0549 (30%). **8***Z*: ^1^H NMR (500 MHz, (CD_3_)_2_SO): δ (ppm) = 11.78 (s, 1H), 11.17 (s, 1H), 7.93 (d, *J* = 2.52 Hz, 1H), 7.70 (dd, *J* = 2.52, 9.68 Hz, 1H), 7.33 (d, *J* = 8.42 Hz, 2H), 7.21 (d, *J* = 8.42 Hz, 2H), 6.25 (d, *J* = 9.68 Hz, 1H), 3.81 (s, 2H). ^13^C NMR (126 MHz, (CD_3_)_2_SO): δ (ppm) = 161.7, 149.7, 141.8, 138.1, 137.5, 131.4, 130.9, 128.9, 119.3, 110.8, 38.8. HR-ESI-MS: calculated for C_13_H_12_ClN_2_O_2_ [*M*+H]^+^ 263.0582 (^35^Cl), 265.0552 (^37^Cl), found 263.0581 (100%), 265.0549 (30%).

Note: From the spectra, it is not entirely clear whether the 2-hydroxy pyridine or the 2-pyridone is the correct tautomer. A pyridone carbon should have a chemical shift above 160 ppm. As only one carbon peak at 160 ppm or higher is visible (the same can be seen for compounds *E*-**7** and *Z*-**7** that are 2-methoxy pyridines) that belongs to the oxime-carbon (−C=NH), we assume that the 2-hydroxy pyridine is the dominant form in (CD_3_)_2_SO. A comparison to D_2_O was not possible due to limited solubility.

##### (*E*)-2-(4-Chlorophenyl)-1-(2,4-dihydroxyphenyl)ethan-1-one *o*-Methyl Oxime (**11**)

2-(4-Chlorophenyl)-1-(2,4-dihydroxyphenyl)ethan-1-one (100 mg, 0.38 mmol, 1.0 equiv) and *o*-methylhydroxylamine•HCl (64 mg, 0.76 mmol, 2.0 equiv) were dissolved in methanol/pyridine (10:1, 4 mL) and added to a flask under a N_2_ atmosphere in solution.^12^ Sodium sulfate (135 mg, 0.95 mmol, 2.5 equiv) was added, and the reaction mixture was heated to 95 °C under reflux. The reaction mixture was refluxed for 18 h and then allowed to cool to room temperature. H_2_O (15 mL) was added followed by ethyl acetate (15 mL). The layers were mixed and separated, and the aqueous layer was extracted with ethyl acetate (2×, 10 mL). The combined organic layers were washed with saturated aqueous NaCl solution, dried over Na_2_SO_4_, filtered, and concentrated to give a pale yellow sticky solid, which was purified by flash column chromatography (heptane:ethyl acetate, gradient from 0 to 100% ethyl acetate) to give the product (80 mg, 72%) as a white solid. ^1^H NMR (500 MHz, CD_3_OD): δ = 7.25 (m, 5H), 6.31 (d, *J* = 2.40 Hz, 1H), 6.28 (q, *J* = 3.71 Hz, 1H), 4.17 (s, 2H), 3.98 (s, 3H). ^13^C NMR (126 MHz, CD_3_OD): δ = 161.3, 161.1, 161.0, 136.8, 133.2, 131.0, 130.7, 129.6, 110.9, 108.3, 104.3, 62.7, 31.3. HR-ESI-MS: calculated for C_15_H_15_ClNO_3_ [*M*+H]^+^ 292.0735 (^35^Cl), 294.0705 (^37^Cl), found 292.0706 (100%), 294.0674 (30%).

##### 4-(2-(4-Chlorophenyl)-1-hydroxyethyl)benzene-1,3-diol (**13**)

2-(4-Chlorophenyl)-1-(2,4-dihydroxyphenyl)ethan-1-one (100 mg, 0.38 mmol, 1.0 equiv) was dissolved in methanol and added to a solution of sodium borohydride (22 mg, 0.58 mmol, 1.5 equiv) in methanol (5 mL).^12^ NaBH_4_ was added two more times (16 mg, 0.42 mmol, 1.1 equiv. and 40 mg, 1.1 mmol, 2.8 equiv), and the reaction was stirred for 1 h after each addition. After full conversion, the solvent was removed under reduced pressure and the solids were dissolved in a mixture of dichloromethane (20 mL), saturated aqueous NaCl solution (5 mL), and water (5 mL). The mixture was acidified to pH 5, and the organic layer was separated. The aqueous layer was further extracted with dichloromethane (2 × 35 mL), dried over Na_2_SO_4_, filtered, and concentrated. The product was purified by reverse-phased flash column chromatography (H_2_O/CH_3_OH gradient from 5 to 100% CH_3_OH) to give the product as a white solid (21 mg, 21%). ^1^H NMR (500 MHz, CD_3_OD): δ (ppm) = 7.20 (d, *J* = 2.81 Hz, 2H), 7.12 (d, *J* = 2.81 Hz, 2H), 6.94 (d, *J* = 7.99 Hz, 1H), 6.23 (m, 1H), 5.04 (d, *J* = 5.36, 7.70 Hz, 1H), 2.95 (ddd, *J* = 5.36, 7.70, 13.56, 54.73 Hz, 2H). ^13^C NMR (126 MHz, CD_3_OD): δ (ppm) = 158.5, 156.6, 139.4, 132.7, 132.2, 128.9, 128.5, 122.5, 107.3, 103.4, 72.0, 44.6. HR-ESI-MS: calculated for C_14_H_12_ClO_3_ [*M*+H]^−^ 263.0480 (^35^Cl), 265.0451 (^37^Cl), found 263.0457 (100%), 265.0426 (30%).

#### Synthesis and Characterization of Indoles

##### Aldehyde Formation (Procedure **I-A**)

When the 1*H*-indole-3-carbaldehydes were not commercially available, they were synthesized following a literature procedure.^41^ POCl_3_ (1.3 equiv) was stirred in DMF for 15 min. A solution of 1*H*-indole (1.0 equiv) in DMF was added, and the reaction mixture was stirred at 80 °C for 15 min. Then, aqueous NaOH solution (2 M) was added and stirred at 110 °C for 45 min. The reaction mixture was diluted with *tert*-butyl methyl ether (TBME) and H_2_O. The organic layer was separated, and the aqueous layer was extracted with TBME (2×). The combined organic layers were dried over Na_2_SO_4_, filtered, and concentrated *in vacuo* to obtain the crude product that was used without further purification unless stated otherwise.

##### S_N_2 Substitution (Procedure **I-B**)

The 1-substituted-1*H*-indole-3-carbaldehyde products were synthesized by following a previously reported procedure.^42^ Sodium hydride (1.8 equiv) was suspended in DMF, and the suspension was cooled to 0 °C under a nitrogen atmosphere. 1*H*-Indole-3-carbaldehyde (1.0 equiv) was added. The mixture was stirred at 25 °C for 30 min, after which benzyl bromide (1.2 equiv) was added. The resulting mixture was stirred for 16 h. Water and ethyl acetate were added, and the layers were separated. The aqueous layer was extracted with ethyl acetate (2×). The combined organic layers were washed with water (3×) and saturated aqueous NaCl solution, dried over Na_2_SO_4_, filtered, and concentrated *in vacuo*. The crude product was directly used without further purification for the next reaction unless stated otherwise.

##### Reduction (Procedure **I-C**)

The (1-substituted-1*H*-indol-3-yl)methanol products were synthesized by following a previously reported procedure.^43^ To a solution of 1-substituted-1*H*-indole-3-carbaldehyde (1.0 equiv) in methanol, sodium borohydride (3.2 equiv) was added portion-wise, and the reaction mixture was stirred at 25 °C for 1 h. Water was added with care to the reaction, and the mixture was extracted with dichloromethane (2×). The combined organic layers were dried over Na_2_SO_4_, filtered, and concentrated *in vacuo*.

##### (1-(3,4-Dichlorobenzyl)-1*H*-pyrrolo[2,3-*b*]pyridin-3-yl)methanol (**14**)

1*H*-Pyrrolo[2,3-*b*]pyridine-3-carbaldehyde (500 mg, 3.4 mmol, 1.0 equiv) and 3,4-dichlorobenzyl-bromide (1.2 g, 4.9 mmol, 1.2 equiv) were added as described in procedure **I-B** to a solution of NaH (150 mg, 60% dispersion in mineral oil, 3.8 mmol, 1.1 equiv) in DMF (14 mL), and the alkylation product (600 mg, 58%) was purified by flash column chromatography (CH_2_Cl/CH_3_OH 99.5:0.5). Following procedure **I-C**, the alkylation product (600 mg, 1.97 mmol, 1.0 equiv) was reduced to the alcohol with NaBH_4_ (240 mg, 6.3 mmol, 3.2 equiv) in methanol (250 mL), and pure product **14** (507 mg, 84% yield) was obtained without further purification as a white solid. ^1^H NMR (500 MHz, CDCl_3_): δ (ppm) = 8.35 (dd, *J* = 1.54, 4.77 Hz, 1H), 8.06 (dd, *J* = 1.54, 7.89 Hz, 1H), 7.36 (d, *J* = 8.27 Hz, 1H), 7.31 (d, *J* = 2.05 Hz, 1H), 7.16 (s, 1H), 7.13 (dd, *J* = 4.77, 7.89 Hz, 1H), 7.05 (dd, *J* = 2.05, 8.27 Hz, 1H), 5.41 (s, 2H), 4.85 (s, 2H). ^13^C NMR (126 MHz, CDCl_3_): δ (ppm) = 147.9, 143.7, 138.0, 132.9, 131.9, 130.8, 129.5, 128.1, 126.9, 125.9, 119.5, 116.3, 114.7, 57.3, 46.8. HR-ESI-MS: calculated for C_15_H_13_Cl_2_N_2_O [*M*+H]^+^ 307.0399 (^35^Cl), 309.0370 (^37^Cl), found 307.0394 (100%), 309.0361 (60%).

##### (1-(3,4-Dichlorophenyl)-1*H*-indol-3-yl)methanol (**15**)

In contrast to procedure **I-B**, the suspension of indole-3-carbaldehyde (500 mg, 3.5 mmol, 1.0 equiv) in DMF (10 mL), 3,4-dichlorofluorobenzene (0.5 mL, 4.2 mmol, 1.2 equiv), and NaH (350 mg, 8.6 mmol, 2.4 equiv) was stirred for 24 h at 190 °C.^44^ After full conversion, the reaction mixture was diluted with TBME (10 mL) and washed with saturated aqueous NaCl solution (3 × 10 mL). The organic layer was dried over Na_2_SO_4_, filtered, and concentrated *in vacuo* to obtain product **55** (960 mg, 3.3 mmol) that was used without further purification. Compound **15** was synthesized following procedure **I-C**, using **55** (960 mg, 3.3 mmol, 1.0 equiv) and NaBH_4_ (430 mg, 11.34 mmol, 3.4 equiv) in methanol (200 mL). The reaction mixture was concentrated, and the residue was dissolved in TBME (50 mL) and washed with saturated aqueous NaCl solution (100 mL) and water (100 mL). The organic layer was dried over Na_2_SO_4_, filtered, and concentrated *in vacuo*. Purification by reversed-phase column chromatography (H_2_O/CH_3_CN, gradient 20 to 100% CH_3_CN) afforded **15** (20% over two steps) as a yellow sticky solid. ^1^H NMR (500 MHz, (CD_3_)_2_SO): δ (ppm) = 7.72 (d, *J* = 7.74 Hz, 1H), 7.58 (m, 4H), 7.22 (t, *J* = 6.91 Hz, 1H), 7.15 (t, *J* = 6.91 Hz, 1H), 4.98 (t, *J* = 5.33 Hz, 1H), 4.70 (d, *J* = 5.33 Hz, 2H). ^13^C NMR (126 MHz, (CD_3_)_2_SO): δ (ppm) = 138.0, 135.4, 131.4, 129.8, 129.7, 128.2, 126.1, 125.1, 125.1, 122.8, 120.2, 119.9, 118.8, 110.3, 55.2. HR-ESI-MS: calculated for C_15_H_10_Cl_2_Ṅ [*M*–OH]˙ 274.0190 (^35^Cl), 276.0161 (^37^Cl), found 274.0184 (100%), 276.0153 (70%).

##### (1-(3,4-Dichlorobenzyl)-1*H*-indazol-3-yl)methanol (**18**)

Following procedure **I-B**, 3-iodo-1*H*-indazole (1.5 g, 6.1 mmol, 1.0 equiv) was dissolved in a suspension of NaH (270 mg, 6.7 mmol, 1.1 equiv) in DMF (40 mL). 3,4-Dichlorobenzyl bromide (1 mL, 7.36 mmol, 1.2 equiv) was added. The crude material was purified by column chromatography (heptane/ethyl acetate 97:3) affording 1-(3,4-dichlorobenzyl)-3-iodo-1*H*-indazole (**56**) as a white solid (2.1 g, 85% yield). A round-bottomed flask was charged with *i*-PrMgCl (2 M in THF, 3.0 mL, 6.0 mmol, 1.5 equiv) and dry THF (5 mL) and was cooled to 0 °C.^45^ A solution of compound **56** (1.6 g, 4.0 mmol) in dry THF (20 mL) was added dropwise, and the resulting mixture was stirred at 0 °C for 1 h. DMF (1.2 mL, 16.0 mmol, 4.0 equiv) was added, and the mixture was stirred for 5 h. After full conversion, aqueous HCl solution (1 M, 10 mL) and toluene (10 mL) were added to the reaction mixture. The layers were separated, and the organic layer was washed with saturated aqueous NaHCO_3_ solution (5 mL) and concentrated *in vacuo*. The crude material was purified using column chromatography (heptane/ethyl acetate 95:5) affording 1-(3,4-dichlorobenzyl)-1*H*-indazole-3-carbaldehyde (**57**) as a white solid (73 mg, 6% yield). Indole **18** was synthesized following procedure **I-C**, using **57** (70 mg, 0.23 mmol, 1.0 equiv) and NaBH_4_ (9 mg, 0.23 mmol, 1.0 equiv) in ethanol (10 mL). The crude product was purified using flash column chromatography (heptane/ethyl acetate 8:2) to provide **18** as a white solid (35 mg, 50%). ^1^H NMR (500 MHz, (CD_3_)_2_SO): δ (ppm) = 7.86 (d, *J* = 8.09 Hz, 1H), 7.70 (d, *J* = 8.49 Hz, 1H), 7.58 (d, *J* = 8.30 Hz, 1H), 7.52 (d, *J* = 1.96 Hz, 1H), 7.39 (m, 1H), 7.15 (m, 2H), 5.62 (s, 2H), 5.32 (t, *J* = 5.81 Hz, 1H), 4.77 (d, *J* = 5.81 Hz, 2H). ^13^C NMR (126 MHz, (CD_3_)_2_SO): δ (ppm) = 145.8, 140.4, 138.9, 131.1, 130.9, 130.2, 129.4, 127.8, 126.6, 122.3, 121.1, 120.3, 109.6, 56.6, 50.2. HR-ESI-MS: calculated for C_15_H_13_Cl_2_N_2_O [*M*+H]^+^ 307.0399 (^35^Cl), 309.0370 (^37^Cl), found 307.0400 (100%), 309.0368 (70%), 289.0293 (20%).

##### 2-(1-(3,4-Dichlorobenzyl)-1*H*-indol-3-yl)ethan-1-ol (**19**)

Tryptophol (0.59 g, 3.7 mmol), *tert*-butyldimethylsilyl (TBDMS) chloride (0.86 g, 5.7 mmol, 1.5 equiv), and imidazole (0.38 g, 5.6 mmol, 1.5 equiv) were dissolved in CH_2_Cl_2_ (50 mL).^46^ The reaction mixture was stirred at 25 °C for 24 h. Then, it was diluted with CH_2_Cl_2_ (50 mL), washed with saturated aqueous NaCl solution (100 mL), and water (100 mL). The organic layer was dried over Na_2_SO_4_, filtered, and concentrated *in vacuo* to obtain crude 3-(2-((*tert*-butyldimethylsilyl)oxy)ethyl)-1*H*-indole (**58**) (1.03 g, 3.75 mmol) as a thick red oil, which was used without further purification in the next step. Compound **58** (1.03 g, 3.75 mmol, 1.0 equiv) and 3,4-dichlorobenzyl bromide (1.08 g, 4.5 mmol, 1.2 equiv) were mixed as described in procedure **I-B** in DMF (10 mL) with NaH (355 mg, 8.9 mmol, 2.4 equiv). After workup, crude 3-(2-((*tert*-butyldimethylsilyl)oxy)ethyl)-1-(3,4-dichlorobenzyl)-1*H*-indole (**59**) was obtained as a yellow-green oil. Purification by normal phase column chromatography (heptane/EtOAc, gradient 0% to 10% EtOAc) afforded compound **59** (0.86 g, 205 mmol). To a solution of compound **59** (0.86 g, 2.05 mmol, 1.0 equiv) in THF (50 mL), *tetra*-butylammonium fluoride (TBAF) in THF (1 M, 4.1 mL, 4.1 mmol, 2.0 equiv) was added. The reaction mixture was stirred at 90 °C for 24 h. The reaction mixture was concentrated and purified by reverse-phase column chromatography (H_2_O/CH_3_CN + 0.1% formic acid, gradient 20% to 100% CH_3_CN), affording compound **19** (0.32 g, 0.99 mmol, 26% over three steps) as a yellowish sticky oil. ^1^H NMR (500 MHz, (CD_3_)_2_SO): δ (ppm) = 7.56 (d, *J* = 8.31 Hz, 1H), 7.54 (d, *J* = 7.89 Hz, 1H), 7.48 (d, *J* = 1.96 Hz, 1H), 7.40 (d, *J* = 8.19 Hz, 1H), 7.32 (s, 1H), 7.10 (m, 2H), 7.01 (m, 1H), 5.37 (s, 2H), 4.67 (t, *J* = 5.32 Hz, 1H), 3.65 (m, 2H), 2.84 (t, *J* = 7.21 Hz, 2H). ^13^C NMR (126 MHz, (CD_3_)_2_SO): δ (ppm) = 139.8, 135.8, 131.1, 130.8, 129.9, 129.1, 128.1, 127.4, 126.6, 121.4, 118.9, 118.7, 112.1, 109.9, 61.6, 47.6, 28.7. HR-ESI-MS: calculated for C_17_H_16_Cl_2_NO [*M*+H]^+^ 320.0603 (^35^Cl), 322.0574 (^37^Cl), found 320.0605 (100%), 322.0573 (70%).

##### (1-(3,4-Dichlorobenzyl)-5-methoxy-1*H*-indol-3-yl)methanol (**21**)

5-Methoxy-1*H*-indole-3-carbaldehyde (500 mg, 2.9 mmol, 1.0 equiv) and 3,4-dichlorobenzyl bromide (823 mg, 3.4 mmol, 1.2 equiv) were mixed as described in procedure **I-B** in a suspension of NaH (125 mg, 60% dispersion in mineral oil, 3.1 mmol, 1.1 equiv) in DMF (14 mL). The alkylation product (950 mg, 99%) was used without further purification. Following procedure **I-C**, the aldehyde (358 mg, 1.07 mmol, 1.0 equiv) was reduced to the alcohol with NaBH_4_ (130 mg, 3.42 mmol, 3.2 equiv) in methanol (100 mL) and the pure product **21** (300 mg, 83%) was obtained without further purification as a yellowish solid. ^1^H NMR (500 MHz, (CD_3_)_2_SO): δ (ppm) = 7.56 (d, *J* = 8.30 Hz, 1H), 7.47 (d, *J* = 1.95 Hz, 1H), 7.38 (s, 1H), 7.31 (d, *J* = 8.90 Hz, 1H), 7.12 (m, 2H), 6.75 (dd, *J* = 2.50, 8.90 Hz, 1H), 5.34 (s, 2H), 4.83 (t, *J* = 5.45 Hz, 1H), 4.61 (d, *J* = 5.45 Hz, 2H), 3.75 (s, 3H). ^13^C NMR (126 MHz, (CD_3_)_2_SO): δ (ppm) = 153.4, 139.8, 131.3, 131.1, 130.7, 129.9, 129.0, 127.7, 127.5, 127.4, 115.9, 111.6, 110.7, 101.2, 55.3, 55.3, 47.8. HR-ESI-MS: calculated for C_17_H_14_Cl_2_NȮ [*M*–OH]˙ 318.0452 (^35^Cl), 320.0423 (^37^Cl), found 318.0415 (100%), 320.0383 (60%).

##### (1-(3-Chlorobenzyl)-4-methoxy-1*H*-indol-3-yl)methanol (**22**)

4-Methoxy-1*H*-indole-3-carbaldehyde (200 mg, 1.14 mmol, 1.0 equiv) was synthesized following procedure **I-A**, using 4-methoxy-1*H*-indole (1.0 g, 6.8 mmol, 1.0 equiv) as the starting material with POCl_3_ (1.3 g, 8.2 mmol, 1.2 equiv) and DMF (2.5 g, 34 mmol, 5.0 equiv) in NaOH (2 M, 40 mL). 3-Chlorobenzyl bromide (282 mg, 1.37 mmol, 1.1 equiv) was added as described in procedure **I-B** with NaH (82 mg, 60% in mineral oil, 2.05 mmol, 1.8 equiv) in DMF (10 mL). Product **22** was synthesized by reduction of 1-(3-chlorobenzyl)-4-methoxy-1*H*-indole-3-carbaldehyde (380 mg, 1.27 mmol, 1.0 equiv) following procedure **I-C**, using NaBH_4_ (154 mg, 4.06 mmol, 3.2 equiv). The product (195 mg, 57% yield over three steps) was obtained after flash column chromatography (petroleum benzine/ethyl acetate, gradient from 0 to 100% ethyl acetate) as a sticky yellow solid. ^1^H NMR (500 MHz, (CD_3_)_2_SO): δ (ppm) = 7.32 (m, 2H), 7.24 (m, 1H), 7.22 (m, 1H), 7.12 (m, 1H), 7.00 (d, *J* = 0.99 Hz, 1H), 6.99 (s, 1H), 6.49 (m, 1H), 5.35 (s, 2H), 4.75 (d, *J* = 2.87 Hz, 2H), 4.65 (t, *J* = 5.54 Hz, 1H), 3.82 (s, 3H). ^13^C NMR (126 MHz, (CD_3_)_2_SO): δ (ppm) = 154.1, 141.1, 137.7, 133.1, 130.5, 127.3, 126.8, 125.8, 125.2, 122.4, 116.7, 116.3, 103.4, 99.5, 56.9, 55.2, 48.4. HR-ESI-MS: calculated for C_17_H_15_ClNȮ [*M*–OH]˙ 284.0842 (^35^Cl), 286.0813 (^37^Cl), found 284.0831 (100%), 286.0800 (70%).

##### (7-Chloro-1-(3,4-dichlorobenzyl)-1*H*-indol-3-yl)methanol (**23**)

7-Chloro-1*H*-indole-3-carbaldehyde (500 mg, 2.8 mmol, 1.0 equiv) and 3,4-dichlorobenzyl bromide (804 mg, 3.4 mmol, 1.2 equiv) were added as described in procedure **I-B** to a suspension of NaH (123 mg, 60% dispersion in mineral oil, 3.1 mmol, 1.1 equiv) in DMF (14 mL). The alkylation product (577 mg, 61%) was purified by flash column chromatography (dichloromethane/heptane 3:1). Following procedure **I-C**, the aldehyde (387 mg, 1.14 mmol, 1.0 equiv) was reduced to the alcohol with NaBH_4_ (140 mg, 3.68 mmol, 3.2 equiv) in methanol (100 mL), and pure product **23** (360 mg, 93%) was obtained without purification as a white solid. ^1^H NMR (500 MHz, (CD_3_)_2_SO): δ (ppm) = 7.62 (dd, *J* = 0.91, 7.85 Hz, 1H), 7.56 (d, *J* = 8.34 Hz, 1H), 7.49 (s, 1H), 7.29 (d, *J* = 2.01 Hz, 1H), 7.13 (dd, *J* = 0.91, 7.50 Hz, 1H), 7.03 (t, *J* = 7.72 Hz, 1H), 6.90 (dd, *J* = 2.01, 8.34 Hz, 1H), 5.73 (s, 2H), 4.98 (t, *J* = 5.38 Hz, 1H), 4.65 (d, *J* = 5.38 Hz, 2H). ^13^C NMR (126 MHz, (CD_3_)_2_SO): δ (ppm) = 141.1, 131.2, 130.9, 130.9, 130.6, 130.2, 129.7, 127.9, 126.2, 123.2, 120.3, 118.8, 117.1, 115.4, 55.0, 49.7. HR-ESI-MS: calculated for C_16_H_11_Cl_3_Ṅ [*M*–OH]˙ 321.9957(^35^Cl), 323.9928 (^37^Cl), found 321.9953 (100%), 323.9921 (95%), 325.9889 (30%).

##### (1-(3-Chlorobenzyl)-4-fluoro-1*H*-indol-3-yl)methanol (**24**)

4-Fluoro-1*H*-indole-3-carbaldehyde (200 mg, 1.23 mmol, 1.0 equiv) was synthesized following procedure **I-A** using 4-fluoro-1*H*-indole (1.0 g, 7.4 mmol, 1.0 equiv) as starting material with POCl_3_ (1.4 g, 8.9 mmol, 1.2 equiv) and DMF (2.7 g, 37 mmol, 5.0 equiv) in NaOH (2 M, 40 mL). From there, 3-chlorobenzyl bromide (302 mg, 1.47 mmol, 1.2 equiv) was added as described in procedure **I-B** with NaH (88 mg, 60% mineral oil, 2.21 mmol, 1.8 equiv) in DMF (10 mL). The final indole **24** was synthesized by reduction of 1-benzyl-4-fluoro-1*H*-indole-3-carbaldehyde (420 mg, 1.46 mmol, 1.0 equiv) following procedure **I-C**, using NaBH_4_ (175 mg, 4.67 mmol, 3.2 equiv) in methanol (10 mL). The product **24** (144 mg, 41% over three steps) was obtained after flash column chromatography (petroleum benzine/ethyl acetate, gradient from 0 to 100% ethyl acetate) as a sticky orange oil. ^1^H NMR (500 MHz, (CD_3_)_2_SO): δ (ppm) = 7.45 (s, 1H), 7.33 (m, 2H), 7.29 (m, 2H), 7.16 (m, 1H), 7.07 (td, *J* = 5.13, 7.79 Hz, 1H), 6.77 (dd, *J* = 7.82, 11.17 Hz, 1H), 5.41 (s, 2H), 4.93 (t, *J* = 5.25 Hz, 1H), 4.69 (d, *J* = 5.25 Hz, 2H). ^13^C NMR (126 MHz, (CD_3_)_2_SO): δ (ppm) = 156.4 (d, *J* = 245.28 Hz), 140.6, 139.0 (d, *J* = 12.18 Hz), 133.2, 130.6, 127.6, 127.5, 127.0, 125.9, 122.1 (d, *J* = 7.74 Hz), 115.2 (d, *J* = 21.04 Hz), 114.7 (d, *J* = 3.39 Hz), 106.7 (d, *J* = 3.00 Hz), 104.2 (d, *J* = 19.07 Hz), 56.0, 48.5. ^19^F-NMR (470 MHz, (CD_3_)_2_SO): δ (ppm) = −122.62 (q, *J* = 5.27 Hz). HR-ESI-MS: calculated for C_16_H_12_ClFṄ [*M*–OH]˙ 272.0642 (^35^Cl), 274.0613 (^37^Cl), found 272.0630 (100%), 274.0600 (30%).

##### (1-Benzyl-4-fluoro-1*H*-indol-3-yl)methanol (**25**)

4-Fluoro-1*H*-indole-3-carbaldehyde (200 mg, 1.23 mmol, 1.0 equiv) was synthesized following procedure **I-A**, using 4-fluoro-1*H*-indole (1.0 g, 7.4 mmol, 1.0 equiv) as starting material with POCl_3_ (1.4 g, 8.9 mmol, 1.2 equiv) and DMF (2.7 g, 37 mmol, 5.0 equiv) in NaOH (2 M, 40 mL). From there, benzyl bromide (252 mg, 1.5 mmol, 1.2 equiv) was added as described in procedure **I-B**, using NaH (88 mg, 2.21 mmol, 1.8 equiv) in DMF (10 mL). The final indole **25** was synthesized by reduction of the aldehyde (330 mg, 1.3 mmol, 1.0 equiv) following procedure **I-C** using NaBH_4_ (158 mg, 4.16 mmol, 3.2 equiv) in methanol (10 mL). The product (203 mg, 67% over three steps) was obtained after flash column chromatography (petroleum benzine/ethyl acetate, gradient 0 to 100% ethyl acetate) as a sticky orange oil. ^1^H NMR (500 MHz, (CD_3_)_2_SO): δ (ppm) = 7.42 (s, 1H), 7.28 (m, 4H), 7.21 (d, *J* = 7.12 Hz, 2H), 7.05 (td, *J* = 5.33, 8.06 Hz, 1H), 6.75 (dd, *J* = 7.82, 11.32 Hz, 1H), 5.39 (s, 2H), 4.90 (t, *J* = 5.21 Hz, 1H), 4.69 (d, *J* = 5.21 Hz, 2H). ^13^C NMR (126 MHz, (CD_3_)_2_SO): δ (ppm) = 156.4 (d, *J* = 245.10 Hz), 139.0 (d, *J* = 12.12 Hz), 138.0, 128.6, 127.7, 127.5, 127.2, 121.9 (d, *J* = 7.76 Hz), 115.2 (d, *J* = 21.04 Hz), 114.4 (d, *J* = 3.37 Hz), 106.8 (d, *J* = 3.12 Hz), 104.0 (d, *J* = 19.11 Hz), 56.0 (d, *J* = 1.18 Hz), 49.2. ^19^F-NMR (470 MHz, (CD_3_)_2_SO): δ (ppm) = −122.76 (q, *J* = 5.42 Hz). HR-ESI-MS: calculated for C_16_H_13_FṄ [*M*–OH]˙ 238.1032, found 238.1021.

##### 1-(3,4-Dichlorobenzyl)-3-methyl-1*H*-indole (**26**)

3-Methyl-1*H*-indole (498 mg, 3.8 mmol, 1.0 equiv) and 3,4-dichlorobenzyl bromide (1.0 g, 4.2 mmol, 1.1 equiv) were added as described in procedure **I-B** to a suspension of NaH (339 mg, 8.5 mmol, 2.2 equiv) in DMF (10 mL), and pure product **26** (210 mg, 19%) was obtained after purification by flash column chromatography (heptane/ethyl acetate, gradient 0 to 100% ethyl acetate) followed by reversed-phase flash column chromatography (H_2_O/CH_3_CN, gradient 20% to 100% CH_3_CN) as an off-white solid. ^1^H NMR (500 MHz, (CD_3_)_2_SO): δ (ppm) = 7.55 (d, *J* = 8.30 Hz, 1H), 7.50 (d, *J* = 7.82 Hz, 1H), 7.47 (d, *J* = 1.99 Hz, 1H), 7.41 (d, *J* = 8.20 Hz, 1H), 7.27 (d, *J* = 0.87 Hz, 1H), 7.10 (m, 2H), 7.01 (td, *J* = 0.87, 7.46 Hz, 1H), 5.36 (s, 2H), 2.26 (d, *J* = 0.94 Hz, 3H). ^13^C NMR (126 MHz, (CD_3_)_2_SO): δ (ppm) = 139.8, 135.9, 131.1, 130.8, 129.9, 129.0, 128.6, 127.4, 126.5, 121.4, 118.8, 118.7, 109.8, 109.8, 47.6, 9.5. HR-ESI-MS: calculated for C_16_H_14_Cl_2_N [*M*+H]^+^ 290.0498 (^35^Cl), 292.0468 (^37^Cl), found 290.0497 (100%), 292.0466 (70%).

##### (1-(3,4-Dichlorobenzyl)-6-fluoro-1*H*-indol-3-yl)methanol (**27**)

6-Fluoro-1*H*-indole-3-carbaldehyde (500 mg, 3.1 mmol, 1.0 equiv) and 3,4-dichlorobenzyl bromide (883 mg, 3.7 mmol, 1.2 equiv) were added as described in procedure **I-B** in a suspension of NaH (135 mg, 60% dispersion in mineral oil, 3.4 mmol, 1.1 equiv) in DMF (14 mL). The alkylation product (1.0 g, 98%) was used without further purification. Following procedure **I-C**, the aldehyde (1.0 g, 3.0 mmol, 1.0 equiv) was reduced to the alcohol with NaBH_4_ (385 mg, 10.2 mmol, 3.4 equiv) in methanol (130 mL), and product **27** (473 mg, 48% yield over two steps) was obtained after purification by flash column chromatography (dichloromethane) as a white solid. ^1^H NMR (500 MHz, (CD_3_)_2_SO): δ (ppm) = 7.59 (m, 2H), 7.55 (d, *J* = 1.95 Hz, 1H), 7.43 (s, 1H), 7.38 (dd, *J* = 2.29, 10.49 Hz, 1H), 7.18 (dd, *J* = 1.95, 8.33 Hz, 1H), 6.89 (m, 1H), 5.36 (s, 2H), 4.90 (t, *J* = 5.40 Hz, 1H), 4.61 (d, *J* = 5.40 Hz, 2H). ^13^C NMR (126 MHz, (CD_3_)_2_SO): δ (ppm) = 159.2 (d, *J* = 235.08 Hz), 139.3, 136.2 (d, *J* = 12.27 Hz), 131.1, 130.9, 130.1, 129.3, 127.6, 127.3 (d, *J* = 3.45 Hz), 123.9, 120.6 (d, *J* = 10.28 Hz), 116.7, 107.4 (d, *J* = 24.49 Hz), 96.5 (d, *J* = 26.48 Hz), 55.2, 47.7. ^19^F-NMR (470 MHz, (CD_3_)_2_SO): δ (ppm) = −120.84 (m). HR-ESI-MS: calculated for C_16_H_11_Cl_2_FṄ [*M*–OH]˙ 306.0253 (^35^Cl), 308.0223 (^37^Cl), found 306.0248 (100%), 308.0215 (70%).

##### 1-(3,4-Dichlorobenzyl)-1*H*-indole (**28**)

Indole (509 mg, 4.35 mmol, 1.0 equiv) and 3,4-dichlorobenzyl bromide (0.87 mL, 5.12 mmol, 1.2 equiv) were added as described in procedure **I-B** to a suspension of NaH (381 mg, 9.5 mmol, 2.2 equiv) in DMF (20 mL). The obtained crude material was purified by flash column chromatography (heptane/ethyl acetate, gradient 0 to 20% ethyl acetate) followed by reversed-phase column chromatography (H_2_O:CH_3_CN + 0.1% formic acid, gradient 20% to 100% CH_3_CN), affording compound **28** (63 mg, 5% yield) as a pale-yellow sticky oil. ^1^H NMR (500 MHz, (CD_3_)_2_SO): δ (ppm) = 7.57 (d, *J* = 8.26 Hz, 1H), 7.56 (d, *J* = 7.83 Hz, 1H), 7.54 (d, *J* = 3.16 Hz, 1H), 7.48 (dd, *J* = 0.68, 8.26 Hz, 1H), 7.45 (q, *J* = 2.98 Hz, 1H), 7.11 (m, 2H), 7.02 (td, *J* = 0.94, 7.47 Hz, 1H), 6.50 (dd, *J* = 0.78, 3.16 Hz, 1H), 5.44 (s, 2H). ^13^C NMR (126 MHz, (CD_3_)_2_SO): δ (ppm) = 139.62, 135.6, 131.1, 130.8, 130.0, 129.1, 129.0, 128.3, 127.4, 121.4, 120.6, 119.4, 110.1, 101.4, 47.8. HR-ESI-MS: calculated for C_15_H_12_Cl_2_N [*M*+H]^+^ 276.0341 (^35^Cl), 278.0312 (^37^Cl), found 276.0339 (100%), 278.0309 (70%).

##### (1-(3,4-Dichlorobenzyl)-5-nitro-1*H*-indol-3-yl)methanol (**29**)

5-Nitro-1*H*-indole-3-carbaldehyde (1.0 g, 5.3 mmol, 1.0 equiv) and 3,4-dichlorobenzyl bromide (1.5 g, 6.3 mmol, 1.2 equiv) were added as described in procedure **I-B** to a suspension of NaH (231 mg, 60% dispersion in mineral oil, 5.8 mmol, 1.1 equiv) in DMF (40 mL). The alkylation product (1.5 g, 98%) was obtained without further purification. Following procedure **I-C**, the aldehyde (2.0 g, 5.7 mmol, 1.0 equiv) was reduced to the alcohol with NaBH_4_ (693 mg, 18.3 mmol, 3.2 equiv) in methanol (600 mL) and pure product **29** (800 mg, 40% over two steps) was obtained after purification by flash column chromatography (dichloromethane/ethyl acetate 95:5) as dark-yellow crystals. ^1^H NMR (500 MHz, (CD_3_)_2_SO): δ (ppm) = 8.63 (d, *J* = 2.28 Hz, 1H), 8.03 (dd, *J* = 2.28, 9.16 Hz, 1H), 7.71 (d, *J* = 9.16 Hz, 2H), 7.59 (m, 2H), 7.18 (dd, *J* = 2.11, 8.30 Hz, 1H), 5.50 (s, 2H), 5.14 (t, *J* = 5.46 Hz, 1H), 4.70 (d, *J* = 5.46 Hz, 2H). ^13^C NMR (126 MHz, (CD_3_)_2_SO): δ (ppm) = 140.7, 139.1, 138.7, 131.3, 131.0, 130.4 130.4, 129.4, 127.6, 126.5, 119.3, 117.0, 116.7, 110.7, 54.9, 48.0. HR-ESI-MS: calculated for C_16_H_11_Cl_2_N_2_O_2_˙ [*M*–OH]˙ 333.0198 (^35^Cl), 335.0168 (^37^Cl), found 333.0193 (100%), 335.0161 (60%).

##### (1-Benzyl-5-bromo-1*H*-indol-3-yl)methanol (**30**)

1-Benzyl-5-bromo-1*H*-indole (100 mg, 0.35 mmol, 1.0 equiv) was added to a solution of POCl_3_ (40 μL, 0.43 mmol, 1.2 equiv) in DMF (3 mL), as described in procedure **I-A**. Crude 1-benzyl-5-bromo-1*H*-indole-3-carbaldehyde (**60**) was used without further purification in the next step. Compound **60** was reduced to the alcohol **30** following procedure **I-C**, using an excess of NaBH_4_ in methanol (50 mL). The pure product (65 mg, 60% over two steps) was obtained without further purification as a white solid. ^1^H NMR (500 MHz, (CD_3_)_2_SO): δ (ppm) = 7.79 (d, *J* = 1.89 Hz, 1H), 7.46 (s, 1H), 7.42 (d, *J* = 8.71 Hz, 1H), 7.30 (m, 2H), 7.22 (m, 4H), 5.38 (s, 2H), 4.91 (t, *J* = 5.48 Hz, 1H), 4.60 (d, *J* = 5.48 Hz, 2H). ^13^C NMR (126 MHz, (CD_3_)_2_SO): δ (ppm) = 138.1, 135.0, 129.0, 128.6, 128.5, 127.5, 127.1, 123.7, 121.7, 115.8, 112.3, 111.5, 55.1, 49.1. HR-ESI-MS: calculated for C_16_H_13_BrṄ [*M*–OH]˙ 298.0232 (^35^Cl), 300.0211 (^37^Cl), found 298.0226 (100%), 300.0202 (95%).

##### (1-(3,4-Dichlorobenzyl)-4-methoxy-1*H*-indol-3-yl)methanol (**31**)

4-Methoxy-1*H*-indole-3-carbaldehyde (500 mg, 2.9 mmol, 1.0 equiv) and 3,4-dichlorobenzyl bromide (820 mg, 3.4 mmol, 1.2 equiv) were added as described in procedure **I-B** to a suspension of NaH (123 mg, 60% dispersion in mineral oil, 3.1 mmol, 1.1 equiv) in DMF (14 mL). The alkylation product (200 mg, 21%) was purified by flash column chromatography (dichloromethane). Following procedure **I-C**, the aldehyde (200 mg, 0.6 mmol, 1.0 equiv) was reduced to the alcohol using NaBH_4_ (113 mg, 3.0 mmol, 5.0 equiv) in methanol (100 mL), and pure product **31** (150 mg, 74% over two steps) was obtained without further purification as a pinkish solid. ^1^H NMR (500 MHz, (CD_3_)_2_SO): δ (ppm) = 7.57 (d, *J* = 8.30 Hz, 1H), 7.45 (d, *J* = 1.94 Hz, 1H), 7.25 (s, 1H), 7.10 (dd, *J* = 1.94, 8.30 Hz, 1H), 7.00 (m, 2H), 6.49 (dd, *J* = 2.94, 5.54 Hz, 1H), 5.35 (s, 2H), 4.75 (d, *J* = 5.33 Hz, 2H), 4.66 (t, *J* = 5.54 Hz, 1H), 3.82 (s, 3H). ^13^C NMR (126 MHz, (CD_3_)_2_SO): δ (ppm) = 154.1, 139.7, 137.6, 131.1, 130.8, 129.9, 129.0, 127.4, 125.1, 122.5, 116.8, 116.4, 103.4, 99.6, 56.9, 55.2, 47.8. HR-ESI-MS: calculated for C_17_H_14_Cl_2_NȮ [*M*–OH]˙ 318.0452 (^35^Cl), 320.0423 (^37^Cl), found 318.0448 (100%), 320.0416 (70%).

##### (1-(3,4-Dichlorobenzyl)-4-fluoro-1*H*-indol-3-yl)methanol (**33**)

The aldehyde **61** (900 mg, 91%) was synthesized as a white solid, following procedure **I-B** using 4-fluoro-1*H*-indole-3-carbaldehyde (500 mg, 3.1 mmol, 1.0 equiv) and 3,4-dichlorobenzyl bromide (0.55 mL, 3.7 mmol, 1.2 equiv) in a suspension of NaH (134 mg, 3.4 mmol, 1.1 equiv) in DMF (40 mL), and purified by flash column chromatography (dichloromethane). Indole **33** was synthesized following procedure **I-C**, using **61** (100 mg, 0.3 mmol, 1.0 equiv) and NaBH_4_ (12 mg, 0.3 mmol, 1.0 equiv) in THF (25 mL). The crude product was purified using flash column chromatography (heptane/ethyl acetate 8:2) to provide **33** (25 mg, 26%). ^1^H NMR (500 MHz, (CD_3_)_2_SO): δ (ppm) = 7.64 (d, *J* = 8.30 Hz, 1H), 7.59 (d, *J* = 2.02 Hz, 1H), 7.52 (s, 1H), 7.35 (d, *J* = 8.25 Hz, 1H), 7.21 (dd, *J* = 2.02, 8.30 Hz, 1H), 7.13 (td, *J* = 5.25, 8.01 Hz, 1H), 6.83 (dd, *J* = 6.27 Hz, 1H), 5.47 (s, 2H), 4.98 (t, *J* = 5.27 Hz, 1H), 4.75 (d, *J* = 5.20 Hz, 2H). ^13^C NMR (126 MHz, (CD_3_)_2_SO): δ (ppm) = 156.8 (d, *J* = 245.14 Hz), 139.7, 139.4 (d, *J* = 12.06 Hz), 131.6, 131.3, 130.54, 129.6, 128.0 (d, *J* = 3.17 Hz), 122.7 (d, *J* = 7.59 Hz), 115.7, 115.5, 115.3 (d, *J* = 3.46 Hz), 107.1 (d, *J* = 3.30 Hz), 104.7 (d, *J* = 18.98 Hz), 56.4 (d, *J* = 1.38 Hz), 48.4. ^19^F-NMR (470 MHz, (CD_3_)_2_SO): δ (ppm) = −122.57 (dd, *J* = 5.33 Hz). HR-ESI-MS: calculated for C_16_H_11_Cl_2_FṄ [*M*–OH]˙ 306.0253 (^35^Cl), 308.0223 (^37^Cl), found 306.0248 (100%), 308.0215 (70%).

##### 1-(1-(3,4-Dichlorobenzyl)-1*H*-indol-3-yl)-*N*-(2,4-dimethoxybenzyl)methan-amine (**34**)

1-(3,4-Dichlorobenzyl)-1*H*-indole-3-carbaldehyde was prepared according to procedure **I-B**. To a solution of 1-(3,4-dichlorobenzyl)-1*H*-indole-3-carbaldehyde (0.5 g, 1.6 mmol, 1.0 equiv) in dichloroethane (33 mL), sodium triacetoxyborohydride (1.05 g, 4.9 mmol, 3.0 equiv), 2,4-dimethoxy benzylamine (0.74 mL, 4.9 mmol, 3.0 equiv), and acetic acid (0.09 mL, 1.6 mmol, 1.0 equiv) were added. The reaction mixture was stirred for 23 h and terminated by the addition of water and a saturated aqueous NaHCO_3_ solution (10 mL). The organic layer was dried over Na_2_SO_4_, filtered, and concentrated *in vacuo*. The crude material was purified by nonpressurized flash column chromatography (CH_2_Cl_2_:CH_3_OH 96:4). The product was a yellow oil (17 mg, 2%). Note: Several attempts with different pressurized column chromatography methods did not provide the clean compound. ^1^H NMR (500 MHz, CD_3_OD): δ (ppm) = 7.56 (d, *J* = 7.90 Hz, 1H), 7.39 (d, *J* = 8.30 Hz, 1H), 7.31 (t, *J* = 5.77 Hz, 2H), 7.24 (d, *J* = 1.95 Hz, 1H), 7.14 (m, 3H), 7.03 (dd, *J* = 3.40, 8.33 Hz, 1H), 6.54 (d, *J* = 2.30 Hz, 1H), 6.48 (dd, *J* = 3.53, 8.34 Hz, 1H), 5.35 (s, 2H), 4.05 (s, 2H), 3.85 (s, 2H), 3.78 (s, 3H), 3.71 (s, 3H). ^13^C NMR (126 MHz, CD_3_OD): δ (ppm) = 162.7, 160.3, 140.4, 137.9, 133.5, 132.5, 132.3, 131.8, 129.8, 129.4, 129.1, 127.8, 123.4, 120.9, 119.6, 118.0, 111.8, 111.1, 105.4, 99.4, 55.8, 55.8, 49.5, 48.3, 43.4. HR-ESI-MS: calculated for C_25_H_25_Cl_2_N_2_O_2_ [*M*+H]^+^ 455.1288 (^35^Cl), 457.1258 (^37^Cl), found 455.1290 (100%), 457.1257 (60%).

##### Synthesis and Characterization of Aminothiazoles

1-(2-Amino-4-methylthiazol-5-yl)-2-bromoethan-1-one (**62**), 2-bromo-1-(2,4-dimethylthiazol-5-yl)ethan-1-one (**63**), and 2-bromo-1-(pyridin-2-yl)ethan-1-one (**64**) were synthesized following literature procedures, and all data were consistent with the reported values.^47,58^

##### Aminothiazole Formation (Procedure **A-A**)

Synthesis of aminothiazoles followed a previously reported procedure.^47^ The impure mixture of α-bromoketones (1.0 equiv) and substituted thiourea (0.95 equiv) were dissolved in absolute ethanol. Then, *N*,*N*-diisopropylethylamine (DIPEA) (1.1 equiv) was added, and the mixture stirred for up to 3 days. TLC analysis showed that the product spot turned red after irradiation with UV light. After completion of the reaction, the solvent was evaporated and the residue diluted with ethyl acetate and filtered through Celite. The filtrate was washed with water (3×) and saturated aqueous NaCl solution (2×), dried over MgSO_4_, filtered, and concentrated *in vacuo*.

##### 5-(Pyridin-4-yl)-*N*-(4-(trifluoromethyl)phenyl)thiazol-2-amine (**36**)

Compounds 2-bromo-1-(pyridin-4-yl)ethan-1-one hydrochloride (150 mg, 0.5 mmol, 1.0 equiv) and 1-(4-(trifluoromethyl)phenyl) thiourea (117 mg, 0.5 mmol, 1.0 equiv) were mixed as described in procedure **A-A** in ethanol (15 mL). The pure product (172 mg, 100%) was afforded as a yellow solid without further purification. ^1^H NMR (500 MHz, CD_3_OD): δ (ppm) = 8.80 (d, *J* = 6.90 Hz, 2H), 8.56 (d, *J* = 6.90 Hz, 2H), 8.18 (s, 1H), 7.95 (d, *J* = 8.65 Hz, 2H), 7.66 (d, *J* = 8.65 Hz, 2H). ^13^C NMR (126 MHz, (CD_3_)_2_SO): δ (ppm) = 163.4, 148.5, 145.5, 143.9, 142.5, 126.5 (d, *J* = 3.66 Hz), 124.6 (q, *J* = 270.96 Hz), 122.4, 121.6 (q, *J* = 31.99 Hz), 117.0, 116.6. ^19^F-NMR (470 MHz, (CD_3_)_2_SO): δ (ppm) = −59.96. HR-ESI-MS: calculated for C_15_H_11_F_3_N_3_S [*M*+H]^+^ 322.0620, found 322.0613.

##### 4-(2-((4-(Trifluoromethyl)phenyl)amino)thiazol-5-yl)benzene-1,3-diol (**38**)

This compound was ordered from Princeton Biomolecular Research and analyzed by NMR and MS prior to IC_50_ determination. ^1^H NMR (500 MHz, (CD_3_)_2_SO): δ (ppm) = 10.63 (s, 1H), 10.46 (s, 1H), 9.50 (s, 1H), 8.18 (s, 1H), 7.76 (m, 2H), 7.58 (t, *J* = 8.03 Hz, 1H), 7.30 (d, *J* = 7.44 Hz, 1H), 7.25 (s, 1H), 6.37 (m, 1H), 6.31 (d, *J* = 8.46 Hz, 1H). ^13^C NMR (126 MHz, (CD_3_)_2_SO): δ (ppm) = 161.5, 158.1, 156.3, 147.4, 141.6, 130.3, 129.8 (q, *J* = 31.54 Hz), 128.9, 124.2 (q, *J* = 272.27 Hz), 120.4, 117.4 (q, *J* = 3.52 Hz), 112.8 (q, *J* = 4.03 Hz), 111.9, 107.0, 102.9, 102.3. ^19^F-NMR (470 MHz, (CD_3_)_2_SO): δ (ppm) = −61.41. HR-ESI-MS: calculated for C_16_H_12_F_3_N_2_O_2_S [*M*+H]^+^ 353.0566, found 353.0559.

##### *N*^2^-(3-Methoxyphenyl)-4′-methyl-[5,5′-bithiazole]-2,2′-diamine (**39**)

Compounds **62** (401 mg, 1.7 mmol, 1.0 equiv) and 1-(3-methoxyphenyl)thiourea (310 mg, 1.7 mmol, 1.0 equiv) were mixed as described in procedure **A-A** in ethanol (15 mL). Purification by preparative HPLC (H_2_O:CH_3_OH + 0.05% formic acid, gradient 5% to 100% CH_3_OH) afforded the product (27 mg, 7%) as a light-pink solid. ^1^H NMR (500 MHz, (CD_3_)_2_SO): δ (ppm) = 10.23 (s, 1H), 8.13 (s, 1H, formic acid), 7.54 (t, *J* = 2.21 Hz, 1H), 7.19 (t, *J* = 8.12 Hz, 1H), 7.01 (dd, *J* = 1.57, 8.12 Hz, 1H), 6.96 (s, 2H), 6.60 (s, 1H), 6.52 (dd, *J* = 2.21, 8.25 Hz, 1H), 3.76 (s, 3H), 2.32 (s, 3H). ^13^C NMR (126 MHz, (CD_3_)_2_SO): δ (ppm) = 165.8, 163.1 (formic acid), 162.1, 159.9, 144.2, 143.6, 142.3, 129.6, 114.3, 109.2, 106.8, 102.5, 99.8, 55.0, 17.1. HR-ESI-MS: calculated for C_14_H_15_N_4_OS_2_ [*M*+H]^+^ 319.0682, found 319.0674.

##### *N*^2^-(3-Chloro-2-methylphenyl)-4′-methyl-[5,5′-bithiazole]-2,2′-diamine (**40**)

Compounds **62** (752 mg, 3.1 mmol, 1.0 equiv) and 1-(3-chloro-2-methylphenyl)thiourea (619 mg, 3.1 mmol, 1.0 equiv) were mixed as described in procedure **A-A** in ethanol (31 mL). Purification by preparative HPLC (H_2_O/CH_3_OH + 0.05% formic acid, gradient from 5% to 100% CH_3_OH) afforded the product (240 mg, 23%) as a pink solid. ^1^H NMR (500 MHz, (CD_3_)_2_SO): δ (ppm) = 9.51 (s, 1H), 8.13 (s, 1H, formic acid), 7.87 (q, *J* = 3.10 Hz, 1H), 7.18 (q, *J* = 5.32 Hz, 2H), 6.93 (s, 2H), 6.58 (s, 1H), 2.31 (s, 3H), 2.27 (s, 3H). ^13^C NMR (126 MHz, CD_3_OD): δ (ppm) = 165.8, 164.3, 163.3 (formic acid), 144.3, 143.5, 141.0, 134.0, 127.2, 127.1, 124.1, 120.1, 114.2, 100.5, 17.0, 15.0. HR-ESI-MS: calculated for C_14_H_14_ClN_4_S_2_ [*M*+H]^+^ 337.0343 (^35^Cl), 339.0313 (^37^Cl), found 337.0334 (100%), 339.0302 (30%).

##### 2′,4′-Dimethyl-*N*-(4-(trifluoromethyl)phenyl)-[5,5′-bithiazol]-2-amine (**41**)

Compounds **63** (100 mg, 0.4 mmol, 1.0 equiv) and 1-(4-(trifluoromethyl)phenyl)thiourea (79 mg, 0.4 mmol, 1.0 equiv) were mixed as described in procedure **A-A** in ethanol (4 mL). Purification by flash column chromatography (petroleum benzine/ethyl acetate, gradient 0% to 100% ethyl acetate) afforded the product (112 mg, 73%) as a yellow solid. ^1^H NMR (500 MHz, (CD_3_)_2_SO): δ (ppm) = 10.76 (s, 1H), 7.85 (d, *J* = 8.50 Hz, 2H), 7.69 (d, *J* = 8.50 Hz, 2H), 7.09 (s, 1H), 2.61 (s, 3H), 2.54 (s, 3H). ^13^C NMR (126 MHz, (CD_3_)_2_SO): δ (ppm) = 162.4, 162.1, 147.3, 144.2, 142.3, 126.4 (q, *J* = 3.59 Hz), 126.4, 124.7 (q, *J* = 270.86 Hz), 121.2 (q, *J* = 31.94 Hz), 116.6, 104.8, 18.7, 17.0. ^19^F-NMR (470 MHz, (CD_3_)_2_SO): δ (ppm) = −59.88. HR-ESI-MS: calculated for C_15_H_13_F_3_N_3_S_2_ [*M*+H]^+^ 356.0497, found 356.0491.

##### 4-(2-((3,4-Dimethylphenyl)amino)thiazol-4-yl)benzene-1,2-diol (**42**)

This compound was ordered from Princeton Biomolecular Research and analyzed by NMR and MS prior to IC_50_ determination. ^1^H NMR (500 MHz, (CD_3_)_2_SO): δ (ppm) = 9.97 (s, 1H), 9.08 (s, 1H), 9.04 (s, 1H), 7.46 (dd, *J* = 2.13, 8.28 Hz, 1H), 7.39 (d, *J* = 2.13 Hz, 1H), 7.30 (d, *J* = 2.05 Hz, 1H), 7.17 (dd, *J* = 2.05, 8.28 Hz, 1H), 7.07 (d, *J* = 8.20 Hz, 1H), 6.91 (s, 1H), 6.76 (d, *J* = 8.20 Hz, 1H), 2.22 (s, 3H), 2.16 (s, 3H). ^13^C NMR (126 MHz, (CD_3_)_2_SO): δ (ppm) = 163.2, 150.7, 145.5, 145.3, 139.4, 136.8, 130.1, 129.1, 126.7, 118.5, 117.3, 115.8, 114.6, 113.6, 99.6, 20.0, 18.9. HR-ESI-MS: calculated for C_17_H_17_N_2_O_2_S [M + H]^+^ 313.1005, found 313.0992.

##### *N*-(2,5-Dimethylphenyl)-4-(pyridin-2-yl)thiazol-2-amine (**43**)

Compounds **64** (156 mg, 0.6 mmol, 1.0 equiv) and 1-(2,5-dimethylphenyl)thiourea (100 mg, 0.6 mmol, 1.0 equiv) were mixed as described in procedure **A-A** in ethanol (5 mL). The pure product (157 mg, 95%) was obtained after workup as an orange-brown solid. ^1^H NMR (500 MHz, (CD_3_)_2_SO): δ (ppm) = 9.37 (s, 1H), 8.57 (dt, *J* = 1.27, 4.78 Hz, 1H), 7.87 (m, 2H), 7.78 (m, 1H), 7.47 (s, 1H), 7.30 (ddd, J = 2.24, 5.57, 6.63 Hz, 1H), 7.11 (d, *J* = 7.55 Hz, 1H), 6.85 (dd, *J* = 1.27, 7.55 Hz, 1H), 2.30 (s, 3H), 2.23 (s, 3H). ^13^C NMR (126 MHz, (CD_3_)_2_SO): δ (ppm) = 165.9, 152.2, 150.1, 149.4, 139.2, 137.4, 135.6, 130.6, 126.1, 124.4, 122.6, 122.0, 120.2, 106.7, 21.0, 17.7. HR-ESI-MS: calculated for C_16_H_16_N_3_S [*M*+H]^+^ 282.1059, found 282.1054.

##### *N*-(3-Chloro-2-methylphenyl)-5-(pyridin-2-yl)thiazol-2-amine (**44**)

Compounds **64** (20 mg, 0.1 mmol, 1.0 equiv) and 1-(3-chloro-2-methylphenyl)thiourea (20 mg, 0.1 mmol, 1.0 equiv) were mixed as described in procedure **A-A** in ethanol (5 mL). The pure product (20 mg, 66%) was obtained after workup as an orange solid. ^1^H NMR (500 MHz, (CD_3_)_2_SO): δ (ppm) = 9.60 (s, 1H), 8.57 (d, *J* = 4.65 Hz, 1H), 8.01 (d, *J* = 8.02 Hz, 1H), 7.86 (m, 2H), 7.53 (s, 1H), 7.29 (ddd, *J* = 1.72, 5.80, 6.99 Hz, 1H), 7.25 (d, *J* = 8.02 Hz, 1H), 7.20 (d, *J* = 7.44 Hz, 1H), 2.34 (s, 3H). ^13^C NMR (126 MHz, (CD_3_)_2_SO): δ (ppm) = 165.2, 152.1, 150.1, 149.4, 140.9, 137.3, 134.0, 127.4, 126.9, 124.0, 122.6, 120.3, 120.0, 107.5, 15.0. HR-ESI-MS: calculated for C_15_H_13_ClN_3_S [*M*+H]^+^ 302.0513 (^35^Cl), 304.0484 (^37^Cl), found 302.0490 (100%), 304.0456 (30%).

##### *N*-(3,4-Dimethylphenyl)-4-(pyridin-2-yl)thiazol-2-amine (**46**)

Compounds **64** (390 mg, 1.39 mmol, 1.0 equiv) and 1-(3,4-dimethylphenyl)thiourea (250 mg, 1.39 mmol, 1.0 equiv) were mixed as described in procedure **A-A** in ethanol (10 mL). The pure product (388 mg, 95%) was obtained after workup as a yellow-brown solid. ^1^H NMR (500 MHz, (CD_3_)_2_SO): δ = 10.13 (s, 1H), 8.55 (m, 1H), 7.97 (d, *J* = 7.85 Hz, 1H), 7.90 (td, *J* = 1.89, 7.57 Hz, 1H), 7.48 (m, 2H), 7.42 (d, *J* = 1.89 Hz, 1H), 7.31 (ddd, *J* = 1.07, 4.80, 7.46 Hz, 1H), 7.10 (d, *J* = 8.24 Hz, 1H), 2.23 (s, 3H), 2.18 (s, 3H). ^13^C NMR (126 MHz, (CD_3_)_2_SO): δ = 163.8, 152.2, 150.3, 149.4, 139.0, 137.5, 136.7, 130.0, 129.2, 122.7, 120.4, 118.5, 114.7, 106.5, 19.9, 18.8. HR-ESI-MS: calculated for C_16_H_16_N_3_S [*M*+H]^+^ 282.1059, found 282.1047.

##### Method I: Cell Culture and Growth-Inhibition Assay of *P. falciparum*

*P. falciparum* 3D7 parasites (Wellcome Trust Dundee) and all derived transgenic cell lines (see below) were maintained in continuous culture at 37 °C and an atmosphere consisting of 90% N_2_, 5% O_2_, and 5% CO_2_, as described previously^48^ with modifications.^49^ Parasites were maintained in 25 mM 4-(2-hydroxyethyl)-1-piperazineethanesulfonic acid (HEPES) and 11.9 mM sodium bicarbonate buffered RPMI 1640 medium supplemented with d-glucose (11 mM), hypoxanthine, AlbuMAX II (0.5% w/v), and 10 μg/mL gentamicin. Fresh 0+ blood was provided by the Brazilian blood bank ProSangue (Brazil) and in agreement with the ethics committee at ICB-USP. The antiplasmodial effect of the *de novo* synthesized compounds was validated against *P. falciparum* 3D7 strain conducting SYBR Green I (Invitrogen) drug assays, as reported.^50,51^ Briefly, 2-fold serial dilutions of compounds were prepared in 96-well plates in a range of 200 to 0.4 μM in triplicate and incubated for 96 h under normal growth conditions using an initial parasitemia of 0.5% and a hematocrit of 2% in a volume of 100 μL per well. Parasite proliferation was determined by measuring DNA load via fluorescence in the wells through addition of 100 μL lysis buffer supplemented with SYBR Green I (0.02% v/v) and incubation for 1 h at room temperature in the dark. Fluorescence was quantified using a CLARIOstar plate reader (BMG LABTECH, Germany) at excitation and emission wavelength bands of 485 (±9) and 530 (±12) nm, respectively. Focal and gain adjustment was performed using the nontreated controls (highest expected fluorescence signal). Data was acquired via the CLARIOstar (V5.20) and MARS software, manually normalized, and plotted using the nonlinear regression curve fit implemented in GraphPad Prism, as described below in more detail (version 7.00 for Windows, GraphPad Software, La Jolla, California, USA, www.GraphPad.com). Nontreated parasites, highest solvent concentration on parasites, and highest drug concentration in medium were used as controls for maximal growth, solvent control, and native drug fluorescence, respectively.

##### Method II: Cell Culture and Growth-Inhibition Assay of *P. falciparum*

*P. falciparum* strain 3D7 was obtained through the MR4 as part of the BEI Resources Repository, NIAID, NIH (www.mr4.org), and strain NF54 was generously supplied by D.A. Fidock (Columbia University). Parasites were cultured in a 2% suspension of human erythrocytes and RPMI 1640 (Sigma) medium supplemented with 27 mM sodium bicarbonate, 11 mM glucose, 5 mM HEPES, 1 mM sodium pyruvate, 0.37 mM hypoxanthine, 0.01 mM thymidine, 10 μg/mL gentamicin, and 0.5% AlbuMAX (Gibco) at 37 °C, 5% O_2_/5% CO_2_/90% N_2_ atmosphere, as previously described.^52,53^ Asynchronous cultures of *P. falciparum* 3D7 and NF54 were diluted to 1% parasitemia and treated with DXPS inhibitors at concentrations ranging from 97.7 nM to 250 μM. Assays were performed in opaque 96-well plates in 100 μL culture volume. After 3 days, parasite growth was quantified by measuring DNA content using PicoGreen (Life Technologies), as described.^54^ Fluorescence was measured on a FLUOstar Omega microplate reader (BMG LABTECH) at 485 nm excitation and 528 nm emission.

##### Method III: Cell Culture and Growth Inhibition Assay of *P. falciparum*

Two laboratory strains of *P. falciparum*, the chloroquine-sensitive 3D7 and the multiresistant Dd2 (obtained from MR4), were kept in complete culture medium (RPMI 1640, 25 mM HEPES, 2 mM l-glutamine, 50 μg/mL gentamicin, and 0.5% w/v AlbuMAX) at 37 °C, 5% CO_2_, and 5% oxygen at 2.5% hematocrit with daily change of medium.^52^ All compounds were dissolved in DMSO at stock solutions between 25 and 100 mM; the reference drug chloroquine diphosphate (MW: 515.86 g/mol) was diluted in distilled water. Further dilutions were prepared in complete culture medium so that final concentrations of solvent did not interfere with parasite growth. Antiplasmodial activity of the different compounds was tested in a drug-sensitivity assay against the two laboratory strains using the histidine-rich protein 2 (HRP2) assay, as described previously.^55,56^ In brief, 96-well plates were precoated with the different compounds in a threefold dilution before ring-stage parasites were added in complete culture medium at a hematocrit of 1.5% and a parasitemia of 0.05% in a total volume of 225 μL per well. After 3 days of incubation, plates were frozen until analyzed by HRP2-ELISA. All compounds were evaluated in duplicate in at least two independent experiments. Statistics: 50% IC_50_ was determined by analyzing the nonlinear regression of log concentration–response curves using the drc package v0.9.0 of R v2.6.1.^57^

##### Hep G2 Cell Culture and Viability Assay as Counter Screens

HepG2 cells (2 × 105 cells per well) were seeded in 24-well, flat-bottomed plates. Culturing of cells, incubations, and OD measurements were performed as described previously with small modifications.^58^ 24 h after seeding the cells, the incubation was started by the addition of compounds in a final DMSO concentration of 1%. The metabolic activity of the living cell mass was determined after 48 h. At least three independent measurements were performed for each compound. The IC_50_ values were determined during logarithmic growth using GraphPad Prism software. All experiments were performed at least in triplicate, and data reported represent the mean ± SD.

##### Metabolic Stability Tests in the Human Liver S9 Fraction

For the evaluation of combined phase I and phase II metabolic stability, the compound (1 μM) was incubated with 1 mg/mL pooled liver S9 fraction (XenoTech), 2 mM NADPH, 1 mM UDPGA, 10 mM MgCl_2_, 5 mM GSH, and 0.1 mM PAPS at 37 °C for 0, 5, 15, 30, and 60 min. The metabolic stabilities of testosterone (1 μM), verapamil (1 μM), and propranolol (1 μM) were determined in parallel to confirm the enzymatic activity of the S9 fraction. The incubation was stopped by precipitation of S9 enzymes with two volumes of cold acetonitrile containing internal standard (150 nM diphenhydramine). Samples were stored on ice for 10 min, and precipitated protein was removed by centrifugation (15 min, 4 °C, 4000 rpm). The concentration of the remaining test compound at the different time points was analyzed by LC-MS/MS (TSQ Quantum Access MAX, Thermo Fisher, Dreieich, Germany) and used to determine half-life (*t*_1/2_).

##### Kinetic Solubility Determination

The desired compounds were sequentially diluted in DMSO in a 96-well plate. 3 μL of each well was transferred into another 96-well plate and mixed with 147 μL of PBS. Plates were shaken for 5 min at 600 rpm at room temperature (r.t.), and the absorbance at 620 nm was measured. Absorbance values were normalized by blank subtraction and plotted using GraphPad Prism 8.4.2 (GraphPad Software, San Diego, California, USA). Solubility (*S*) was determined based on the First X value of AUC function using a threshold of 0.005.

##### IDP Rescue Assay in *P. falciparum*

*P. falciparum* cultures were treated as described in method II. For IDP (Isoprenoids.com) rescue experiments, 125 μM IDP was added to the appropriate wells for the duration of the experiment. The well-described DXR inhibitor FSM was used as a positive control. IC_50_ values were calculated by nonlinear regression analysis using GraphPad Prism software. All experiments were performed at least in triplicate, and data reported represent the mean ± SD.

##### *P. falciparum* Sample Preparation for Mass Spectrometry Analysis

*P. falciparum* strain 3D7 was cultured at 37 °C in 30 mL volumes in 100 mm tissue culture dishes (Techno Plastic Products) at 4% hematocrit until >6.5% parasitemia. Cultures were synchronized until >75% of parasites were in the ring-stage growth and then treated for 12 h with or without compound **1** at 7.65 μM (5× the 3D7 IC_50_) in triplicate. Cultures were lysed with 5% saponin, the parasite pellets washed with 1× phosphate-buffered saline (PBS; Gibco), and the pellets were stored at −80 °C.

##### *E. coli* Sample Preparation for Mass Spectrometry Analysis

Overnight cultures of *E. coli* Δ*T*olC were diluted 1:1000 in LB media and grown at 37 °C until reaching the mid logarithmic phase (OD_600_ = 0.67–7.2). Cultures were then treated with or without compound **1** at 48 μM (10× the IC_50_) in triplicate for 2 h while shaking at 37 °C. For normalization, the OD_600_ was determined after 2 h of treatment with the inhibitor. Cells were pelleted by centrifugation for 5 min at 3000*g* at 4 °C. The supernatants were removed, and cells were washed twice with 1× PBS. The supernatants were removed, and the pellets were stored at −80 °C until analysis.

##### LC-MS/MS Analysis

Metabolites were extracted via the addition of glass beads (212–300 u) and 600 μL of chilled H_2_O:chloroform:methanol (3:5:12 v/v) spiked with PIPES (piperazine-*N*,*N*′-bis(2-ethanesulfonic acid) as internal standard. The cells were disrupted with the TissueLyser II instrument (Qiagen) using a microcentrifuge tube adaptor set prechilled for 2 min at 20 Hz. The samples were then centrifuged at 16,000*g* at 4 °C, the supernatants collected, and pellet extraction repeated once more. The supernatants were pooled, and 300 μL of chloroform and 450 μL of chilled water were added to the supernatants. The tubes were vortexed and centrifuged. For *E. coli* samples, the upper layer was transferred to a new tube and dried using a speed-vac. For *P. falciparum* samples, the upper layer was transferred to a 2 mL tube PVDF filter (Thermo Fisher, F2520-5) and centrifuged for 5 min at 4000*g* at 4 °C. The samples were then transferred to new tubes and dried using a speed-vac. Both *E. coli* and *P. falciparum* pellets were redissolved in 100 μL of 50% acetonitrile.

For pyruvate analysis from the *E. coli* samples, the LC separation was done on the Shimadzu Nexera II using the Poroshell 120 (Agilent, 2.7 μm, 150 × 2.1 mm) flowing at 0.5 mL/min. The gradient of the mobile phases A (20 mM ammonium acetate, pH 9.8, 5% ACN) and B (100% acetonitrile) was as follows: 85% B for 1 min, to 40% B in 9 min, hold at 40% B for 2 min, and then back to 85% B in 0.5 min. For the TCA/glycolysis/pentose phosphate pathway metabolites from the *P. falciparum* samples, the same mobile phases were used on a Luna-NH2 column (3 μm, 150 × 2 mm, Phenomenex) at a flow rate of 1 mL/min. The gradient was as follows: 80% B for 1 min, to 30% B in 6 min, hold at 30% B for 5 min, and then back to 80% B in 0.5 min. Finally, for the MEP metabolites, the same column and mobile phases were used as the latter, except the gradient as follows: 60% B for 1 min, to 6% B in 3 min, hold at 6% B for 5 min, and then back to 60% B in 0.5 min. The LC system was interfaced with a Sciex QTRAP 6500+ mass spectrometer equipped with a TurboIonSpray (TIS) electrospray ion source. Analyst software (version 1.6.3) was used to control sample acquisition and data analysis. The QTRAP 6500+ mass spectrometer was tuned and calibrated according to the manufacturer’s recommendations. Metabolites were detected using MRM transitions that were previously optimized using standards. The instrument was set up to acquire in negative mode. For quantification, an external standard curve was prepared using a series of standard samples containing different concentrations of metabolites and fixed concentration of the internal standard. The limit of detection for deoxyxylulose 5-phosphate (DOXP), methylerythritol phosphate (MEP), cytidine diphosphate methylerythritol (CDP-ME), and methylerythritol cyclodiphosphate (MEcPP) was 0.0064 μM for a 10 μL injection volume. The limits of detection for a 5 μL injection volume for the TCA cycle and glycolytic and pentose phosphate metabolites were as follows: aconitate, malate, succinate = 0.31 μM; glucose-6-phosphate and glycerol-3-phosphate = 0.78 μM; citrate, glucose-1-phosphate, and fructose-6-phosphate = 1.56 μM; ribose-5-phosphate and ribulose-5-phosphate = 2.34 μM; 2-phospho gylceric acid, 3-phospho glyceric acid, and lactate = 3.12 μM; fumarate, pyruvate, phosphoenolpyruvate, and sedoheptulose-7-phosphate = 6.25 μM. *t* tests were used to test for significance between untreated (UNT) and drug-treated bacteria (Prism).

##### Generation of Transgenic Parasite Lines

The open reading frame (ORF) encoding 1-deoxy-d-xylulose-5-phosphate synthase (*Pf*DXPS; PF3D7_1337200) was amplified from genomic DNA isolated from unsynchronized *P. falciparum* 3D7 cultures using the Platinum PCR SuperMix High Fidelity (Invitrogen). Forward and reverse primers contained restriction sites for *Kpn*I and *Avr*II in sense and antisense orientation, respectively (oligonucleotides *pf*DXPS-Kpn1-S: GAGAGGTACCATGATTTTTAATTATGTGTTTTTTAAGAAC; *pf*DXPSmyc-Avr2-AS: GAGACCTAGGTTACAGGTCCTCCTCTGAGATCAGCTTCTGCTCGCCTGTAGGATTATTTTTAAGATAATTTTTAATTCTATTGAC). PCR products and the transfection vector pARL1a-hDHFR P*f*ARG-GFP were digested with *Kpn*I and *Avr*II, purified (Gel extraction Kit and PCR purification kit; Qiagen), and cloned into the transfection vector yielding the construct for overexpression pARL-DXS-strep. XL 10-Gold *E. coli* ultracompetent cells (Agilent Technologies) were transformed with the generated construct to amplify the plasmid and colonies checked using restriction analysis and sequencing of the plasmid. Bacterial clones carrying the overexpression construct and the empty pARL1a vector (MOCK plasmid) were amplified in overnight cultures, isolated (Plasmid Maxi Kit; QIAGEN), and subsequently used to transfect *P. falciparum* 3D7 ring-stage parasites, as already described.^59^ Briefly, 120 μg of plasmid DNA was centrifuged and air-dried, and the pellet was resuspended in TE buffer and cytomix reagent and mixed with the *P. falciparum* culture. Parasites were transfected using electroporation and selected with WR 99210. The generation and characterization of the *Pf*TPK overexpression cell line has been reported before.^60^

##### Quantification of DXPS Overexpression in *P. falciparum* 3D7 Transgenic Cell Lines

Total RNA was isolated from *P. falciparum* using TRIzol Reagent (Invitrogen). First-strand cDNA synthesis was prepared using a RevertAid H Minus First Strand cDNA Synthesis Kit (Thermo Fisher Scientific) from a total of 1 μg of RNA. Real-time PCR was performed with 1 μL of cDNA, 7.5 μL of SYBR Green Fast Master Mix (Applied Biosystems), 0.45 μL (10 μM) each of the forward and reverse primers, and 5.6 μL of DEPC-treated water, on an Applied Biosystems QuantStudio 3 Real-Time PCR System (Thermo Fisher Scientific). After cycling, melting curve analysis was performed. The relative transcription levels of MOCK (control) and the DXS (DXS-pARL) were determined by the ΔΔCT method.^61^ Target transcription levels were normalized to the housekeeping gene, *Pf*Aldolase, as an endogenous control reference as reported before.^60^ Sequences of primers were as follows: *Pf*Aldolase forward: tgtaccaccagccttaccag; reverse: ttccttgccatgtgttcaat; DXS-pARL forward: tcagtggagagggtgaaggt; reverse: gttggccatggaacaggtag. Three technical replicates from three biological replicates were performed for each experiment. Expression was found to be six times higher in comparison to the MOCK control cell line.

##### Growth-Inhibition Assay of Transgenic *P. falciparum* Cell Lines

*P. falciparum* cultures were treated as described in method I. For target specification toward *Pf*TPK and *Pf*DXPS, compounds were tested in comparison against overexpressing cell lines and the respective MOCK line containing only the transfected vector backbone. Parasite viability/proliferation was determined. Analysis of the IC_50_ values and interpretation of the curves was performed, as described in Section 4.1.2.44.

##### Nonlinear Regression Fit and Analysis of Dose–Response Drug Assays of Compounds **1**, **2**, and **3**

Nonlinear regression as implemented in GraphPad Prism 7.00 (log(inhibitor) vs response – variable slope (four parameters)) was used to fit the measured data to interpolate the IC_50_ value from the curve. No specific model was applied. Data was preprocessed by normalizing according to the following formula:

**eq 1:** Fluorescence data normalization
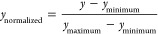
where *y* is the fluorescence signal in each well, *y*_minimum_ is the background fluorescence, and *y*_maximum_ is the highest measured fluorescence signal in the untreated wells. Drug concentrations (in μM) were transformed to the log(10)of the values. Means of each independent experiment were plotted as individual values, and the SD of the mean from the means shown as error bars. The 95% CI is indicated as measure of error for the calculated IC_50_s. In case of transgenic cell lines, the built-in comparison Akaike’s information criteria (AICc) method was applied to the Log IC_50_s of the different cell lines. The test calculates a percentage probability of the simpler model “Log IC_50_ is the same for datasets” being correct. The test for *homoscedasticity* was performed to confirm if no weighting of values was appropriate.

##### Computational Methods, Homology Model of *Pf*DXPS

As template for the homology model of *Pf*DXPS, the crystal structure of DXPS from *D. radiodurans* (2o1x, chain B) was used because the corresponding region in the active center of the likewise related DXPS from *E. coli* (2o1s) strongly deviates in comparison to the DXPS from *M. tuberculosis*. Subsequently, the actual homology model was generated by the SWISS-MODEL web service, yielding a QMEAN4 value of −6.43.^62,63^ The magnesium atom was added manually using the coordinates from the template.

##### Docking Studies

The AlphaFold-^64^ predicted structure of pfDXPS was used in the molecular modeling studies (UniProt^65^ accession code: W7KAR5). The structure was loaded into SeeSAR v13.0,^66^ and the binding sites were predicted in the Binding Site module of the software. The ThDP pocket connected to the substrate was the highest scoring (Druggability Score) and was therefore selected for the docking studies. In the Docking Module, 50 poses were generated using the Standard Docking procedure and rescored using the HYDE scoring function. Ligand pose diagrams were generated using the PoseEdit^67^ software available on the proteins.plus Web server.

## Supporting Information Available

Homology model; detailed assay results; and structures, inhibition data, *t* tests, and docking scores (PDF)
NMR, HRMS, and HPLC spectra of all tested compounds and *Pf*DXPS homology model based on structure 2O1X (PDF)

## Abbreviations used

ACTs: artemisinin-based combination therapies
DIPEA: *N,N*-diisopropylethylamine
DMADP: dimethylallyl diphosphate
DOXP: 1-deoxy-d-xylulose 5-phosphate
DXPS: 1-deoxy-d-xylulose-5-phosphate synthase
DXR: 1-deoxy-d-xylulose 5-phosphate reductoisomerase
ERAD: endoplasmic reticulum assisted degradation
FabI: enoyl-[acyl-carrier-protein] reductase
FAS: fatty acid biosynthesis pathway
FSM: fosmidomycin
GAP: glyceraldehyde 3-phosphate
Hep G2: human hepatocytes
HEPES: 4-(2-hydroxyethyl)-1-piperazineethanesulfonic acid
HRP2: histidine-rich protein 2
HTS: high-throughput screening
Huh7: human hepatoma cells
IDP: isopentenyl diphosphate
KasA: β-ketoacyl ACP synthase
LBVS: ligand-based virtual screening
MEP: methylerythritol 4-phosphate
NaHMDS: sodium bis(trimethylsilyl)amide
OAc: acetate
ORF: open reading frame
PBMN: polymyxin B nonapeptide
PPB: polypharmacology browser
TBDMS: *tert*-butyldimethylsilyl chloride
TBME: *tert*-butyl methyl ether
TCA: tricarboxyclic acid
ThDP: thiamine diphosphate
TPK: thiamine pyrophosphokinase
VCP: valosin-containing protein
WHO: World Health Organization.
