# Supplementary material for: High Target Homology Does Not Guarantee Inhibition: Aminothiazoles Emerge as Inhibitors of Plasmodium falciparum
Source: ACS Infect Dis. 2024 Feb 17;10(3):1000–22. doi: 10.1021/acsinfecdis.3c00670 (PMC10928712; doi:10.1021/acsinfecdis.3c00670)
Supplement: Supplementary file 1 — id3c00670_si_001.pdf [file id3c00670_si_001.pdf]

Supplementary Information

High Target Homology does not Guarantee Inhibition: Aminothiazoles Emerge as Inhibitors of *Plasmodium falciparum*

Sandra Johannsen[1,2,†], Robin M. Gierse[1,2,3,†], Arne Krüger[4], Rachel L. Edwards[5], Vittoria Nanna[1], Anna Fontana[1], Di Zhu[1,3], Tiziana Masini[3], Lais Pessanha de Carvalho[6], Mael Poizat[7], Bart Kieftenbelt[7], Dana M. Hodge[8], Sophie Alvarez[9], Daan Bunt[3], Antoine Lacour[1,2], Atanaz Shams[1,2], Kamila Anna Meissner[4], Edmarcia Elisa de Souza[4], Melloney Dröge[7], Bernard van Vliet[7], Jack den Hartog[7], Michael C. Hutter[10], Jana Held[6], Audrey R. Odom John[8], Carsten Wrenger[4], Anna K. H. Hirsch[1,2,3,*]

1 Helmholtz Institute for Pharmaceutical Research Saarland (HIPS) – Helmholtz Centre for Infection Research (HZI), Campus Building E8.1, 66123 Saarbrücken, Germany

2 Saarland University, Department of Pharmacy, Campus Building E8.1, 66123 Saarbrücken, Germany

3 Stratingh Institute for Chemistry, University of Groningen, Nijenborgh 7, 9747 AG Groningen, The Netherlands

4 Unit for Drug Discovery, Department of Parasitology, Institute of Biomedical Sciences, University of São Paulo, Av. Prof. Lineu Prestes 1374, 05508-000 São Paulo-SP, Brazil

5 Department of Pediatrics, Washington University School of Medicine, Saint Louis, Missouri 63110, United States

6 Institute of Tropical Medicine, University of Tübingen, Wilhelmstraße 27, 72074 Tübingen, Germany

7 Symeres, Kadijk 3, 9747 AT Groningen, The Netherlands

8 Department of Pediatrics, Children's Hospital of Philadelphia, Perelman School of Medicine, University of Pennsylvania, Philadelphia PA 19104, United States

9 Proteomics & Metabolomics Facility, Center for Biotechnology, Department of Agronomy and Horticulture, University of Nebraska-Lincoln, Lincoln, Nebraska 68588, United States

10 Center for Bioinformatics, Saarland University, Campus Building E2.1, 66123 Saarbrücken, Germany

Table of content

Alternative synthetic schemes 2
Solubility test of oximes 4
Comparison of actives sites of *Mt*DXPS and *Pf*DXPS 4
Results IDP rescue assay against *P. falciparum* 3D7 6
Results LC-MS assay 9
Results of IC_{50} determination against *Pf*DXPS and *Pf*TPK overexpressing *P. falciparum* 3D7 13
Inhibition data for all compounds 14
PPB results for the Oxime-class 23
PPB results for the Indole-class 23
Docking and scoring 25
Human off-target enzymes 29
Table of compounds as SMILES with biological activity and PAINS count. 31
References 32

Alternative synthetic schemes

The syntheses of oximes **2**, **9**, **10** and **12** have been reported elsewhere.[1]

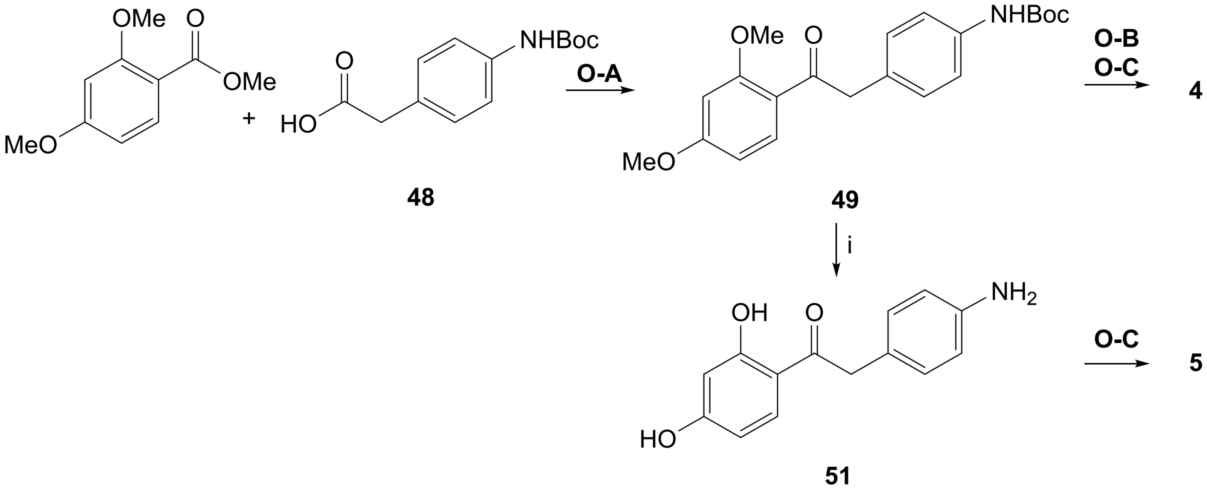

Scheme S1. Synthesis of oxime **5** not following general procedures **O-B**. i) Pyridine hydrochloride, microwave, 15 W, 110 °C, 5 min.

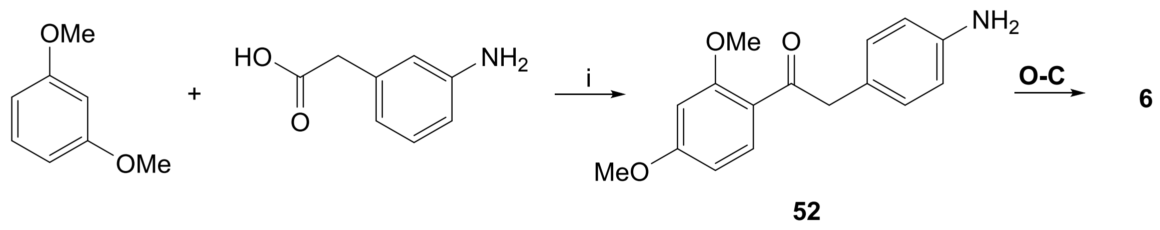

Scheme S2. Synthesis of oxime **6** not following general procedures **O-A** and **O-B**. i) Polyphosphoric acid, dichloroethane, 85 °C, 3 h.[2]

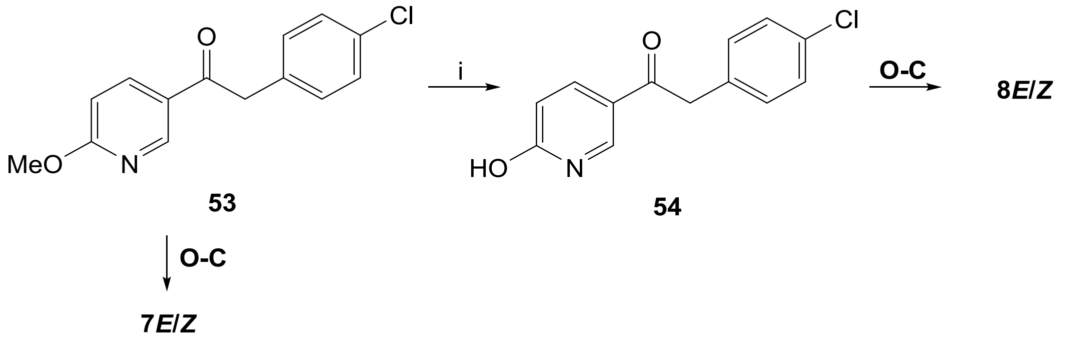

Scheme S3. Synthesis of oximes **8*E*** and **8*Z*** not following general procedure **O-B**. i) LiCl (5.0 equiv.), *p*-toluenesulfonic acid (5.0 equiv.), dimethylformamide, 150 °C, 24 h.

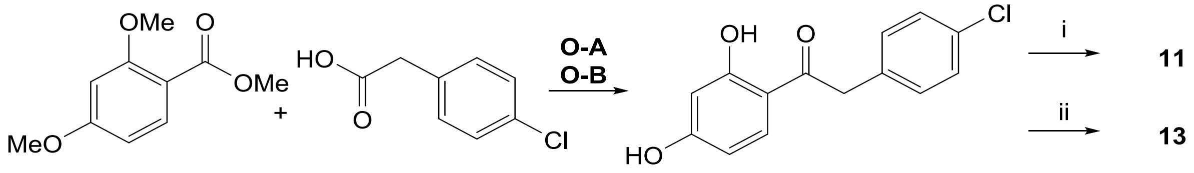

Scheme S4. Synthesis of oximes **11** and **13** not following general procedure **O-C**. i) CH_3ONH_2 (2.0 equiv.), MeOH/pyridine (10:1), $NaSO_4$ (2.5 equiv.), reflux, 18 h. ii) $NaBH_4$ (1.5 equiv.), MeOH, 25 °C, 3 h.

The syntheses of indoles **3**, **16**, **17**, **20** and **32** have been reported elsewhere.[1]

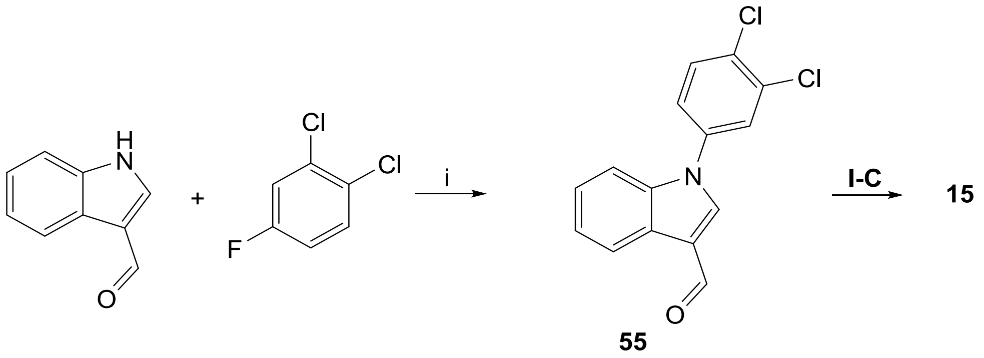

Scheme S5. Synthesis of indole **15** not following general procedures **I-A** and **I-B**. i) NaH (2.4 equiv.), 190 °C, 24 h.

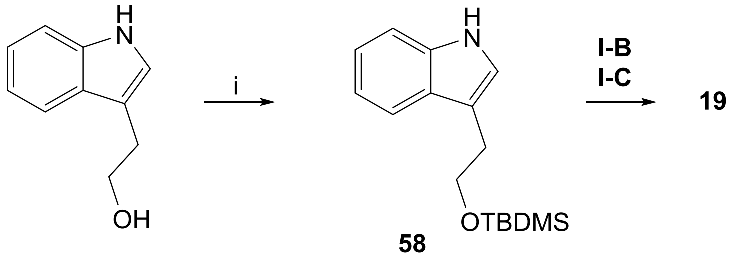

Scheme S6. Synthesis of indole **19** not following general procedures **I-A**. i) *tert*-Butyldimethylsilyl (TBDMS) chloride (1.5 equiv.), imidazole (1.5 equiv.), dichloromethane, 25 °C, 24 h.

Solubility test of oximes

Table S1. Solubility in PBS buffer and 2% DMSO of new oxime derivatives in comparison to the original hit **2**.

Compound	Max. solubility [µM]
**2**	114.2 ± 6.7
**4**	202.6 ± 1.6
**5**	193.6 ± 95.7
**6**	119.4 ± 27.4

Comparison of actives sites of *Mt*DXPS and *Pf*DXPS

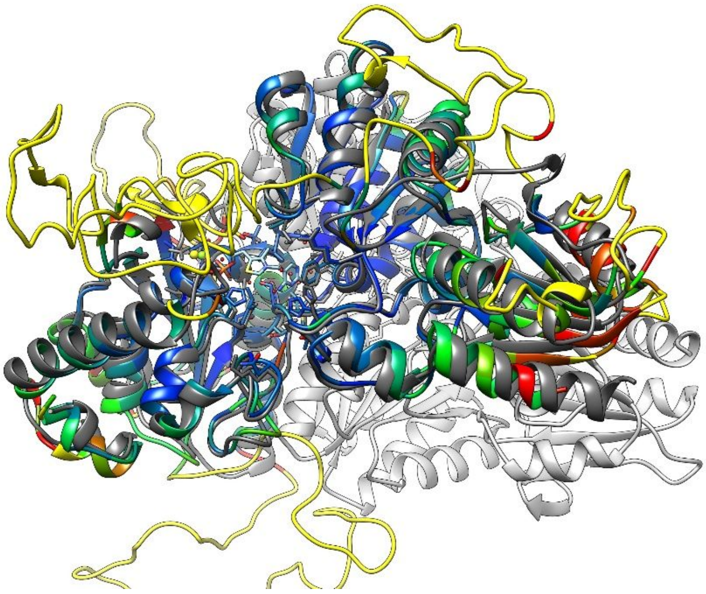

Figure S1. Superposition of *Mt*DXPS crystal structure (grey, PDB: 6a9h) with the *Pf*DXPS homology model based on *dr*DXPS structure 2o1x. The core of the enzyme shows a high similarity with the *Mt*DXPS structure. *Pf*DXPS is colored by Cα-RMSD to the *Mt*DXPS structure, from blue = low RMSD via green to red = high RMSD; yellow: no corresponding amino acids in *Mt*DXPS structure. Typical for *Plasmodium*

falciparum, its structure shows many additional loops on the surface of the protein.

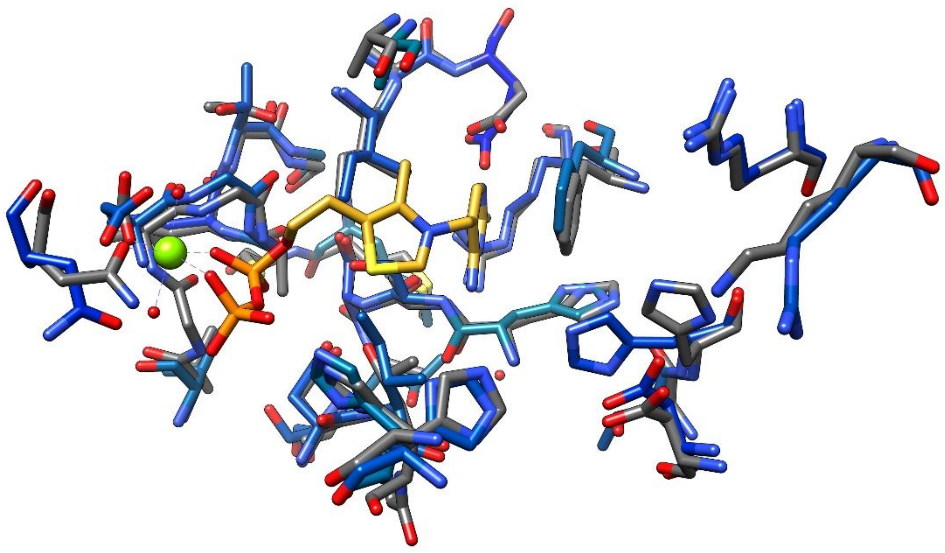

Figure S2. Superposition of the active site of *Mt*DXPS and *Pf*DXPS. The *Pf*DXPS amino acids are colored by Cα-RMSD to the *Mt*DXPS structure, from blue = low RMSD via green to red = high RMSD; the carbon atoms of the ThDP ligand are shown in gold; heteroatoms are colored in red for oxygen, blue for nitrogen and yellow for sulfur atoms; the green sphere represents a Mg^{2+}-ion. Most amino acids are found in identical arrangements, except His941 (corresponding to His416 of *Mt*DXPS) at the bottom right, which is predicted to be in a different rotameric state.

Docking analysis

For the oxime series, we found that compound **2** was predicted to bind in the ThDP binding site. H-bonding interactions between one of the phenol OH groups and the backbone carbonyl of Val660 as well as between the oxime oxygen atom and the side-chain of His941 were identified (**Figure S3**). In the predicted binding mode of compound **13**, the two aromatic rings have switched positions compared to the binding mode of compound **2** and three H-bonds are now formed with His941, Gly503 and Ser532 (**Figure S4**). This difference contributes to the higher predicted affinity of compound **2**, which again follows the trend observed in the experimental data.

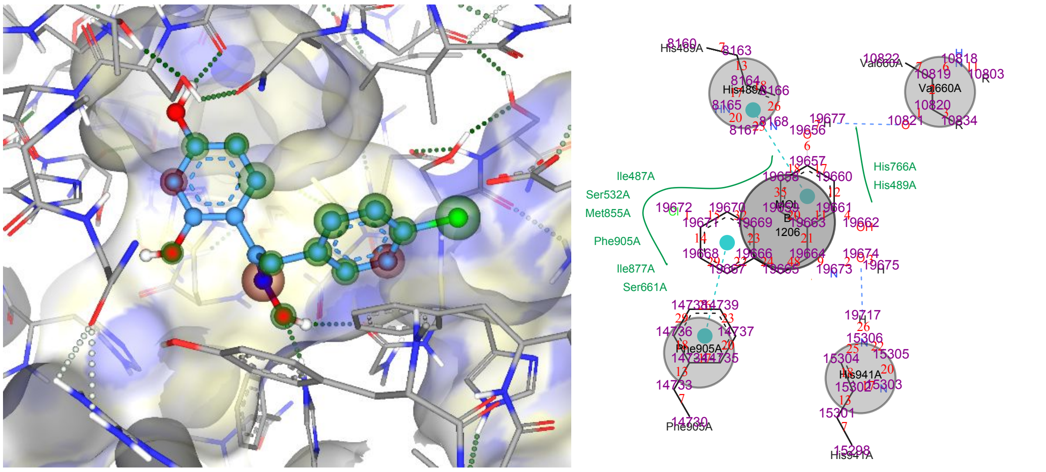

Figure S3: Binding mode and pose diagram of compound **2**. Protein surface representation is clipped for clarity. Green and red spheres represent positive or negative contribution to the predicted affinity, respectively.

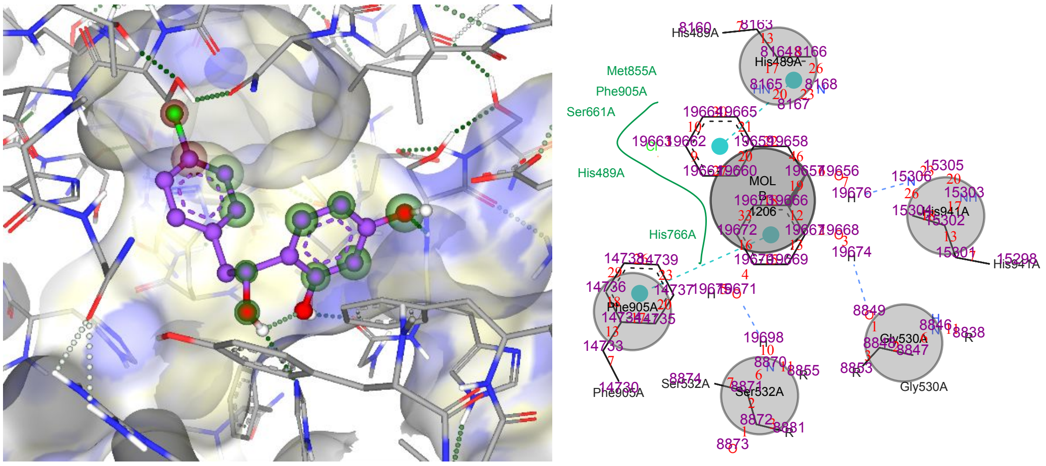

Figure S4: Binding mode and pose diagram of compound **13**. Protein surface representation is clipped for clarity. Green and red spheres represent positive or negative contribution to the predicted affinity, respectively.

For the indole series, we found that compound **3** was predicted to bind in the ThDP binding site. The OH group of the hydroxymethylene moiety was predicted to make a H-bond interaction with Val660 (**Figure S5**). Compound **33** was predicted to have the same binding mode as compound 3, with the additional fluorine atom contributing positively to the predicted binding affinity (**Figure S6**), as observed in the experimental data.

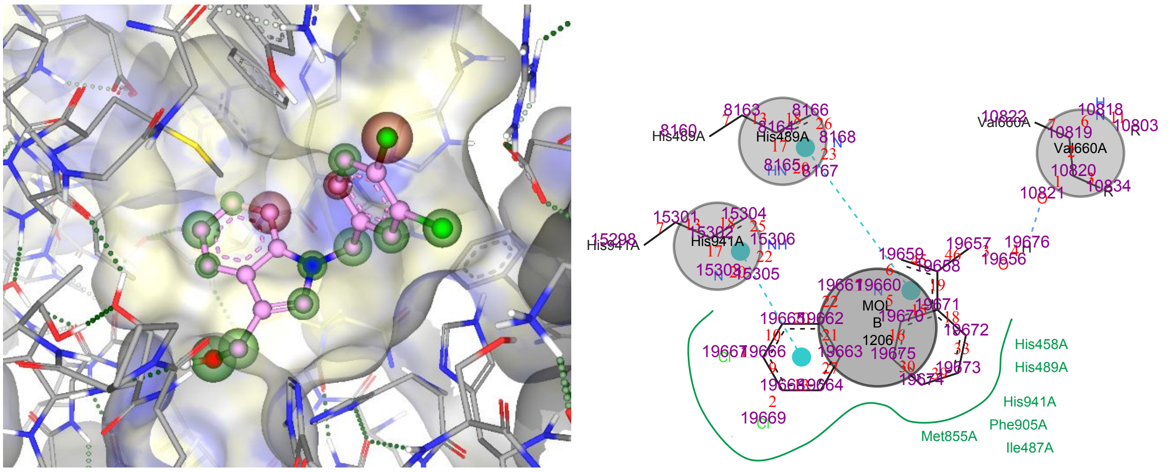

Figure S5: Binding mode and pose diagram of compound **3**. Protein surface representation is clipped for clarity. Green and red spheres represent positive or negative contribution to the predicted affinity, respectively.

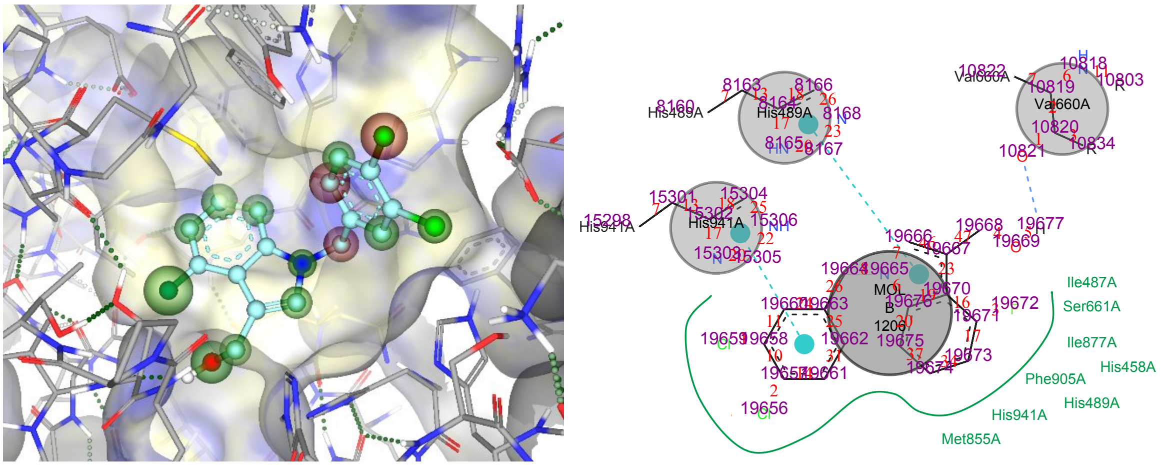

Figure S6: Binding mode and pose diagram of compound **33**. Protein surface representation is clipped for clarity. Green and red spheres represent positive or negative contribution to the predicted affinity, respectively.

Table S2: Estimated affinity values for docked compounds.

Name	Estimated affinity [nM]
Compound **1**	69.7
Compound **33**	5613.0
Compound **3**	13266.8
Compound **13**	28423.9
Compound **47**	95799.6
Compound **2**	221990

Results IDP rescue assay against *P. falciparum* 3D7

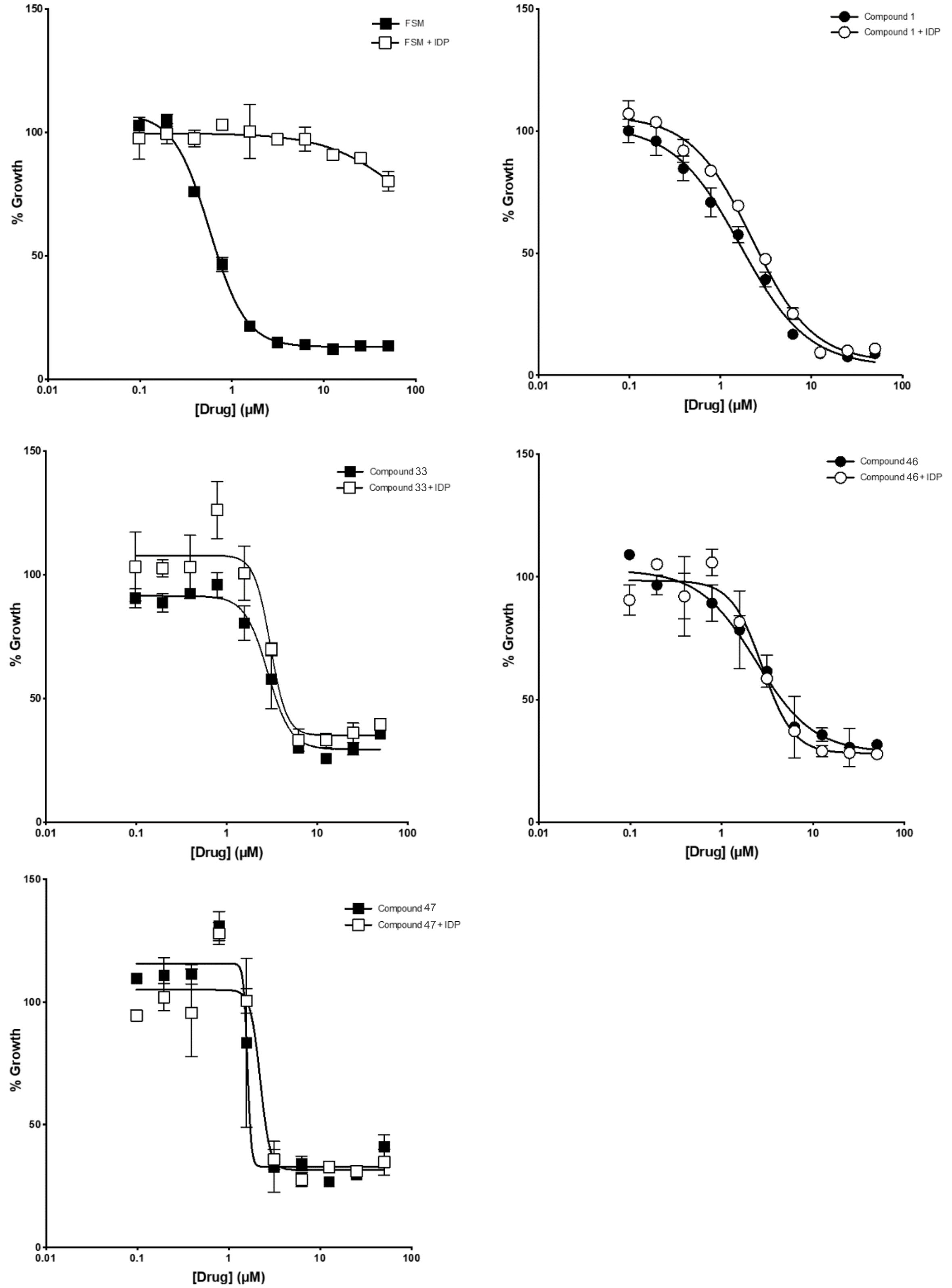

Figure S7. IDP rescue assay: Representative graphs of growth inhibition of *P. falciparum* 3D7 of compounds **1**, **33**, **46**, **47** and fosmidomycin (FSM) as positive control.

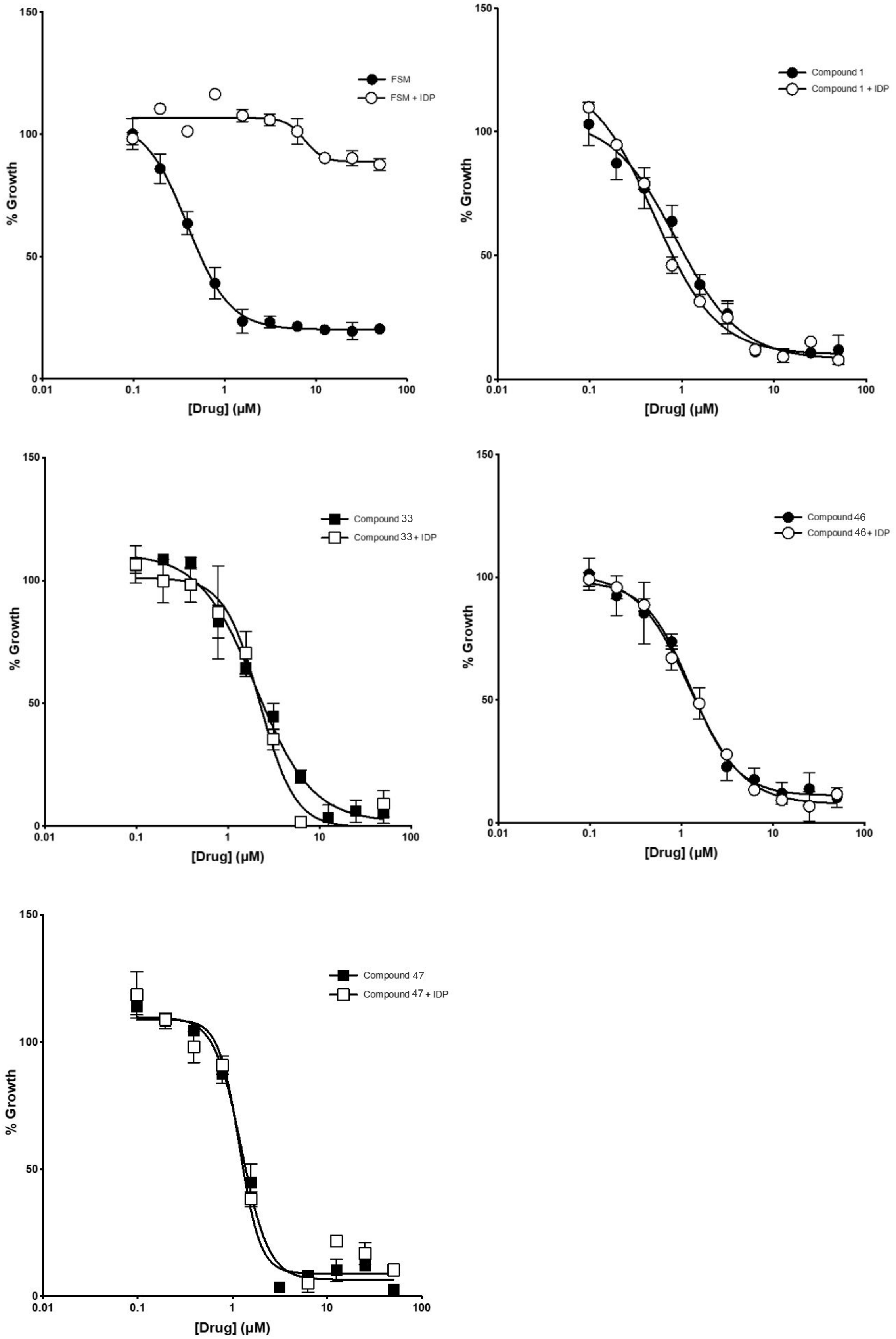

Figure S8. IDP rescue assay: Representative graphs of growth inhibition of *P. falciparum* NF54 of compounds **1**, **33**, **46**, **47** and fosmidomycin (FSM) as positive control.

Table S3. IDP rescue assay: IC_{50} values with and without IDP against *P. falciparum* 3D7 and NF54. SD is displayed as error measure when more than one experiment was performed. a = mean of triplicates, b = mean of duplicates.

**Cmpd**	**IC_{50} 3D7 [µM]**	**IC_{50} NF54 [µM]**
FSM	0.9 ± 0.4[a]	0.9 ± 0.5[b]
FSM + IDP	37.4 ± 18.2[a]	7.8 ± 0.3[b]
**1**	1.9 ± 0.2[b]	0.8 ± 0.1
**1** + IDP	2.4 ± 0.2[b]	0.5
**33**	2.7 ± 0.3	2.4 ± 0.6
**33** + IDP	3.1	2.2
**46**	2.3 ± 0.4	2.1 ± 0.8
**46** + IDP	2.8	1.2
**47**	2.0 ± 0.7	1.6 ± 0.3
**47** + IDP	2.2	1.2

Results LC-MS assay

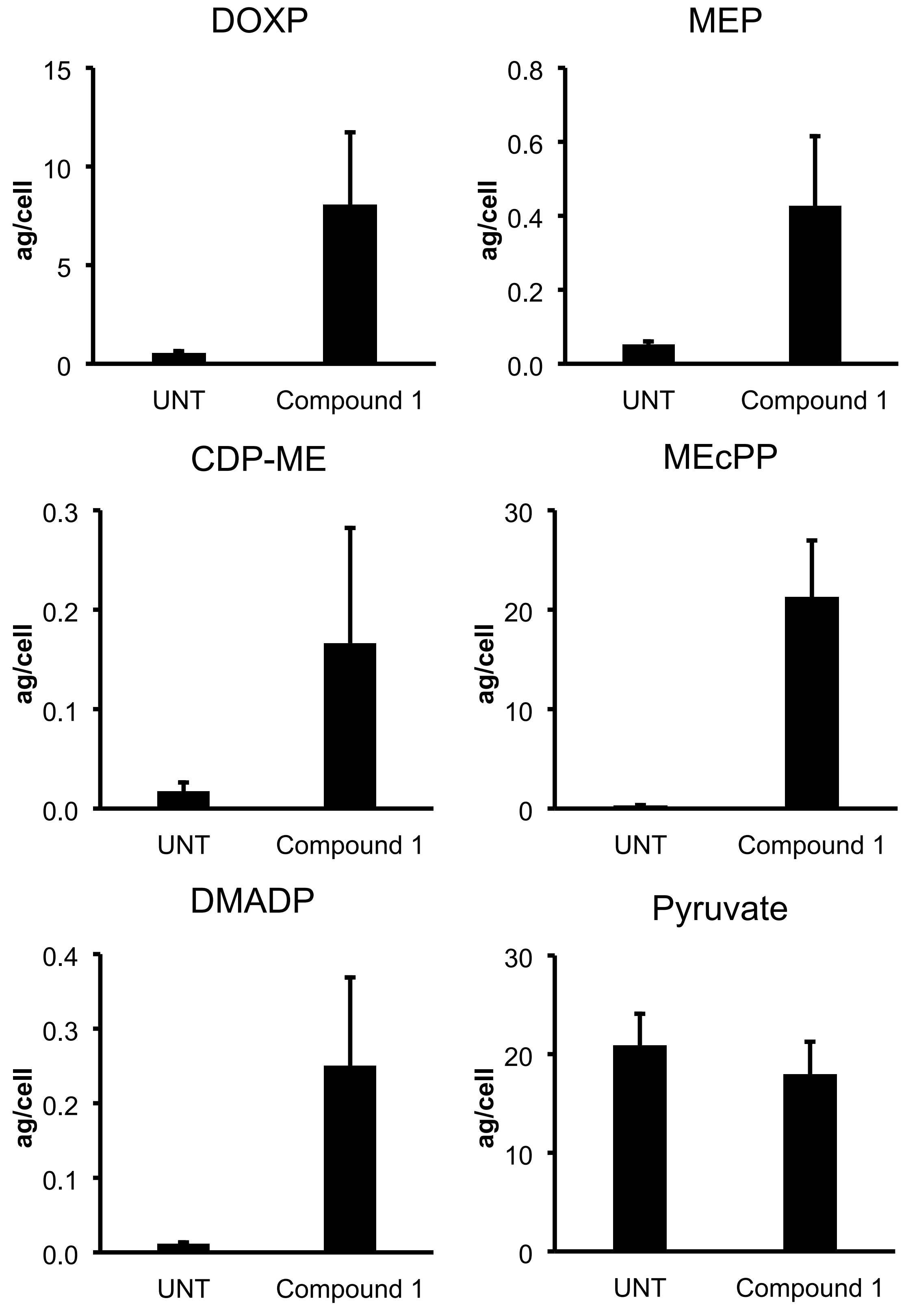

Figure S9. LC-MS data of MEP pathway metabolites in *Escherichia coli* after treatment with compound **1** at ten times the IC_{50}. Pyr = pyruvate, DOXP = 1-deoxy-D-xylulose-5-phosphate, MEP = methylerythritol phosphate, CDP-ME = 4-

diphosphocytidyl-2-*C*-methyl-D-erythritol, MEcDP = methylerythritol cyclodiphosphate, DMADP = dimethylallyl diphosphate, UNT = untreated.

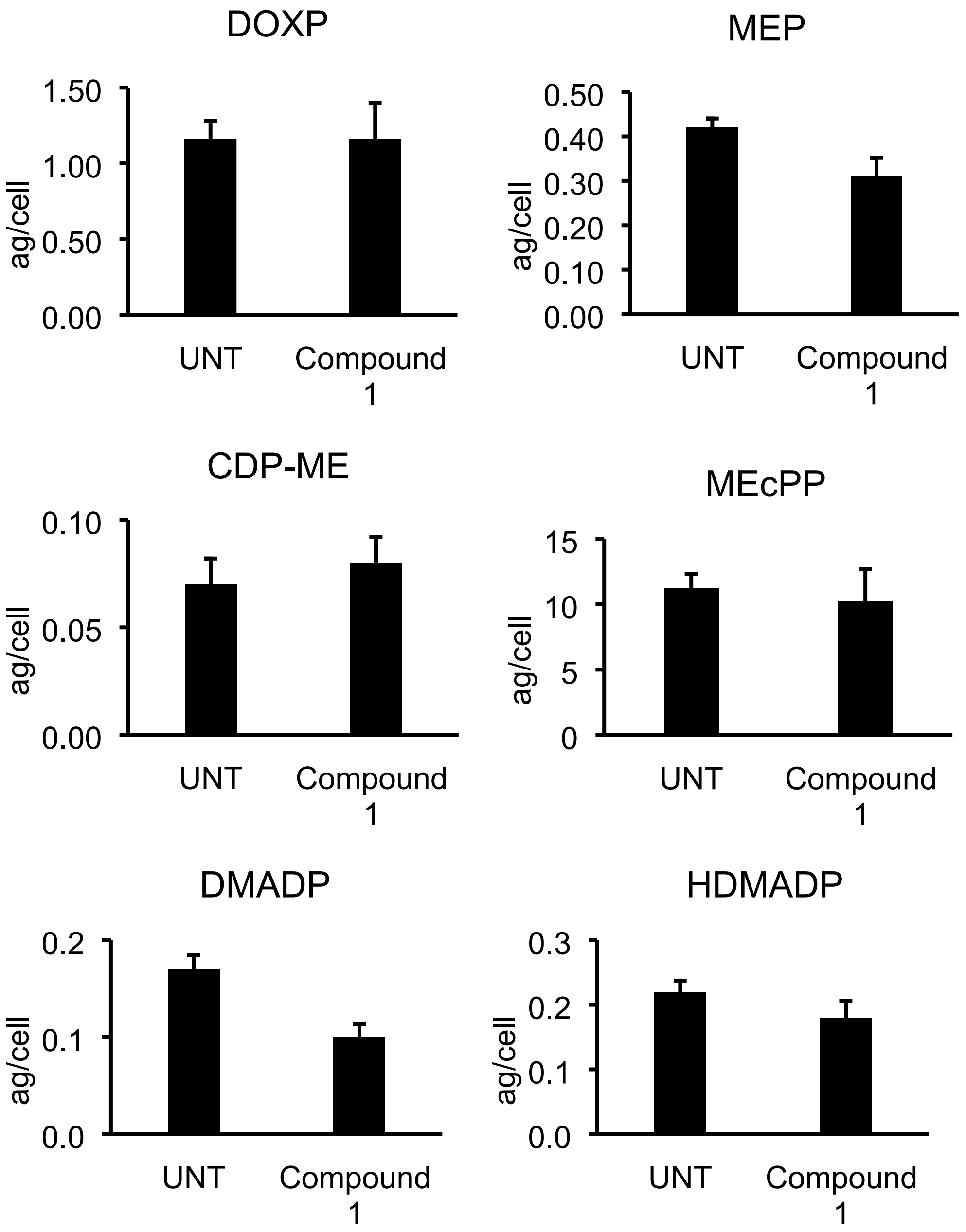

Figure S10. LC-MS data of MEP pathway metabolites in *Plasmodium falciparum* with and without treatment with compound **1** at five times the IC_{50}. Pyr = pyruvate, DOXP = 1-deoxy-D-xylulose-5-phosphate, MEP = methylerythritol phosphate, CDP-ME = 4-diphosphocytidyl-2-C-methyl-D-erythritol, MEcDP = methylerythritol cyclodiphosphate, DMADP = dimethylallyl diphosphate, HDMADP = 1-hydroxy-2-methyl-2-buten-4-yl 4-diphosphate, UNT = untreated.

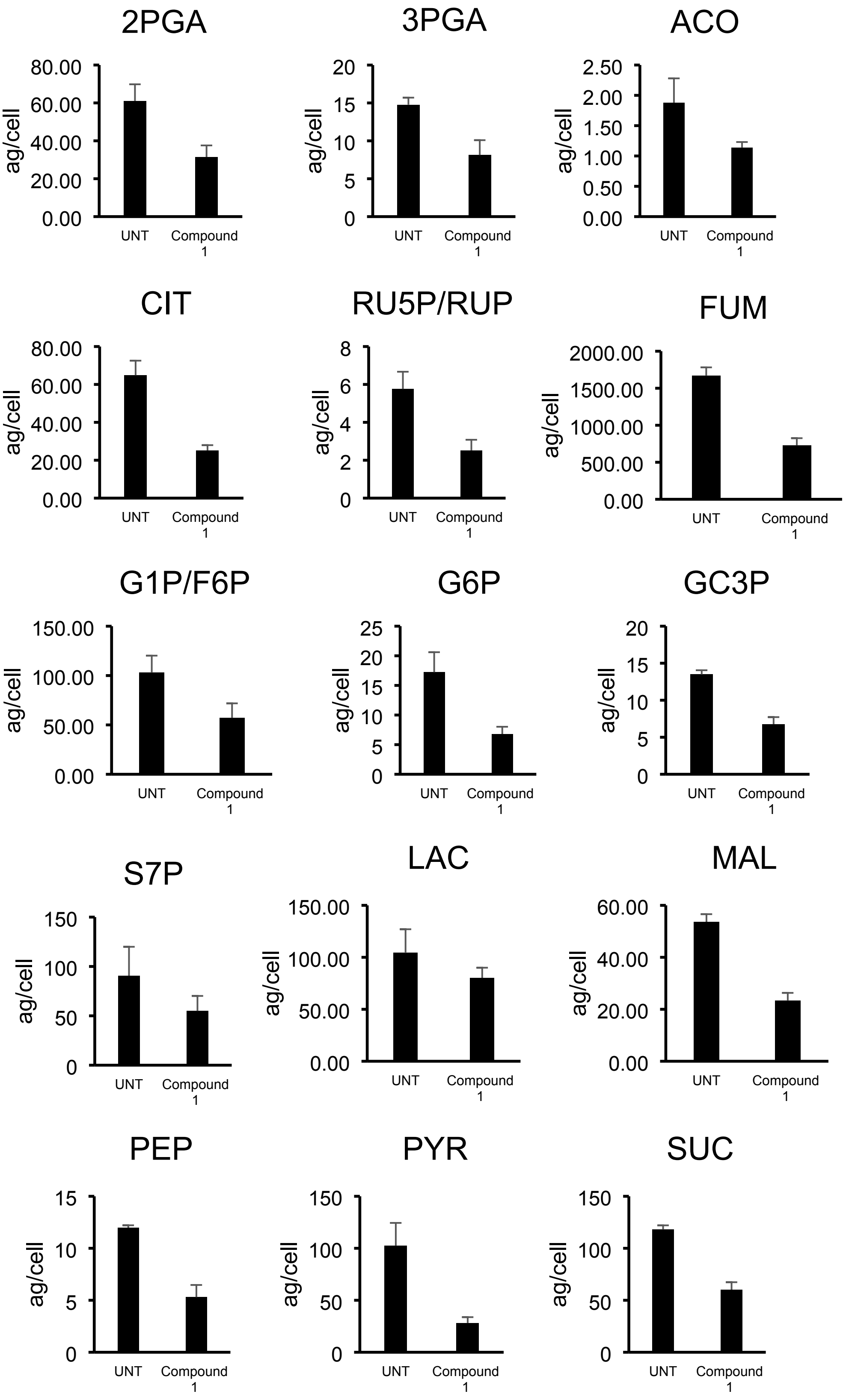

Figure S11. LC-MS analysis of TCA cycle, glycolytic and pentose phosphate pathway metabolites in *Plasmodium falciparum* with and without treatment of compound **1**. 2PGA = 2-phospho glyceric acid, 3PGA = 3-phospho glyceric acid, G1P = glucose-1-phosphate, F6P = fructose-6-phosphate, G6P = glucose-6-phosphate, R5P = ribose-

5-phosphate, Ru5P = ribulose-5-phosphate, PEP = phosphoenolpyruvate, GC3P = glycerol-3-phosphate, S7P = sedoheptulose-7-phosphate, ACO = aconitate, LAC = lactate, SUC = succinate, FUM = fumarate, MAL = malate, CIT = citrate, PYR = pyruvate, UNT = untreated.

Table S4. T-test analysis of LC-MS data for MEP pathway and TCA cycle metabolites. UNT = untreated, DOXP = 1-deoxy-D-xylulose-5-phosphate, MEP = methylerythritol phosphate, CDP-ME = 4-diphosphocytidyl-2-C-methyl-D-erythritol, MEcDP = methylerythritol cyclodiphosphate, DMADP = dimethylallyl diphosphate, HDMADP = 1-hydroxy-2-methyl-2-buten-4-yl 4-diphosphate, 2PGA = 2-phospho glyceric acid, 3PGA = 3-phospho glyceric acid, ACO = aconitate, CIT = citrate, FUM = fumarate, G1P = glucose-1-phosphate, F6P = fructose-6-phosphate, G6P = glucose-6-phosphate, GC3P = glycerol-3-phosphate, LAC = lactate, MAL = malate, PEP = phosphoenolpyruvate, PYR = pyruvate, R5P = ribose-5-phosphate, Ru5P = ribulose-5-phosphate, S7P = Sedoheptulose-7-phosphate, SUC = succinate.

Cmpd	Discovery? P value	Mean of UNT	Mean of + 1	Diff.	SE of diff.
**DOXP**	No >0,99999	1,163	1,163	0,000	0,2689
**MEP**	No 0,070645	0,4233	0,3100	0,1133	0,04631
**CDP-ME**	No 0,587914	0,06667	0,07667	-0,010	0,01700
**MEcDP**	No 0,720090	11,24	10,20	1,043	2,713
**DMADP**	No 0,032540	0,1667	0,1033	0,0633	0,01972
**HDMADP**	No 0,306022	0,2200	0,1833	0,0367	0,03127
**2PGA**	No 0,050194	61,07	31,49	29,58	10,67
**3PGA**	No 0,037010	14,78	8,127	6,657	2,163
**ACO**	No 0,144422	1,877	1,140	0,7367	0,4068
**CIT**	No 0,008553	64,80	25,15	39,65	8,234
**FUM**	No 0,003295	1668	727,7	940,6	149,9
**G1P/F6P**	No 0,112890	103,0	57,13	45,85	22,64
**G6P**	No 0,044056	17,23	6,793	10,43	3,596
**GC3P**	No 0,003896	13,48	6,757	6,720	1,121
**LAC**	No 0,388380	104,2	80,23	23,97	24,80
**MAL**	No 0,001940	53,67	23,30	30,37	4,200
**PEP**	No 0,005029	11,98	5,297	6,687	1,196
**PYR**	No 0,030535	102,5	28,19	74,26	22,65
**RU5P/RUP**	No 0,038874	5,757	2,520	3,237	1,069
**S7P**	No 0,340281	90,64	54,93	35,71	33,01
**SUC**	No 0,002161	118,0	60,23	57,74	8,218

Results of IC_{50} determination against *Pf*DXPS and *Pf*TPK overexpressing *P. falciparum* 3D7

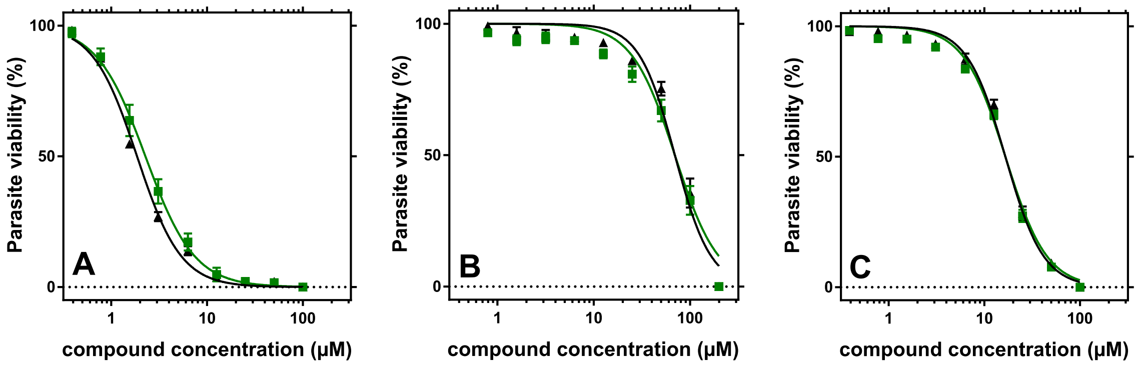

Figure S12. Target verification using *Pf*DXPS overexpressing parasites (3D7-DXPS⁺). Antiplasmodial activity of compounds (**A**: compound **1**, **B**: compound **2**, **C**: compound **3**) against 3D7-DXPS⁺ overexpressing parasites (green squares) in comparison to the MOCK cell line (black triangles). All data were averaged from three independent experiments conducted in triplicate and is shown including SEM (error bars). For IC_{50} determination, data was analyzed using nonlinear regression of the log-concentration-response curves and interpolated from the sigmoidal curve.

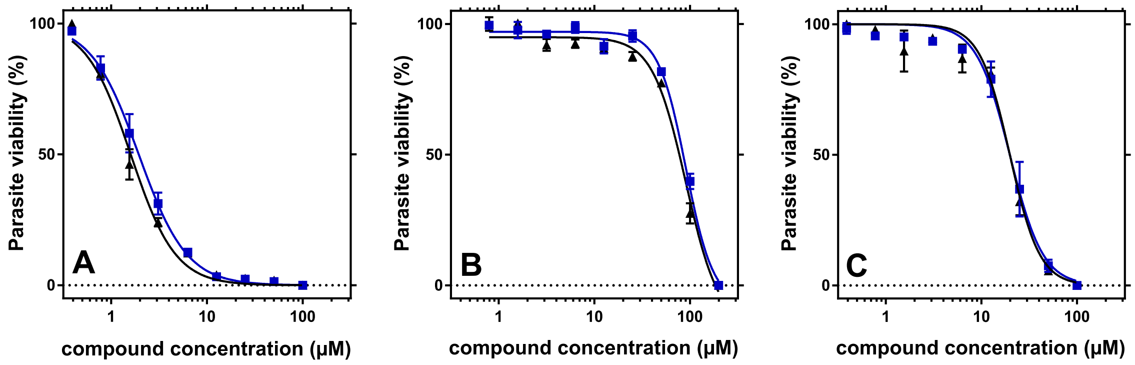

Figure S13. Target verification using *Pf*TPK overexpressing parasites (3D7-DXPS⁺). Antiplasmodial activity of compounds (**A**: compound **1**, **B**: compound **2**, **C**: compound **3**) against *Pf*TPK overexpressing parasites (blue squares) in comparison to the MOCK cell line (black triangles). Data represent the result from one experiment with technical triplicates (error bars indicate SEM). For IC_{50} determination, data was analyzed using nonlinear regression of the log-concentration-response curves and interpolated from the sigmoidal curve.

Table S5. Compound evaluation against *P. falciparum* 3D7-TPK⁺ and *P. falciparum* 3D7-DXPS⁺ in comparison to MOCK cell line. The table displays the IC_{50} values from antiplasmodial screens using *P. falciparum* 3D7-*Pf*TPK$^+$ (3D7-TPK$^+$) and *Plasmodium falciparum* 3D7-*Pf*DXPS (3D7-DXPS$^+$) overexpression cell lines, as interpolated from nonlinear regression curves. 95% CI is displayed as error measure. Percentages indicate statistical probability of the simpler model "LogIC_{50} same for all data sets" being correct.

Cmpd	*Pf*DXPS IC_{50} (µM)	MOCK IC_{50} (µM)	Difference (yes/no)	*Pf*TPK[a] IC_{50} (µM)	MOCK[a] IC_{50} (µM)	Difference (yes/no)
**2**	109.9 (89.7–167.6)	109.9 (89.7–167.6)	78.49%	90.4 (78.6–116.9)	90.4 (78.6–116.9)	No 91.69%
**3**	17.9 (17.1–18.8)	17.9 (17.1–18.8)	77.18%	21.1 (19.4–23.1)	23.8	No 83.76%
**1**	2.08 (1.66–2.48)	1.61 (1.45–1.76	Yes 15.25%	1.76 (1.36–2.1)	1.18 (0.87–1.41)	Yes 7.50%

[a]Single measurement.

Inhibition data for all compounds

Table S6. Summary of determined IC_{50} values of all compounds. The numbers I-III are indicating the used method.

ID	Structure	IC_{50} 3D7-III [µM]	IC_{50} Dd2-III [µM]	IC_{50} 3D7-II [µM]	IC_{50} NF54-II [µM]	IC_{50} 3D7-I [µM]
	FSM	n.d.	n.d.	0.9 ± 0.2	0.7 ± 0.3	n.d.
	Chloroquine	2.8 ± 0.7 nM	0.19 ± 0.03	n.d.	n.d.	17.8 ± 3.1 nM
**2**	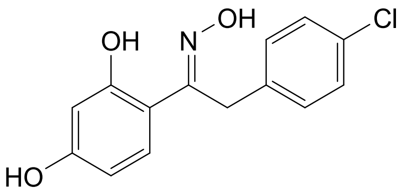	94 ± 8	89 ± 31	42 ± 4	41 ± 10	81 ± 3
**4**	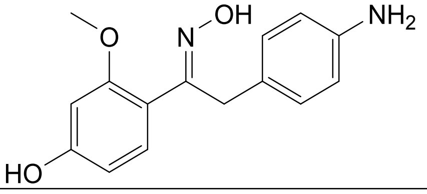	>111	94 ± 16			

ID	Structure	IC_{50} 3D7-III [µM]	IC_{50} Dd2-III [µM]	IC_{50} 3D7-II [µM]	IC_{50} NF54-II [µM]	IC_{50} 3D7-I [µM]
**5**	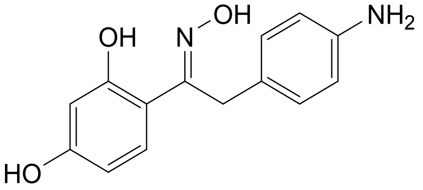	>111	>111			
**6**	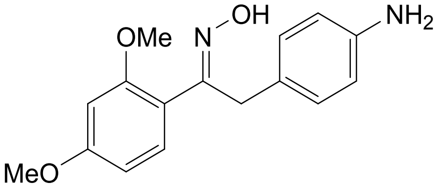	>111	>111			
**7Z**	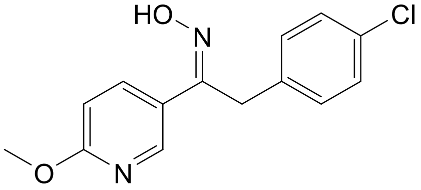	>111	>111			
**8E**	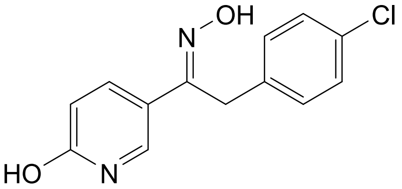	>111	>111			
**8Z**	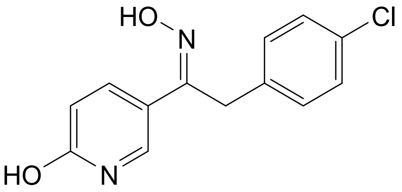	>28	>28			
**9**	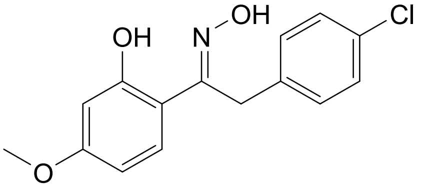	47 ± 15	24 ± 6			
**7E**	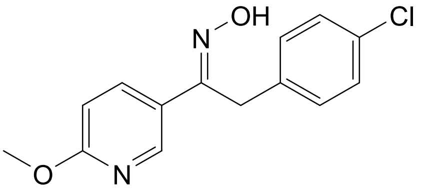	44 ± 10	>55			
**10**	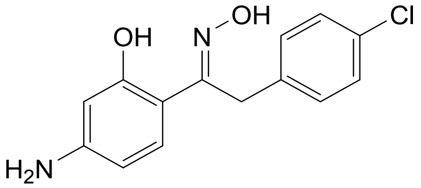	38 ± 11	24 ± 4			

ID	Structure	IC_{50} 3D7-III [µM]	IC_{50} Dd2-III [µM]	IC_{50} 3D7-II [µM]	IC_{50} NF54-II [µM]	IC_{50} 3D7-I [µM]
**11**	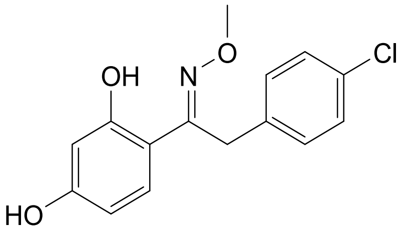	28 ± 9	19 ± 3			
**12**	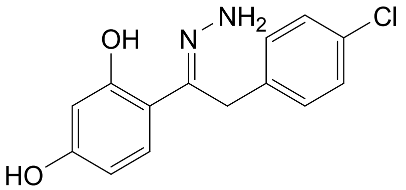	16 ± 0	14 ± 5	46 ± 3	45 ± 7	
**13**	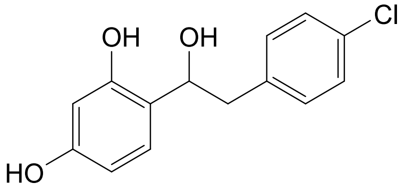	10 ± 2	28 ± 13			
**3**	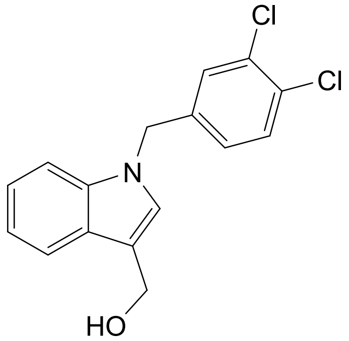	24 ± 6	13 ± 9	17 ± 2	15 ± 2	25 ± 3
**14**	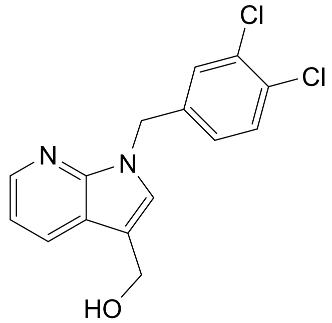	> 111	91 ± 18			
**15**	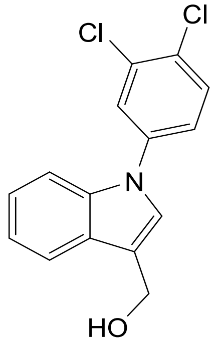	> 56	54 ± 6			

ID	Structure	IC_{50} 3D7-III [µM]	IC_{50} Dd2-III [µM]	IC_{50} 3D7-II [µM]	IC_{50} NF54-II [µM]	IC_{50} 3D7-I [µM]
**16**	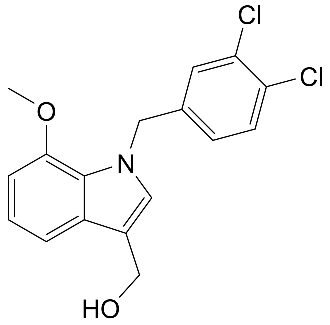	> 56	48 ± 13			
**17**	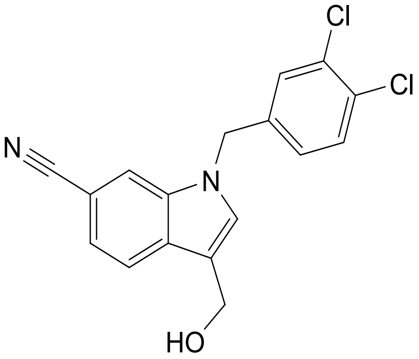	44 ± 19	44 ± 9			
**18**	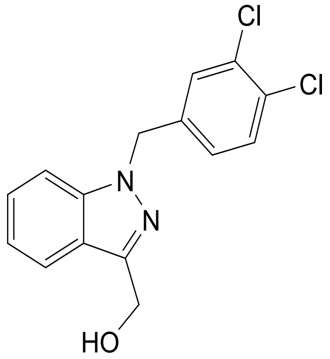	34 ± 0	70 ± 21			
**19**	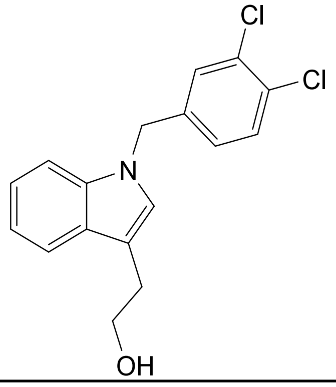	29 ± 1	12 ± 3			

ID	Structure	IC_{50} 3D7-III [μM]	IC_{50} Dd2-III [μM]	IC_{50} 3D7-II [μM]	IC_{50} NF54-II [μM]	IC_{50} 3D7-I [μM]
**20**	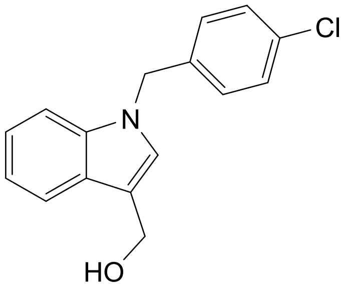	28 ± 14	22 ± 5			
**21**	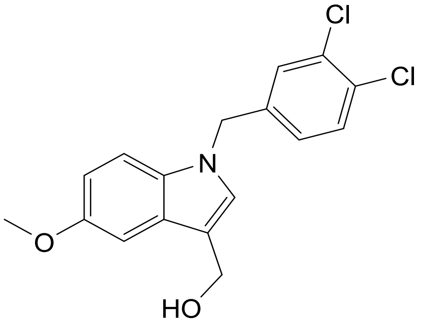	19 ± 1	54 ± 6			
**22**	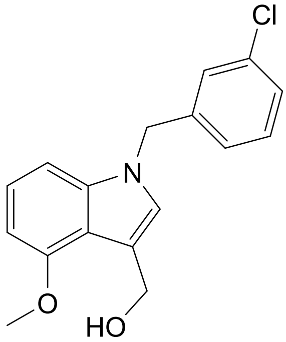	16 ± 3	14 ± 4			
**23**	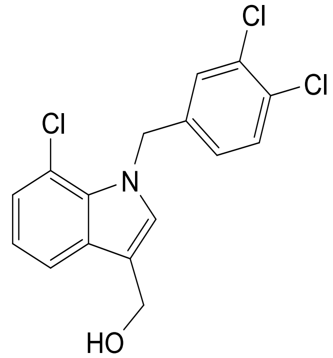	14 ± 1	36 ± 16			
**24**	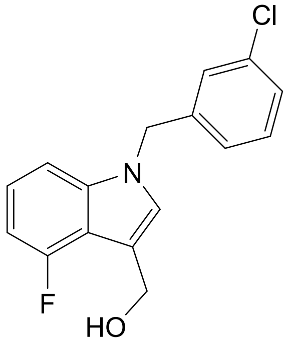	12 ± 1	10 ± 1			

ID	Structure	IC_{50} 3D7-III [µM]	IC_{50} Dd2-III [µM]	IC_{50} 3D7-II [µM]	IC_{50} NF54-II [µM]	IC_{50} 3D7-I [µM]
**25**	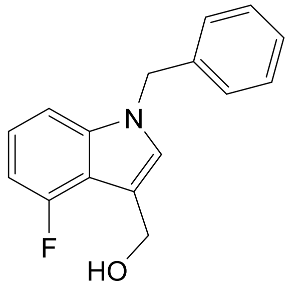	12 ± 2	9 ± 0			
**26**	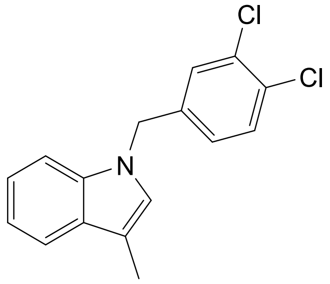	10 ± 3	11 ± 5			
**27**	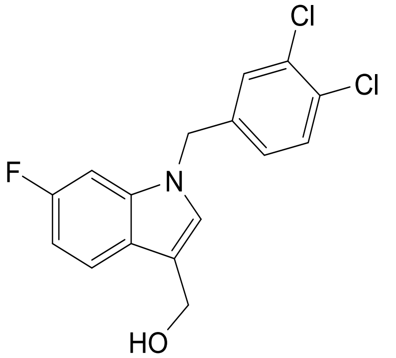	9 ± 2	10 ± 5			
**28**	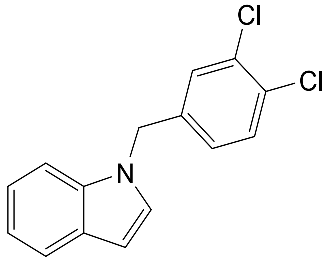	8 ± 0	18 ± 7			
**29**	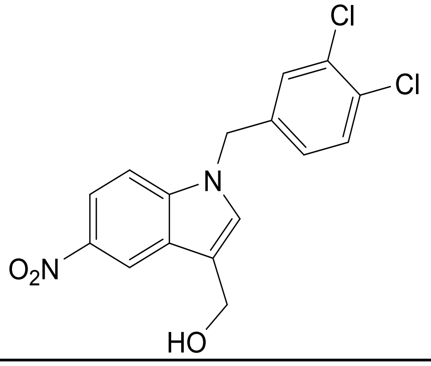	7 ± 0	4 ± 1			

ID	Structure	IC_{50} 3D7-III [µM]	IC_{50} Dd2-III [µM]	IC_{50} 3D7-II [µM]	IC_{50} NF54-II [µM]	IC_{50} 3D7-I [µM]
**30**	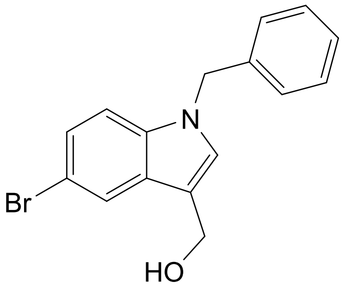	6 ± 1	5 ± 3			
**31**	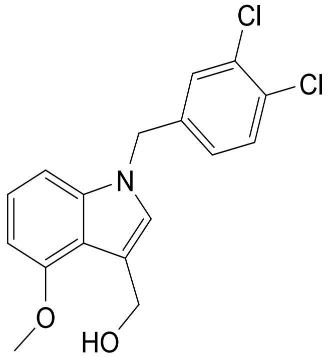	6 ± 2	5 ± 2			
**32**	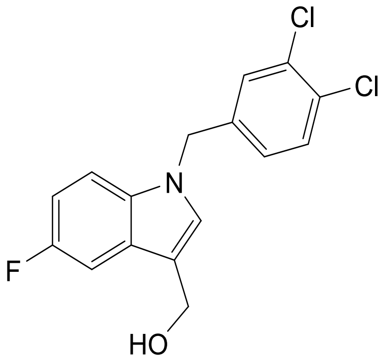	5 ± 1	8 ± 2			
**33**	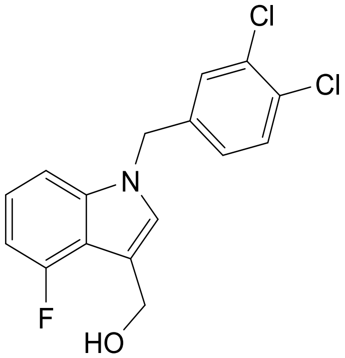	2 ± 0	3 ± 0	3 ± 0	3 ± 1	

ID	Structure	IC_{50} 3D7-III [μM]	IC_{50} Dd2-III [μM]	IC_{50} 3D7-II [μM]	IC_{50} NF54-II [μM]	IC_{50} 3D7-I [μM]
**34**	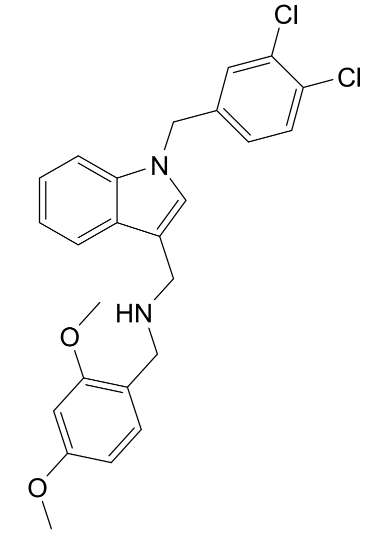	47 ± 15	21 ± 6			
**1**	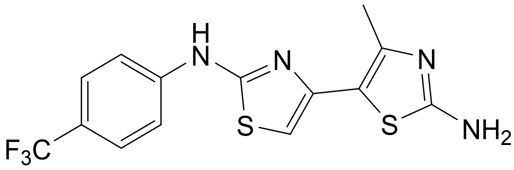	0.6 ± 0.2	0.8 ± 0.4	1.7 ± 0.6	0.8 ± 0.1	1.6 ± 0.6
**35**	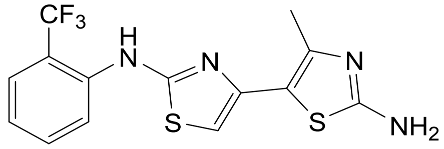	43 ± 2	48 ± 12			
**36**	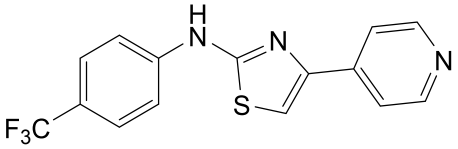	34 ± 2	25 ± 2			
**37**	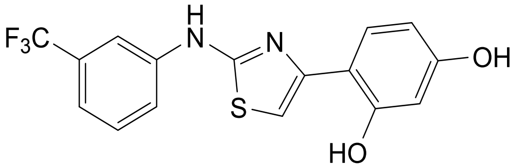	23 ± 1	19 ± 2			
**38**	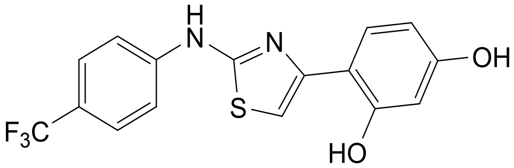	22 ± 6	25 ± 5			
**39**	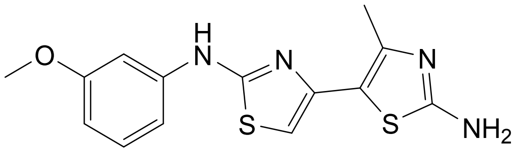	18 ± 0	43 ± 24			

ID	Structure	IC_{50} 3D7-III [µM]	IC_{50} Dd2-III [µM]	IC_{50} 3D7-II [µM]	IC_{50} NF54-II [µM]	IC_{50} 3D7-I [µM]
**40**	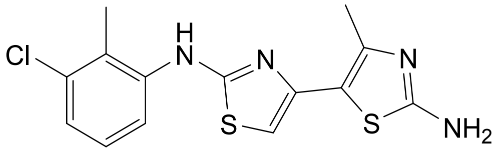	16 ± 2	17 ± 10			
**41**	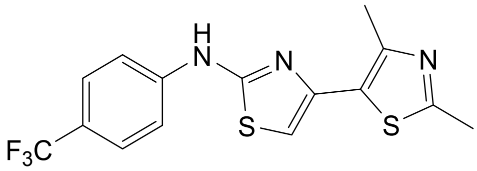	10 ± 1	5 ± 0			
**42**	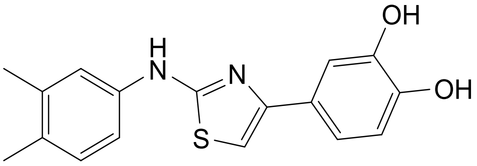	4 ± 1	7 ± 1			
**43**	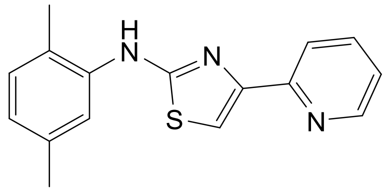	4 ± 2	5 ± 1			
**44**	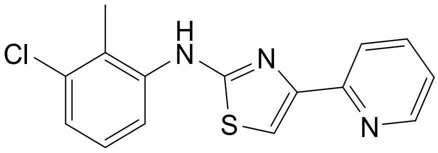	3 ± 0	2 ± 1			
**45**	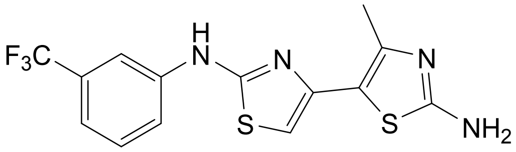	3 ± 0	5 ± 0			
**46**	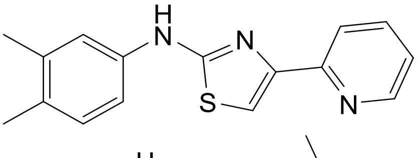	2 ± 1	2 ± 0	2 ± 0	2 ± 1	
**47**	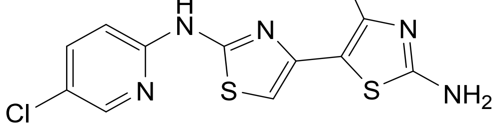	1 ± 0	2 ± 0	2 ± 1	2 ± 0	

PPB results for the Oxime-class

Note: References in this section refer to the main manuscript.

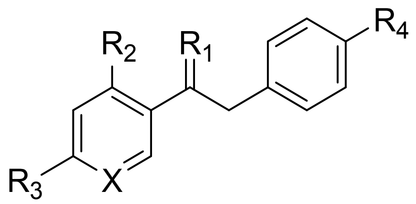

Oxime core structure

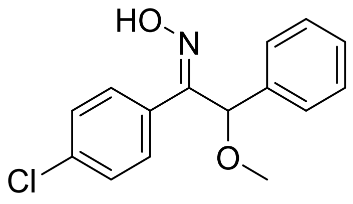

CHEMBL581471

Figure S14. The oxime scaffold and the related compound **CHEMBL581471**. For elucidation of X and the R_1–R_4 groups, please see **Error! Reference source not found.**.

Similar to our core structure, we found the related oxime **CHEMBL581471** (**Figure S14**). This compound was identified in 2008 by Novartis in a high-throughput screening (HTS) and showed activity against *P. falciparum* in a liver-stage assay. The oxime was a hit in the initial HTS, but could not be confirmed and was therefore not evaluated further. The reported EC_{50} values are >0.912 µM in 3D7 and >1.607 µM in W2. The compound also showed good cytotoxicity behavior with >10 µM Huh7 (human hepatoma cells) inhibition, about 10 fold higher than the EC_{50}.[(21)]

Although no molecular target is assigned to compound **CHEMBL581471**, the finding gives us additional insights for the design of further derivatives. It seems that the CH_2 of the linker might be a possible growth vector. Together with the replacement of the oxime functionality with an amino or hydroxyl-group, as suggested by our SAR study, the initial oxime hit offers the potential to be developed into a different class in the future.

PPB results for the Indole-class

Note: References in this section refer to the main manuscript.

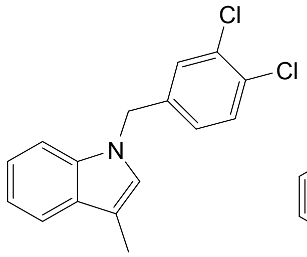

26
IC_{50} *P.f.* = 10.2 µM

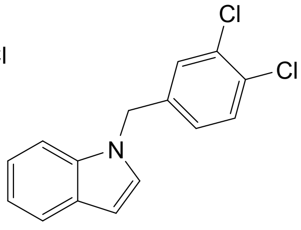

28
IC_{50} *P.f.* = 8.1 µM

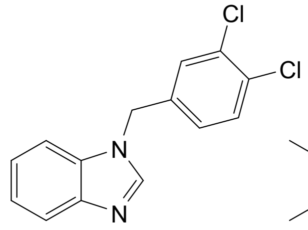

CHEMBL570782
IC_{50} FabI = 4.7 µM

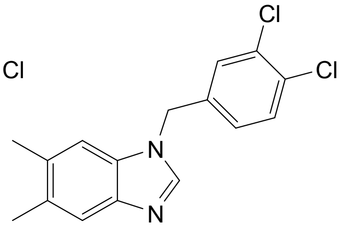

CHEMBL1945694
IC_{50} FabI = 0.3 µM

Figure S15. Compounds **26** and **28** (IC_{50} cell-based using *Plasmodium falciparum* 3D7, Method III) shown with the related compounds inhibiting the Enoyl-[acyl-carrier-protein] reductase (FabI) (IC_{50} based on enzymatic assay).

Our search for compounds **26** and **28** yielded the similar compounds **CHEMBL1945694** and **CHEMBL570782** (**Figure S15**), which are reported to inhibit the Enoyl-[acyl-carrier-protein] reductase (ENR or FabI) from *Francisella tularensis*.[22] The two compounds we found belong to a larger class of benzimidazoles, first identified to inhibit *F. tularensis* FabI in 2012 using a LBVS approach by Johnson and coworkers.[(23)] In follow-up studies, they reported derivatives with improved activity against the FabI enzyme of up to 14 nM and determined the crystal structure of FabI from *F. tularensis* in complex with **CHEMBL1945694**. [(24,25)]

The FabI enzyme is part of the fatty acid biosynthesis pathway-II (FAS-II) found in a variety of microorganisms, including *P. falciparum.* The mammalian counterpart FAS-I consists of one protein complex with low homology to the FAS-II enzymes.[(27–29)] The FabI enzyme catalyzes the final reduction step, suppling fatty acids for cell-wall biosynthesis. Enzymes of the pathway are used for the development of new antimicrobial agents.[(30–33)] It was, however, shown that the blood stage of *P. falciparum* does not require the FAS-II pathway for proliferation.[(34–36)] Therefore, inhibition of FabI cannot explain the activity we observed in the blood stage assays. However, it could be beneficial for a new anti-malarial drug to inhibit FabI as a second target, as FabI is essential for liver stage proliferation and therefore transmission.[(36)]

Taken together, the structural similarity of the compounds hints to FabI being an additional target of our hit classes. In particular, the indole-class is very likely to bind to FabI, as there are co-crystal structures of related compounds. Inhibition of FabI does not explain the observed effects in culture in our assays, as this pathway is only important during the liver stage of *P. falciparum* growth.[(28)]

Human off-target enzymes

Table S7. Summary of possible human off-target enzymes encountered during the search for bacterial targets. The molecule drawn in “search hit” is reported in the cited source to be targeting the enzyme or pathway given in “target enzyme”. Only one hit molecule is shown, the cited source often reports many more derivatives. No ranking and in-depth analysis of likelihood to be a target was performed.

Search molecule	Search hit	Target enzyme(s)	Reference
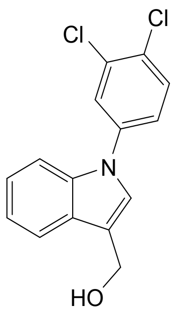	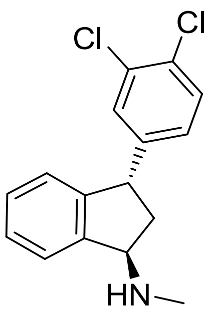<br>Indatraline	non-selective monoamine transporter inhibitor	6,7

Search molecule	Search hit	Target enzyme(s)	Reference
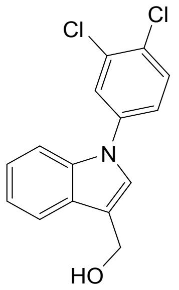	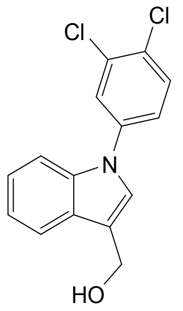 Oncrasin-1	RNA polymerase II	[8, 9]
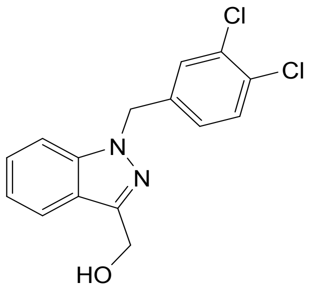	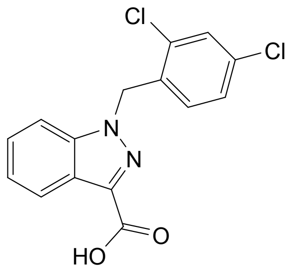 Lonidamine	Inhibitor of aerobic glycoloysis in cancer cells, in particular hexokinase	[10,11]
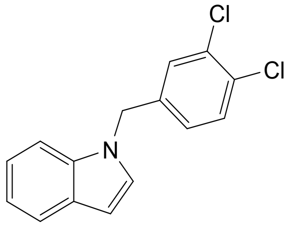	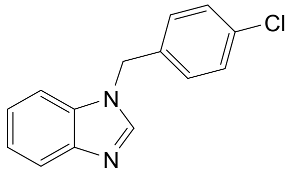	cortisol biosynthesis, CYP11B1	[12]
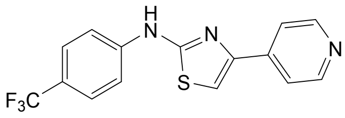	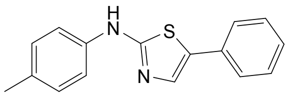	KDR kinase	[13]
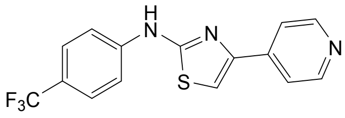	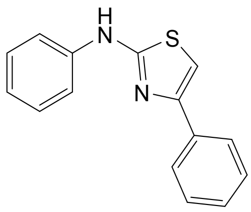	Eicosanoid metabolism	[14]

Search molecule	Search hit	Target enzyme(s)	Reference
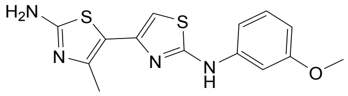	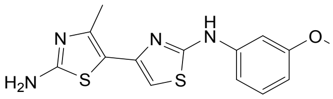	Several human targets reported	[15]
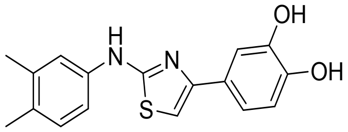	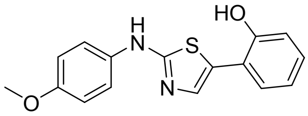	α-amylase	[16]

Table of compounds as SMILES with biological activity and PAINS count.

Structure	ID	MW	IC_{50} [µM]	IC_{50} [µM] StdDev	PAINS count*
c1ccc2c(c1)c(cn2Cc3ccc(c(c3)Cl)Cl)CO	**3**	306.2	23.6	6.4	0
c1ccc2c(c1)c(cn2Cc3ccc(cc3)Cl)CO	**20**	271.7	27.2	13.6	0
c1cc2c(cn(c2c(c1)Cl)Cc3ccc(c(c3)Cl)Cl)CO	**22**	340.6	13.8	0.9	0
COc1cccc2c1n(cc2CO)Cc3ccc(c(c3)Cl)Cl	**16**	336.2	55.5	0	0
c1ccc2c(c1)ccn2Cc3ccc(c(c3)Cl)Cl	**28**	276.2	8.1	0.1	0
COc1cccc2c1c(cn2Cc3ccc(c(c3)Cl)Cl)CO	**31**	336.2	5.5	1.5	0
Cc1cn(c2c1cccc2)Cc3ccc(c(c3)Cl)Cl	**26**	290.2	10.2	3.4	0
c1cc(c(cc1Cn2cc(c3c2cc(cc3)F)CO)Cl)Cl	**27**	324.2	8.9	2.4	0
c1ccc2c(c1)c(cn2Cc3ccc(c(c3)Cl)Cl)CCO	**19**	320.2	28.9	1.2	0
c1cc(c(cc1Cn2cc(c3c2cc(cc3)C#N)CO)Cl)Cl	**17**	331.2	43.8	19.2	0
c1cc(c(cc1Cn2cc(c3c2ccc(c3)N(=O)=O)CO)Cl)Cl	**29**	351.2	7.2	0.1	0
c1cc(c(cc1Cn2cc(c3c2ccc(c3)F)CO)Cl)Cl	**32**	324.2	5.3	1.3	0
COc1ccc2c(c1)c(cn2Cc3ccc(c(c3)Cl)Cl)CO	**21**	336.2	18.5	1.1	0
c1cc2c(cn(c2nc1)Cc3ccc(c(c3)Cl)Cl)CO	**14**	307.2	>111	0	0
c1ccc(cc1)Cn2cc(c3c2ccc(c3)Br)CO	**30**	316.2	6.1	1.3	0
c1ccc2c(c1)c(cn2c3ccc(c(c3)Cl)Cl)CO	**15**	292.2	55.5	0	0
COc1ccc(c(c1)OC)CNCc2cn(c3c2cccc3)Cc4ccc(c(c4)Cl)Cl	**34**	455.4	0.8	0.2	0
c1cc2c(c(c1)F)c(cn2Cc3ccc(c(c3)Cl)Cl)CO	**33**	324.2	2.4	0.2	0
c1ccc2c(c1)c(nn2Cc3ccc(c(c3)Cl)Cl)CO	**18**	307.2	33.9	0.2	0
c1cc(cc(c1)Cl)Cn2cc(c3c2ccc(c3)F)CO	**24**	289.7	12	0.1	0
COc1ccc2c(c1)c(cn2Cc3ccc(c(c3)Cl)Cl)CO	**22**	336.2	15.6	3.3	0
c1ccc(cc1)Cn2cc(c3c2ccc(c3)F)CO	**25**	255.3	11.9	1.5	0
c1cc(ccc1CC(=NO)c2ccc(cc2O)O)N	**5**	258.3	>111	0	0
COc1ccc(c(c1)OC)/C(=N/O)/Cc2ccc(cc2)N	**6**	286.3	>111	0	0
COc1cc(ccc1C(=NO)Cc2ccc(cc2)N)O	**4**	272.3	>111	0	0
c1cc(ccc1CC(c2ccc(cc2O)O)O)Cl	**13**	264.7	9.9	1.7	0
c1cc(ccc1CC(=NN)c2ccc(cc2O)O)Cl	**12**	276.7	16	0.2	0

Structure	ID	MW	IC_{50} [µM]	IC_{50} [µM] StdDev	PAINS count*
CON=C(Cc1ccc(cc1)Cl)c2ccc(cc2O)O	**11**	291.7	28	8.8	0
c1cc(ccc1CC(=NO)c2ccc(cc2O)N)Cl	**10**	276.7	38.2	11.2	0
COc1ccc(c(c1)O)C(=NO)Cc2ccc(cc2)Cl	**9**	291.7	46.8	1.5	0
c1cc(ccc1CC(=NO)c2ccc(cc2O)O)Cl	**2**	277.7	93.8	0.8	0
c1cc(ccc1C/C(=N\O)/c2ccc(nc2)O)Cl	**8*Z***	262.7	>28	0	0
c1cc(ccc1C/C(=N\O)/c2ccc(nc2)O)Cl	**8*E***	262.7	>111	0	0
COc1ccc(cn1)C(=NO)Cc2ccc(cc2)Cl	**7*Z***	276.7	>111	0	0
COc1ccc(cn1)C(=NO)Cc2ccc(cc2)Cl	**7*E***	276.7	43.6	9.5	0
Cc1c(cccc1Cl)Nc2nc(cs2)c3ccccn3	**44**	301.8	2.8	0.1	0
Cc1c(sc(n1)C)c2csc(n2)Nc3ccc(cc3)C(F)(F)F	**41**	355.4	9.5	1.1	0
Cc1c(sc(n1)N)c2csc(n2)Nc3ccccc3C(F)(F)F	**35**	356.4	43	2.4	0
c1cc(ccc1C(F)(F)F)Nc2nc(cs2)c3ccncc3	**36**	321.3	34.2	1.8	0
c1cc(ccc1C(F)(F)F)Nc2nc(cs2)c3ccc(cc3O)O	**38**	352.3	22.1	5.6	0
c1cc(cc(c1)Nc2nc(cs2)c3ccc(cc3O)O)C(F)(F)F	**37**	352.3	23	0.7	0
Cc1c(sc(n1)N)c2csc(n2)Nc3cccc(c3)OC	**39**	318.4	17.9	0.4	0
Cc1c(cccc1Cl)Nc2nc(cs2)c3c(nc(s3)N)C	**40**	336.9	15.9	1.8	0
Cc1ccc(cc1C)Nc2nc(cs2)c3ccc(c(c3)O)O	**42**	312.4	4.4	0.1	0
c1ccc(cc1)Nc2nc(cs2)c3ccccn3	**43**	253.3	3.7	1.6	0
Cc1c(sc(n1)N)c2csc(n2)Nc3cccc(c3)C(F)(F)F	**45**	356.4	2.5	0.1	0
Cc1ccc(cc1C)Nc2nc(cs2)c3ccccn3	**46**	281.4	1.5	0.8	0
Cc1c(sc(n1)N)c2csc(n2)Nc3ccc(cn3)Cl	**47**	323.8	1	0.1	0
Cc1c(sc(n1)N)c2csc(n2)Nc3ccc(cc3)C(F)(F)F	**1**	356.4	0.6	0.2	0

*Compounds were checked for PAINS motives using the PAINS filter of the software StarDrop, which searches for functional groups defined in the publication by J.Baell and G. Holloway.(17)

References

(1) Di, Z.; Johannsen, S.; Masini, T.; Simonin, C.; Haupenthal, J.; Andreas, A.; Awale, M.; Gierse, R. M.; van der Laan, T.; van der Vlag, R.; Nasti, R.; Poizat, M.; Buhler, E.; Reiling, N.; Müller, R.; Fischer, M.; Reymond, J.-L.; Hirsch, A. K. H. Discovery of novel drug-like antitubercular hits targeting the MEP pathway enzyme DXPS by strategic application of ligand-based virtual screening. *Chem. Sci.* **2022**.

(2) Ivanov, I.; Nikolova, S.; Statkova-Abeghe, S. Efficient one-pot Friedel–Crafts Acylation of benzene and its derivatives with unprotected aminocarboxylic acids in polyphosphoric acid. *Synth. Commun.* **2006**, *36* (10), 1405–1411.

(3) Rarey, M.; Kramer, B.; Lengauer, T.; Klebe, G. A fast flexible docking method using an incremental construction algorithm. *J. Mol. Biol.* **1996**, *261* (3), 470–489.

(4) Schneider, N.; Lange, G.; Hindle, S.; Klein, R.; Rarey, M. A consistent description of HYdrogen bond and DEhydration energies in protein-ligand complexes: methods behind the HYDE scoring function. *J. Comput. Aided Mol. Des.* **2013**, *27* (1), 15–29.

(5) Reulecke, I.; Lange, G.; Albrecht, J.; Klein, R.; Rarey, M. Towards an integrated description of hydrogen bonding and dehydration: decreasing false positives in virtual screening with the HYDE scoring function. *ChemMedChem* **2008**, *3* (6), 885–897.

(6) Bøgesø, K. P.; Christensen, A. V.; Hyttel, J.; Liljefors, T. 3-Phenyl-1-indanamines. Potential antidepressant activity and potent inhibition of dopamine, norepinephrine, and serotonin uptake. *J. Med. Chem.* **1985**, *28* (12), 1817–1828.

(7) EMBL-EBI. *CHEMBL296602*, **accessed 2021**. https://www.ebi.ac.uk/chembl/compound_report_card/CHEMBL296602/.

(8) Mehboob, S.; Hevener, K. E.; Truong, K.; Boci, T.; Santarsiero, B. D.; Johnson, M. E. Structural and enzymatic analyses reveal the binding mode of a novel series of Francisella tularensis enoyl reductase (FabI) inhibitors. *J. Med. Chem.* **2012**, *55* (12), 5933–5941.

(9) Wu, S.; Wang, L.; Guo, W.; Liu, X.; Liu, J.; Wei, X.; Fang, B. Analogues and derivatives of oncrasin-1, a novel inhibitor of the C-terminal domain of RNA polymerase II and their antitumor activities. *J. Med. Chem.* **2011**, *54* (8), 2668–2679.

(10) EMBL-EBI. *CHEMBL1257030*, **accessed 2021**. https://www.ebi.ac.uk/chembl/compound_report_card/CHEMBL1257030/.

(11) Floridi, A.; Paggi, M. G.; Marcante, M. L.; Silvestrini, B.; Caputo, A.; Martino, C. de. Lonidamine, a selective Iinhibitor of aerobic glycolysis of murine tumor cells. *J. Natl. Cancer Inst.* **1981**.

(12) Hille, U. E.; Zimmer, C.; Vock, C. A.; Hartmann, R. W. First selective CYP11B1 inhibitors for the treatment of cortisol-dependent diseases. *ACS Med. Chem. Lett.* **2011**, *2* (1), 2–6.

(13) Bilodeau, M. T.; Rodman, L. D.; McGaughey, G. B.; Coll, K. E.; Koester, T. J.; Hoffman, W. F.; Hungate, R. W.; Kendall, R. L.; McFall, R. C.; Rickert, K. W.; Rutledge, R. Z.; Thomas, K. A. The discovery of N-(1,3-thiazol-2-yl)pyridin-2-amines as potent inhibitors of KDR kinase. *Bioorg. Med. Chem. Lett.* **2004**, *14* (11), 2941–2945.

(14) Rödl, C. B.; Vogt, D.; Kretschmer, S. B. M.; Ihlefeld, K.; Barzen, S.; Brüggerhoff, A.; Achenbach, J.; Proschak, E.; Steinhilber, D.; Stark, H.; Hofmann, B. Multi-dimensional target profiling of N,4-diaryl-1,3-thiazole-2-amines as potent inhibitors of eicosanoid metabolism. *Eur. J. Med. Chem.* **2014**, *84*, 302–311.

(15) EMBL-EBI. *CHEMBL1384007*, **accessed 2021**. https://www.ebi.ac.uk/chembl/compound_report_card/CHEMBL1384007/.

(16) Al-Asri, J.; Fazekas, E.; Lehoczki, G.; Perdih, A.; Görick, C.; Melzig, M. F.; Gyémánt, G.; Wolber, G.; Mortier, J. From carbohydrates to drug-like fragments: Rational development of novel α-amylase inhibitors. *Bioorg. Med. Chem.* **2015**, *23* (20), 6725–6732.

(17) Baell, J. B.; Holloway, G. A. New substructure filters for removal of pan assay interference compounds (PAINS) from screening libraries and for their exclusion in bioassays. *J. Med. Chem.* **2010**, *53* (7), 2719–2740.
