# Supplementary material for: High Target Homology Does Not Guarantee Inhibition: Aminothiazoles Emerge as Inhibitors of Plasmodium falciparum
Source: ACS Infect Dis. 2024 Feb 17;10(3):1000–22. doi: 10.1021/acsinfecdis.3c00670 (PMC10928712; doi:10.1021/acsinfecdis.3c00670)
Supplement: Supplementary file 2 — id3c00670_si_002.pdf [file id3c00670_si_002.pdf]

Identification of three new inhibitor classes against *Plasmodium falciparum*

Spectra of oxime derivatives

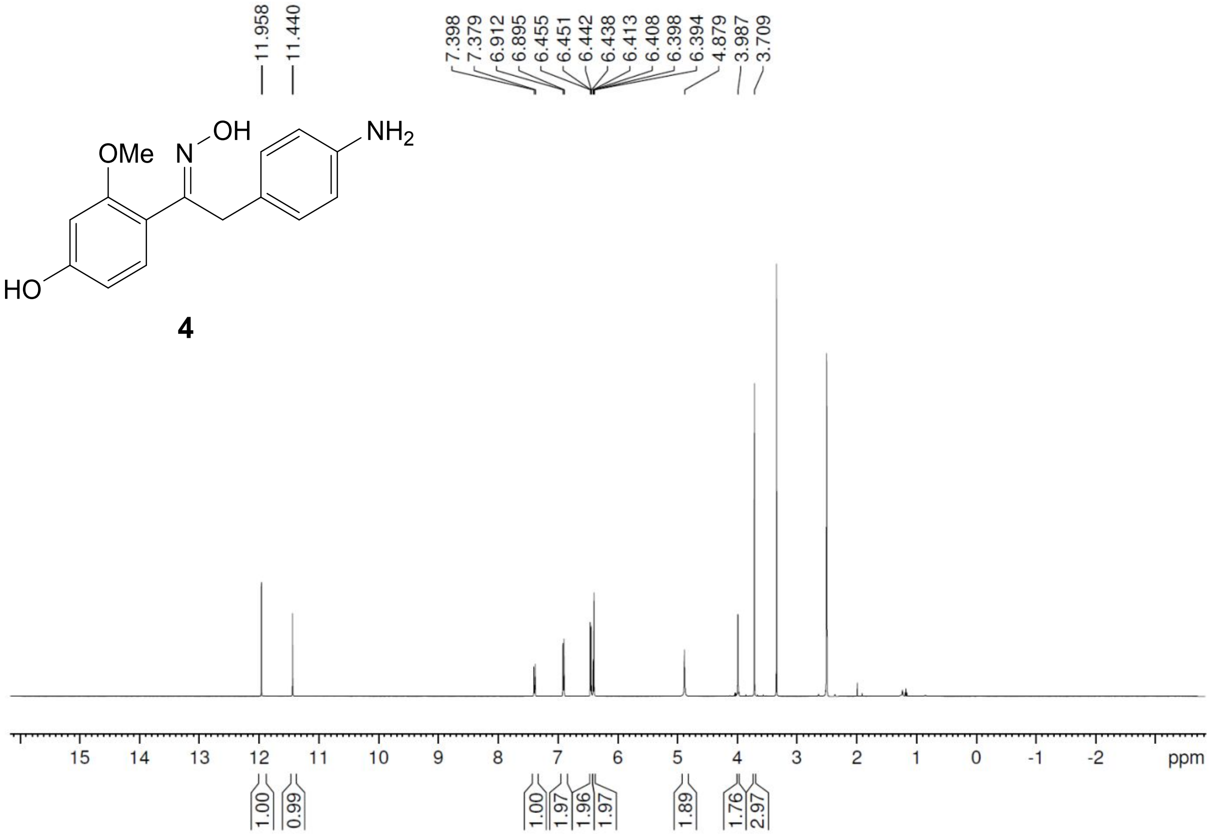

Spectrum 1: ^{1}H-NMR spectrum of oxime **4** in $(CD_3)_2OS$.

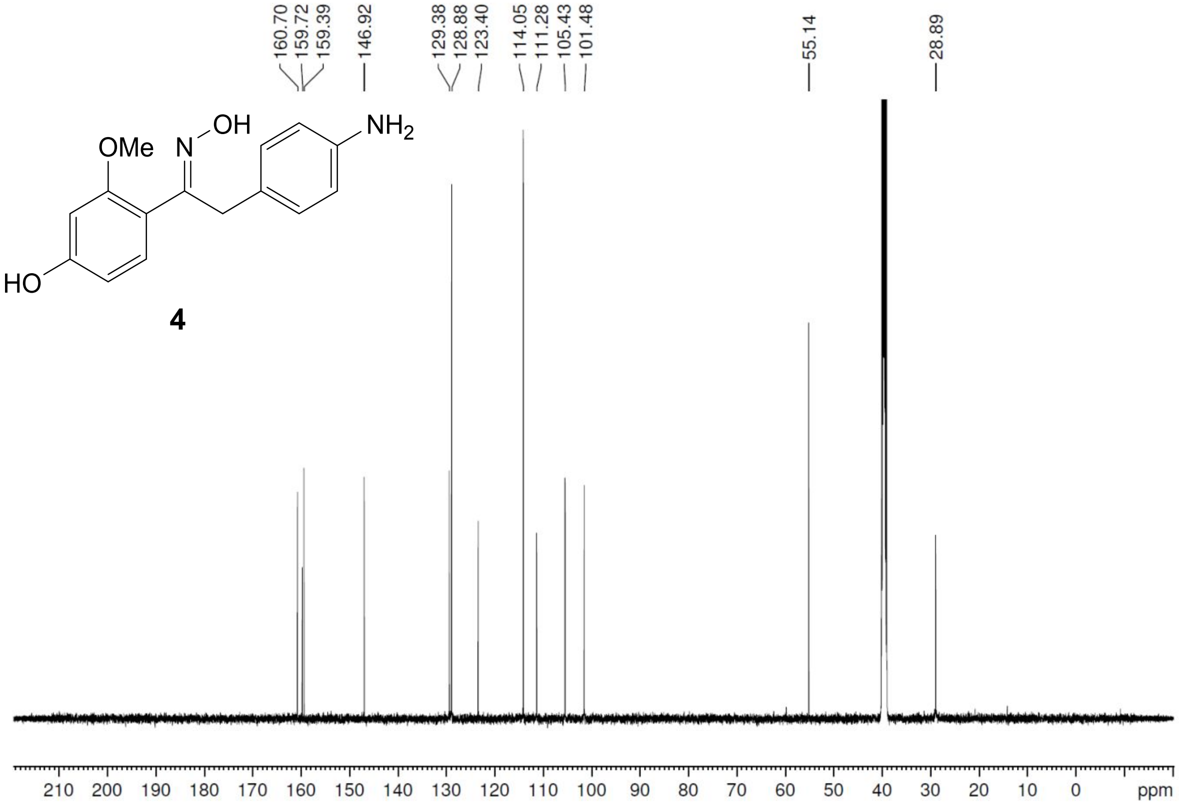

Spectrum 2: ^{13}C-NMR spectrum of oxime **4** in $(CD_3)_2OS$.

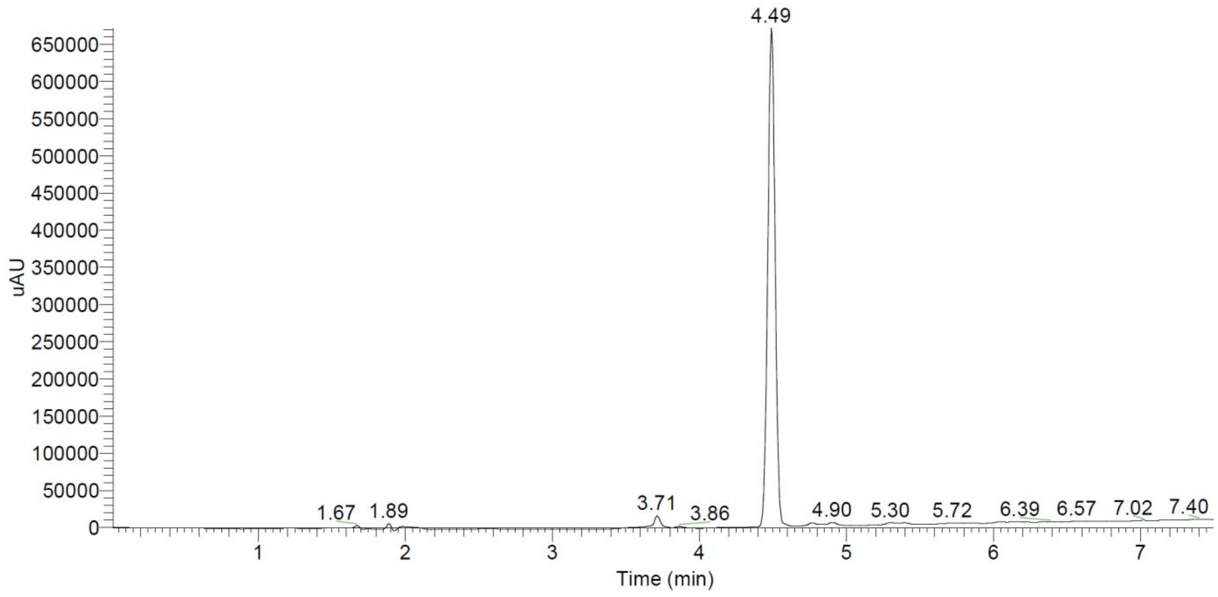

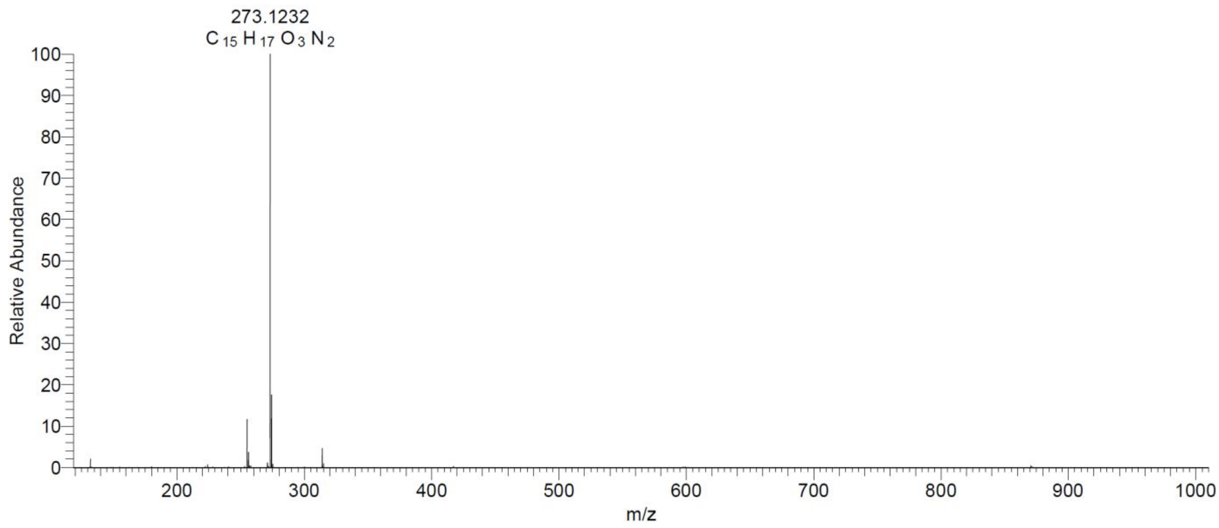

Spectrum 3: UV-trace and mass of main peak of oxime **4.** Purity determined by peak area 98%.

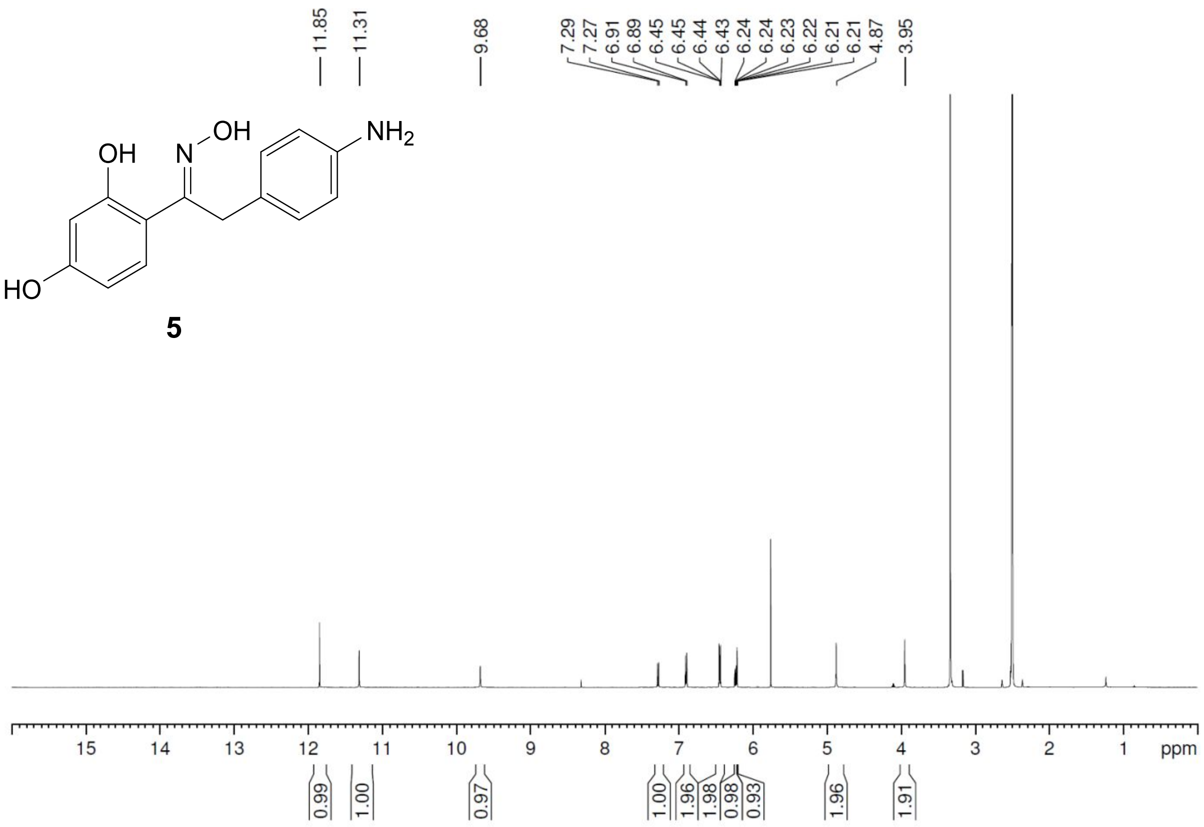

Spectrum 4: ^{1}H-NMR spectrum of oxime **5** in $(CD_3)_2OS$.

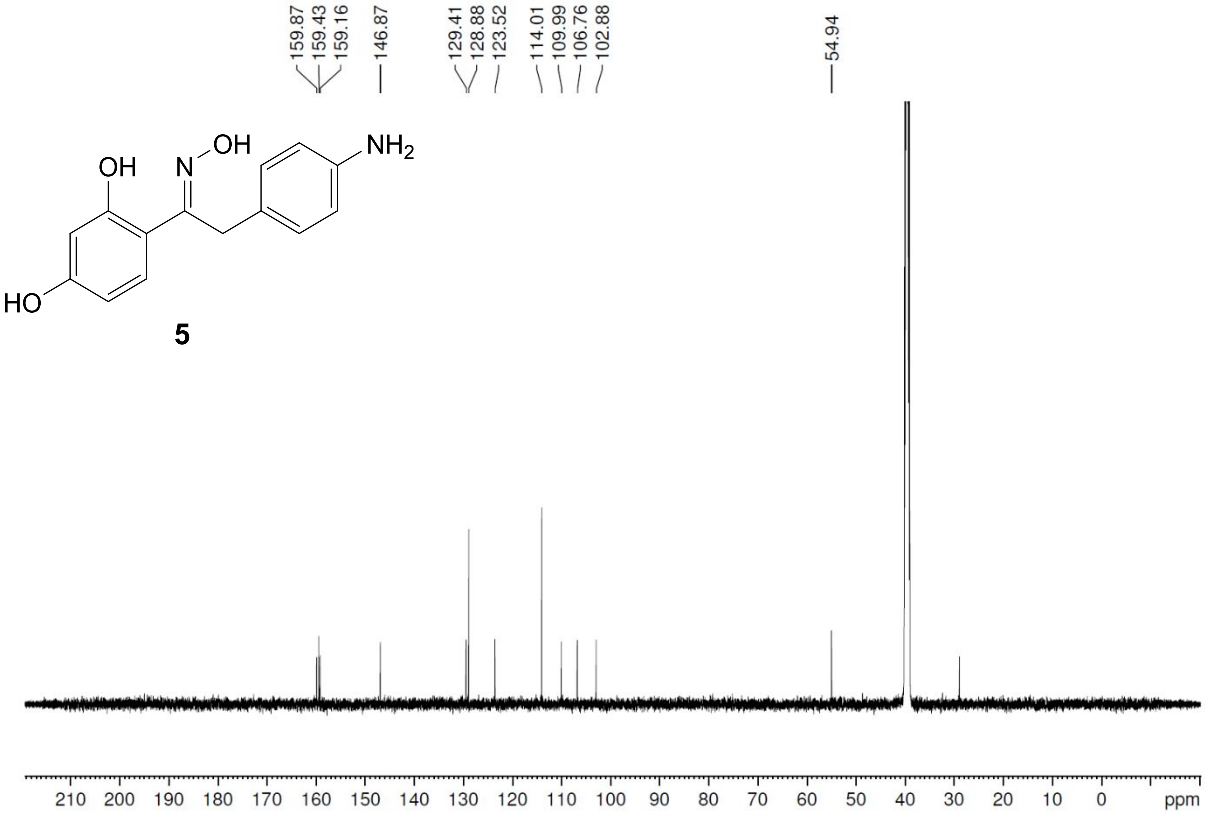

Spectrum 5: ^{13}C-NMR spectrum of oxime **5** in $(CD_3)_2OS$.

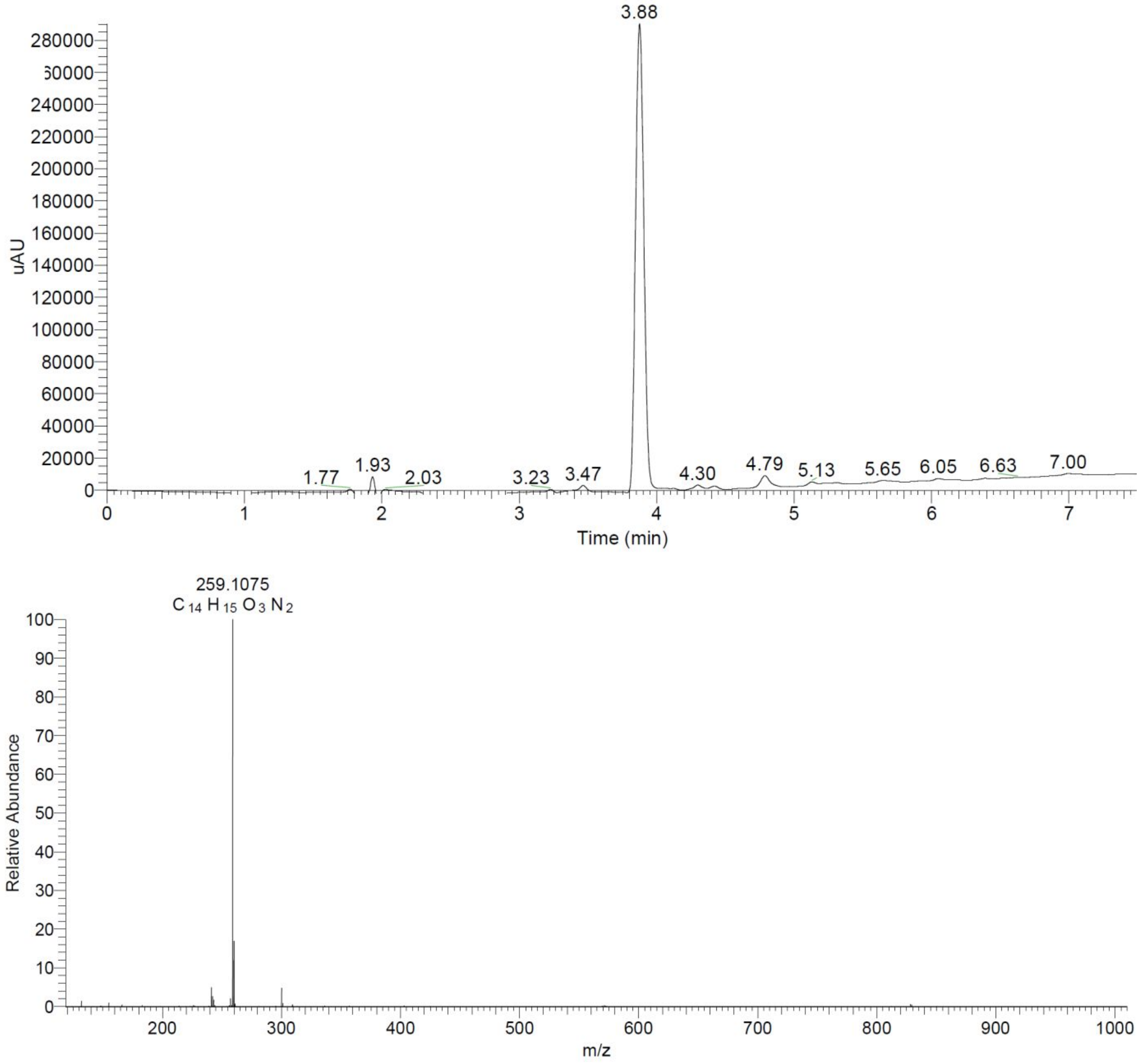

Spectrum 6: UV-trace and mass of main peak of oxime **5**. Purity determined by peak area 95%.

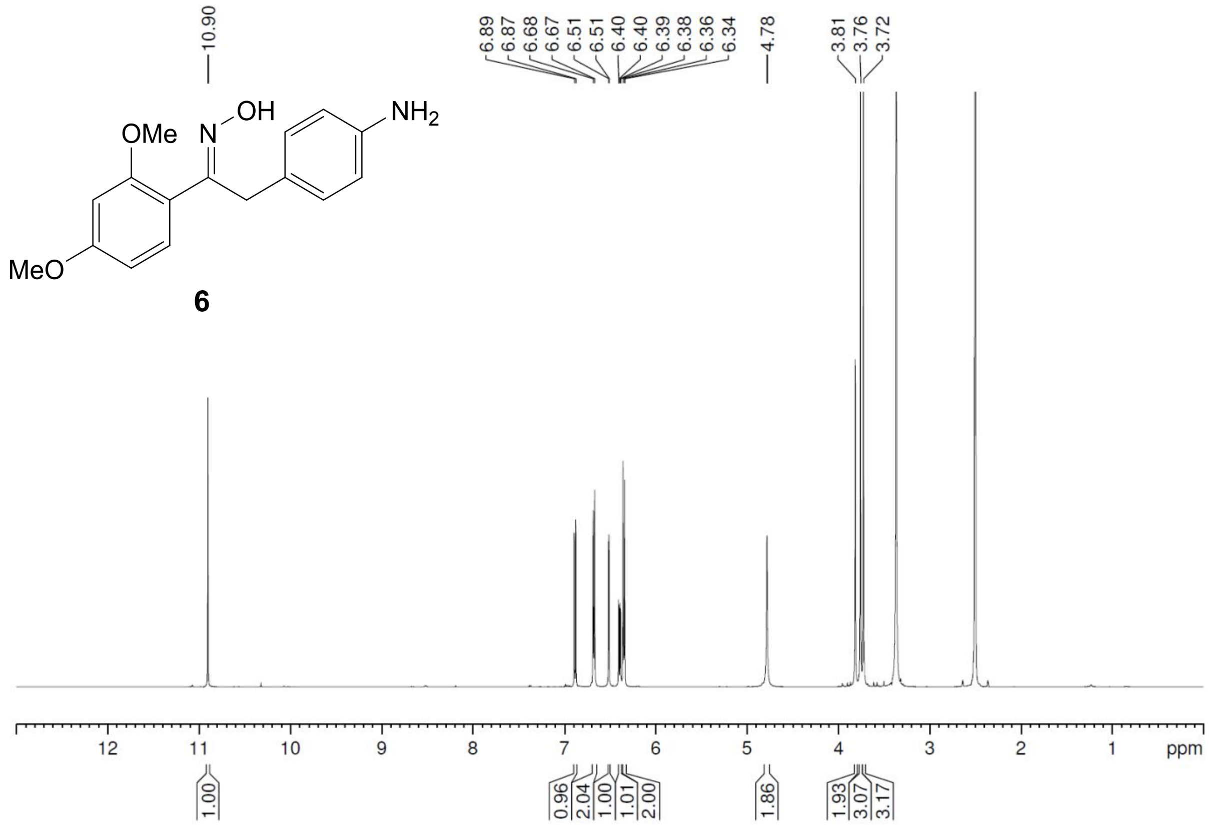

Spectrum 7: ^{1}H-NMR spectrum of oxime **6** in $(CD_3)_2OS$.

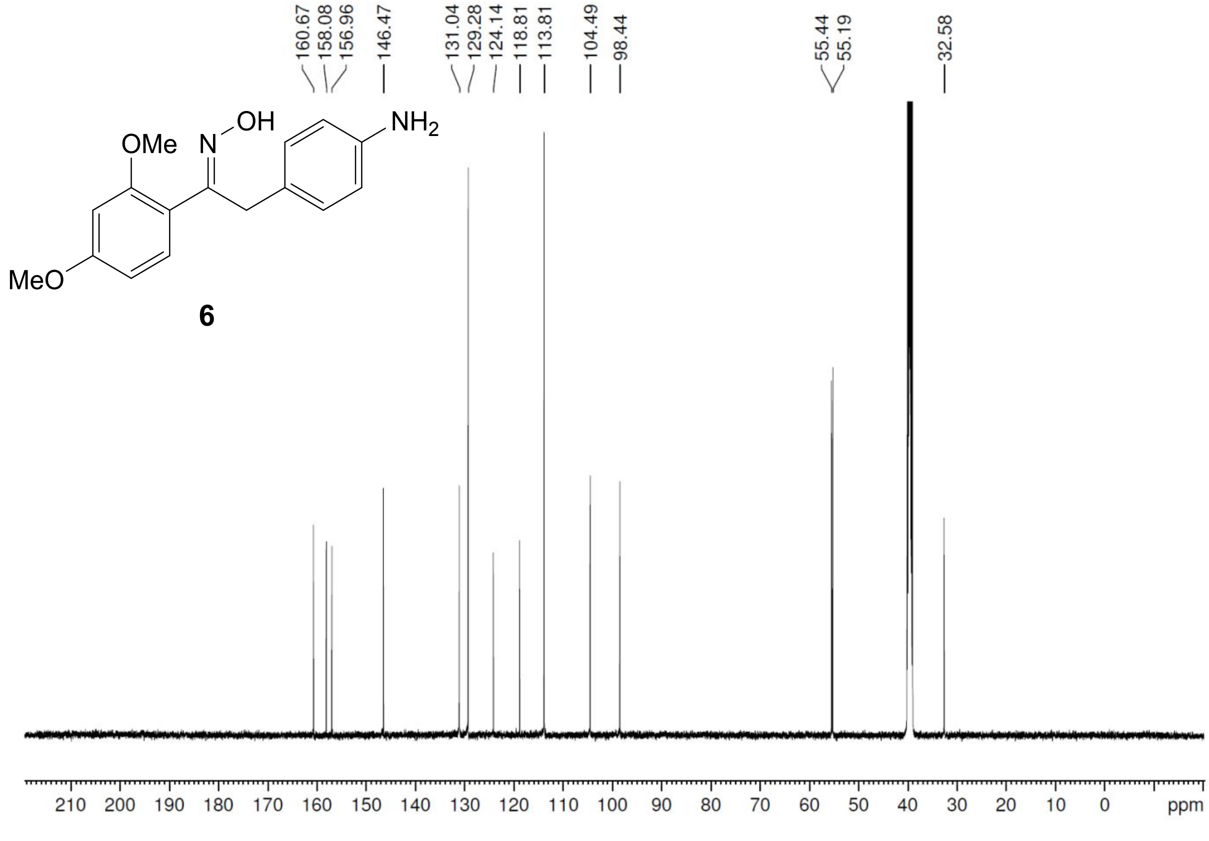

Spectrum 8: ^{13}C-NMR spectrum of oxime **6** in $(CD_3)_2OS$.

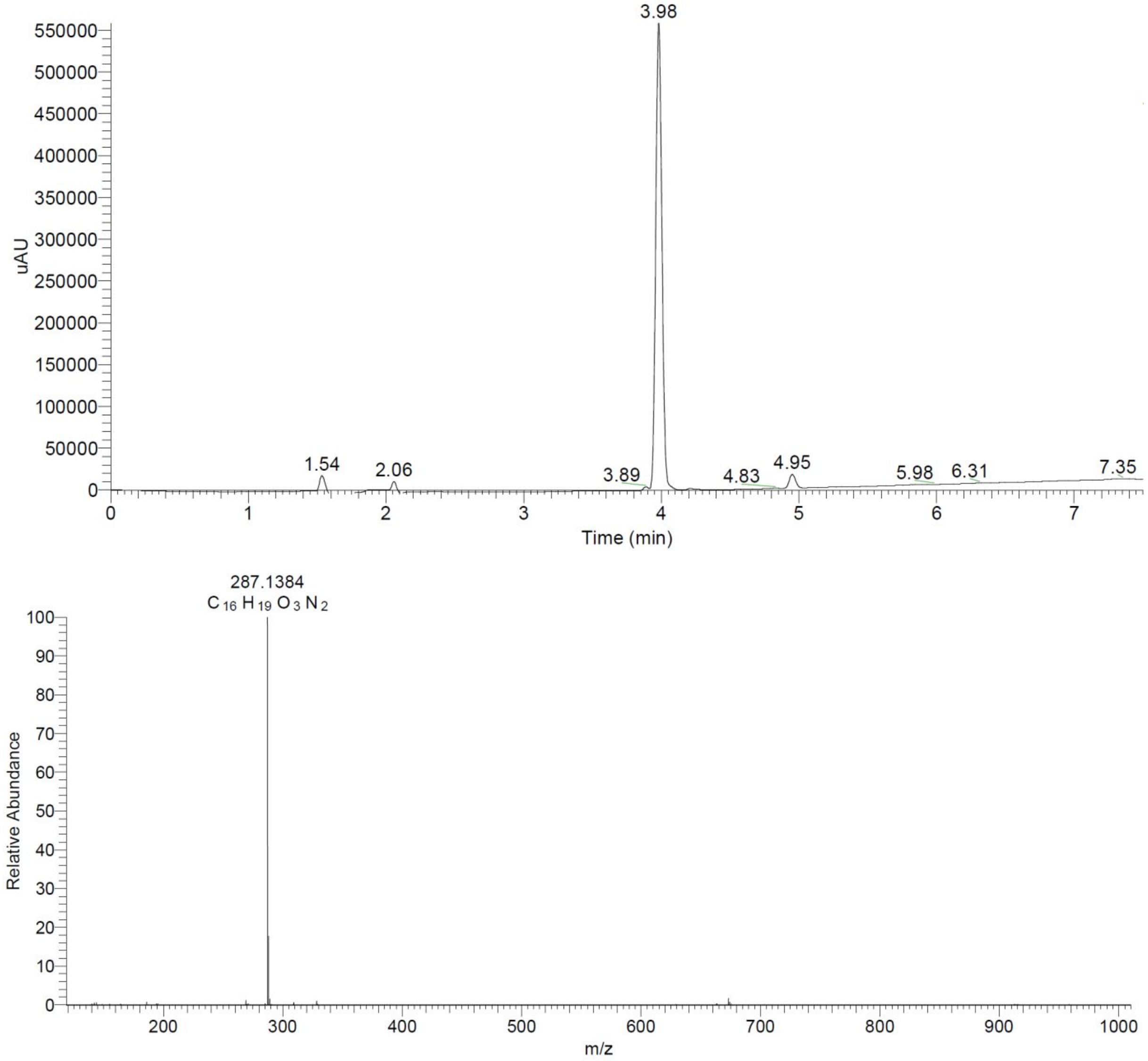

Spectrum 9: UV-trace and mass of main peak of oxime **6**. Purity determined by peak area 97%.

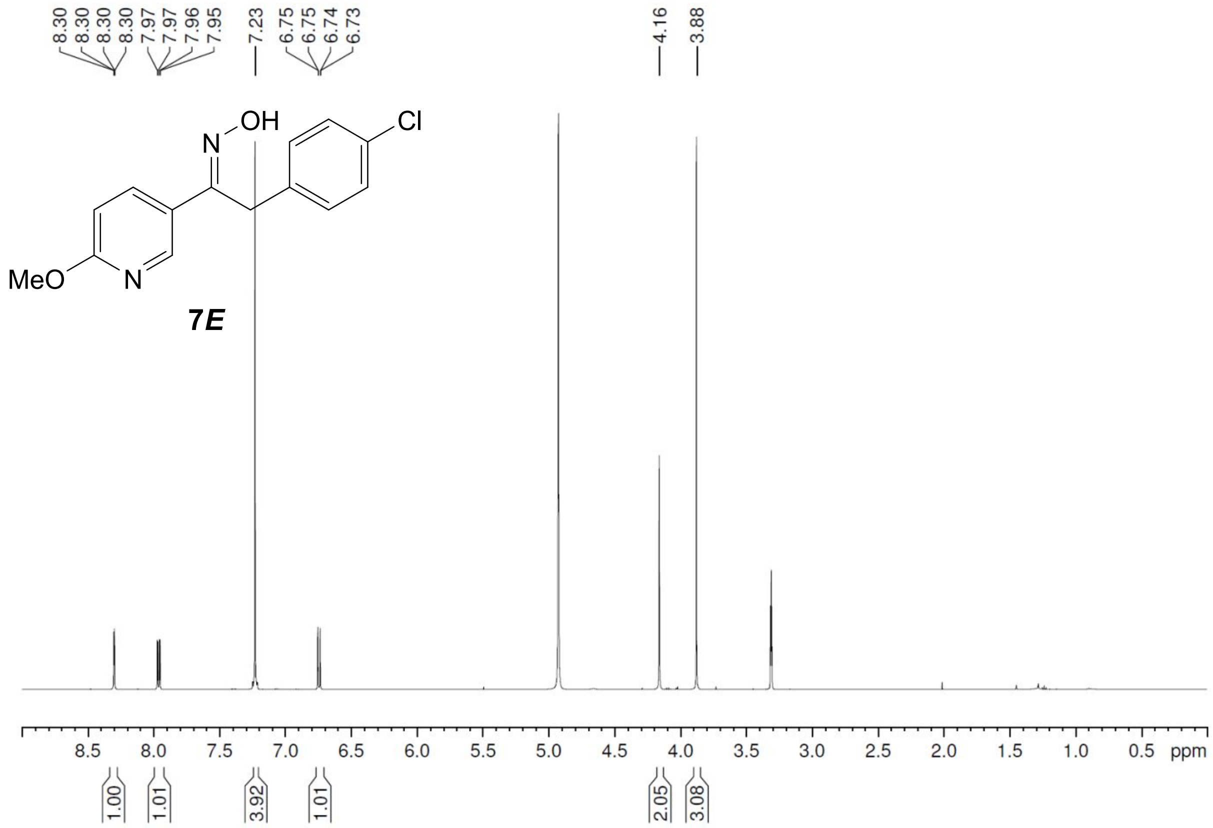

Spectrum 10: ^{1}H-NMR spectrum of oxime ***7E*** in CD_3OD.

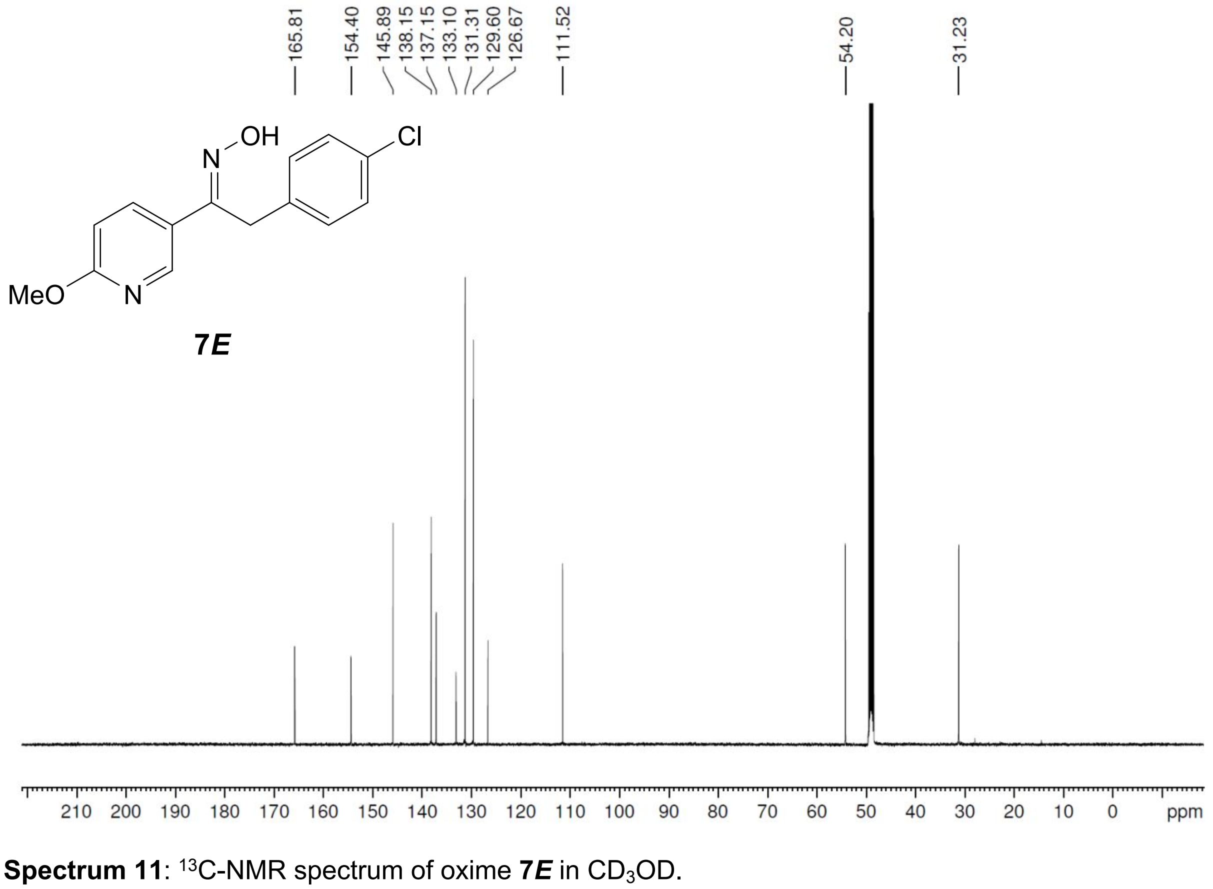

Spectrum 11: ^{13}C-NMR spectrum of oxime ***7E*** in CD_3OD.

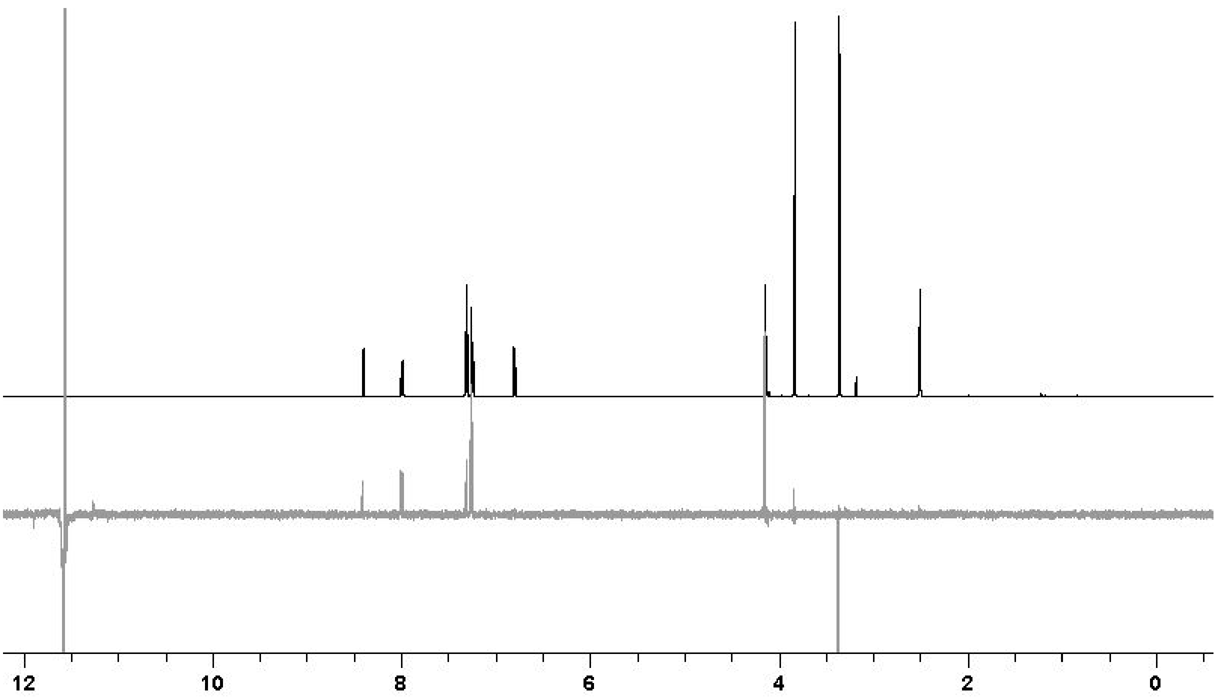

Spectrum 12: 1D-NOESY-NMR spectrum of oxime ***7E*** in $(CD_3)_2OS$. Black: ^{1}H-NMR, grey: 1D-NOESY. Irradiation time 800 ms.

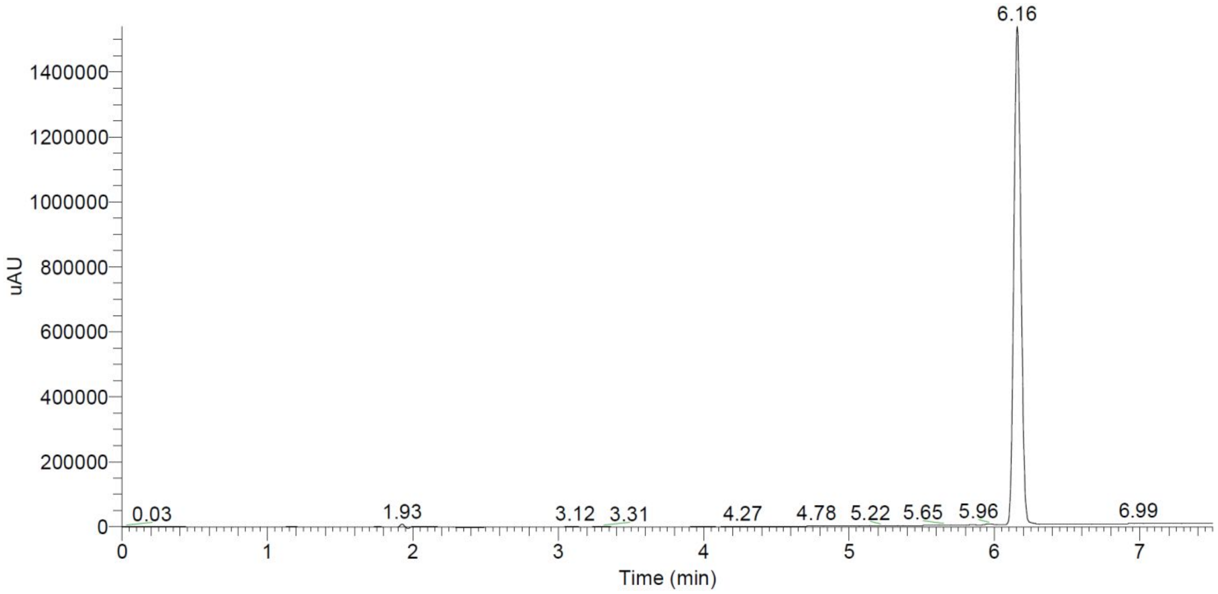

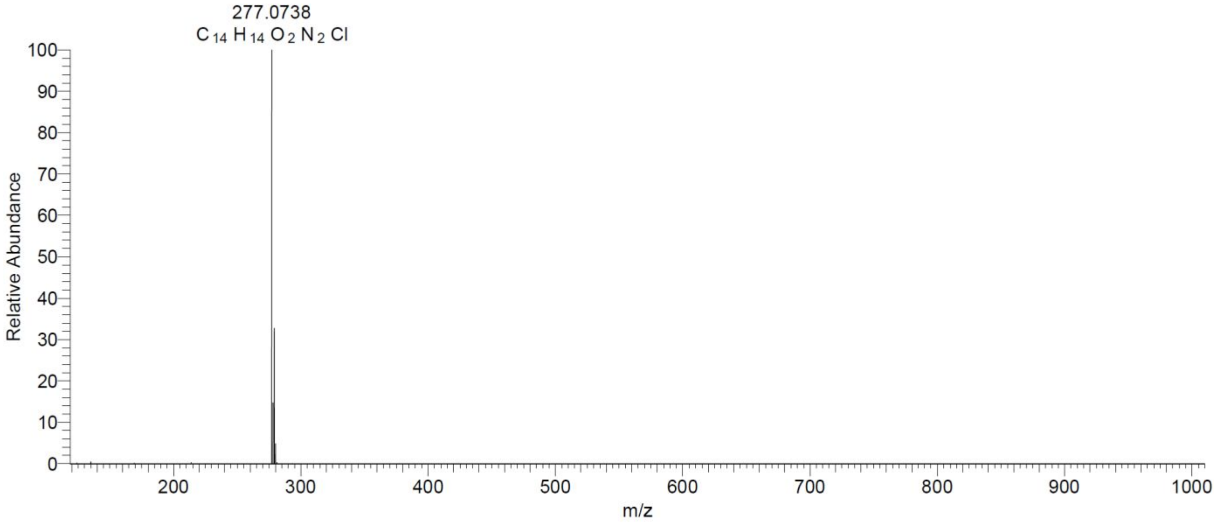

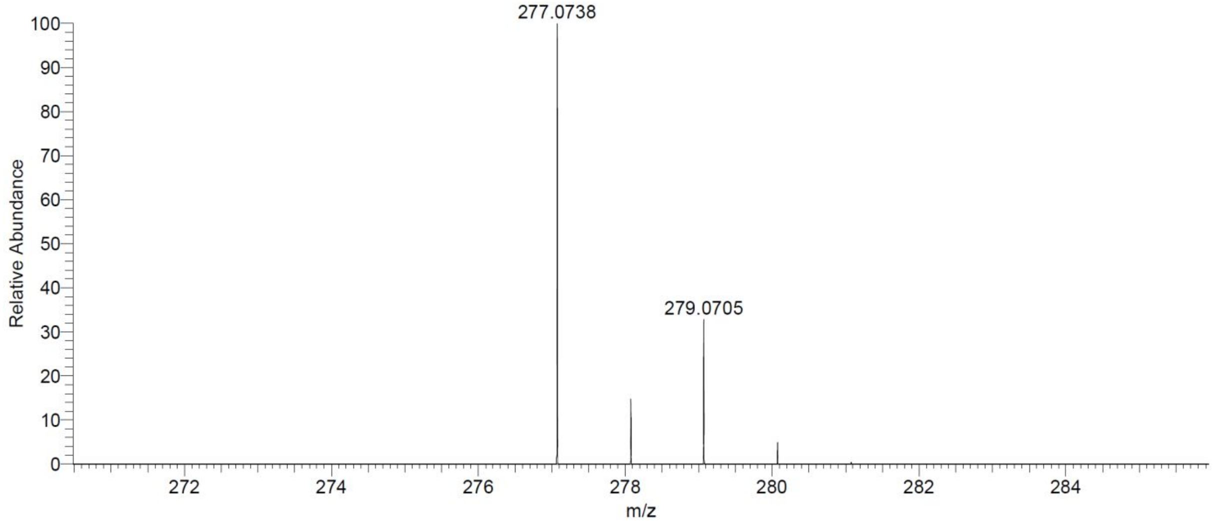

Spectrum 13: UV-trace and mass of main peak of oxime **7*E***. Purity determined by peak area 99%.

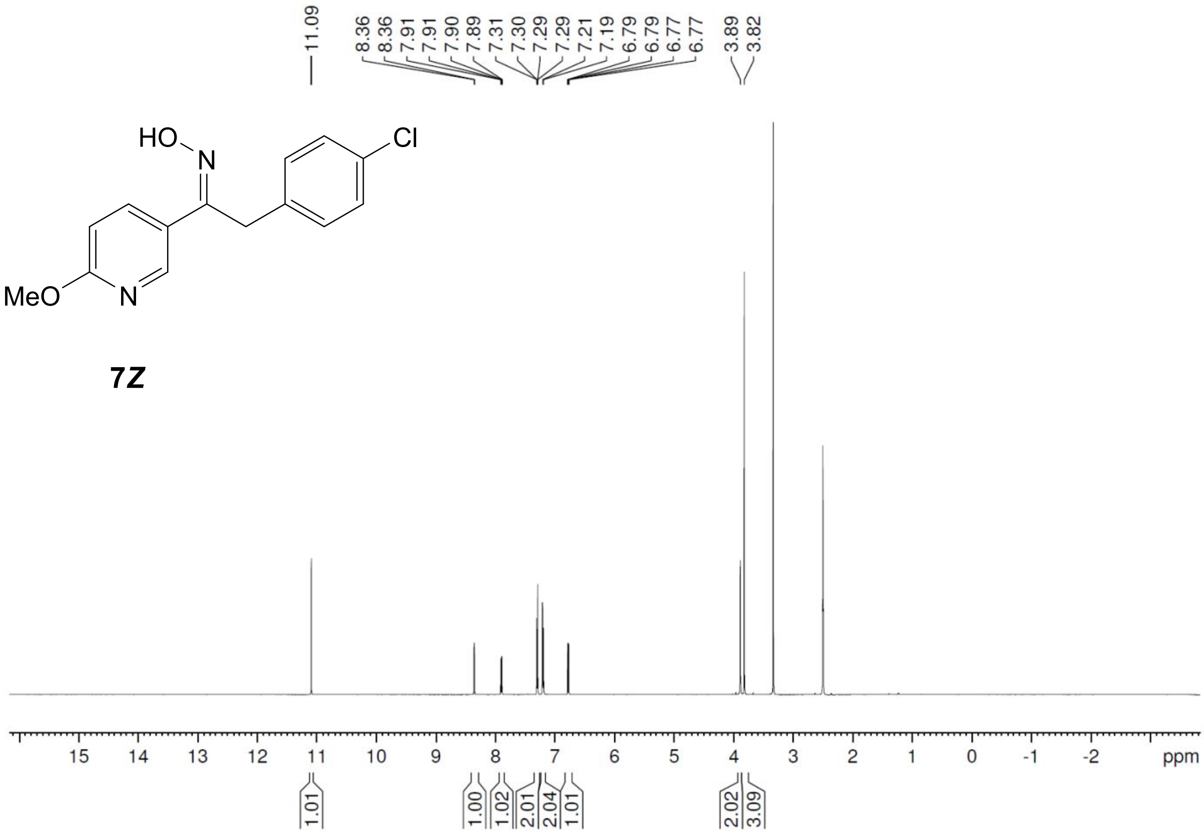

Spectrum 14: ^{1}H-NMR spectrum of oxime **7Z** in $(CD_3)_2OS$.

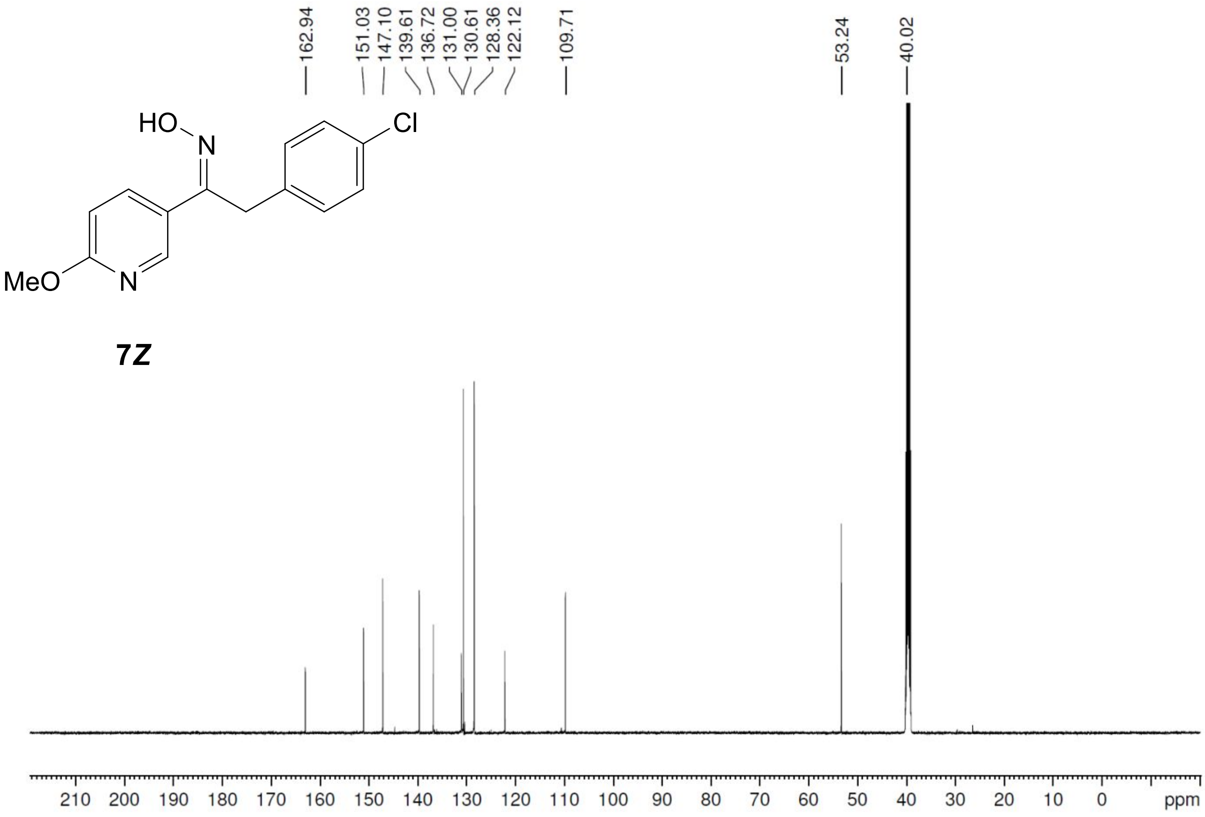

Spectrum 15: ^{13}C-NMR spectrum of oxime **7Z** in $(CD_3)_2OS$.

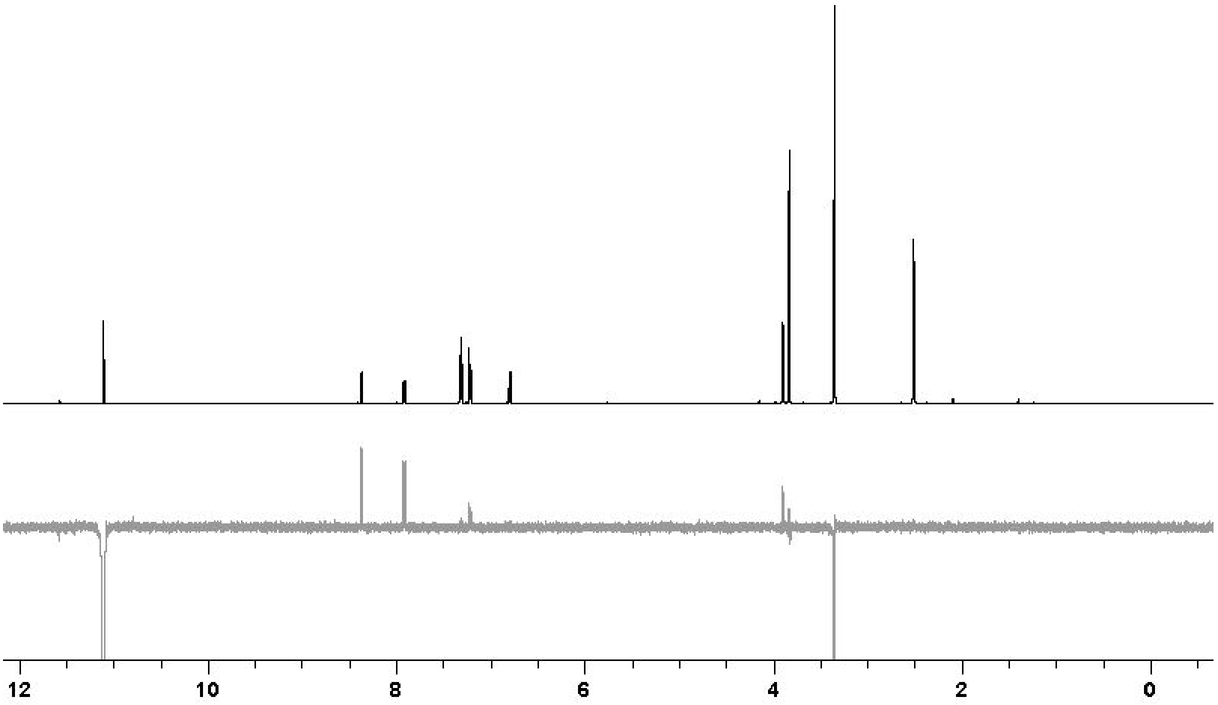

Spectrum 16: 1D-NOESY-NMR spectrum of oxime **7*Z*** in $(CD_3)_2OS$. Black: ^{1}H-NMR, grey: 1D-NOESY. Irradiation time 800 ms.

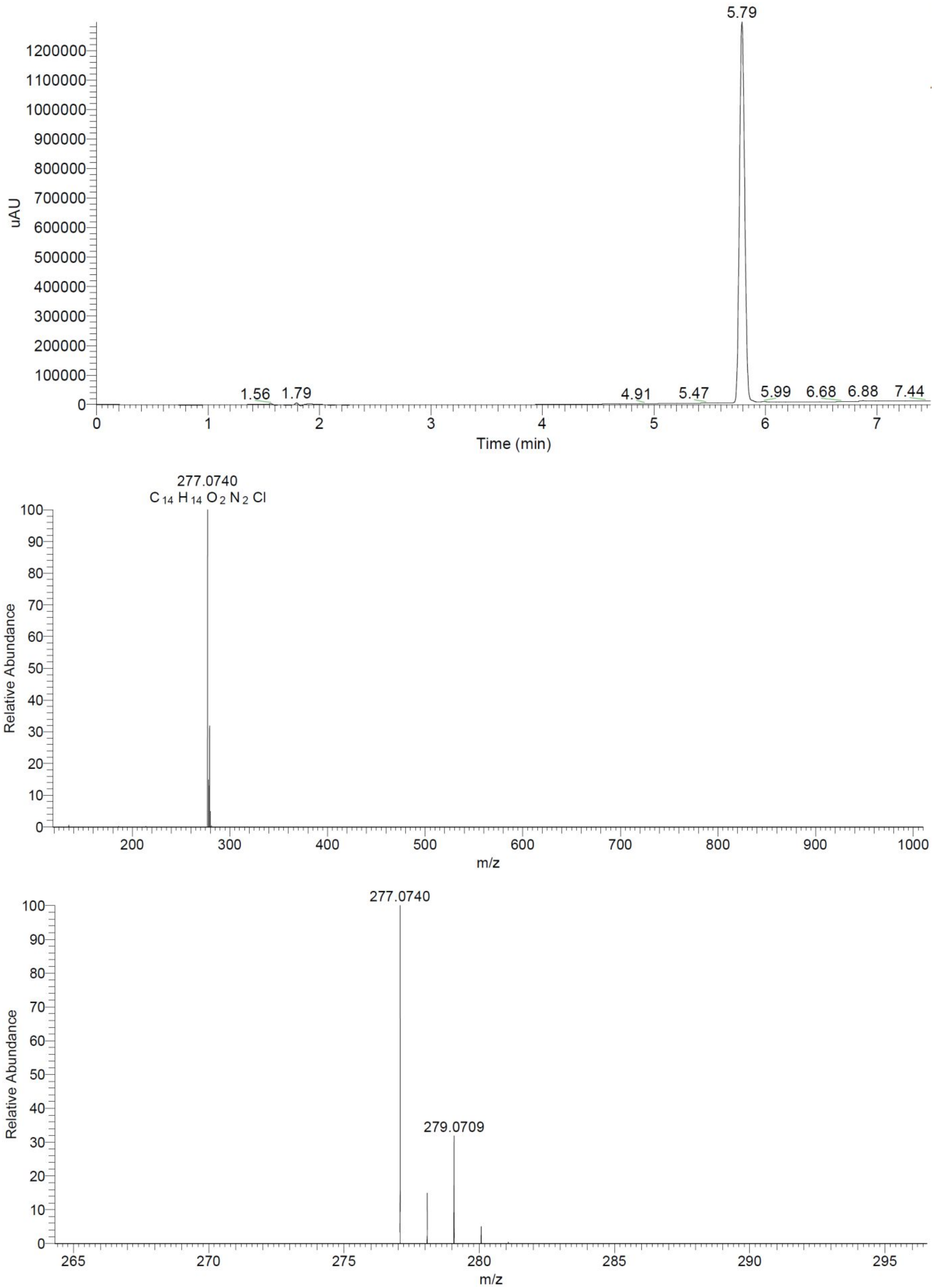

Spectrum 17: UV-trace and mass of main peak of oxime **7Z**. Purity determined by peak area 99%.

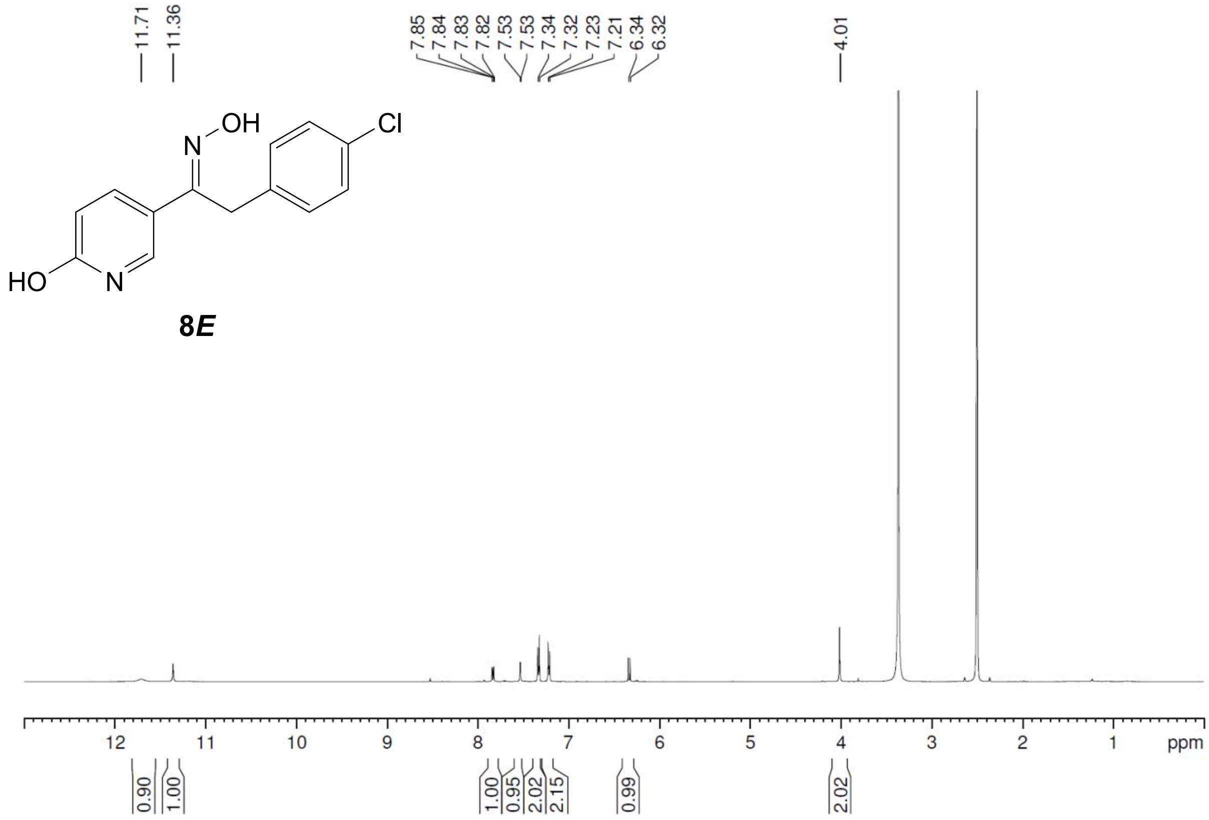

Spectrum 18: ^{1}H-NMR spectrum of oxime **8*E*** in $(CD_3)_2OS$.

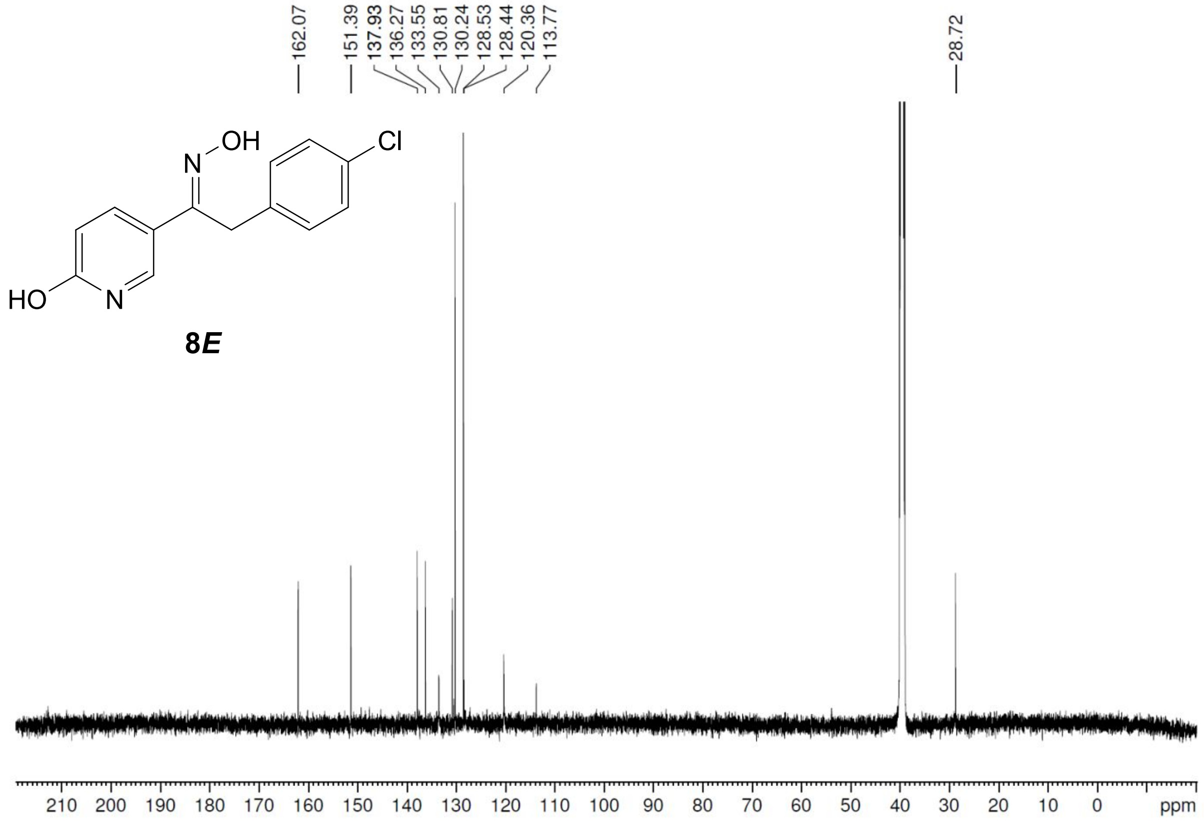

Spectrum 19: ^{13}C-NMR spectrum of oxime **8*E*** in $(CD_3)_2OS$.

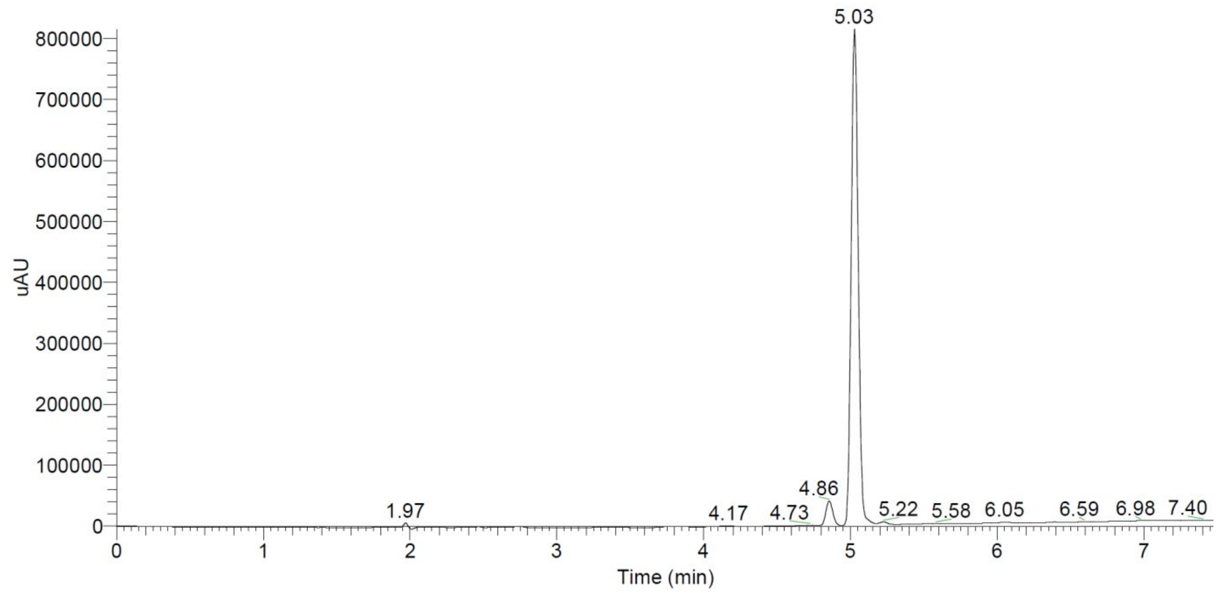

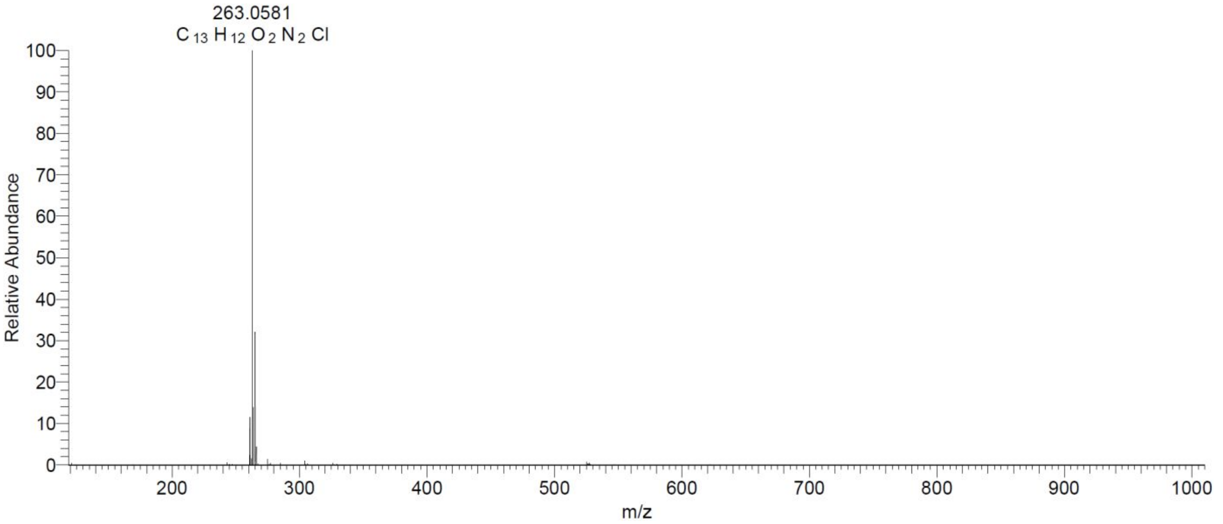

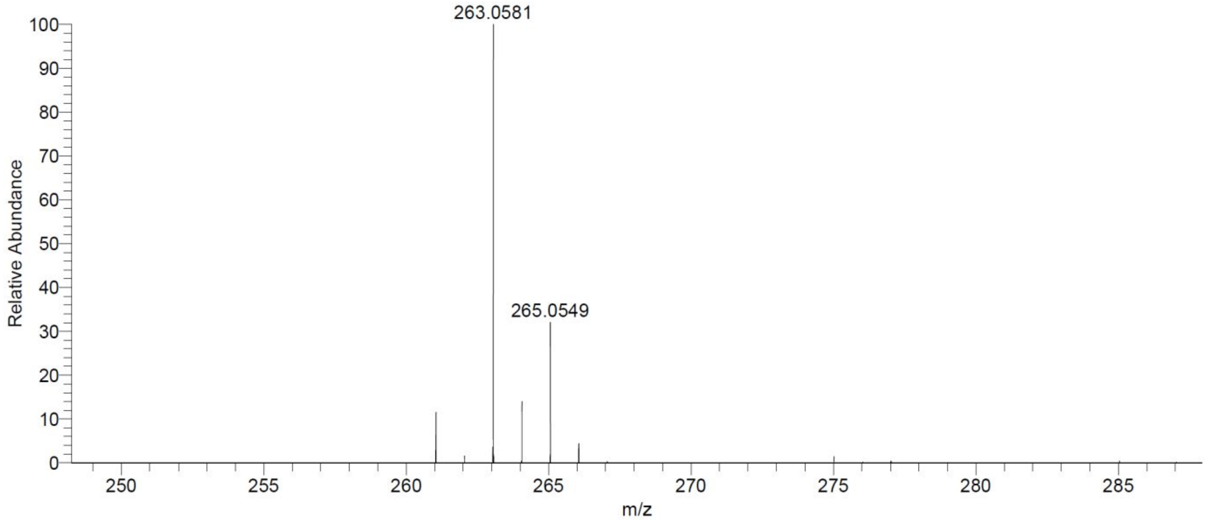

Spectrum 20: UV-trace and mass of main peak of oxime **8*E***. Purity determined by peak area 95%.

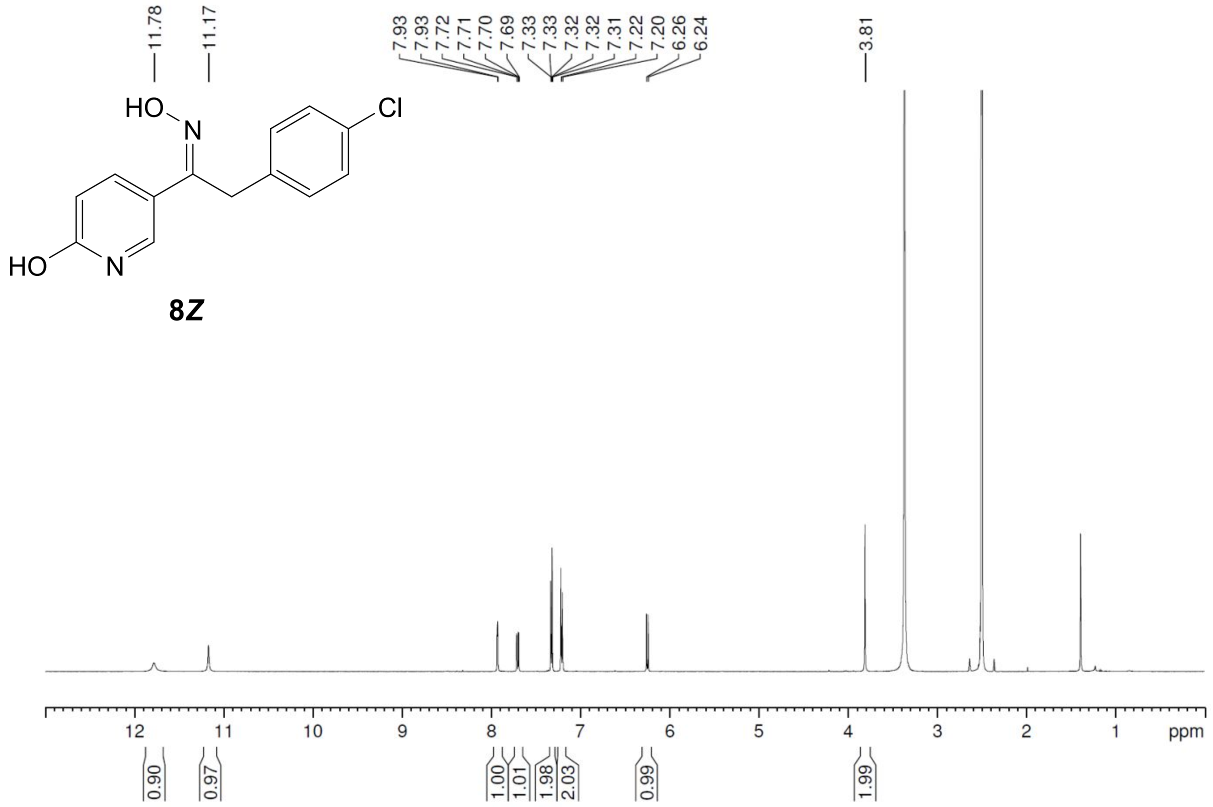

Spectrum 21: ^{1}H-NMR spectrum of oxime **8*Z*** in $(CD_3)_2OS$.

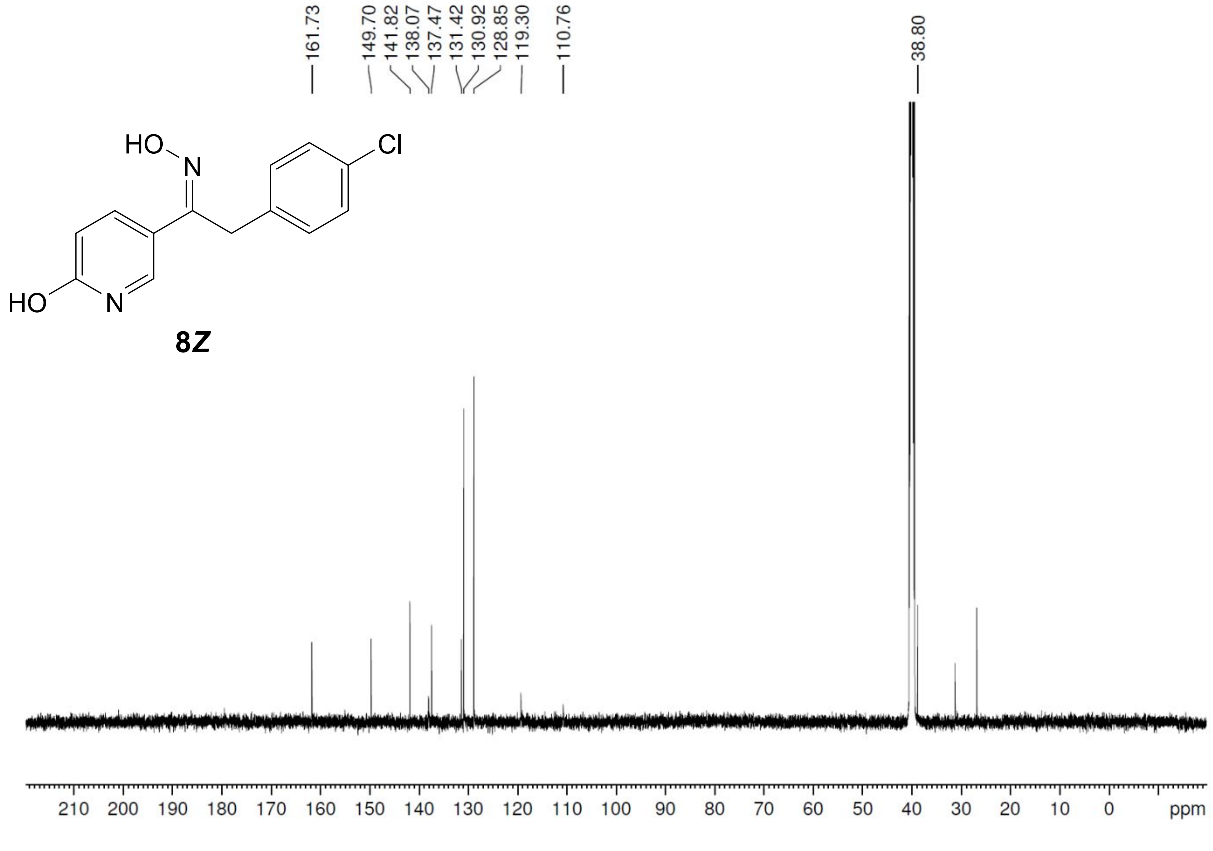

Spectrum 22: ^{13}C-NMR spectrum of oxime **8*Z*** in $(CD_3)_2OS$.

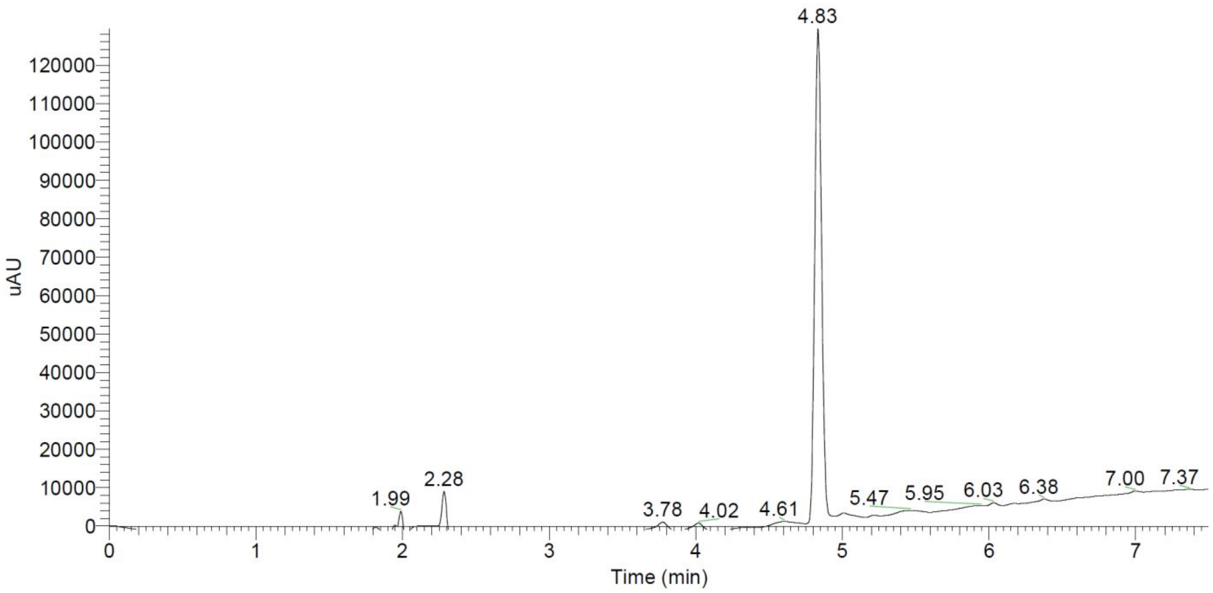

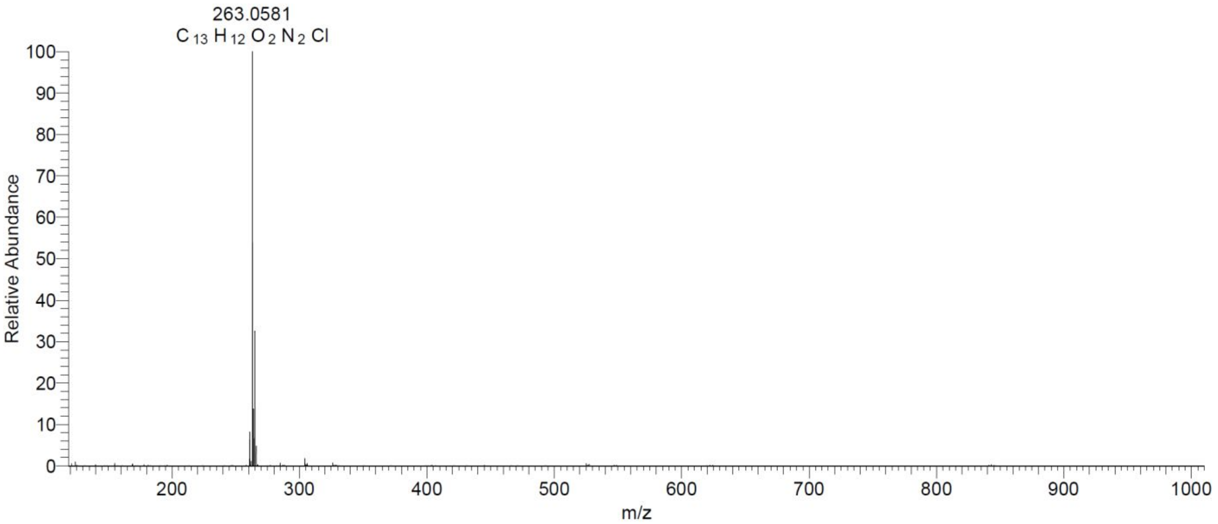

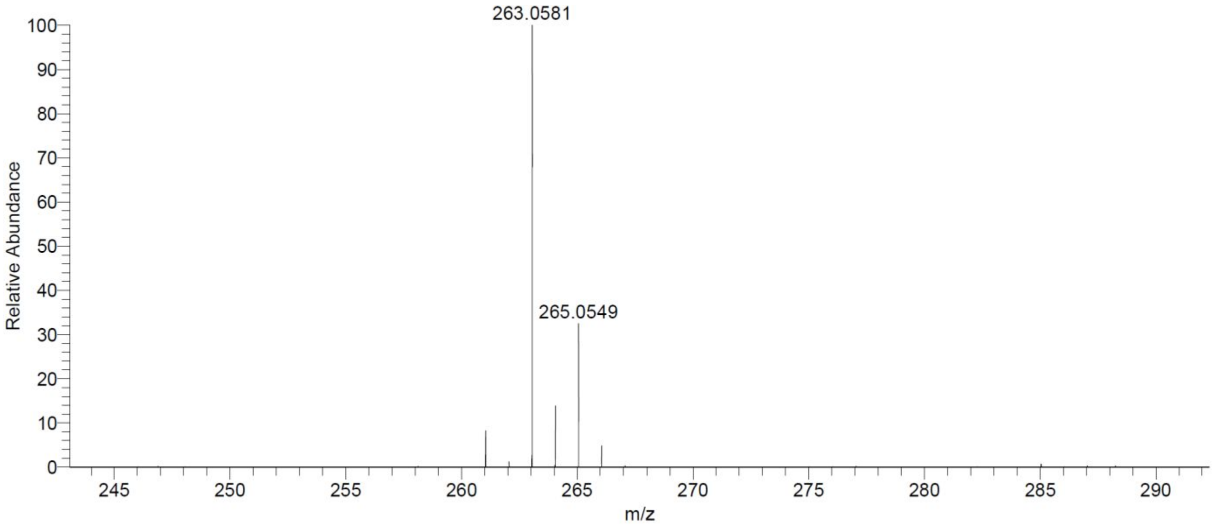

Spectrum 23: UV-trace and mass of main peak of oxime **8Z**. Purity determined by peak area 97%.

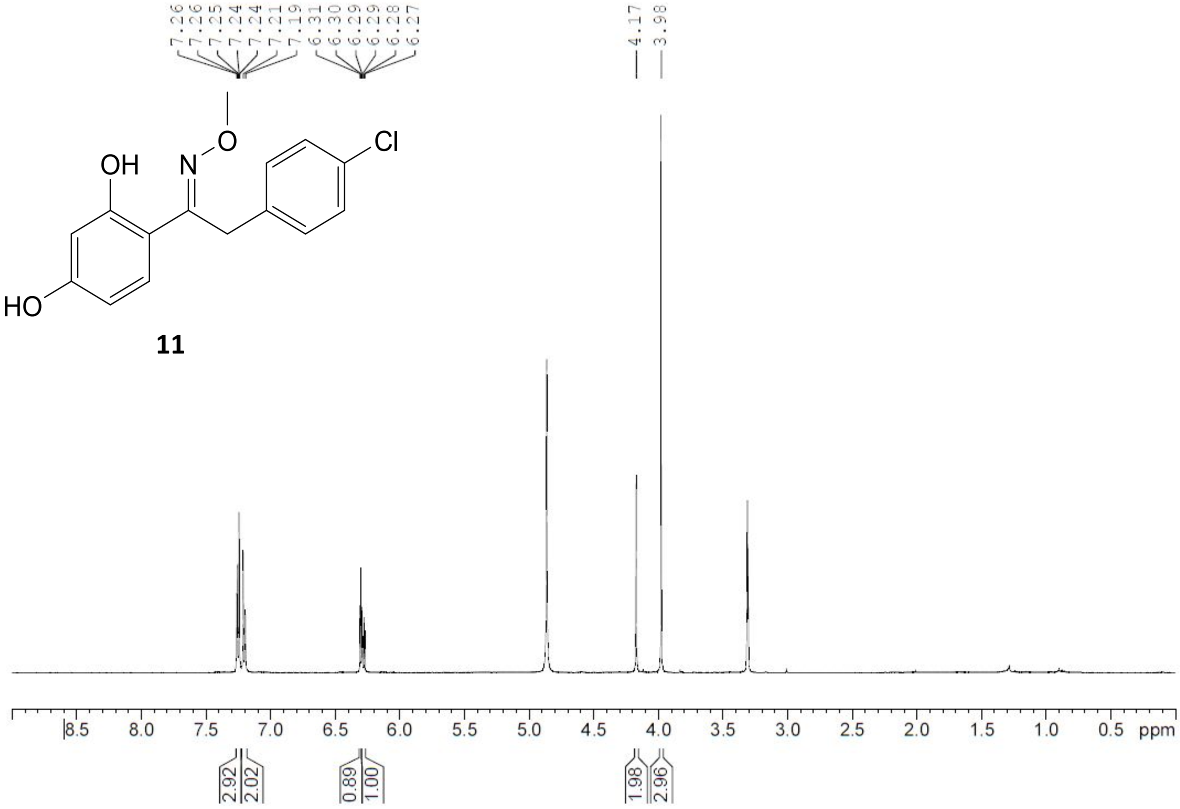

Spectrum 24: ^{1}H-NMR spectrum of oxime derivative **11** in CD_3OD.

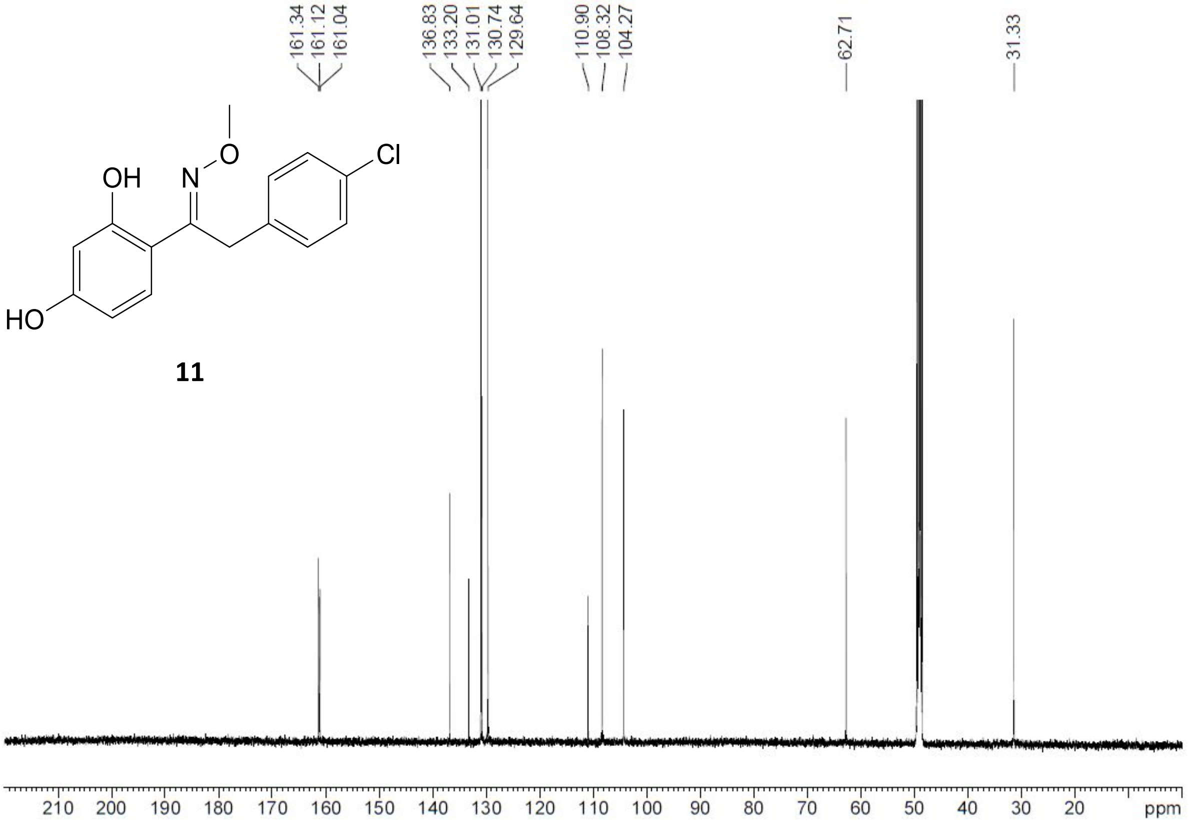

Spectrum 25: ^{13}C-NMR spectrum of oxime derivative **11** in CD_3OD.

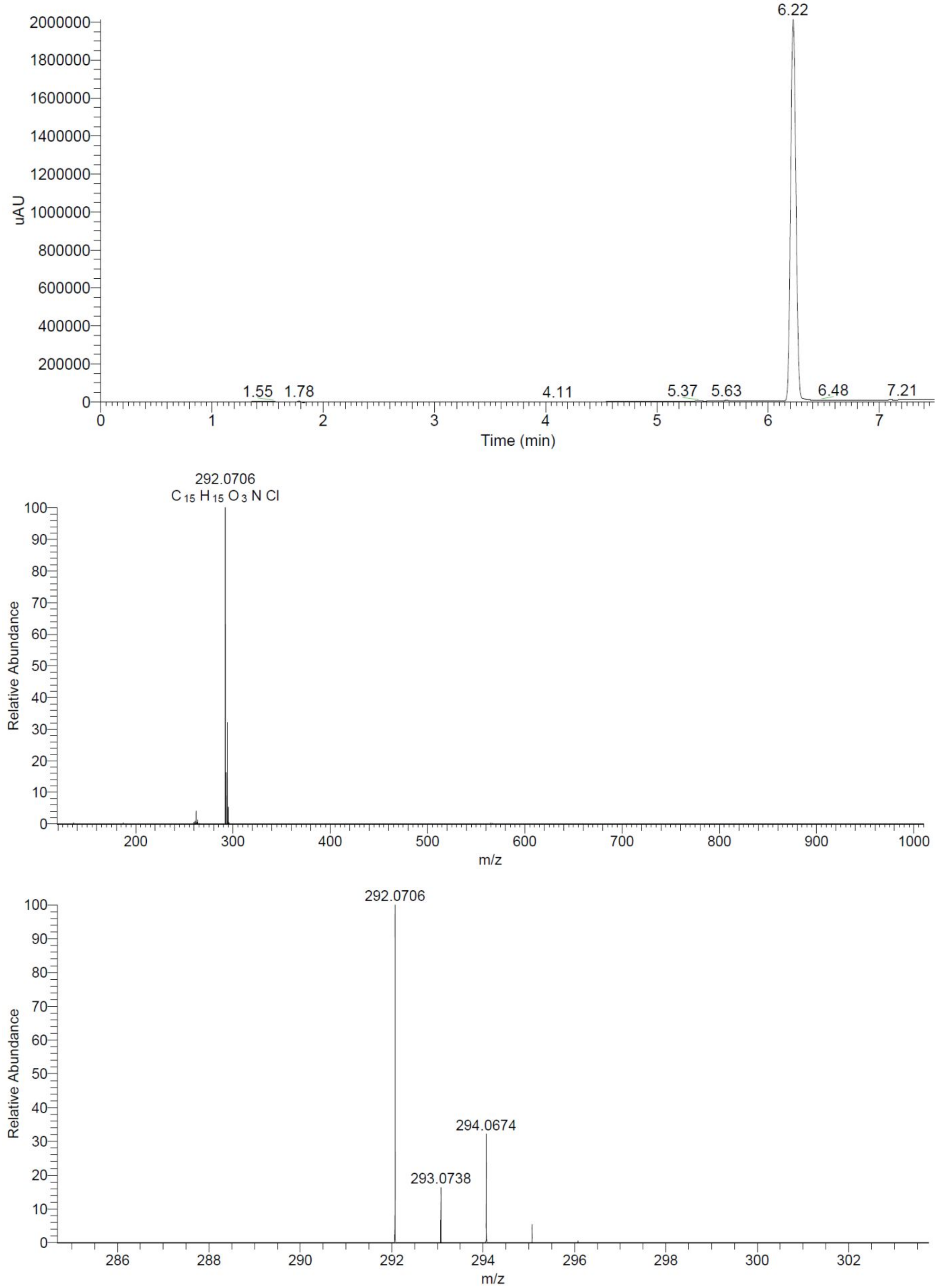

Spectrum 26: UV-trace and mass of main peak of oxime **11**. Purity determined by peak area 99%.

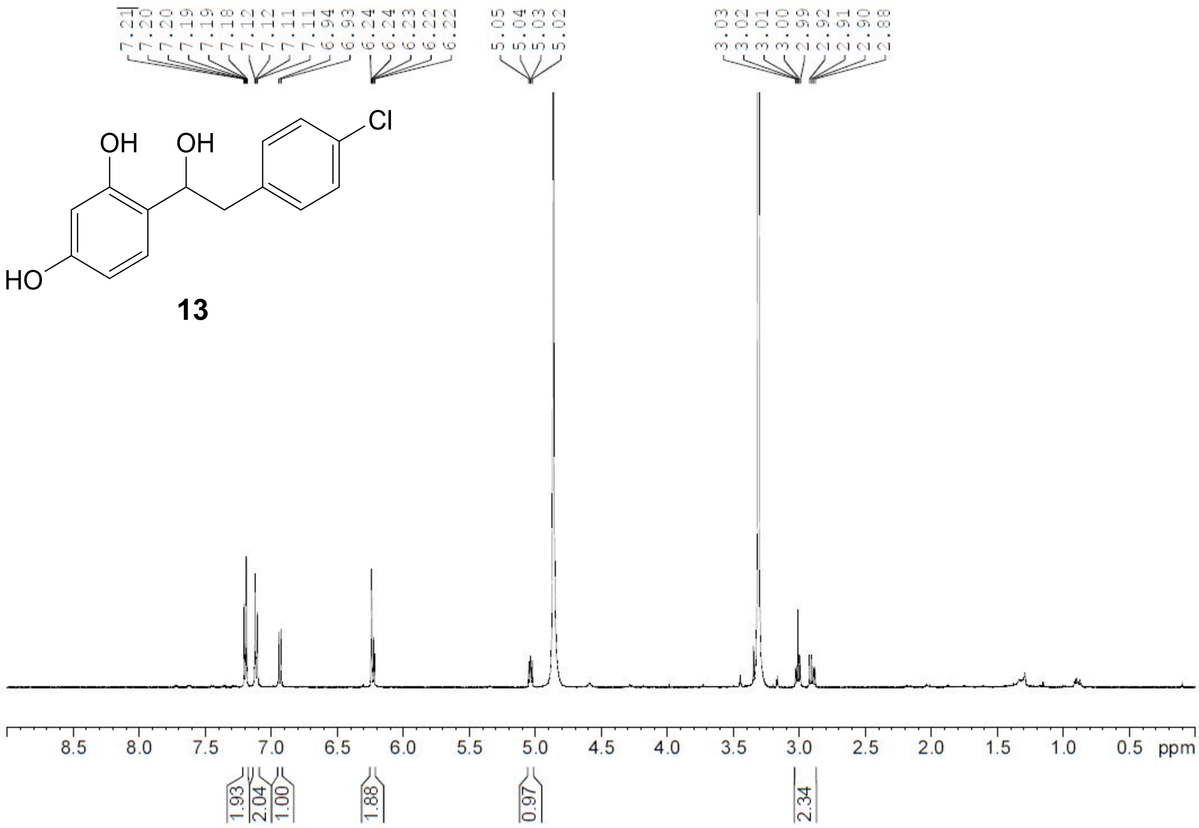

Spectrum 27: ^{1}H-NMR spectrum of oxime derivative **13** in CD_3OD.

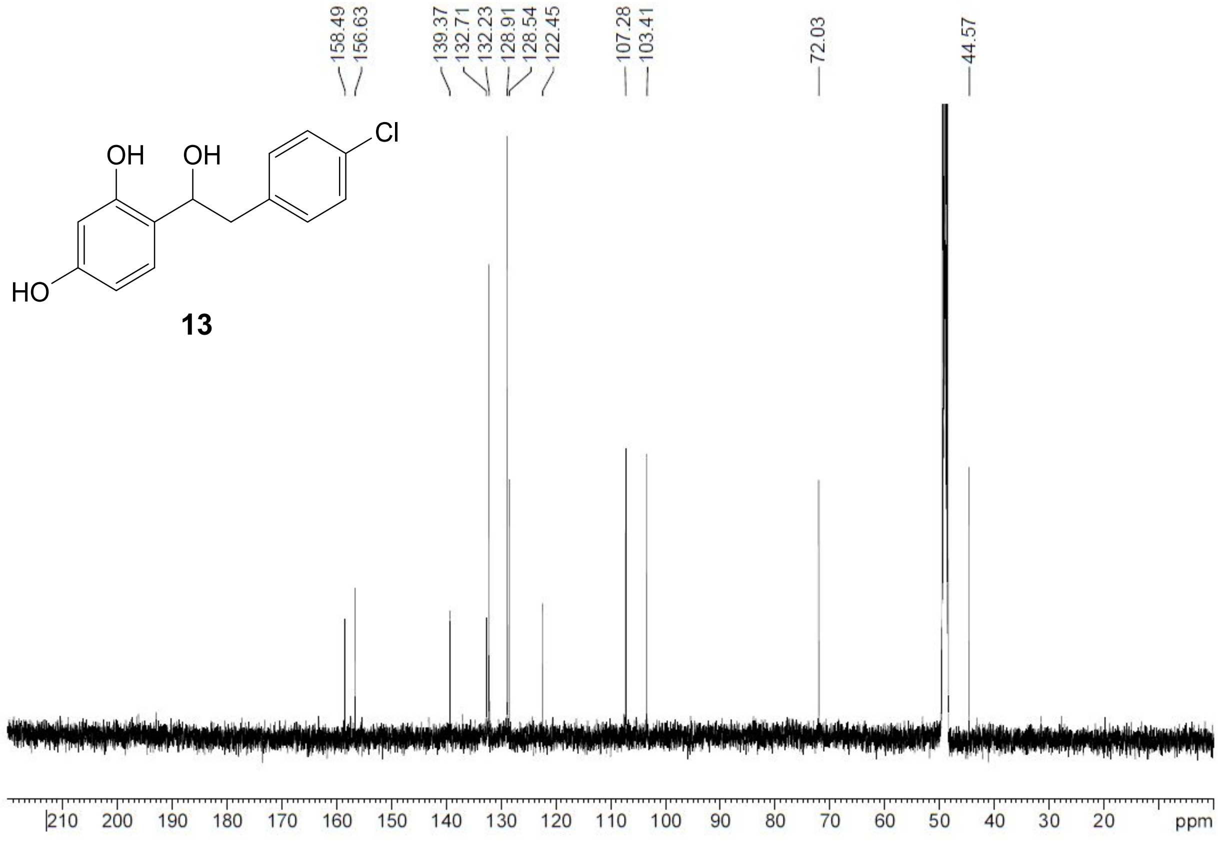

Spectrum 28: ^{13}C-NMR spectrum of oxime derivative **13** in CD_3OD.

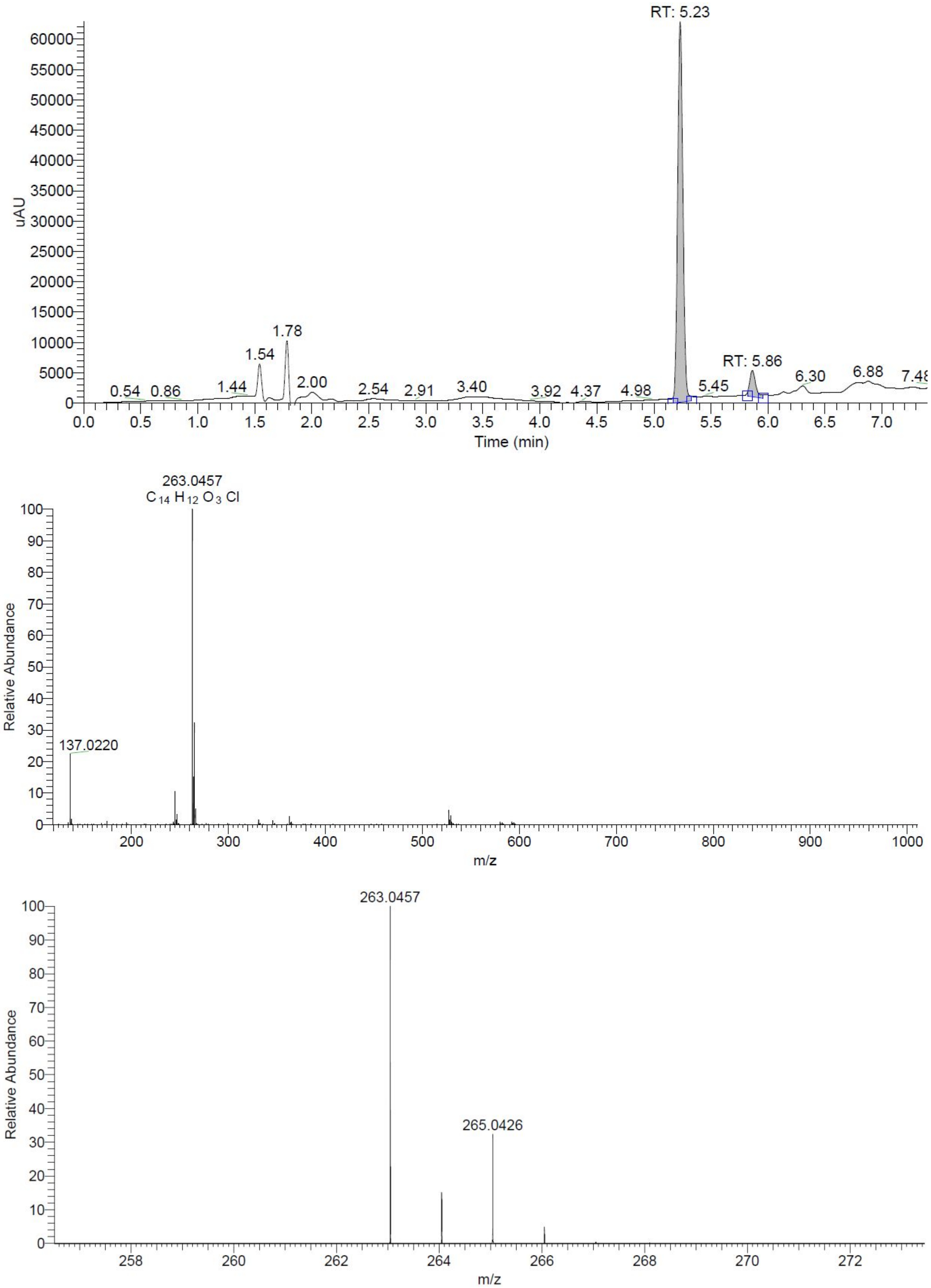

Spectrum 29: UV-trace and mass of main peak of oxime **13**. Purity determined by peak area 95%.

Spectra of indole derivatives:

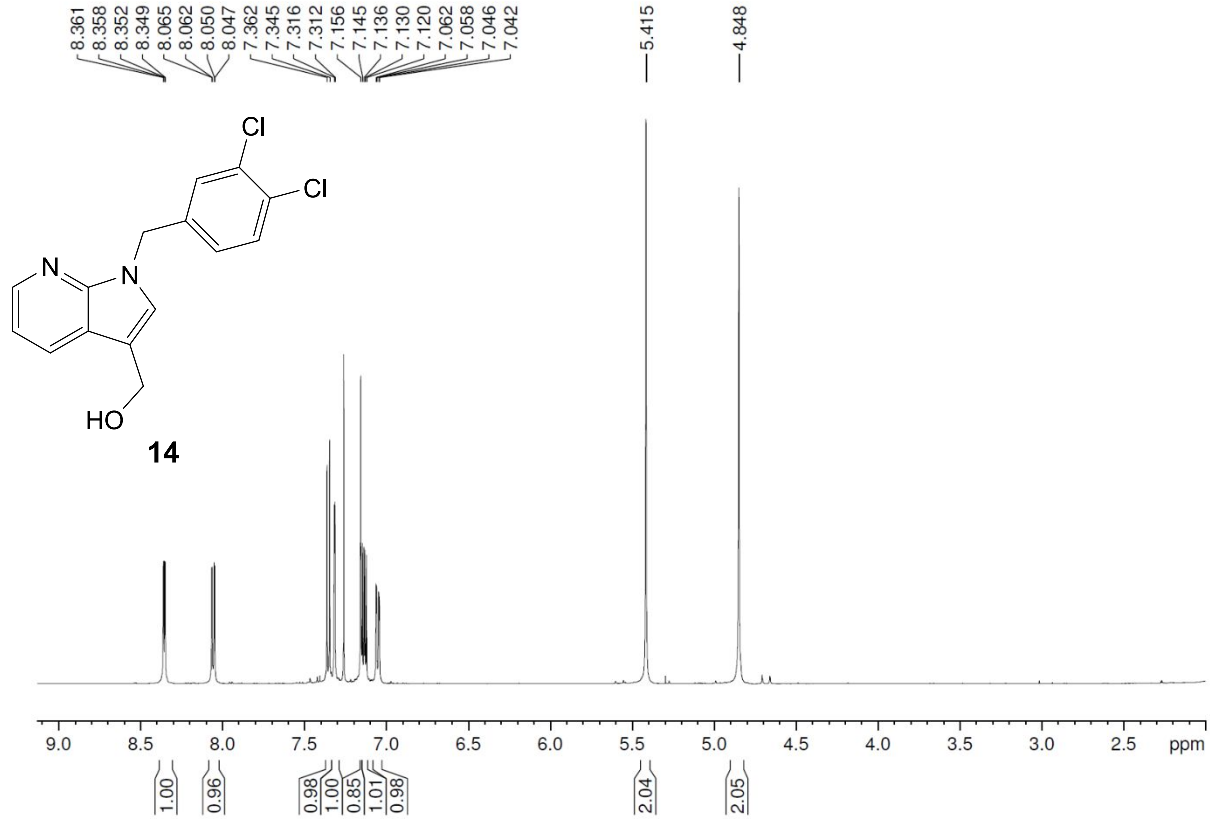

Spectrum 30: ^{1}H-NMR spectrum of indole **14** in $CDCl_3$.

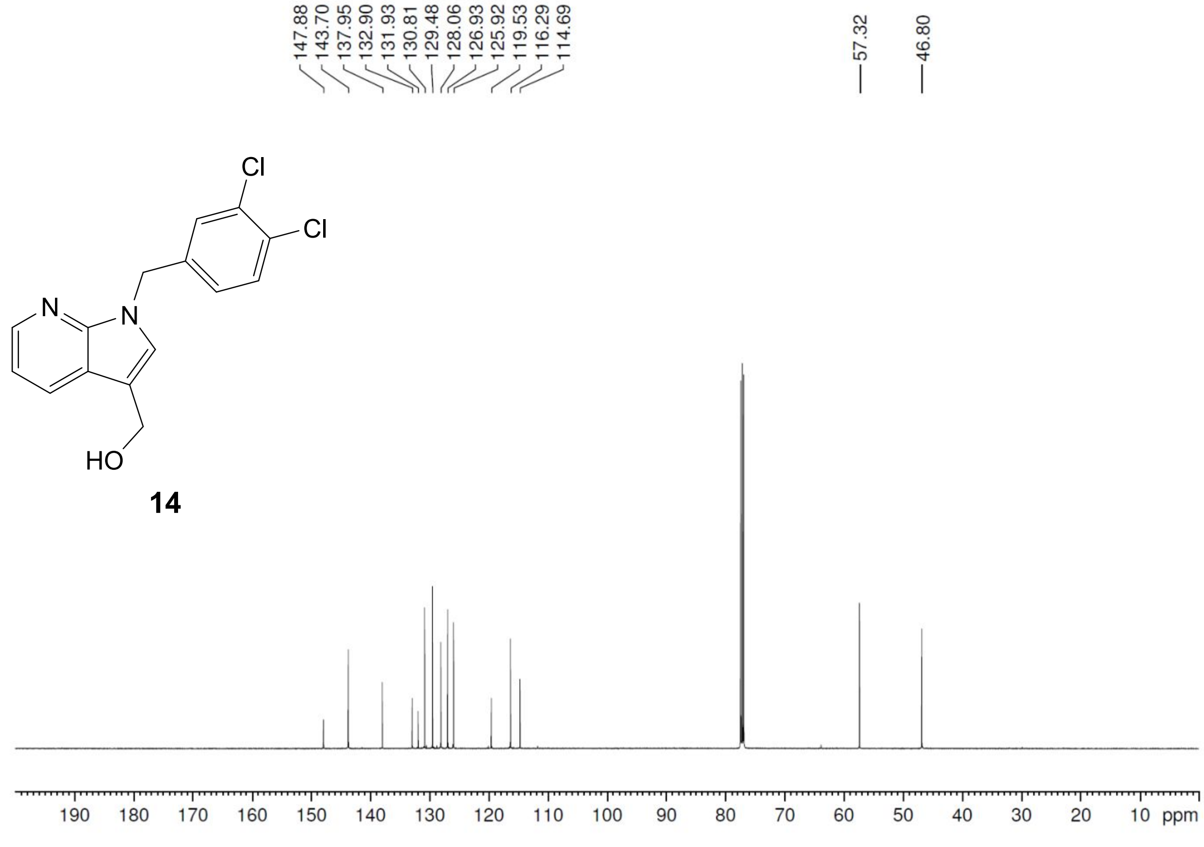

Spectrum 31: ^{13}C-NMR spectrum of indole **14** in $CDCl_3$.

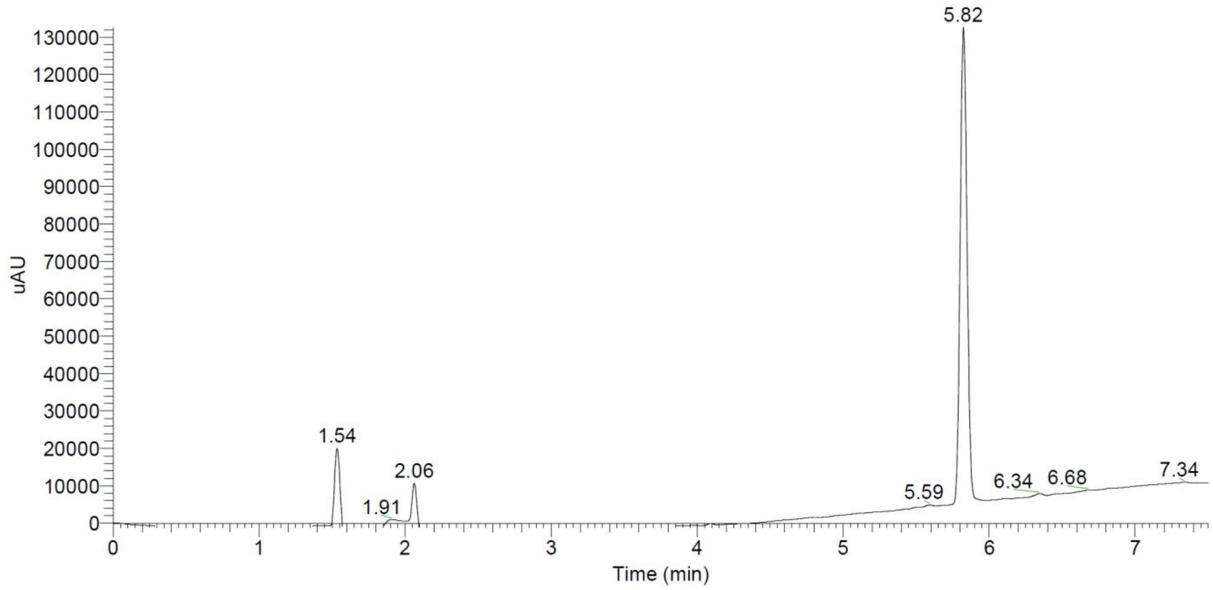

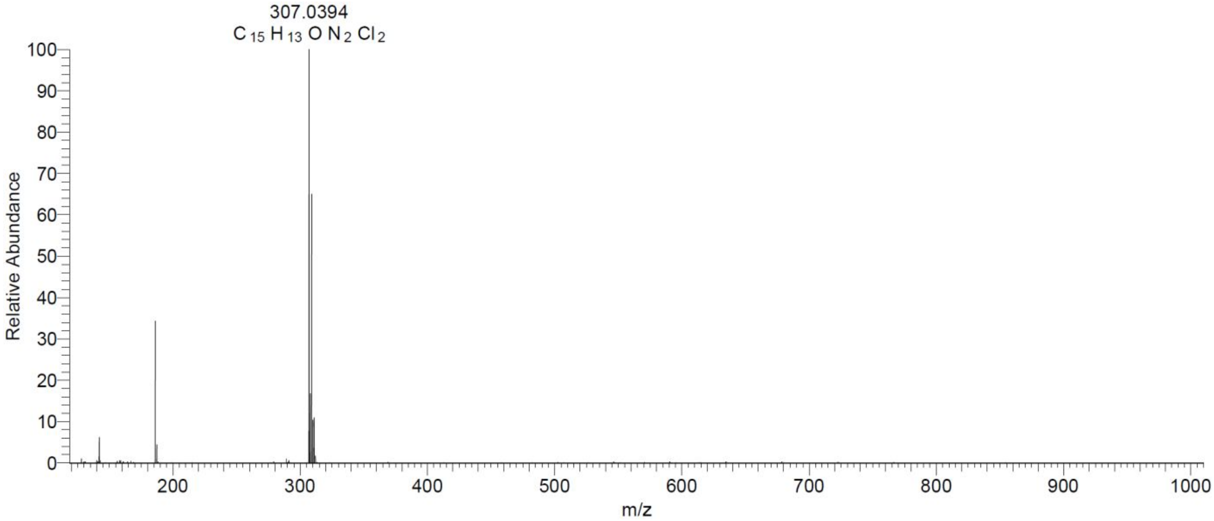

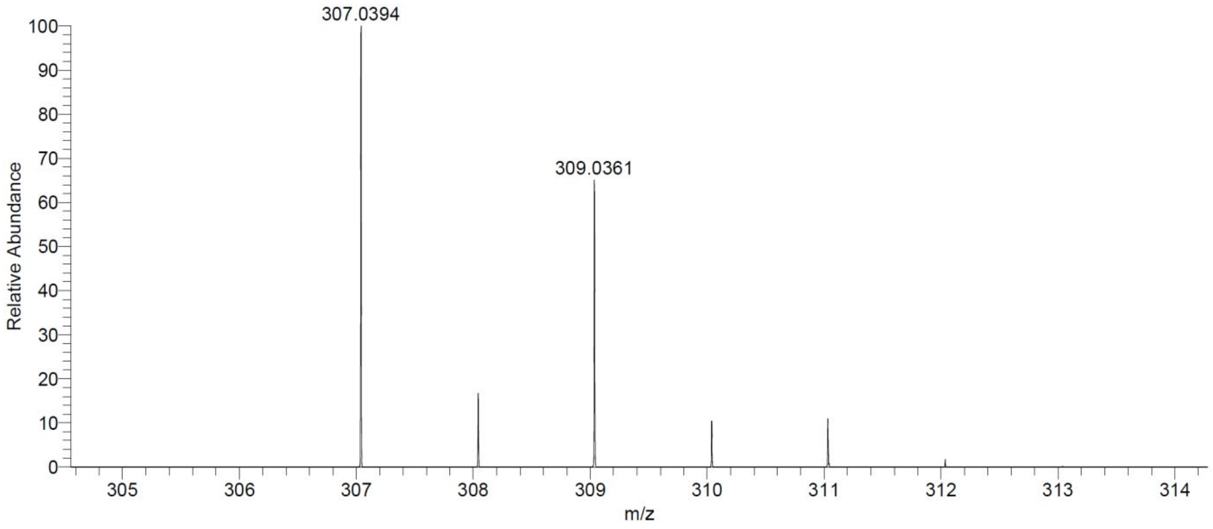

Spectrum 32: UV-trace and mass of main peak of indole **14**. Purity determined by peak area 98%.

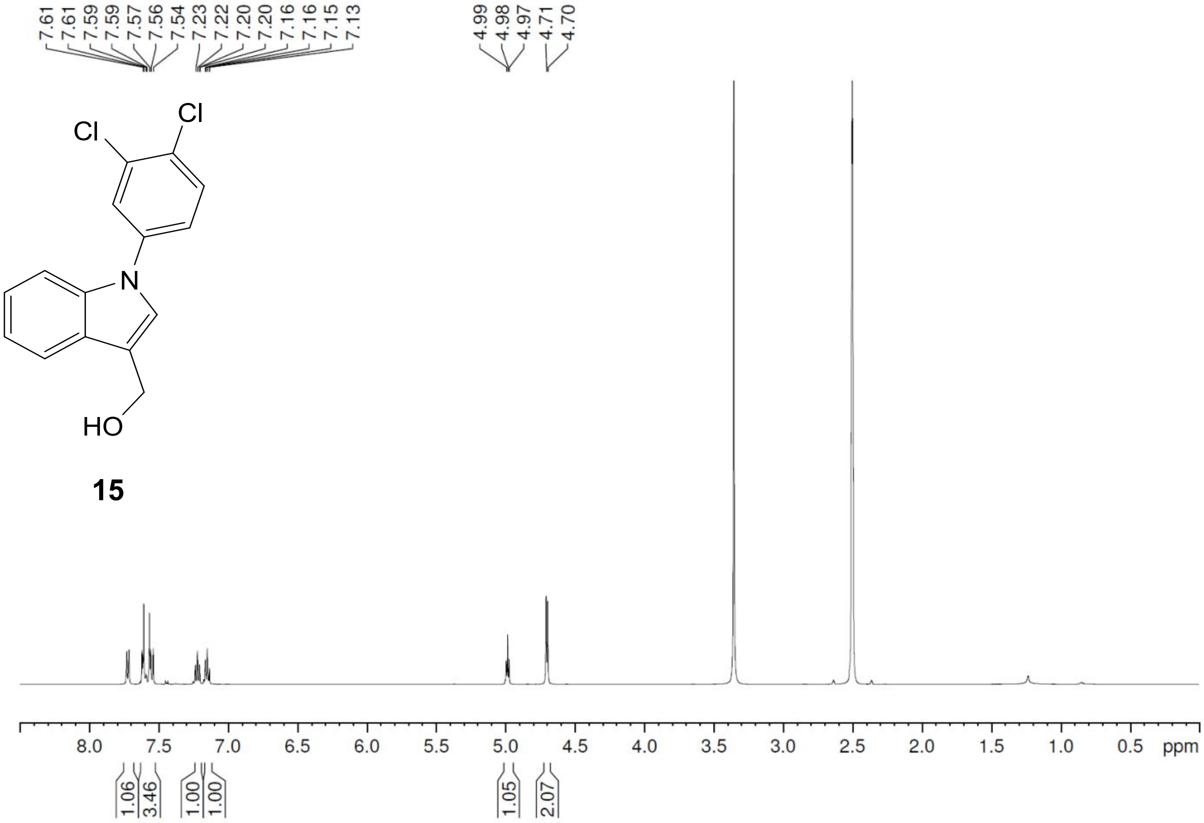

Spectrum 33: ^{1}H-NMR spectrum of indole **15** in $(CD_3)_2OS$.

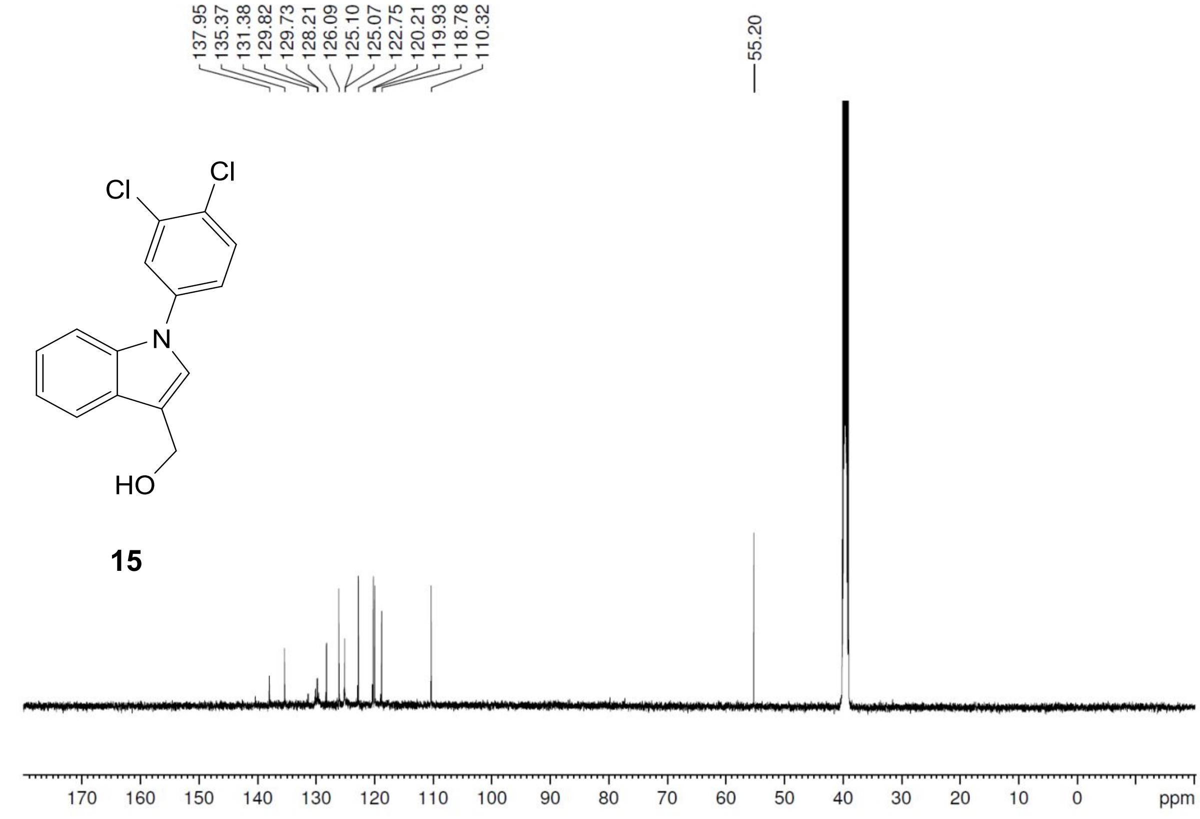

Spectrum 34: ^{13}C-NMR spectrum of indole **15** in $(CD_3)_2OS$.

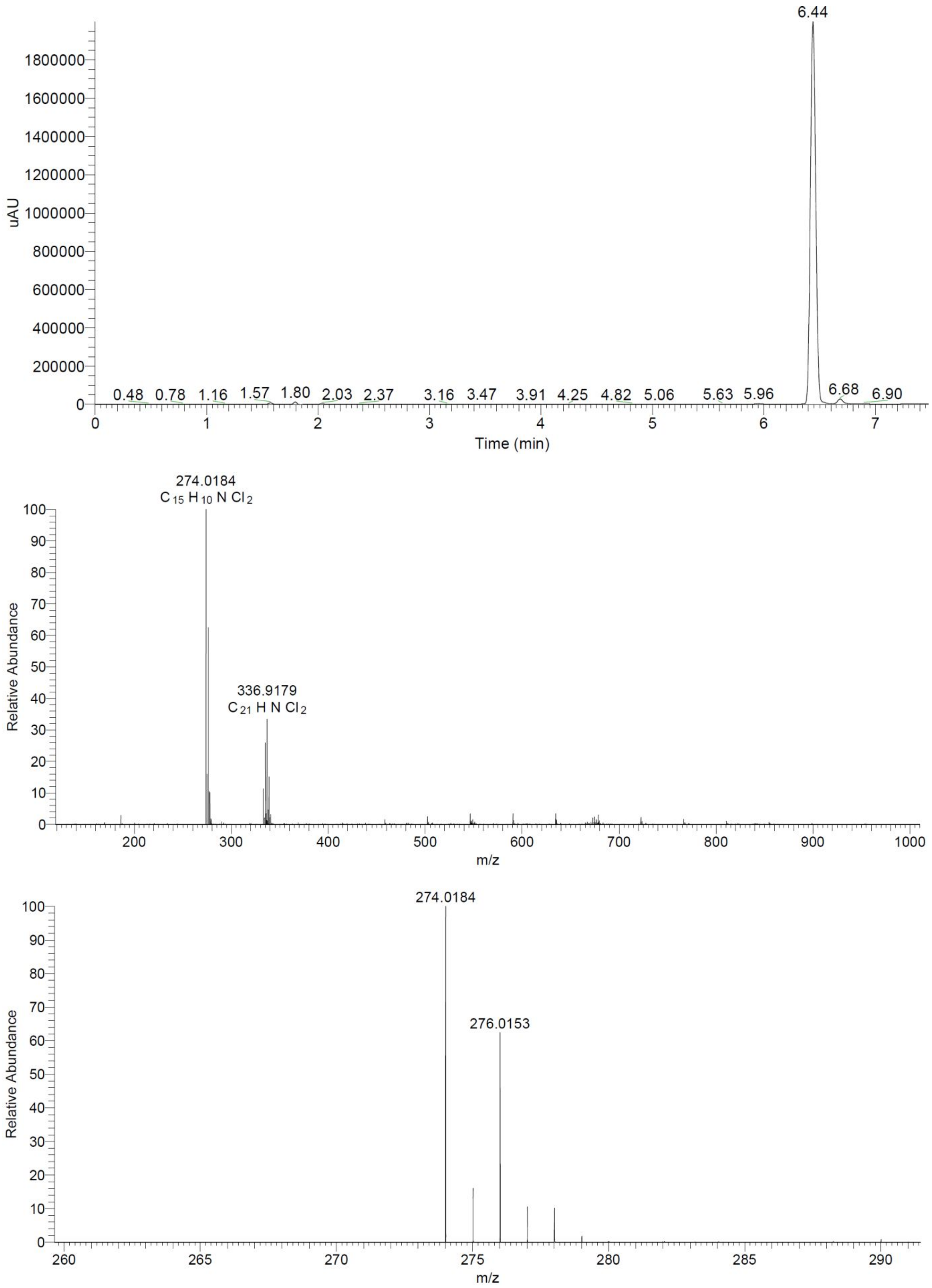

Spectrum 35: UV-trace and mass of main peak of indole **15**. Purity determined by peak area 97%.

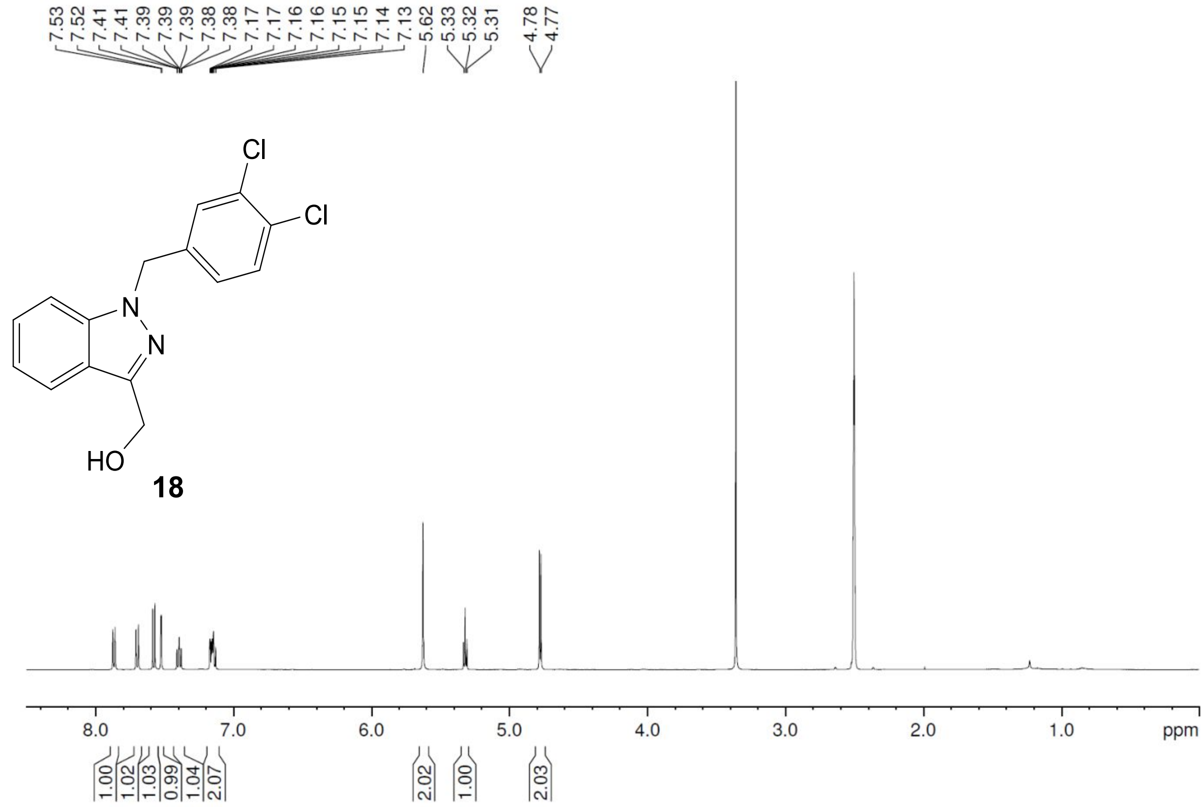

Spectrum 36: ^{1}H-NMR spectrum of indazole **18** in $(CD_3)_2OS$.

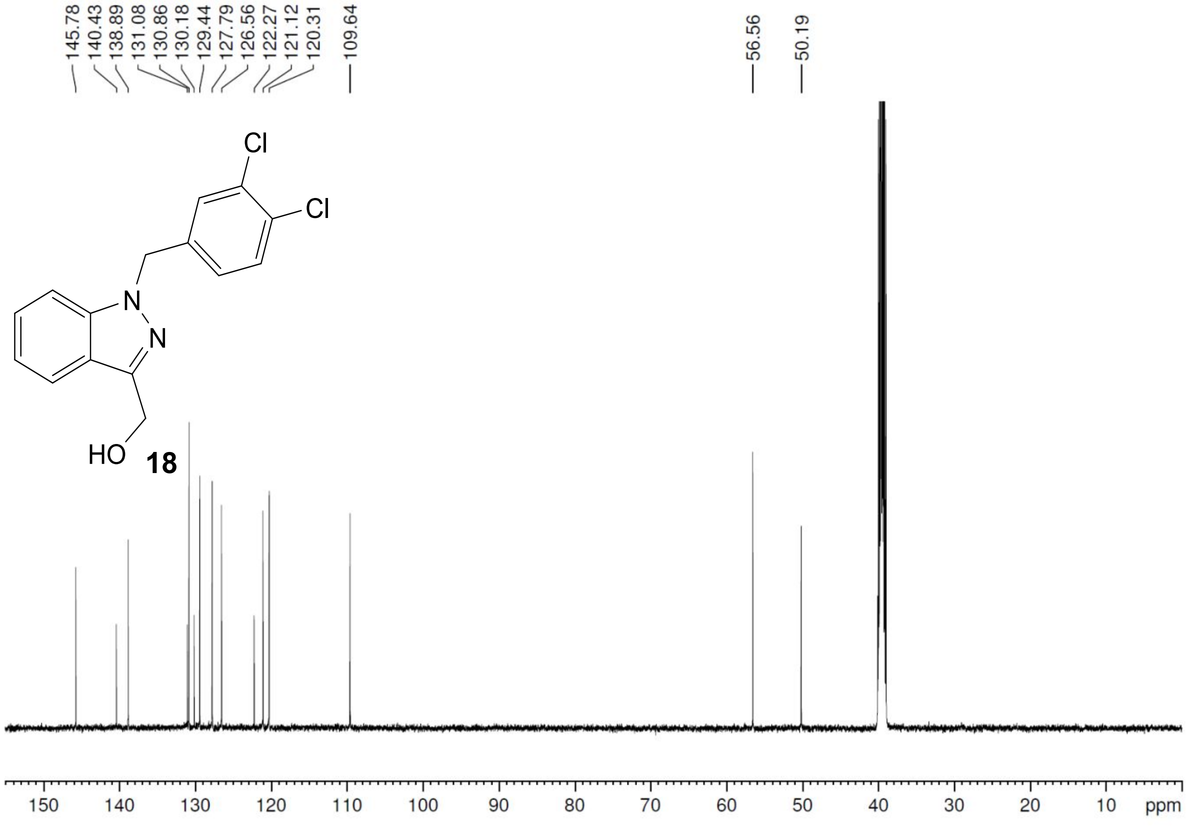

Spectrum 37: ^{13}C-NMR spectrum of indazole **18** in $(CD_3)_2OS$.

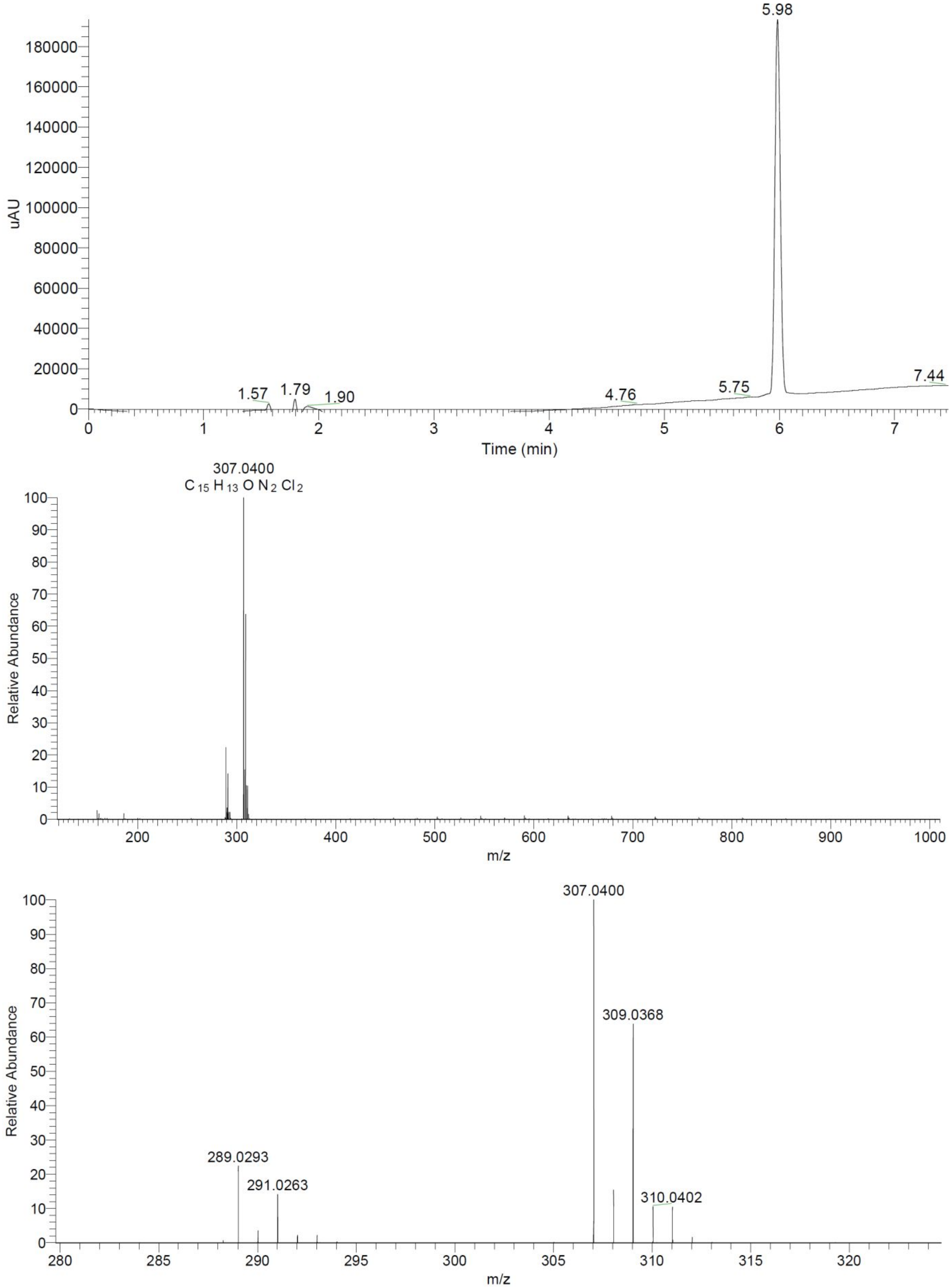

Spectrum 38: UV-trace and mass of main peak of indazole **18**. Purity determined by peak area 98%.

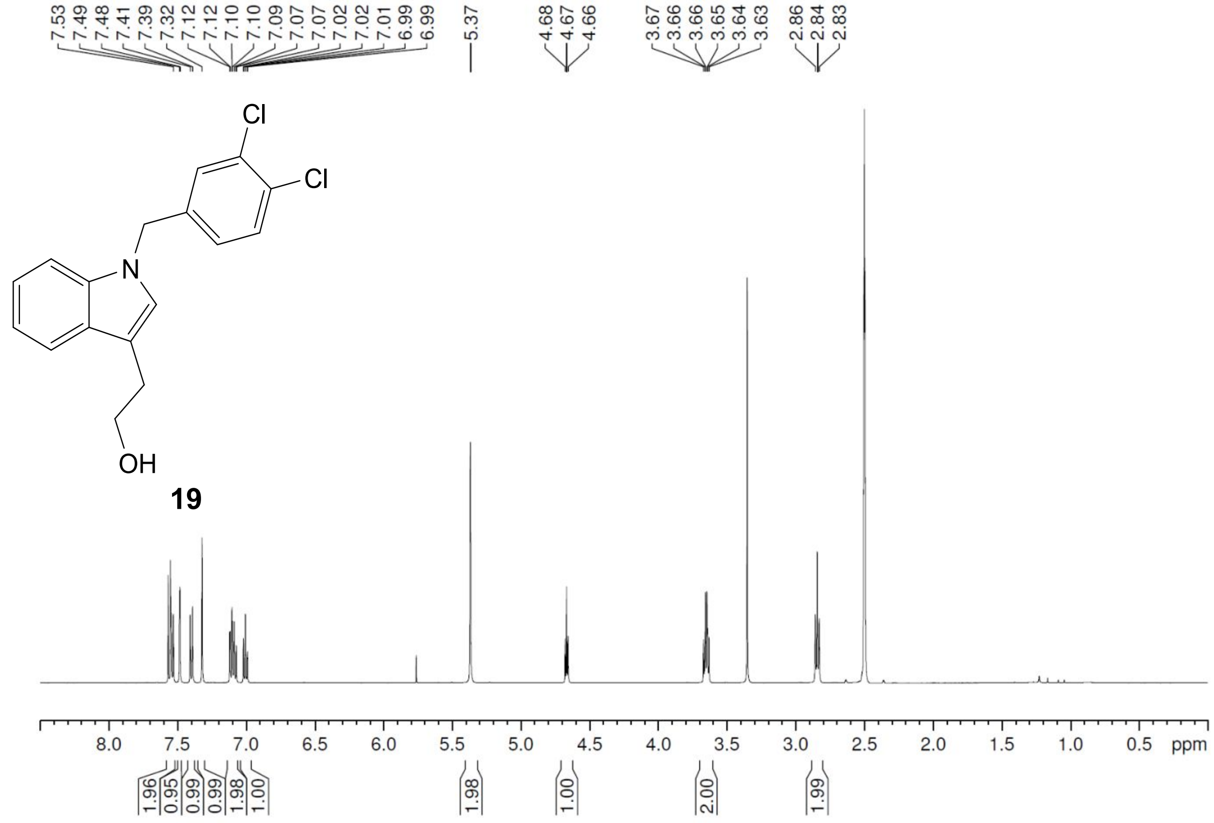

Spectrum 39: ^{1}H-NMR spectrum of indole **19** in $(CD_3)_2OS$.

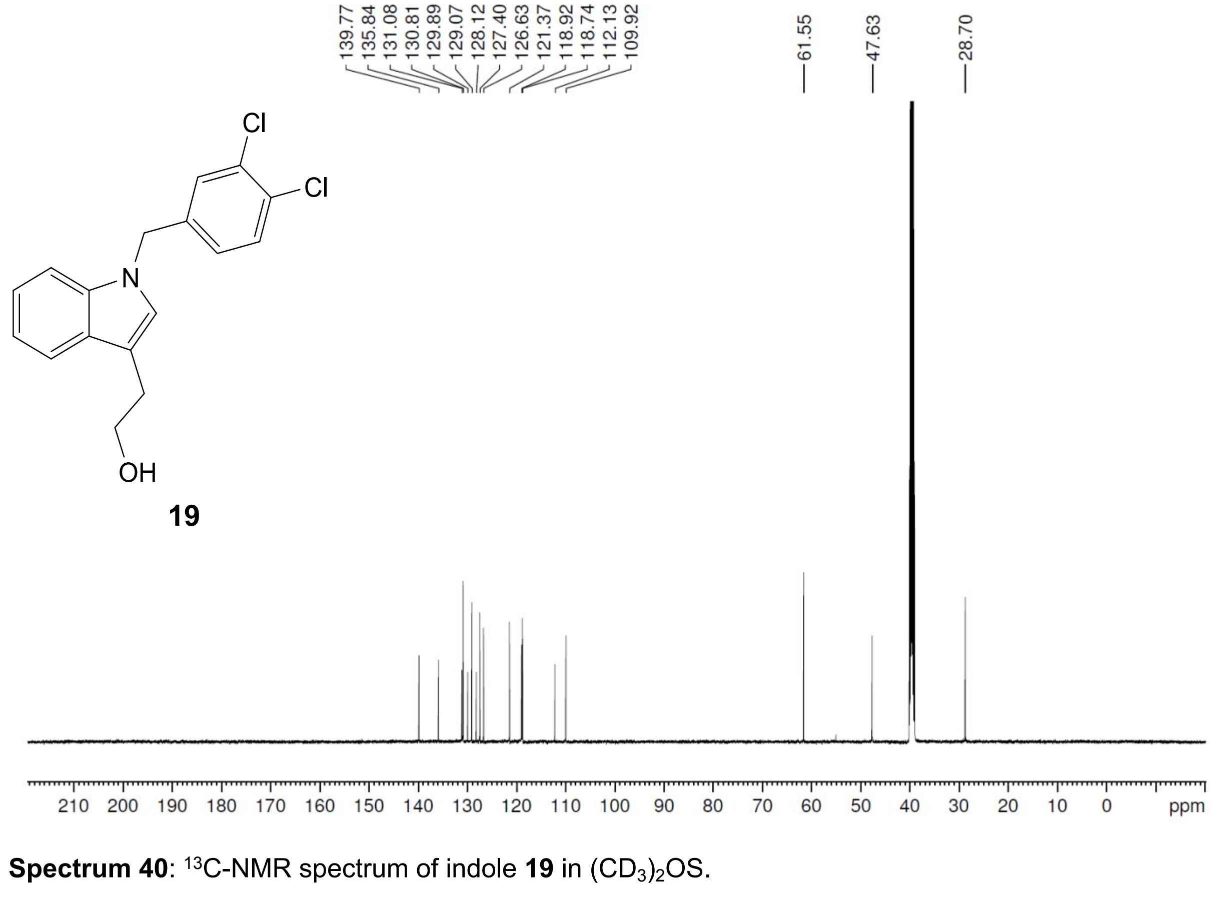

Spectrum 40: ^{13}C-NMR spectrum of indole **19** in $(CD_3)_2OS$.

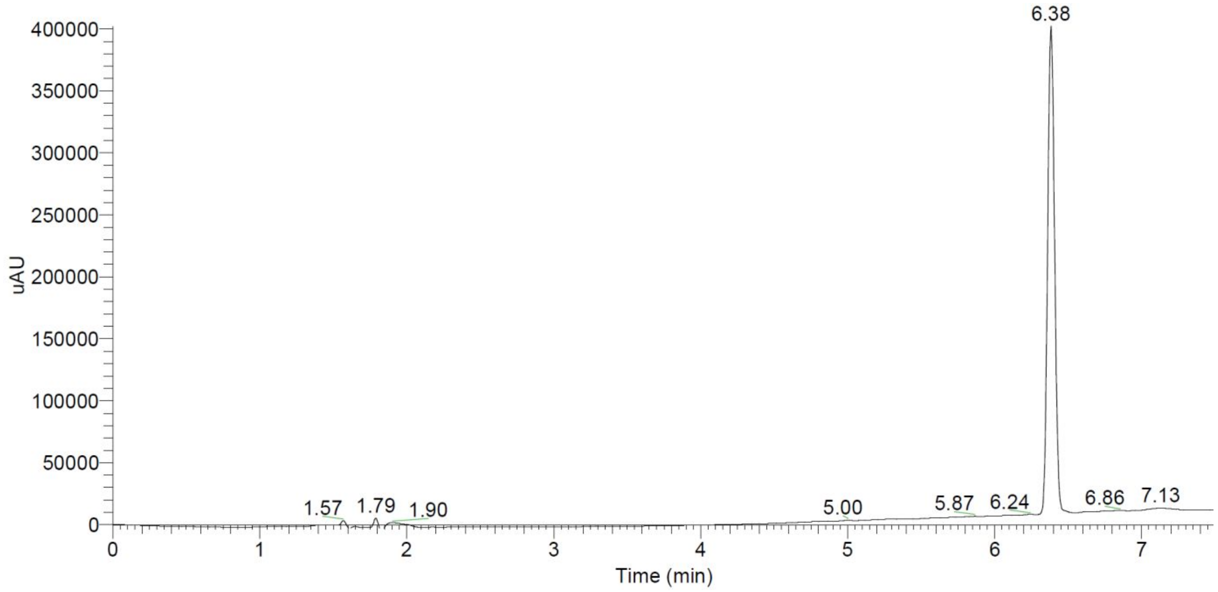

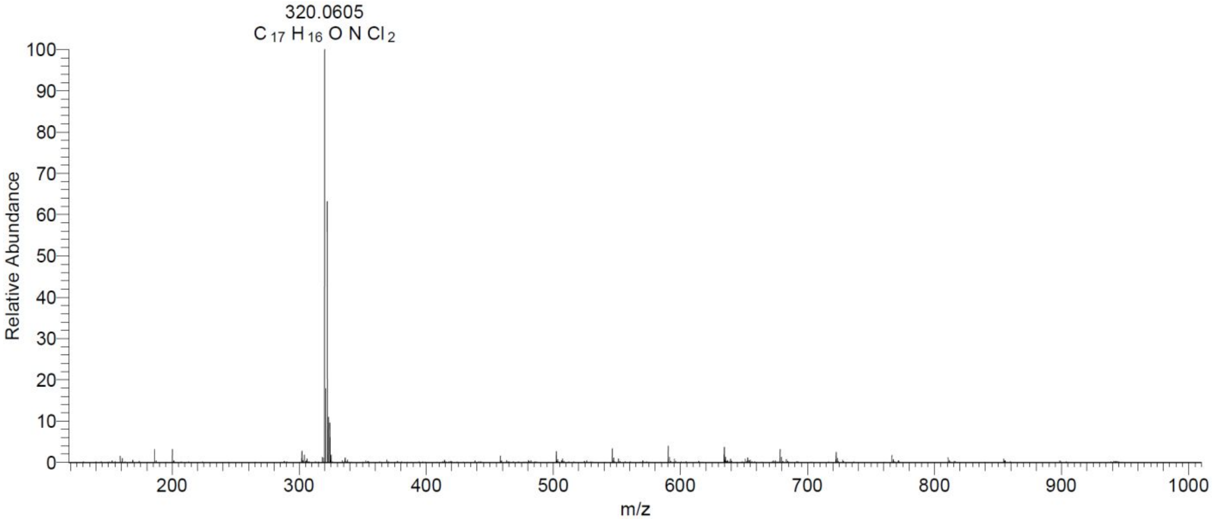

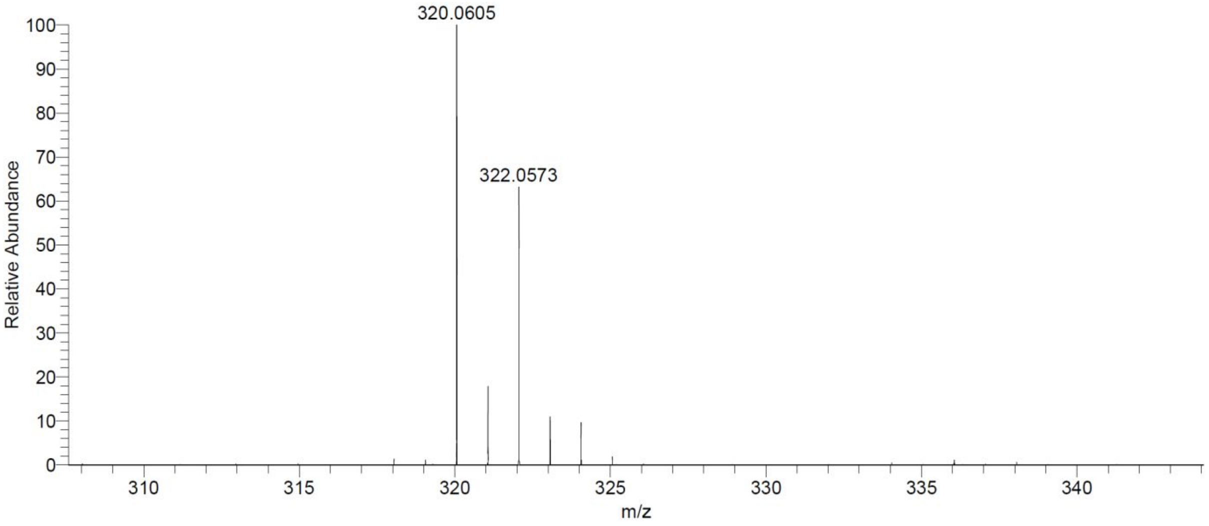

Spectrum 41: UV-trace and mass of main peak of indole **19**. Purity determined by peak area 98%.

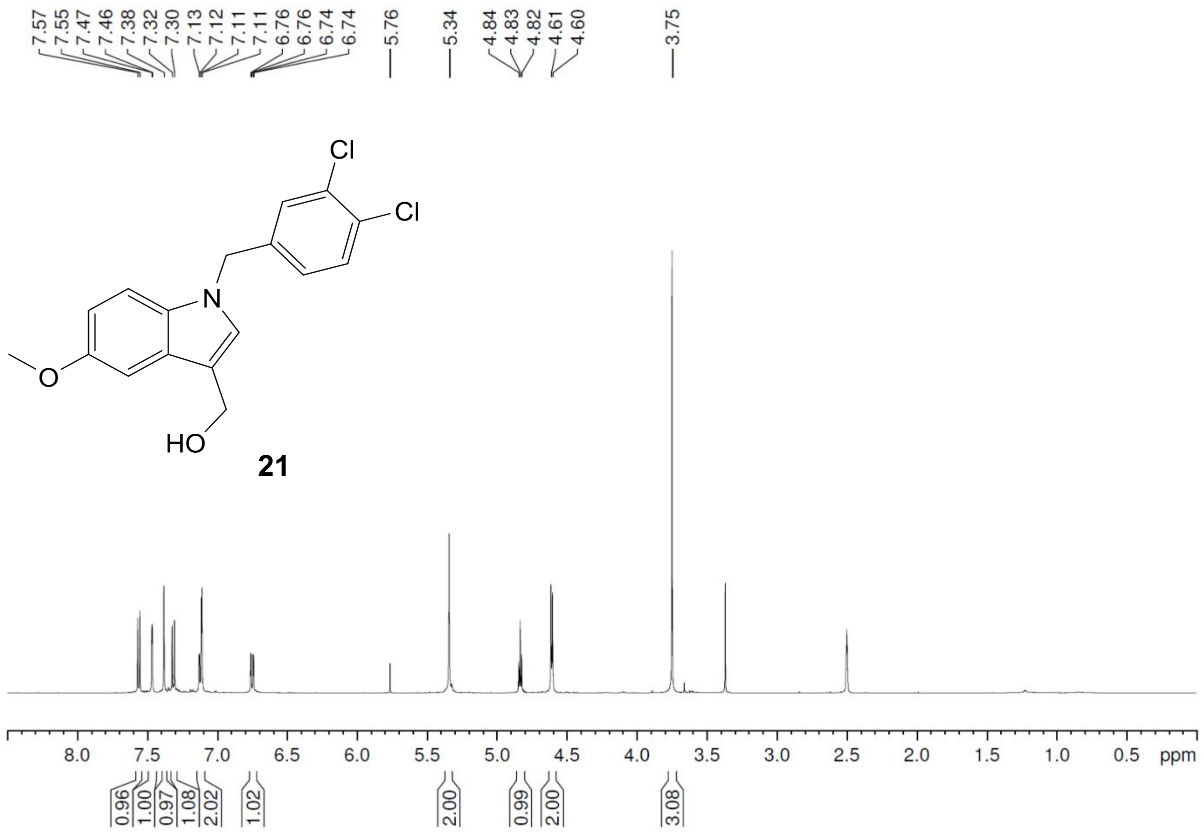

Spectrum 42: ^{1}H-NMR spectrum of indole **21** in $(CD_3)_2OS$.

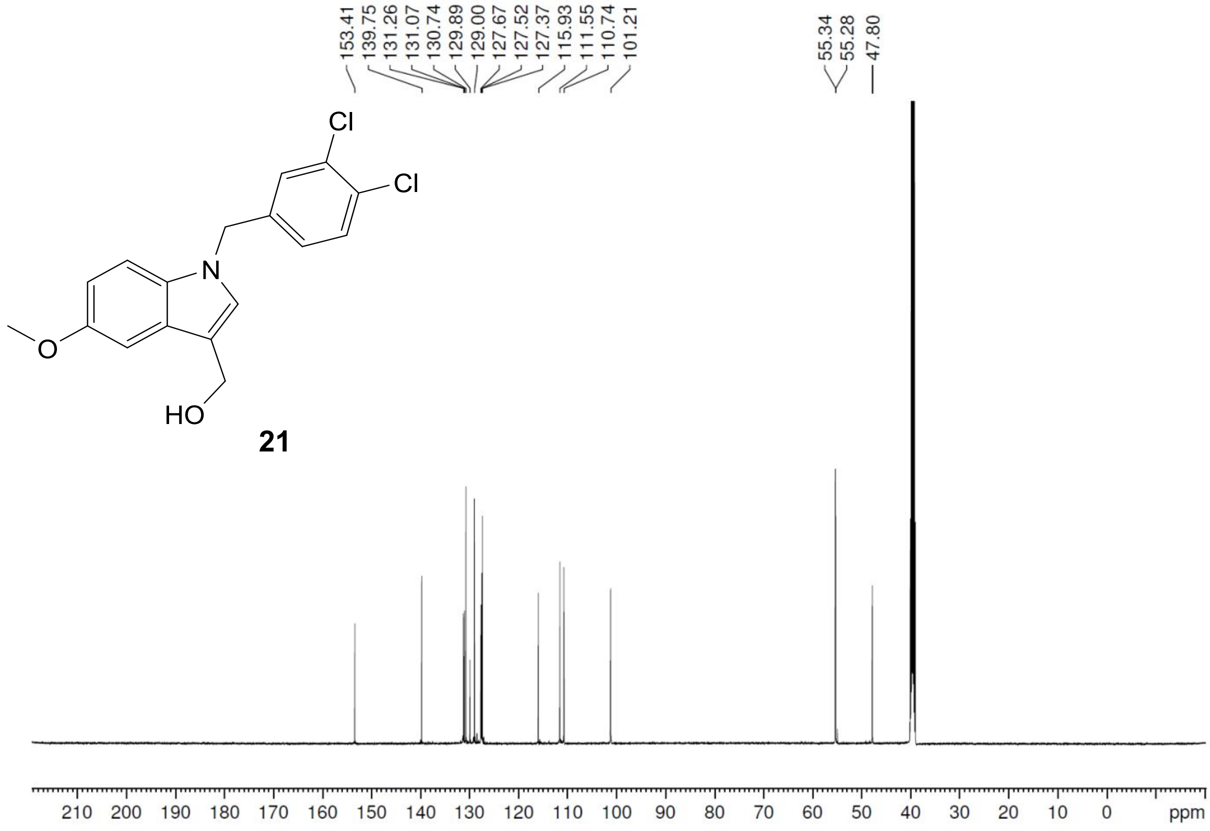

Spectrum 43: ^{13}C-NMR spectrum of indole **21** in $(CD_3)_2OS$.

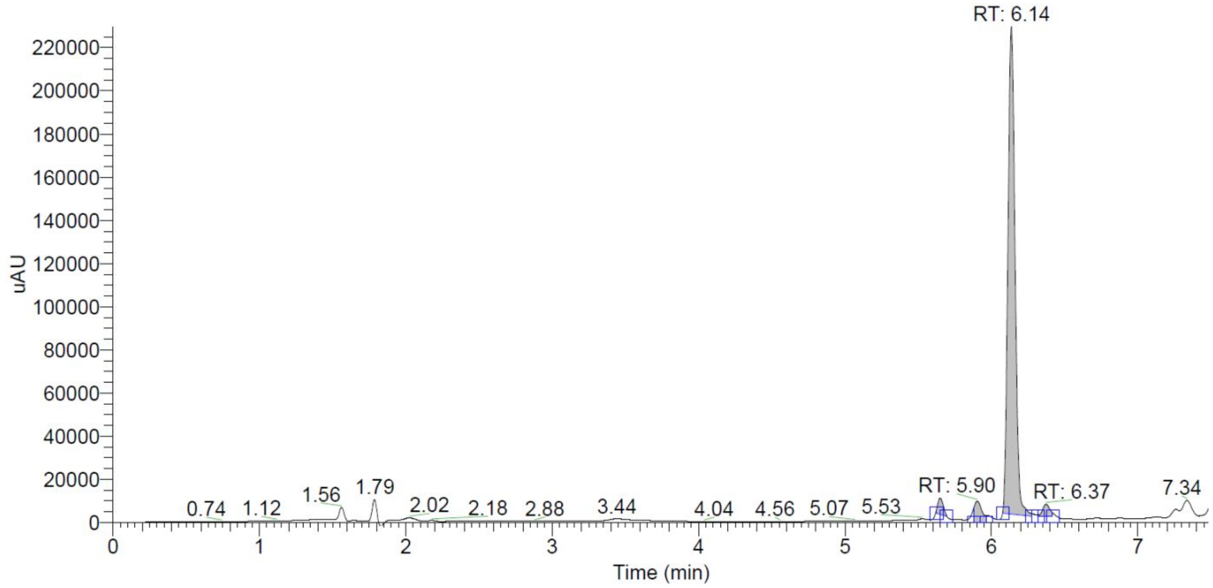

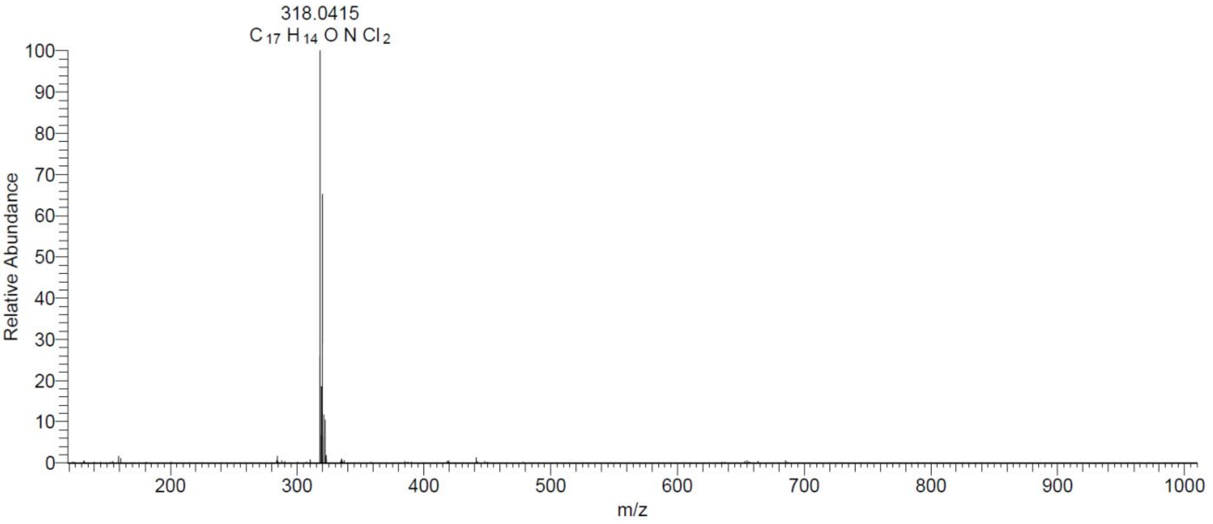

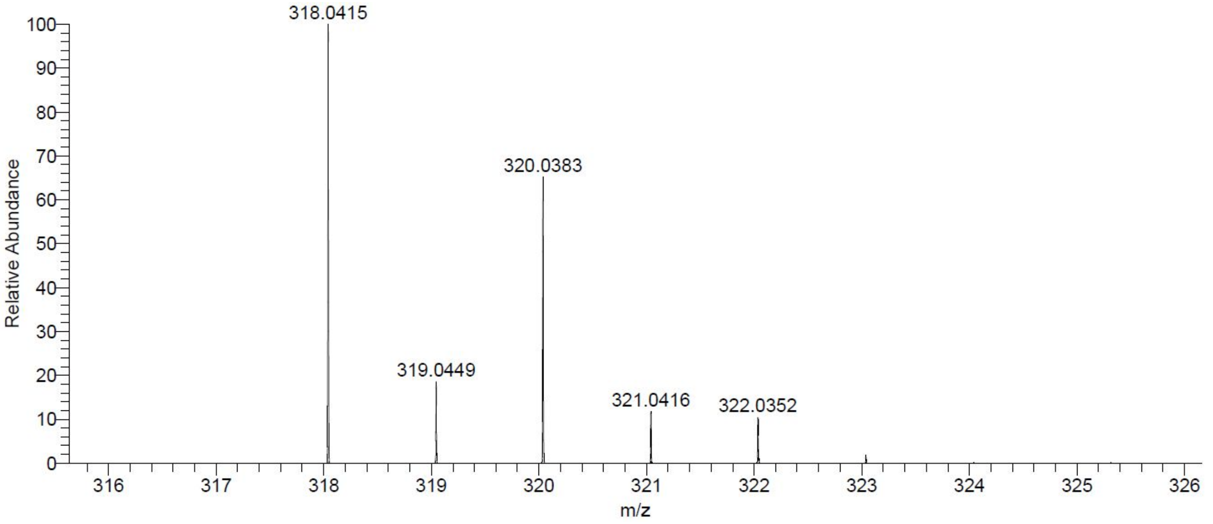

Spectrum 44: UV-trace and mass of main peak of indole **21**. Purity determined by peak area 95%.

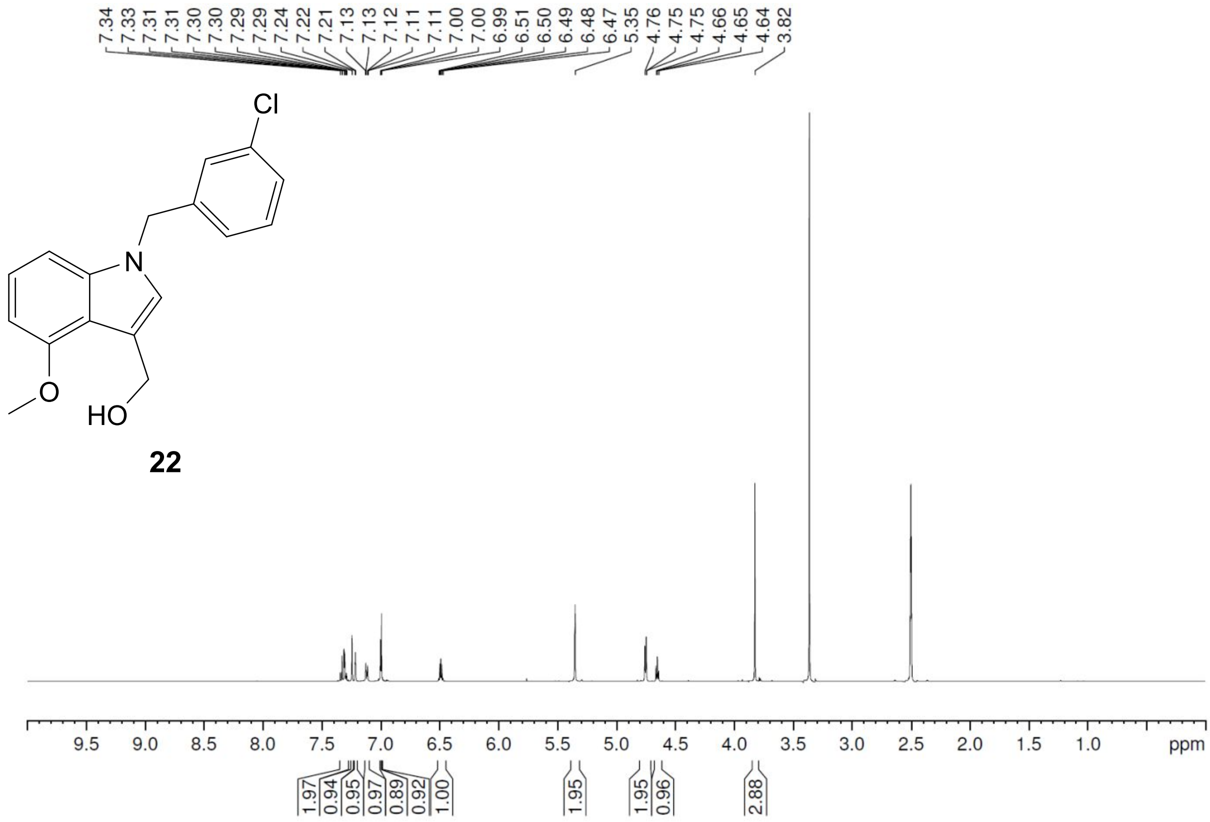

Spectrum 45: ^{1}H-NMR spectrum of indole **22** in $(CD_3)_2OS$.

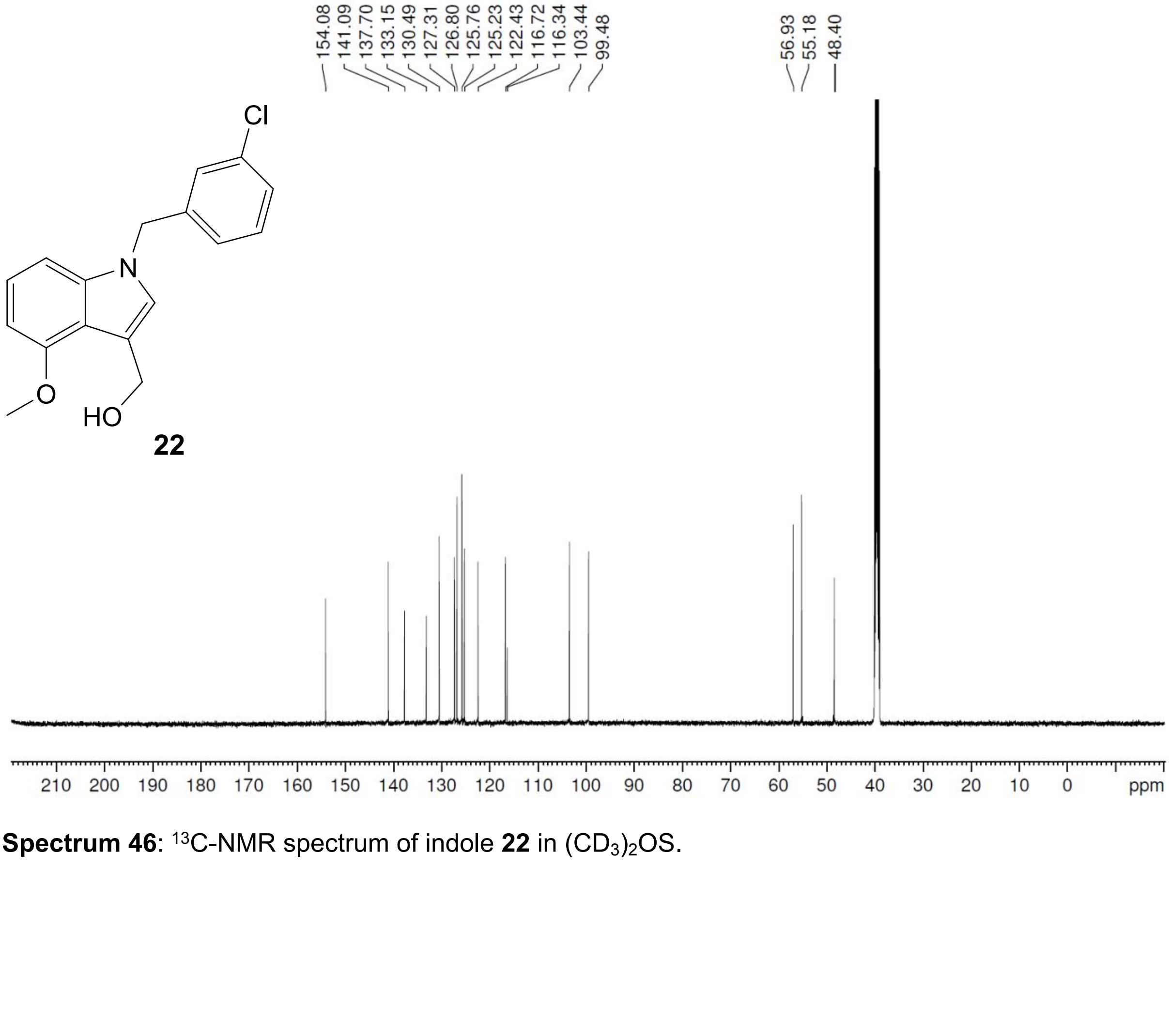

Spectrum 46: ^{13}C-NMR spectrum of indole **22** in $(CD_3)_2OS$.

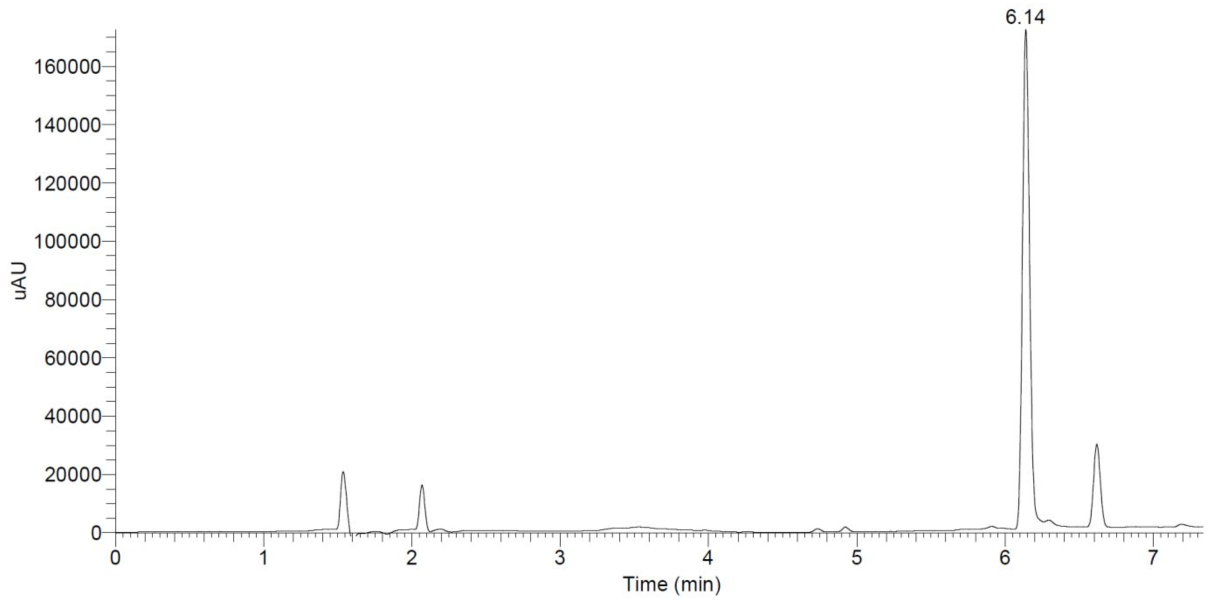

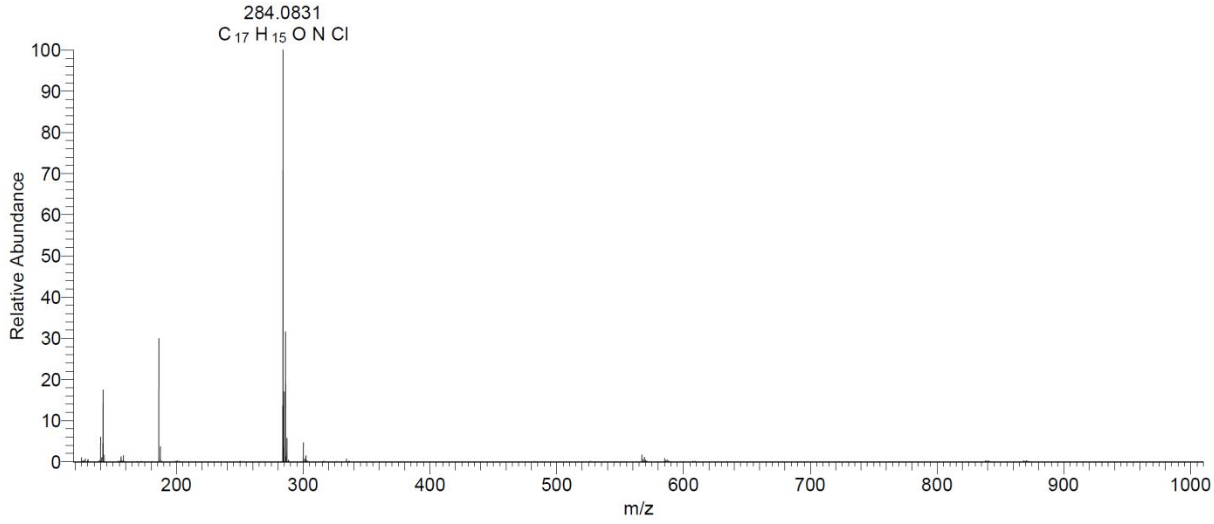

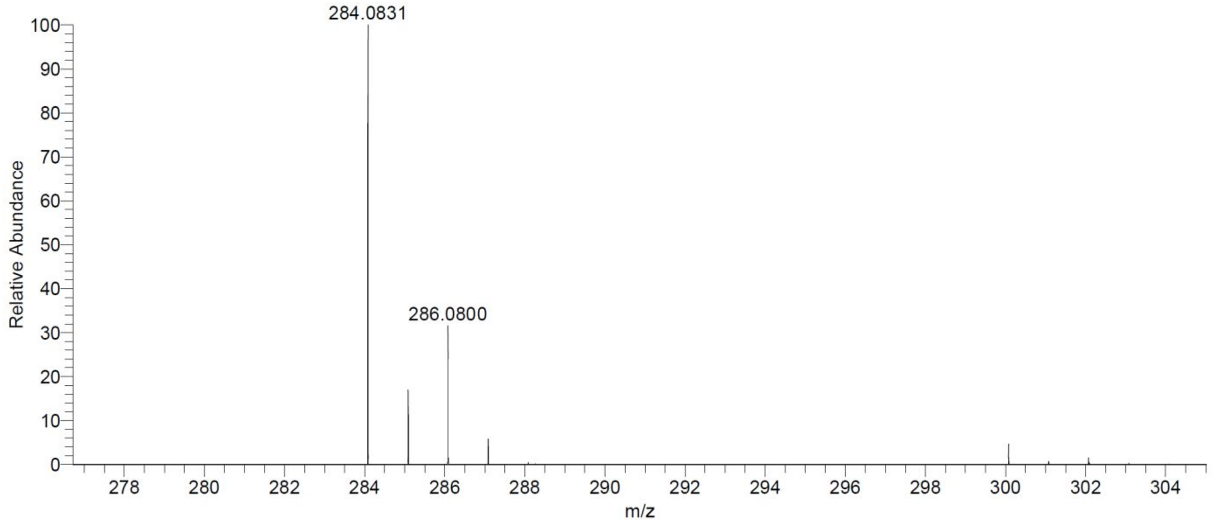

Spectrum 47: UV-trace and mass of main peak of indole **22**. Purity determined by peak area 95%. Both main peaks show the same mass.

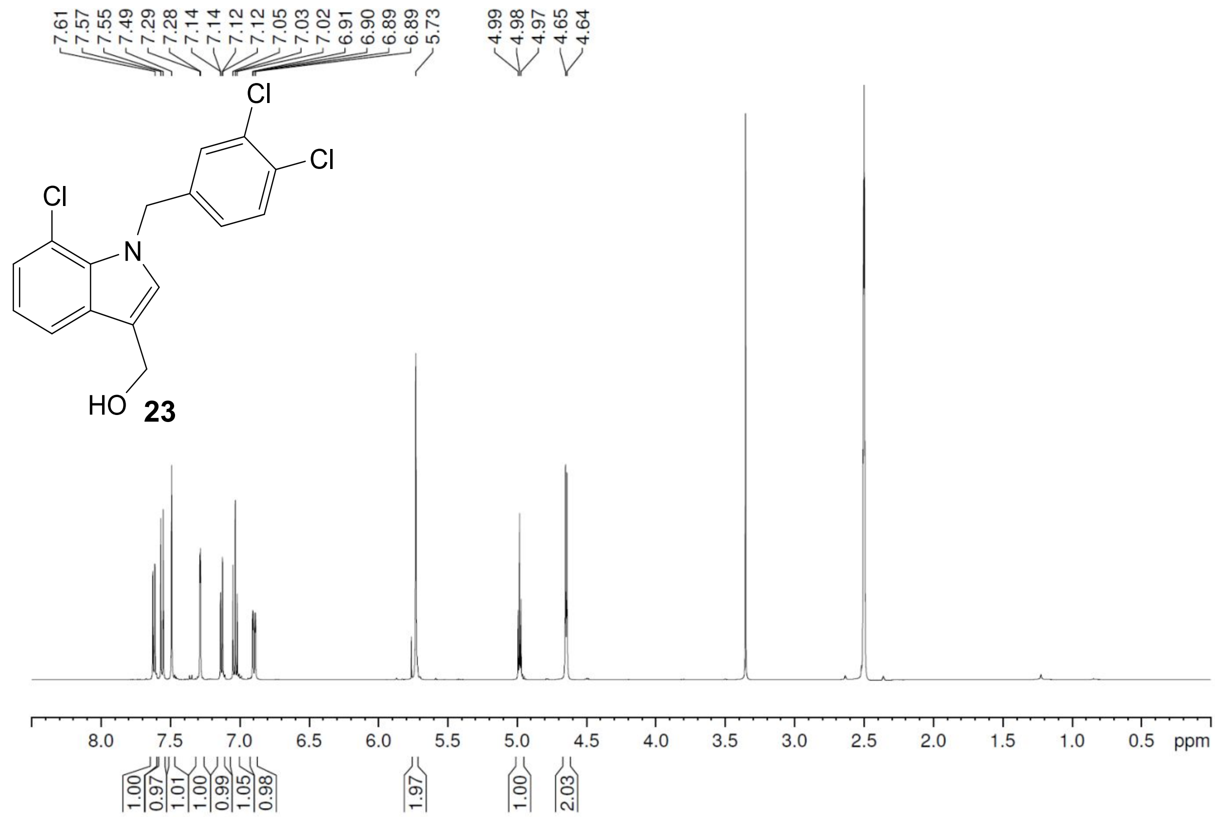

Spectrum 48: ^{1}H-NMR spectrum of indole **23** in $(CD_3)_2OS$.

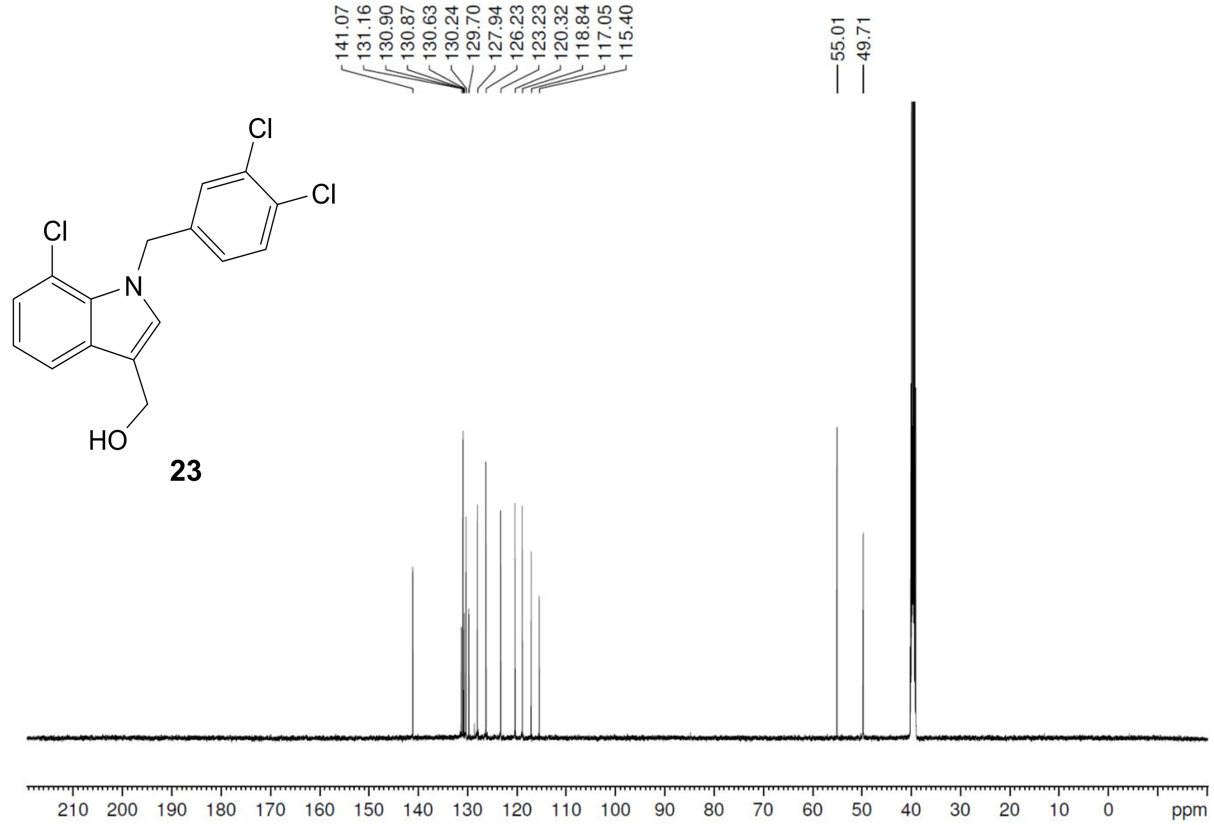

Spectrum 49: ^{13}C-NMR spectrum of indole **23** in $(CD_3)_2OS$.

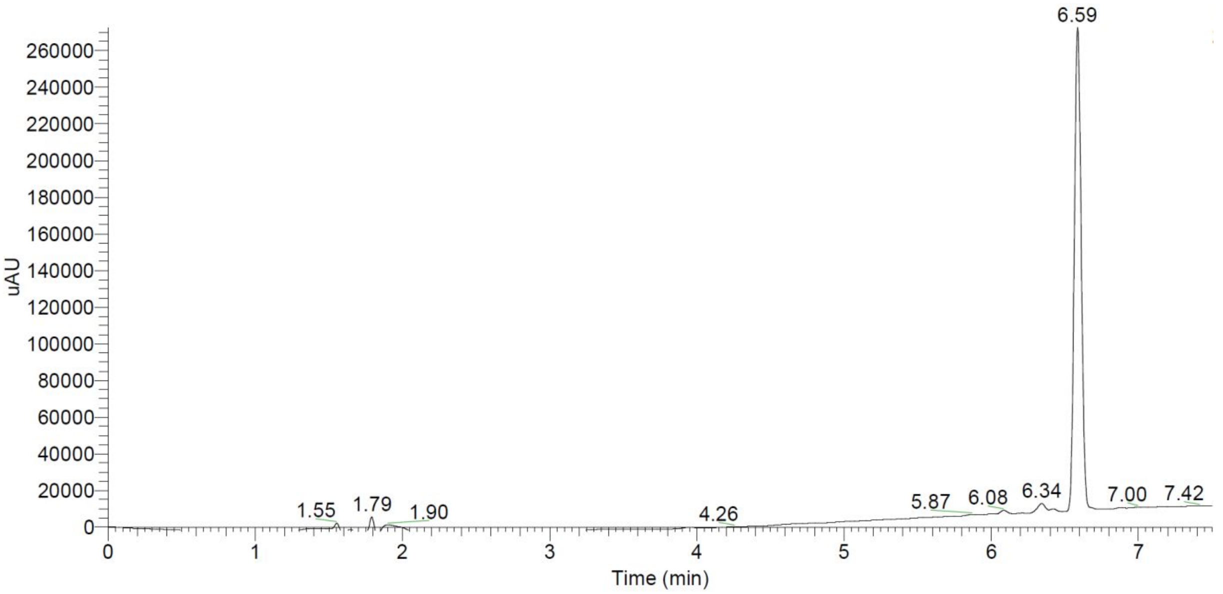

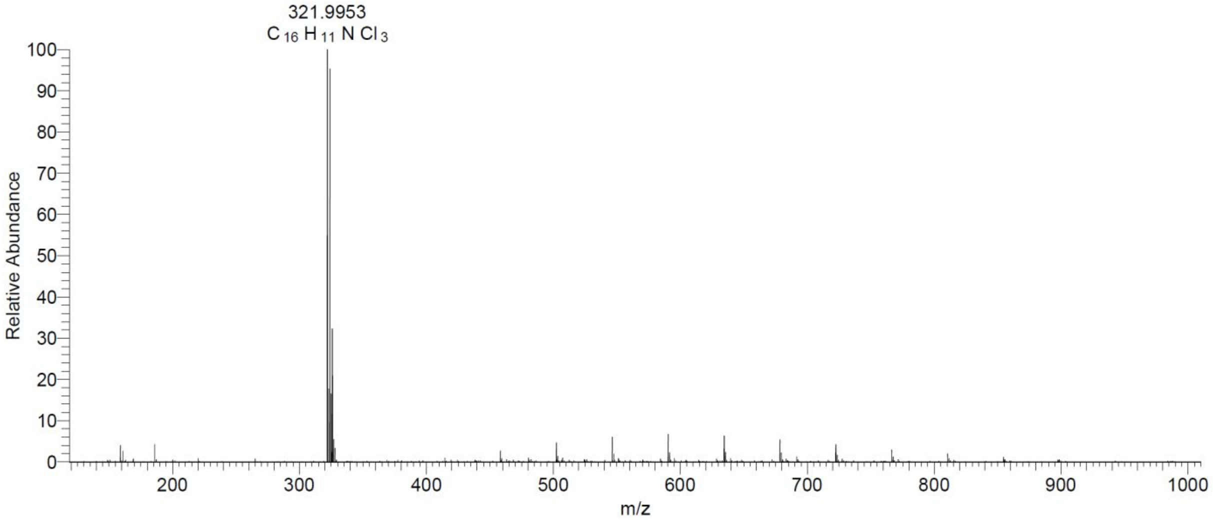

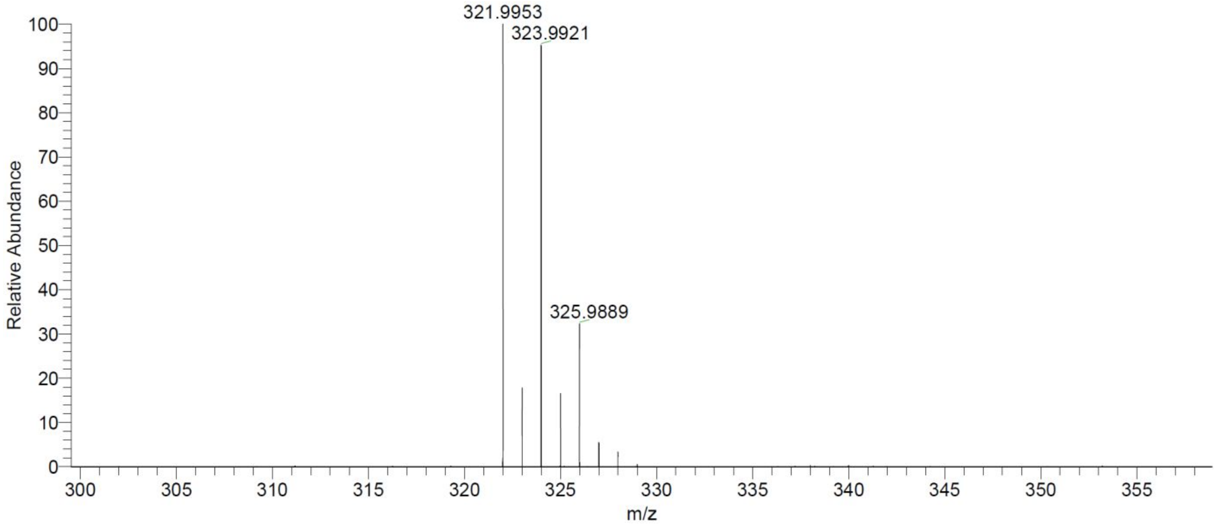

Spectrum 50: UV-trace and mass of main peak of indole **23**. Purity determined by peak area 98%.

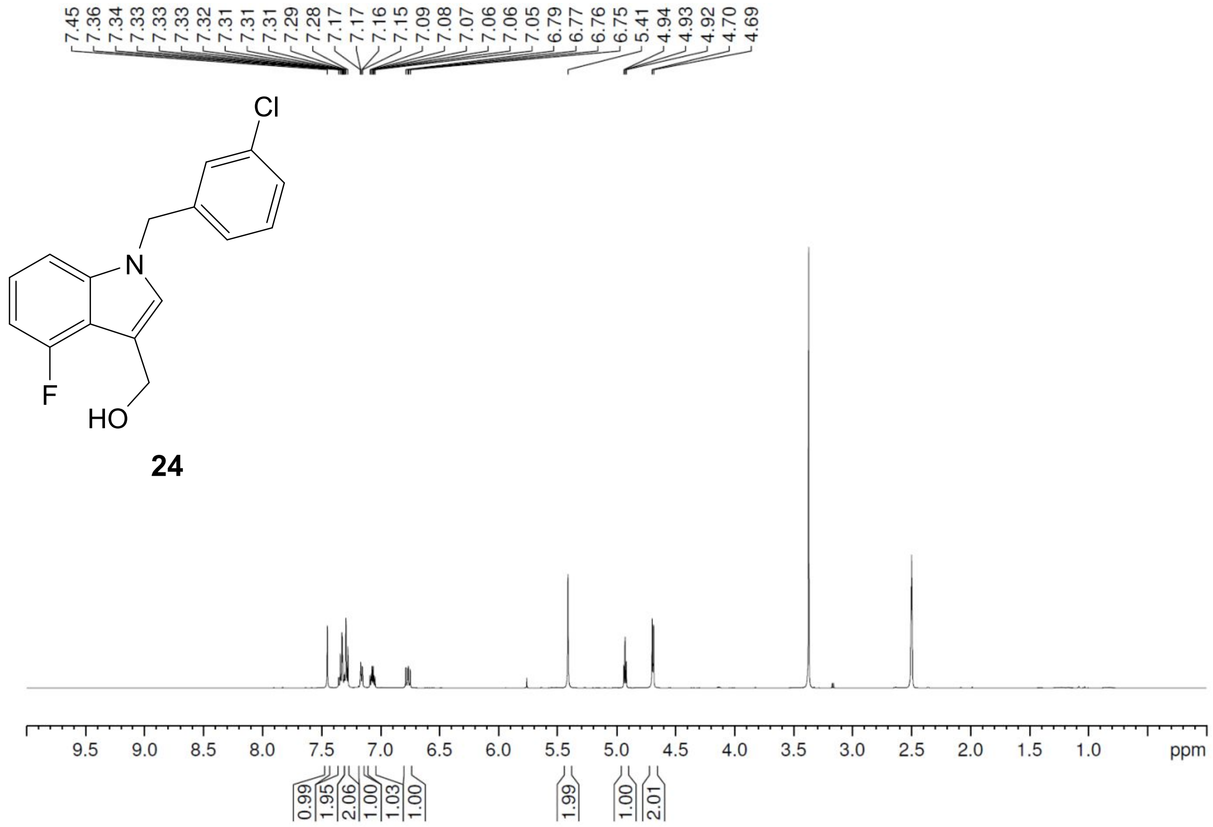

Spectrum 51: ^{1}H-NMR spectrum of indole **24** in $(CD_3)_2OS$.

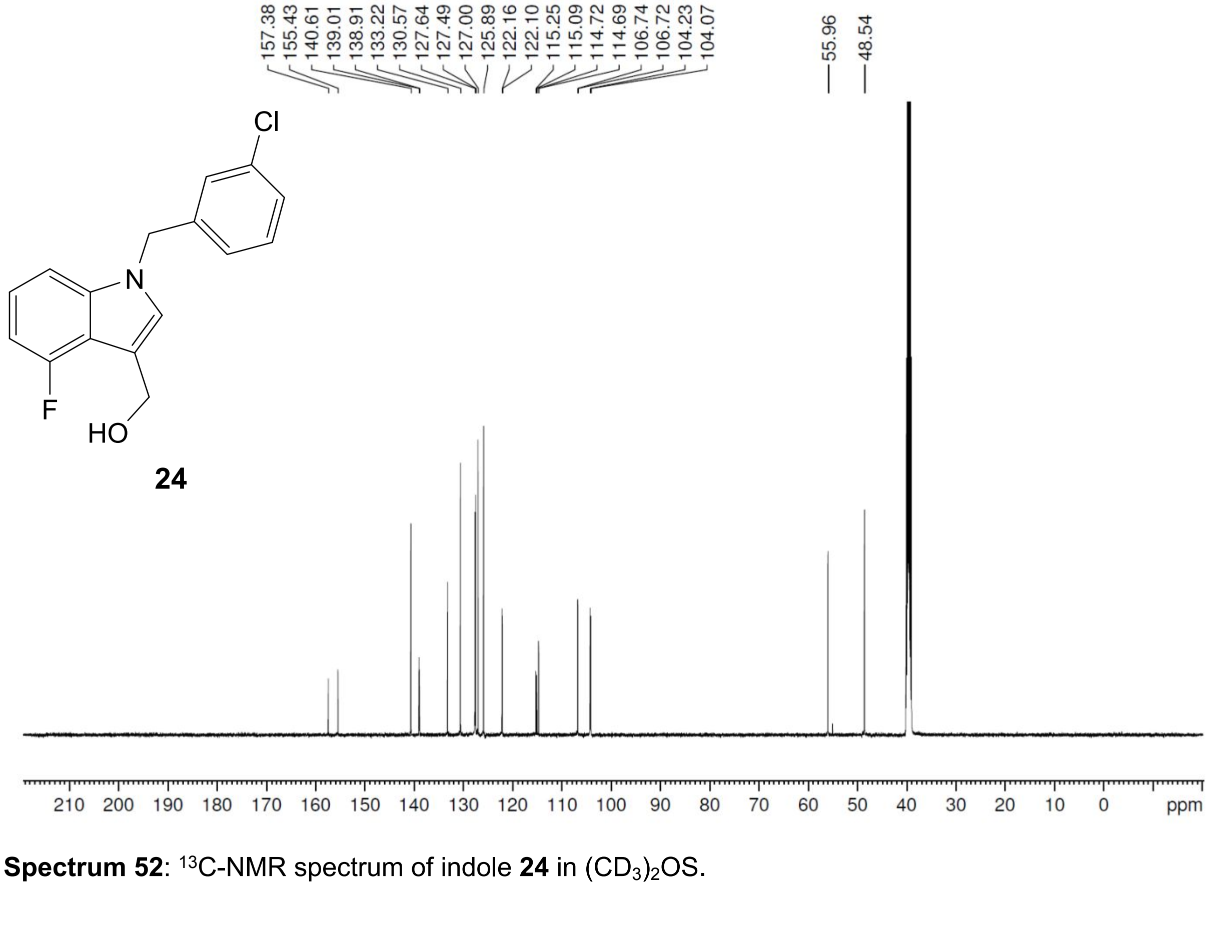

Spectrum 52: ^{13}C-NMR spectrum of indole **24** in $(CD_3)_2OS$.

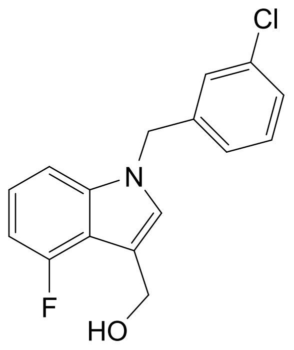

24

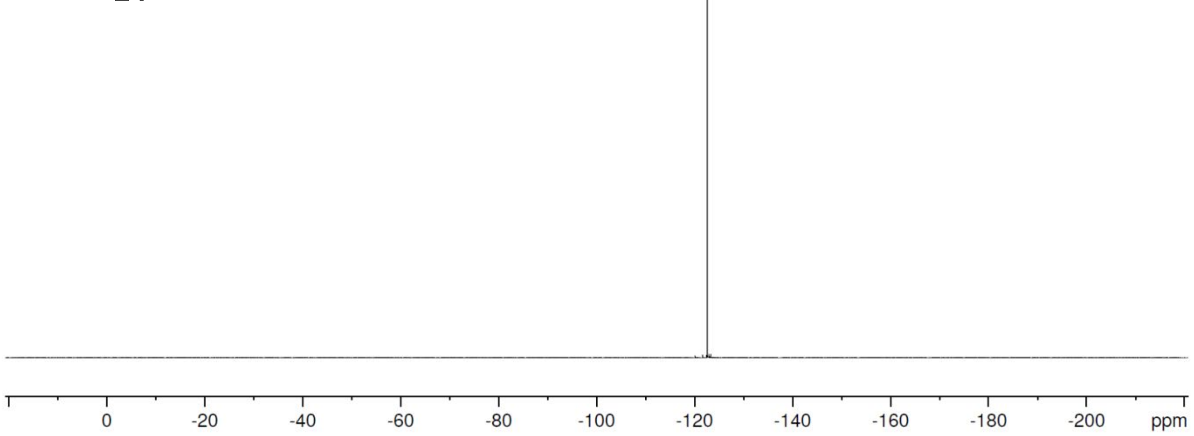

Spectrum 53: ^{19}F-NMR spectrum of indole **24** in $(CD_3)_2OS$.

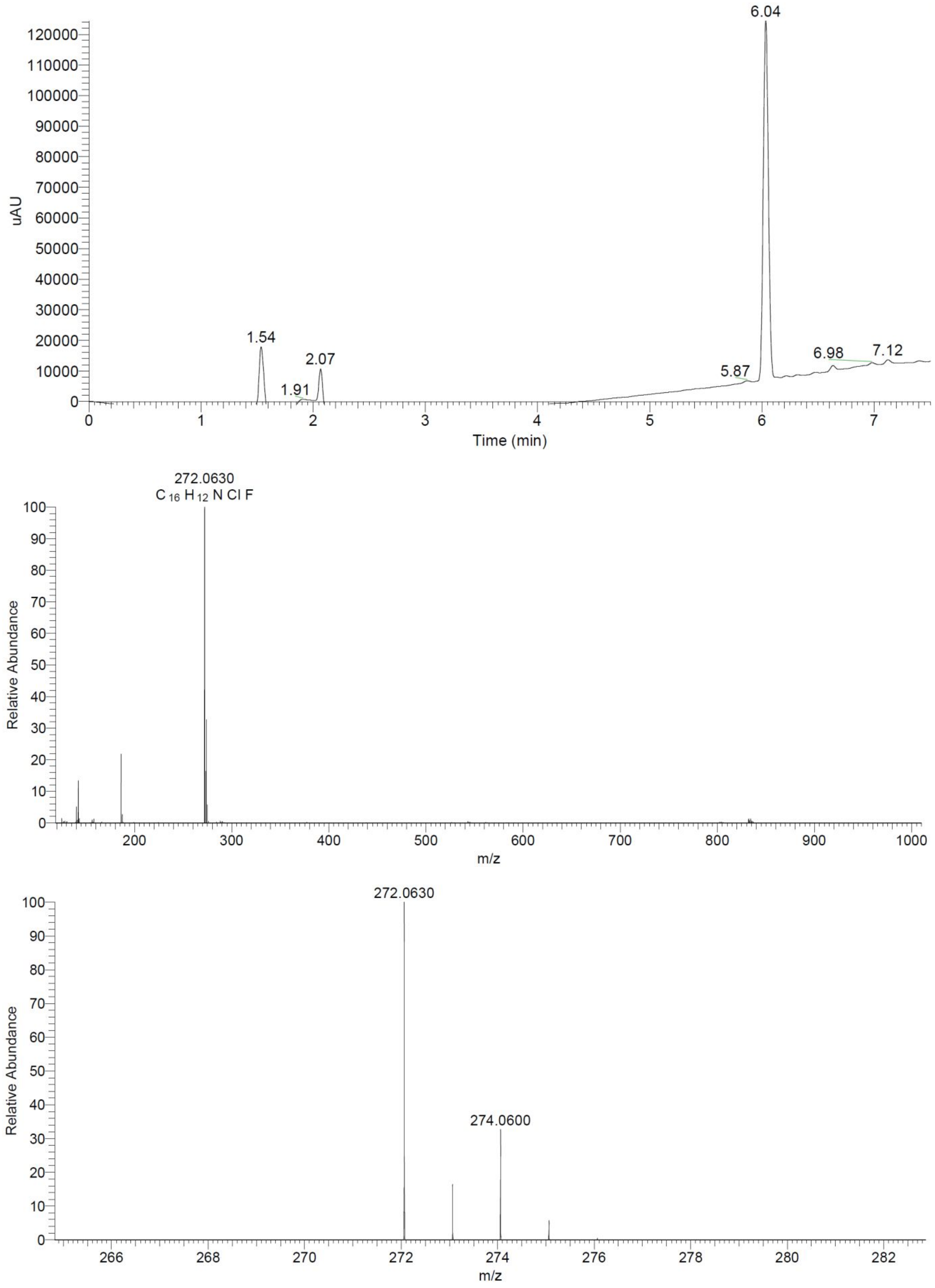

Spectrum 54: UV-trace and mass of main peak of indole **24**. Purity determined by peak area 98%.

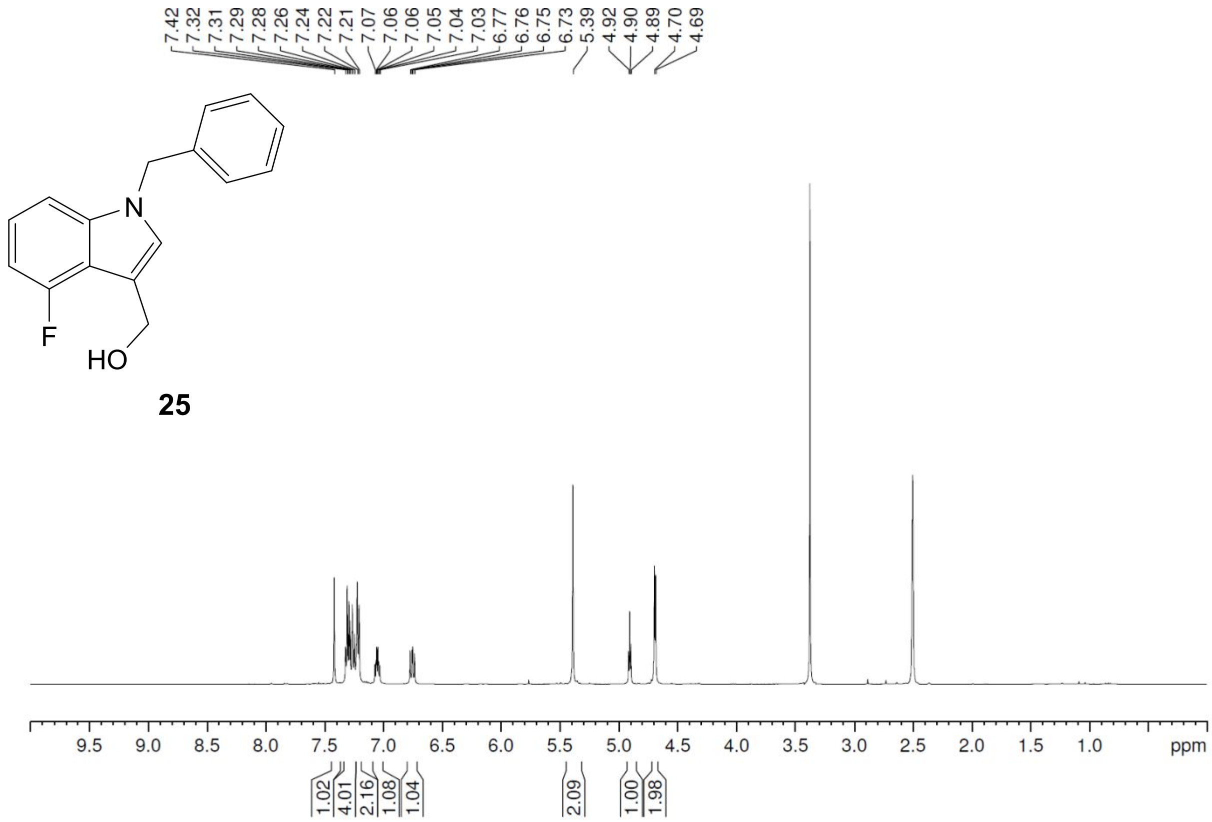

Spectrum 55: ^{1}H-NMR spectrum of indole **25** in $(CD_3)_2OS$.

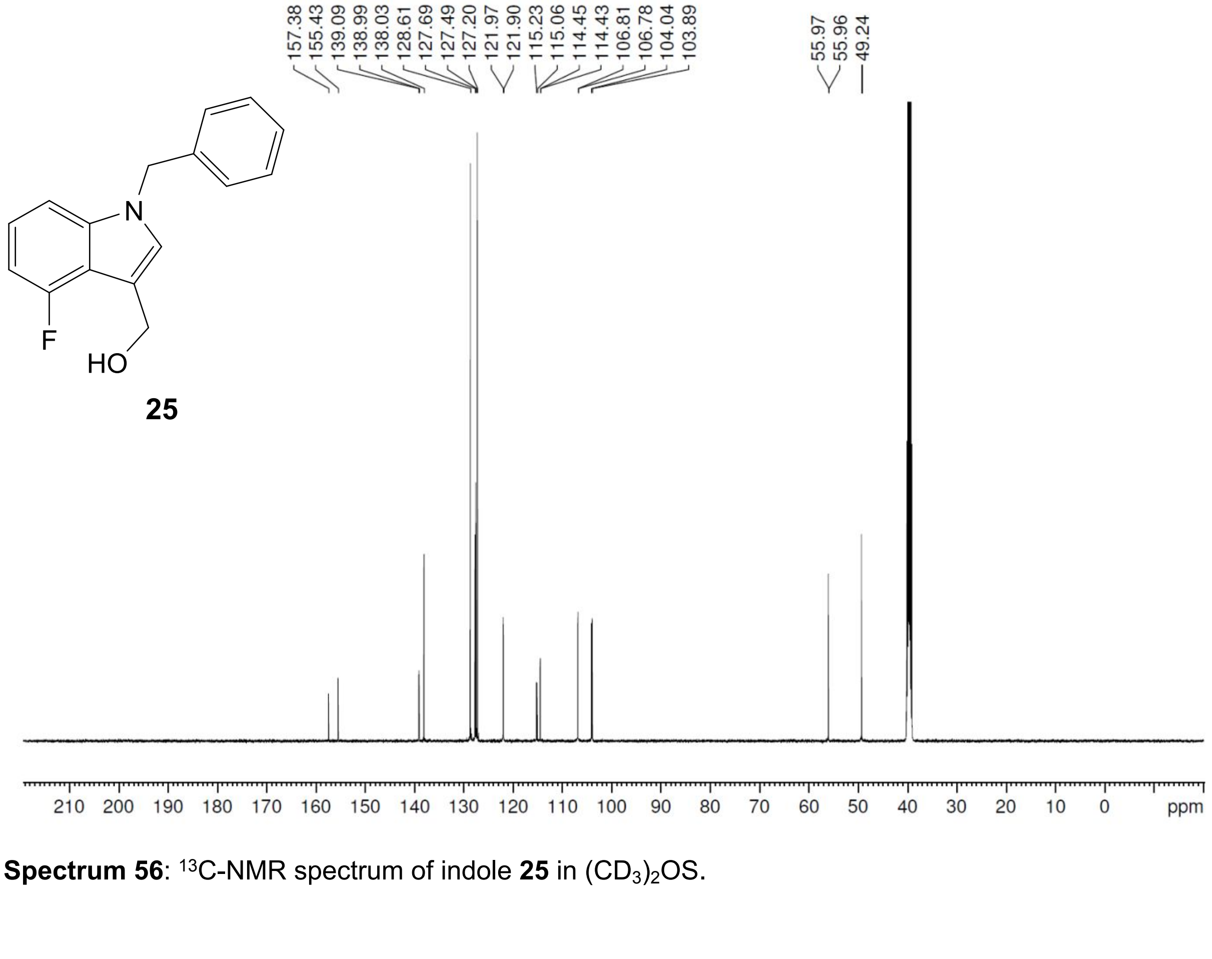

Spectrum 56: ^{13}C-NMR spectrum of indole **25** in $(CD_3)_2OS$.

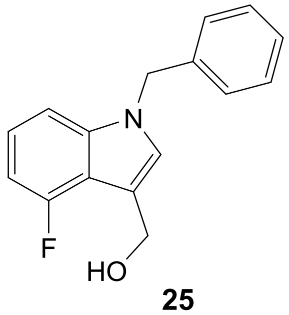

25

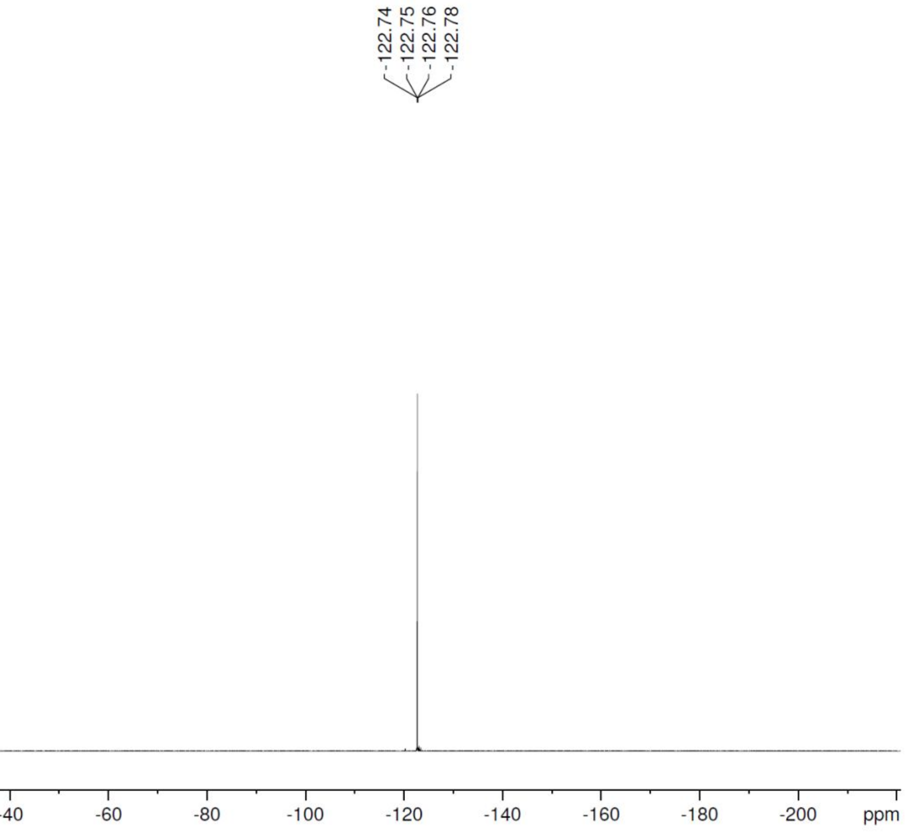

Spectrum 57: ^{19}F-NMR spectrum of indole **25** in $(CD_3)_2OS$.

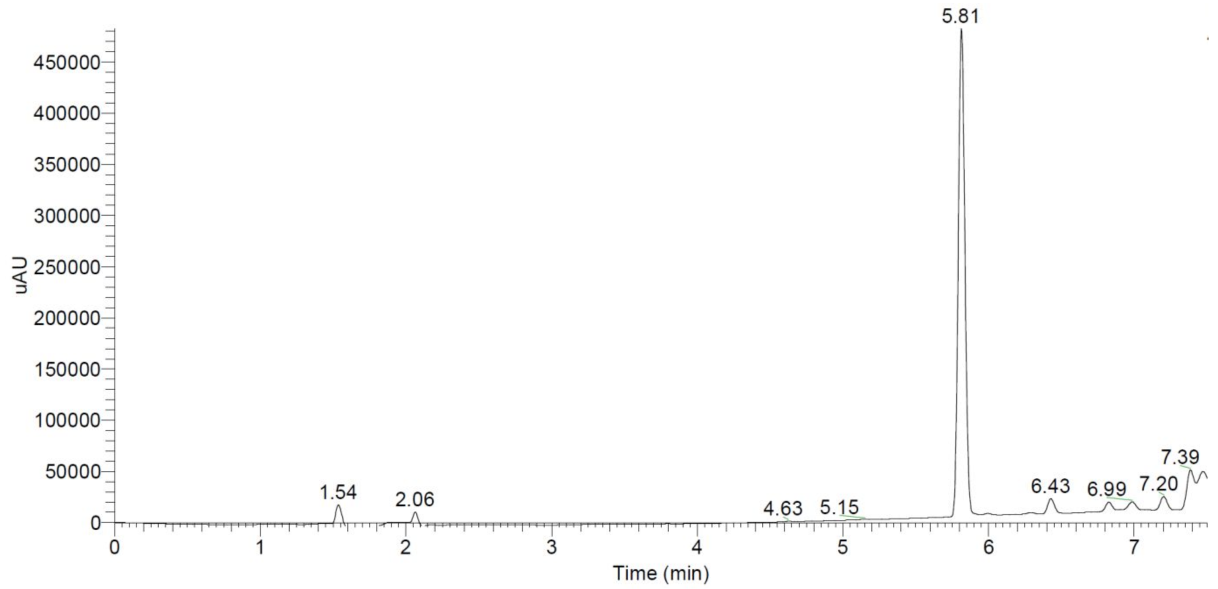

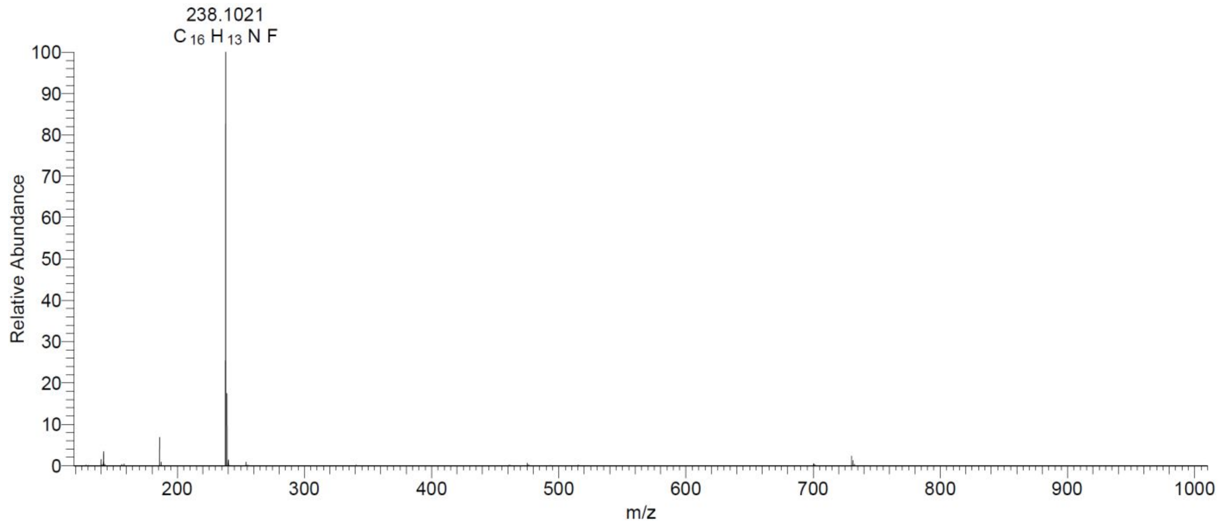

Spectrum 58: UV-trace and mass of main peak of indole **25**. Purity determined by peak area 95%.

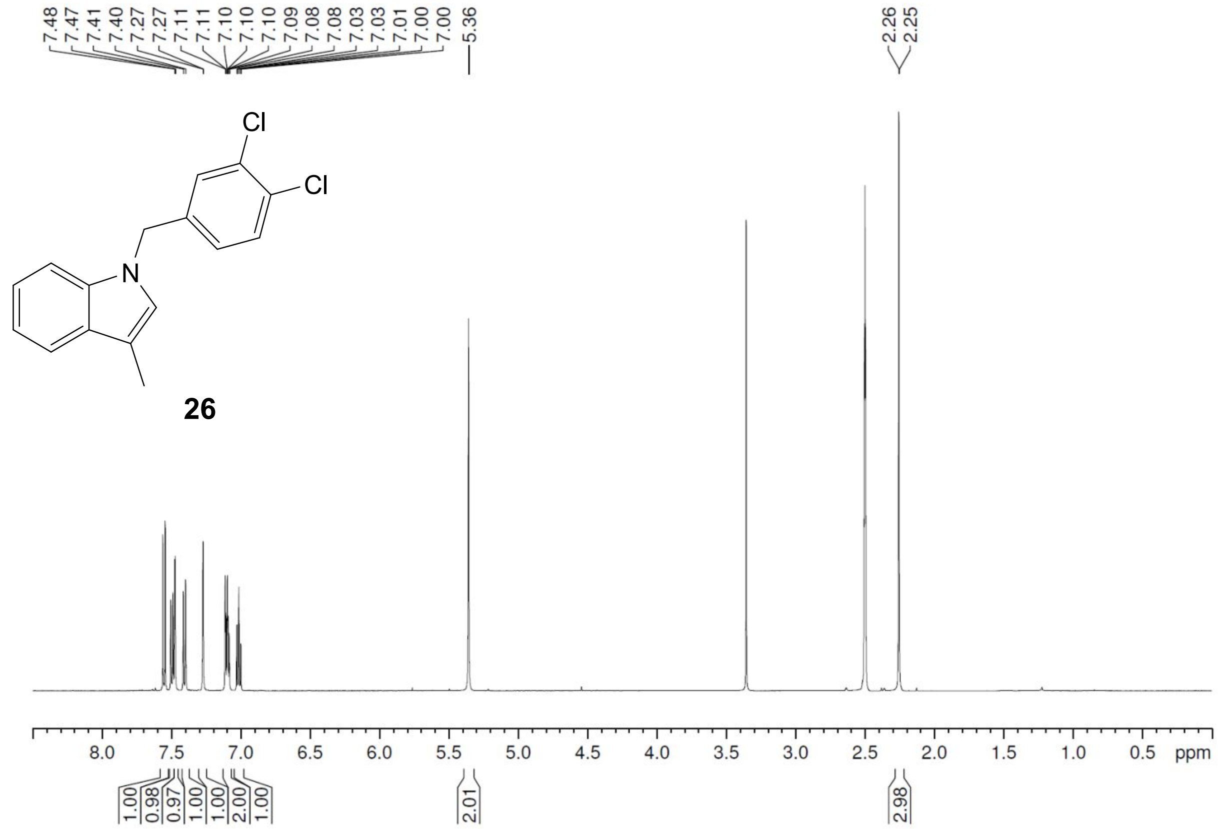

Spectrum 59: ^{1}H-NMR spectrum of indole **26** in $(CD_3)_2OS$.

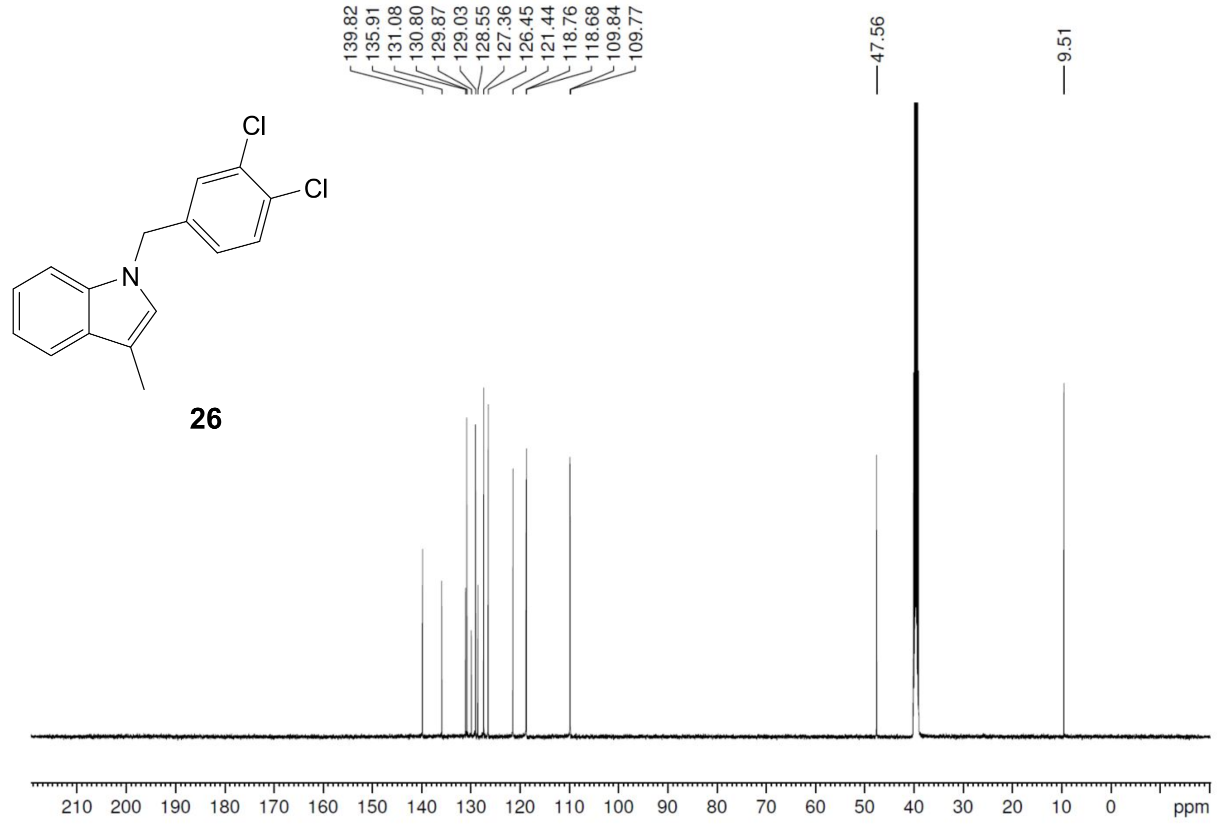

Spectrum 60: ^{13}C-NMR spectrum of indole **26** in $(CD_3)_2OS$.

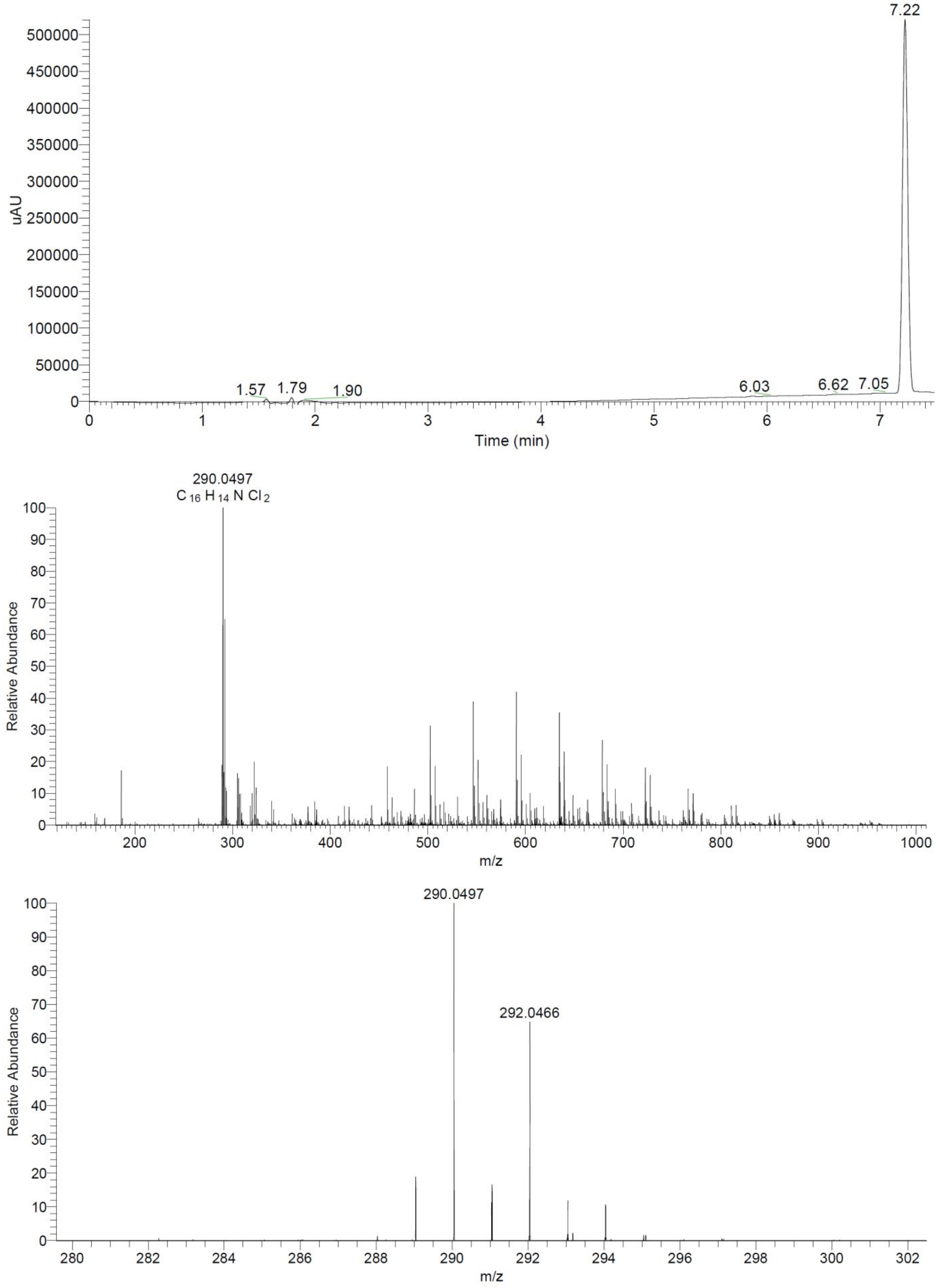

Spectrum 61: UV-trace and mass of main peak of indole **26**. Purity determined by peak area 98%.

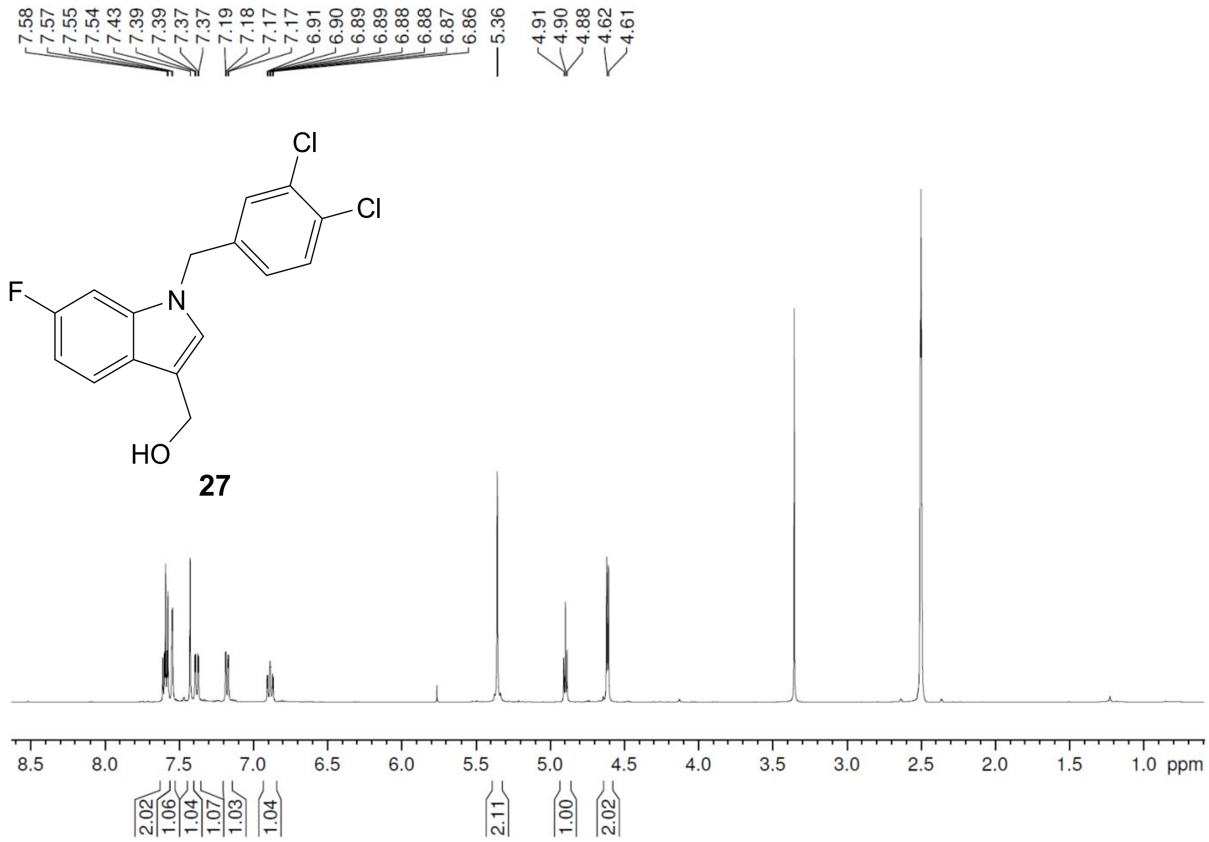

Spectrum 62: ^{1}H-NMR spectrum of indole **27** in $(CD_3)_2OS$.

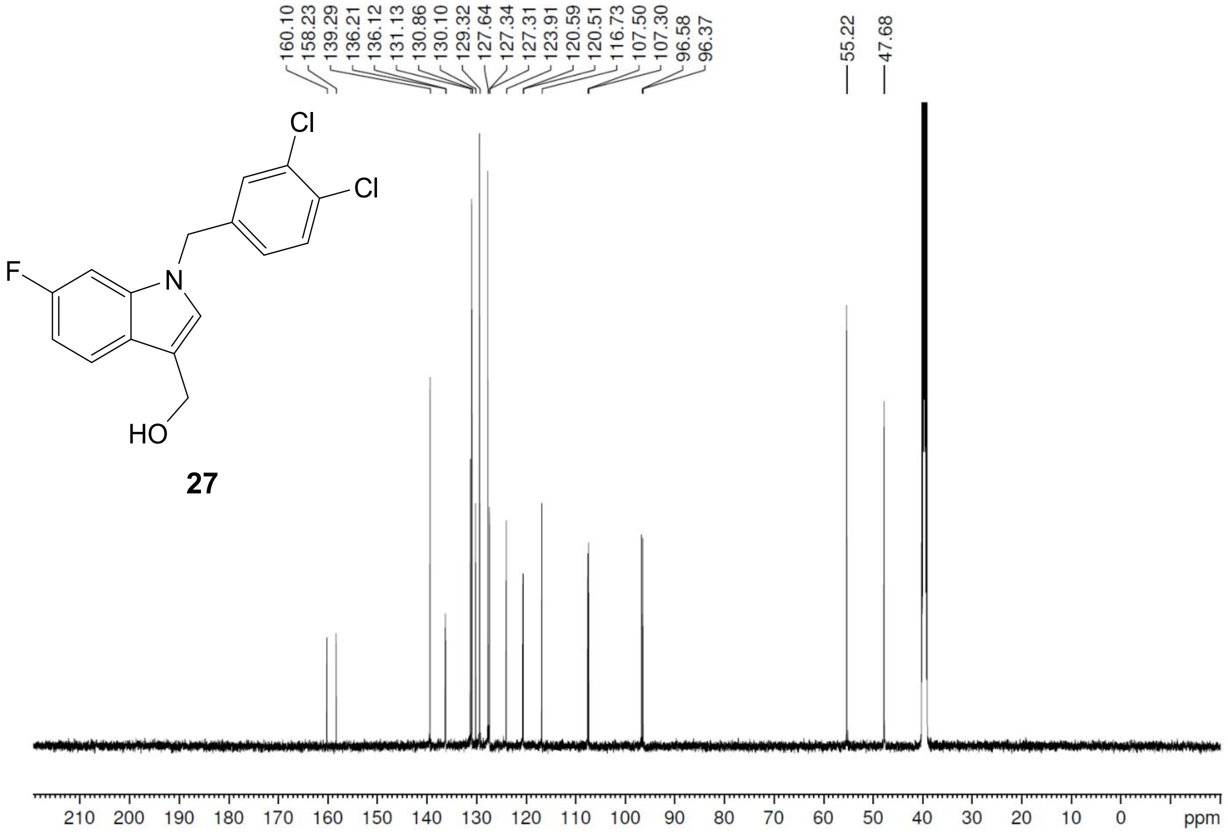

Spectrum 63: ^{13}C-NMR spectrum of indole **27** in $(CD_3)_2OS$.

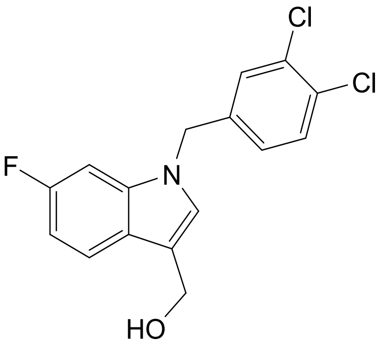

27

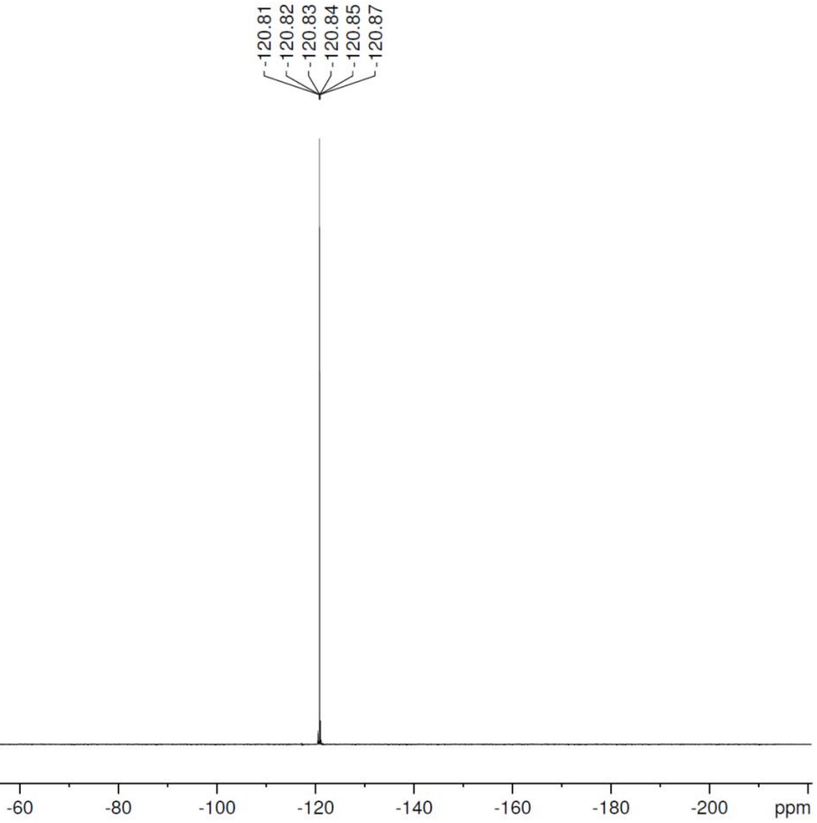

Spectrum 64: ^{19}F-NMR spectrum of indole **27** in $(CD_3)_2OS$.

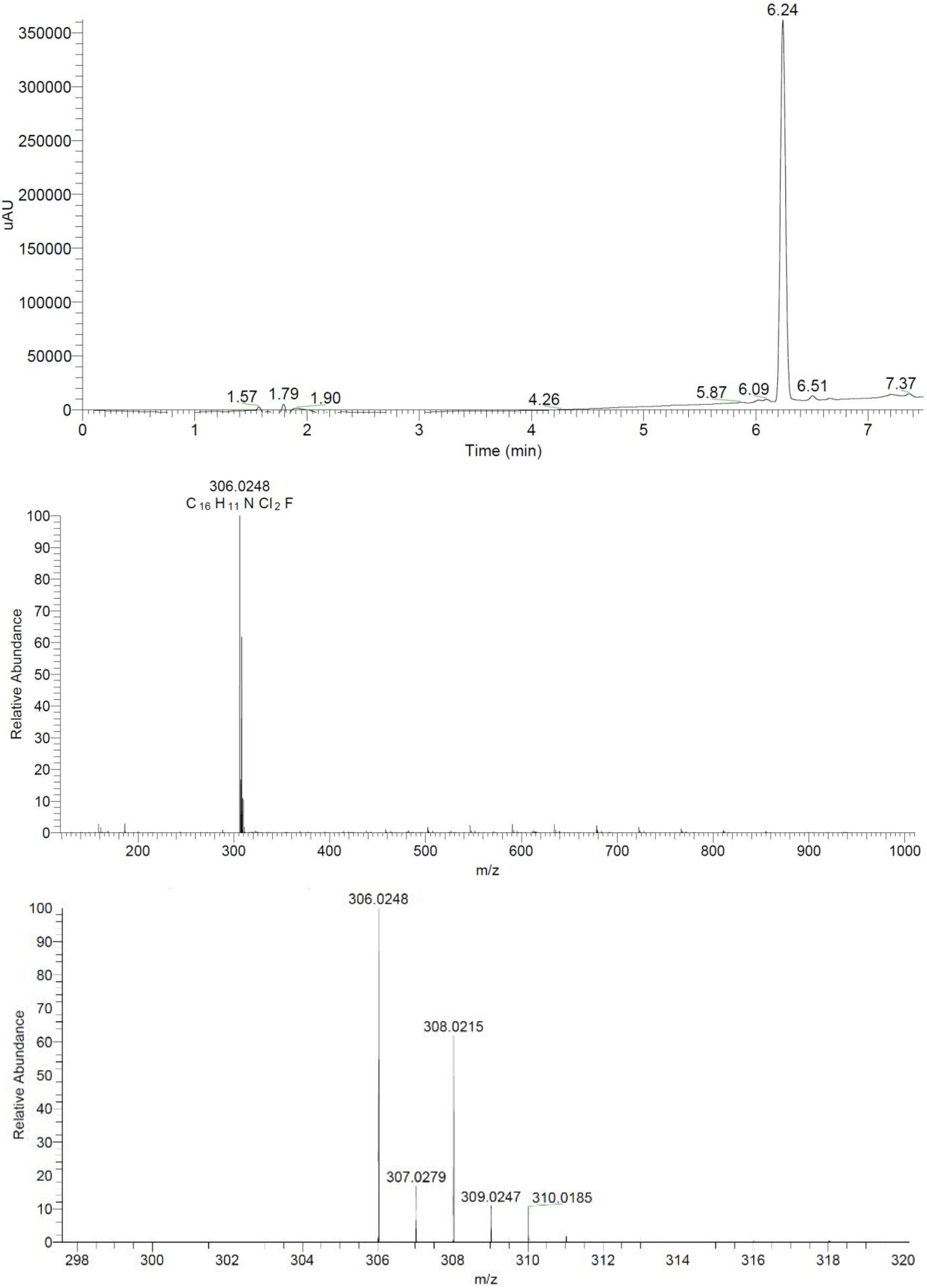

Spectrum 65: UV-trace and mass of main peak of indole **27**. Purity determined by peak area 97%.

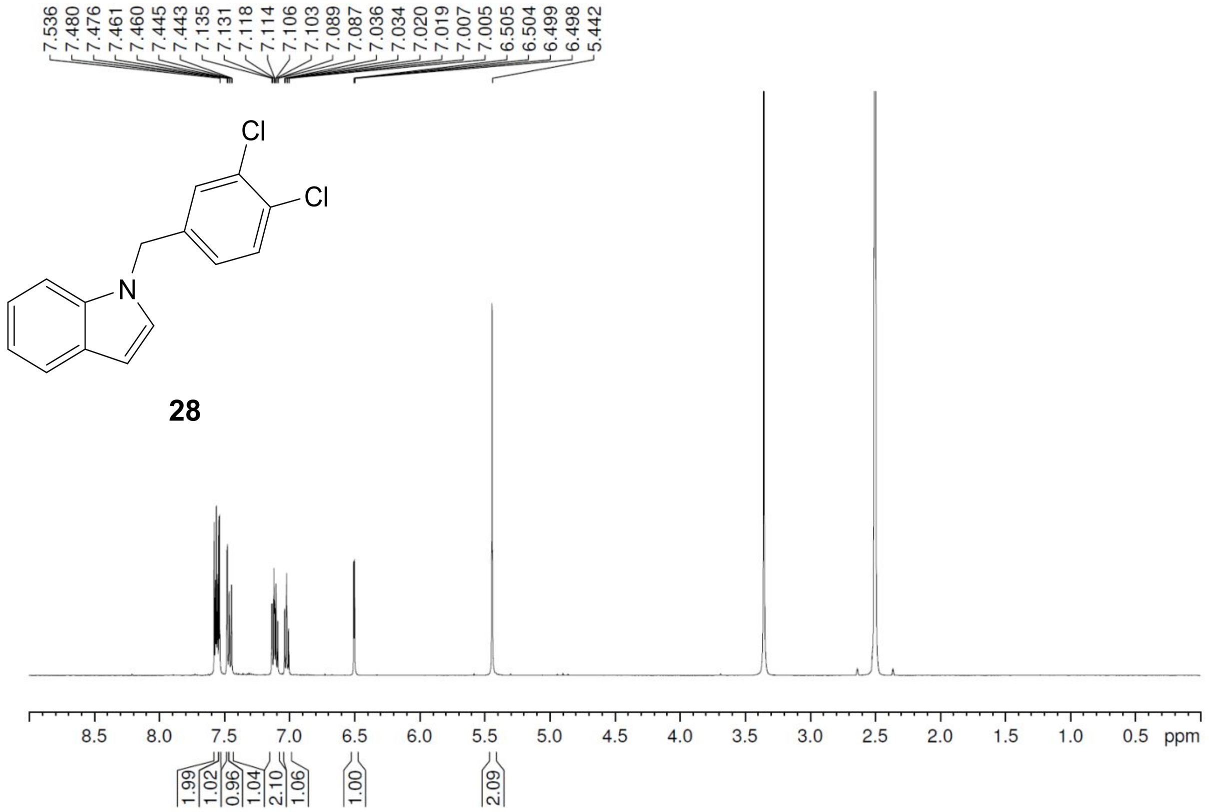

Spectrum 66: ^{1}H-NMR spectrum of indole **28** in $(CD_3)_2OS$.

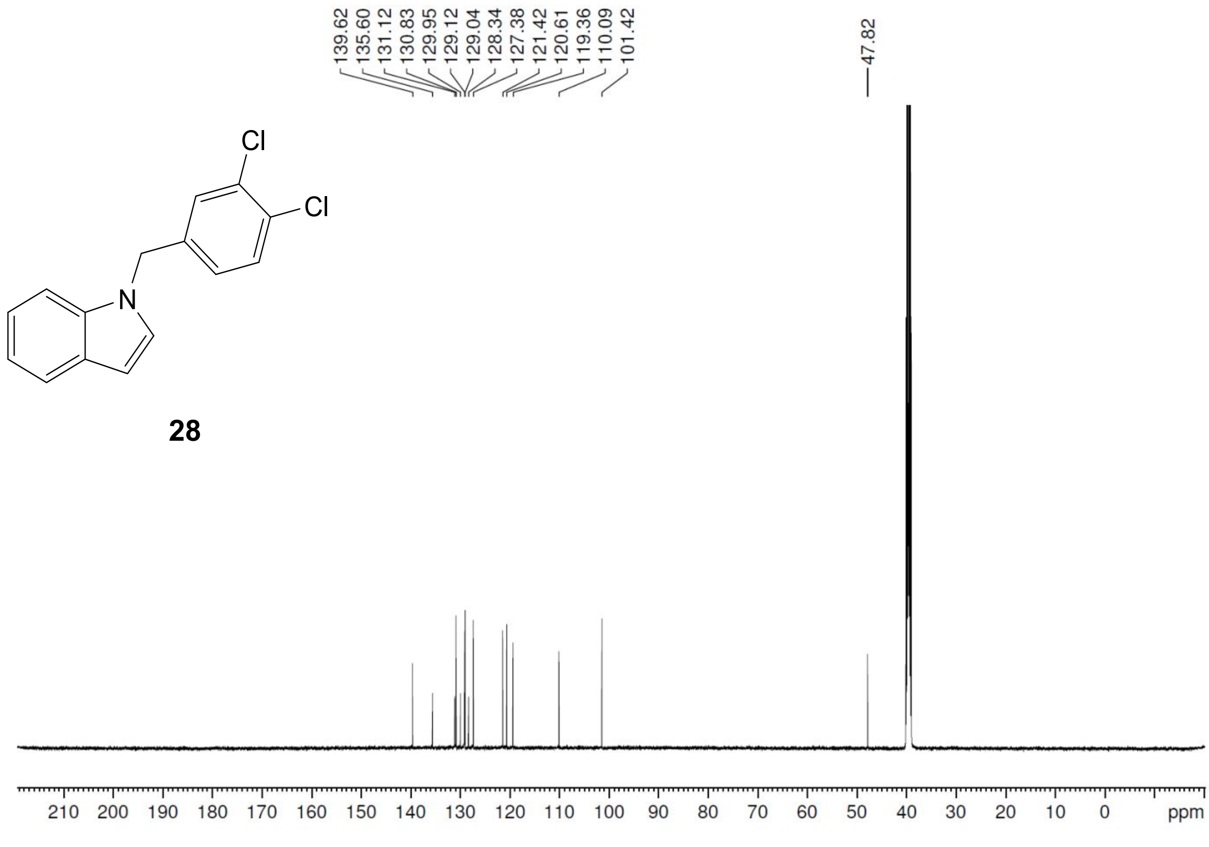

Spectrum 67: ^{13}C-NMR spectrum of indole **28** in $(CD_3)_2OS$.

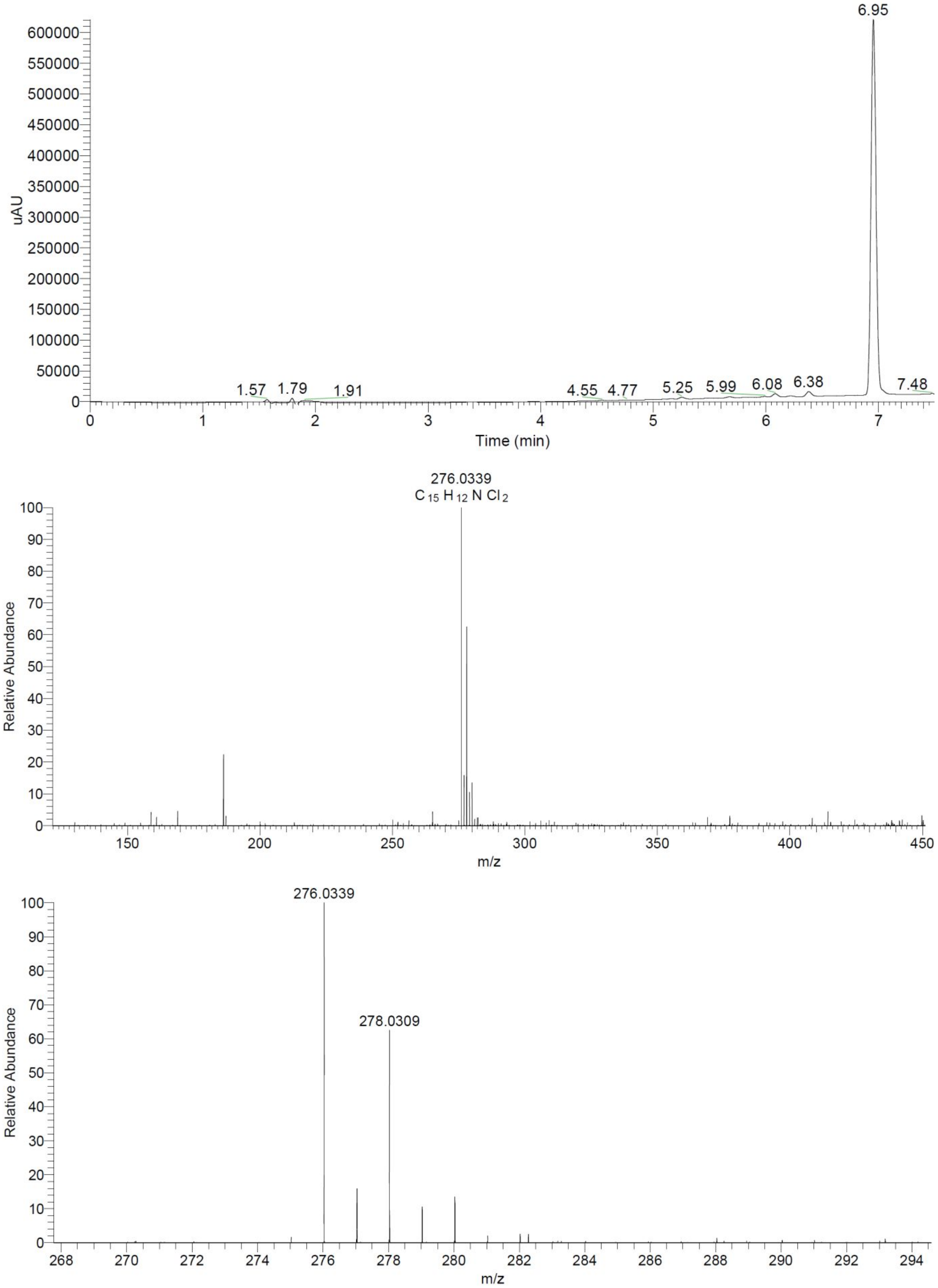

Spectrum 68: UV-trace and mass of main peak of indole **28**. Purity determined by peak area 99%.

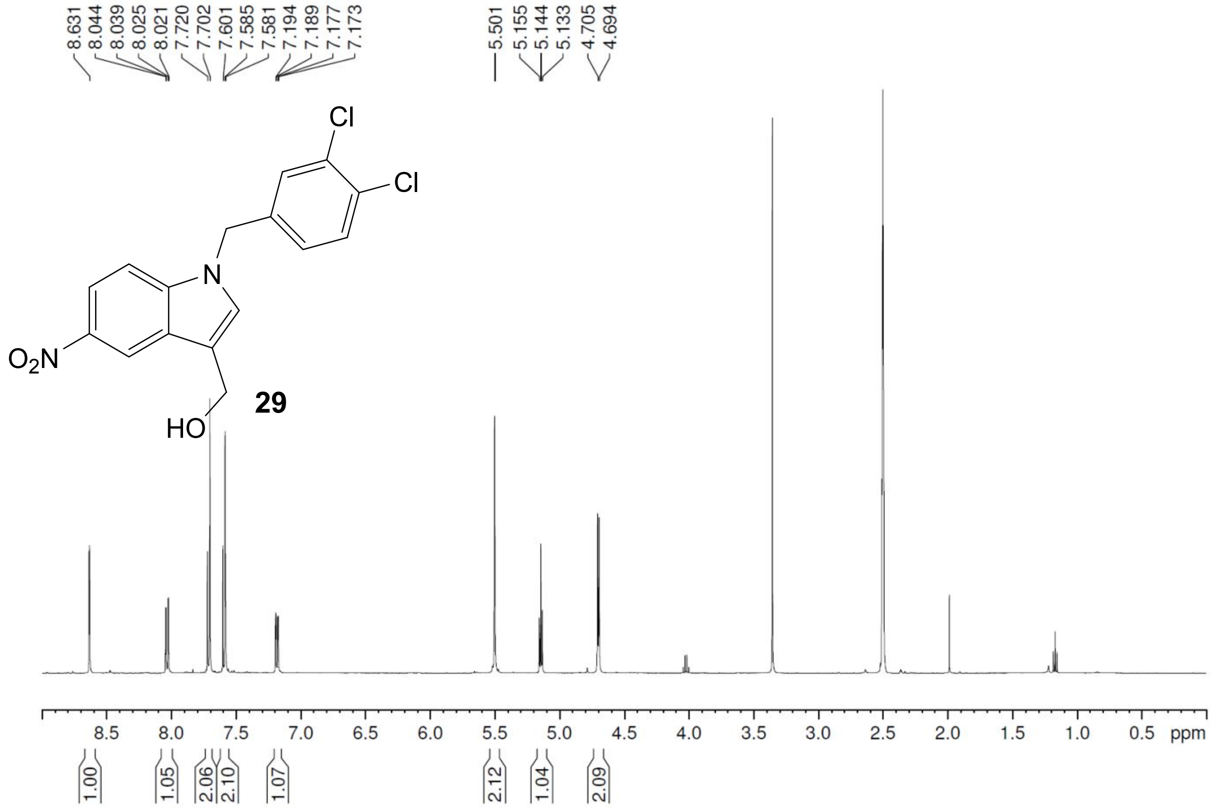

Spectrum 69: ^{1}H-NMR spectrum of indole **29** in $(CD_3)_2OS$.

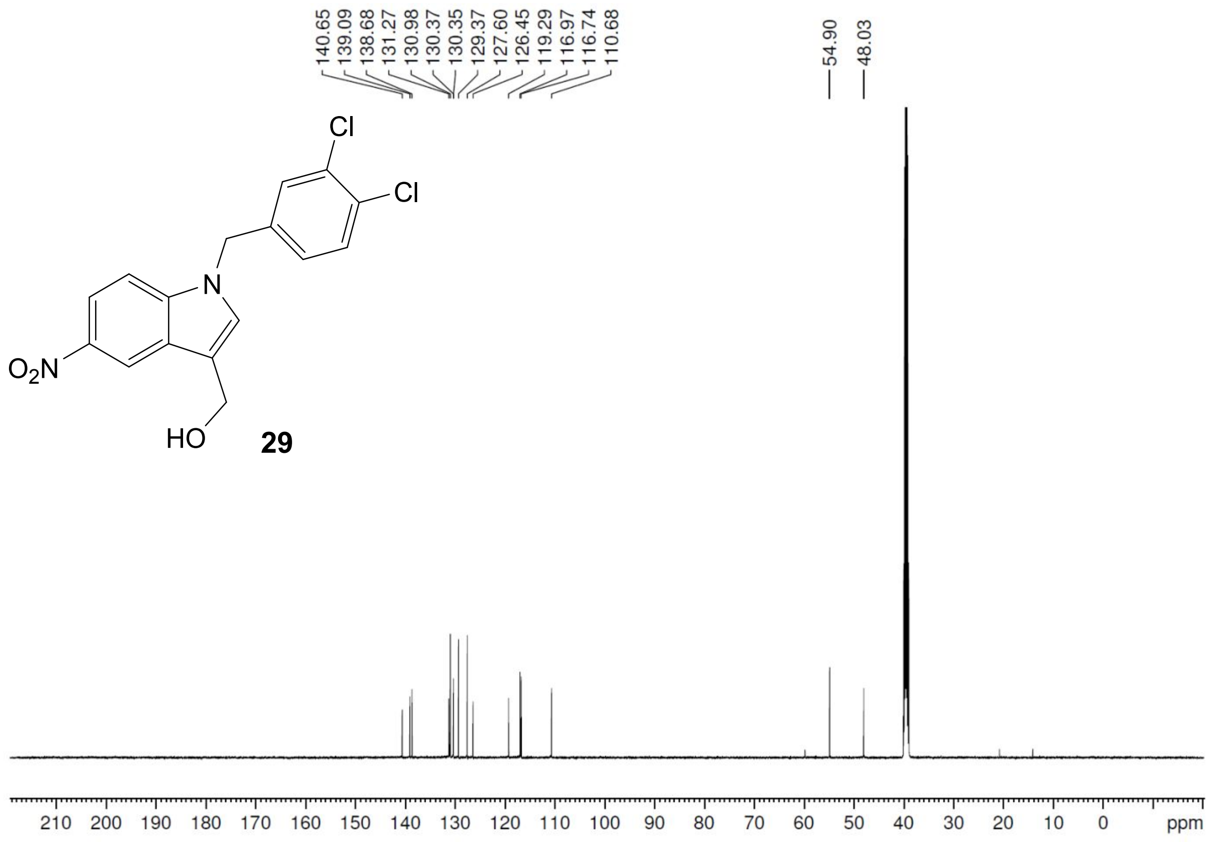

Spectrum 70: ^{13}C-NMR spectrum of indole **29** in $(CD_3)_2OS$.

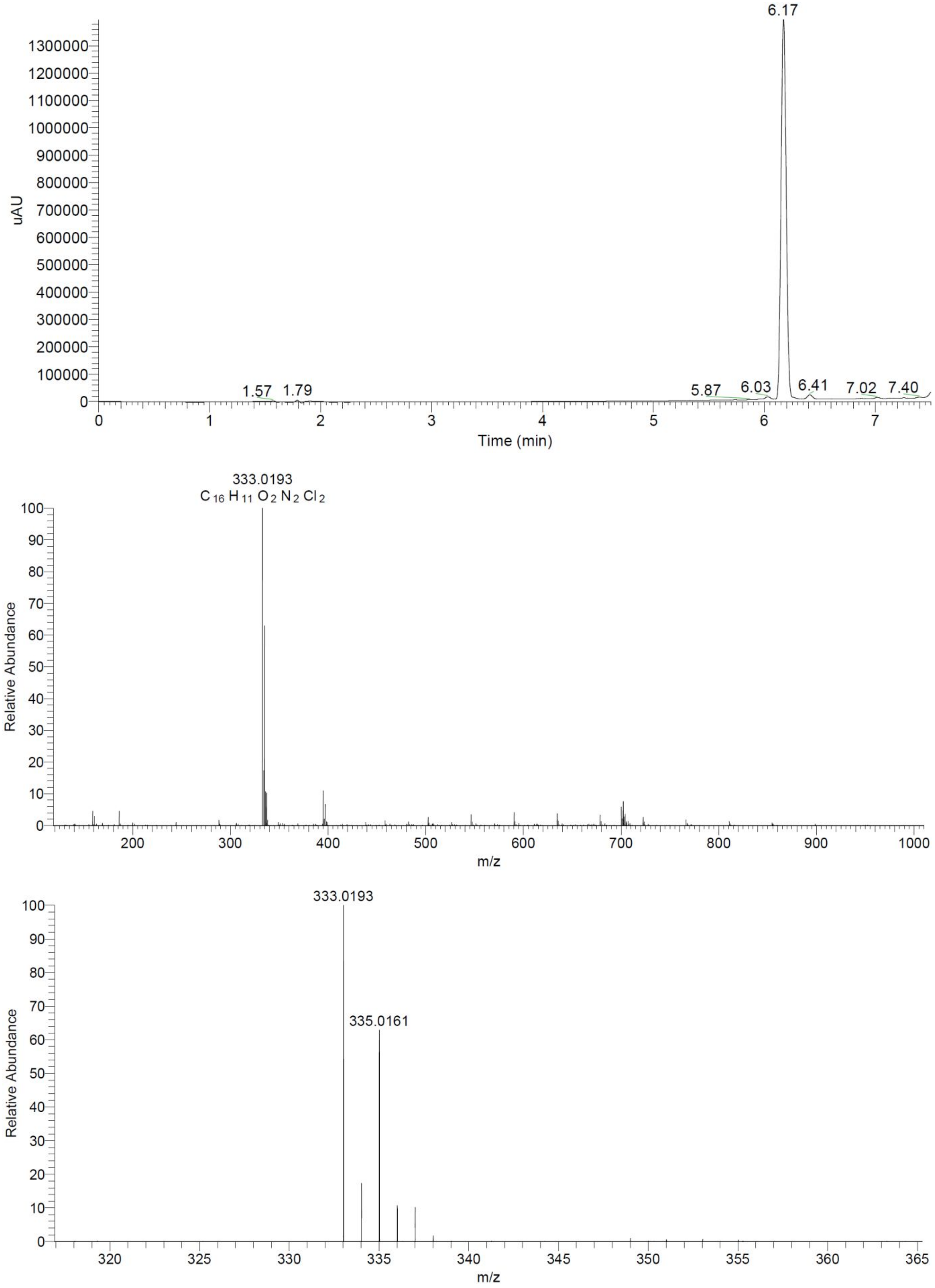

Spectrum 71: UV-trace and mass of main peak of indole **29**. Purity determined by peak area 96%.

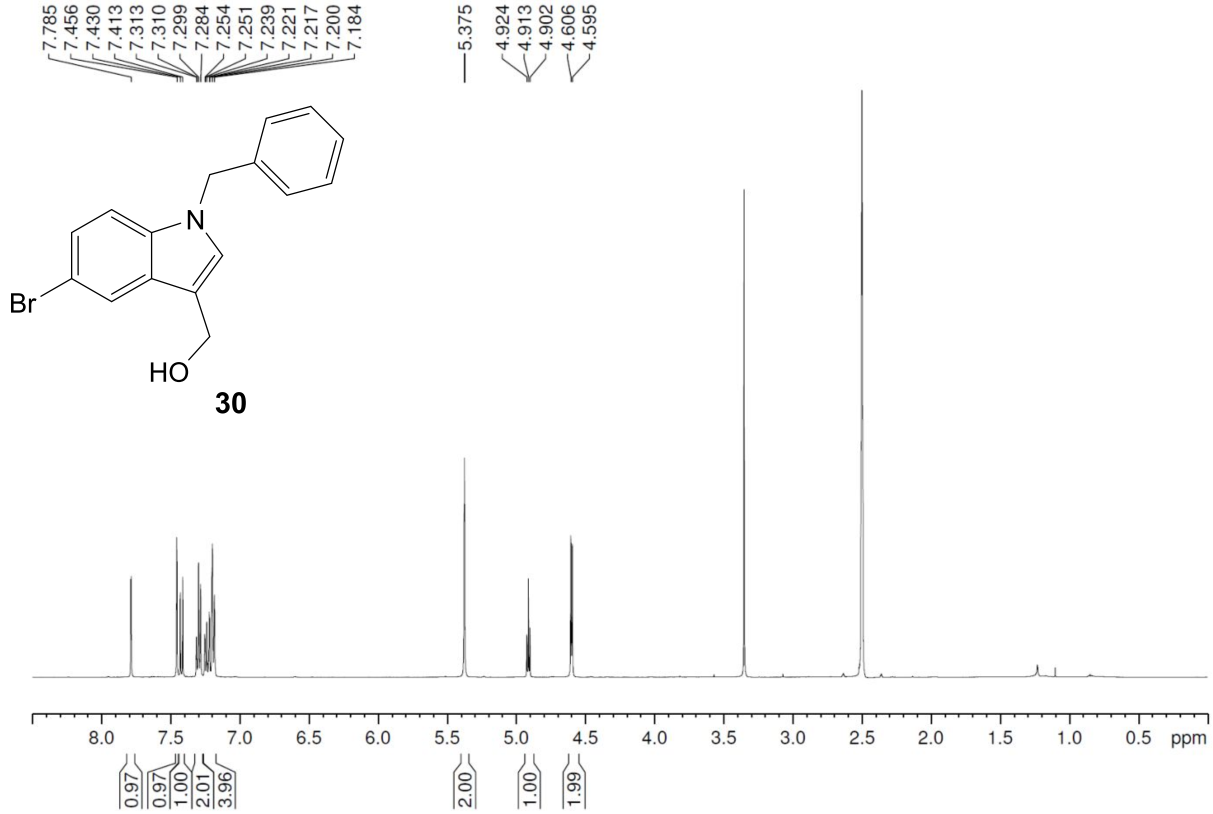

Spectrum 72: ^{1}H-NMR spectrum of indole **30** in $(CD_3)_2OS$.

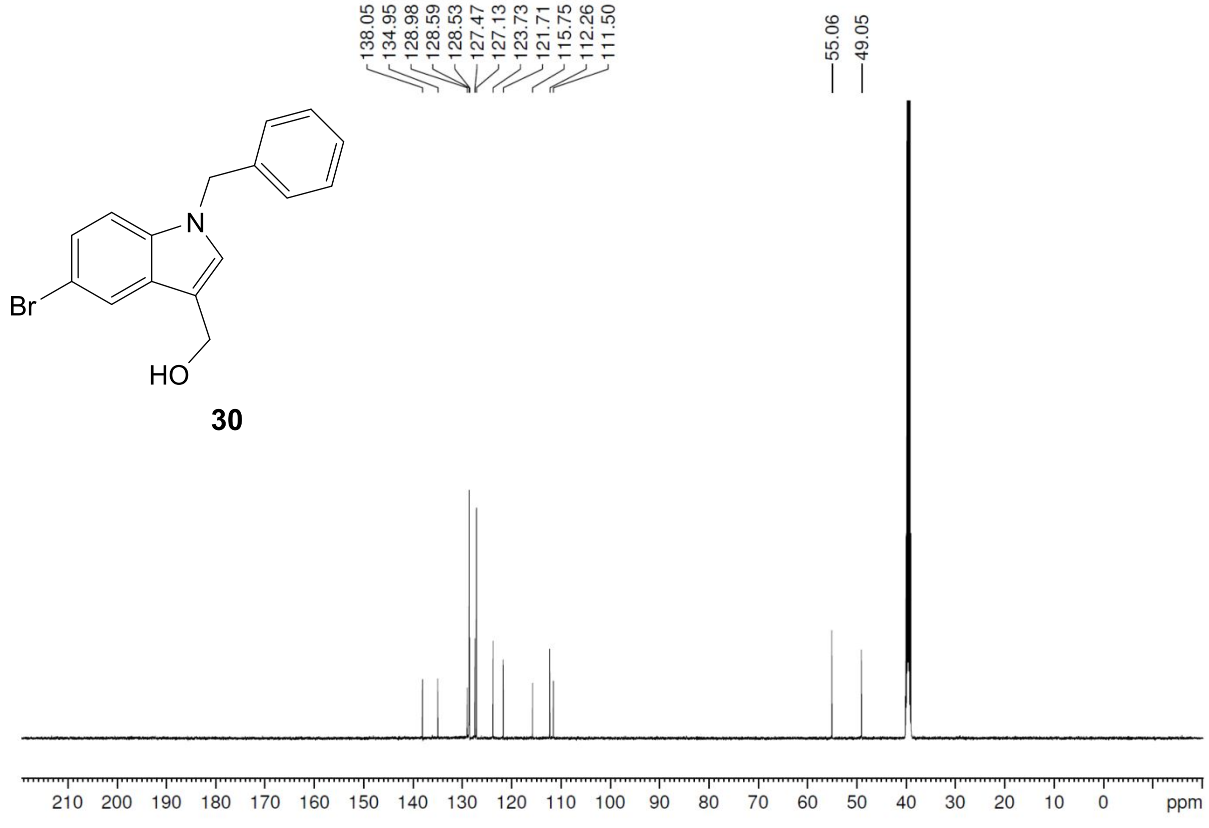

Spectrum 73: ^{13}C-NMR spectrum of indole **30** in $(CD_3)_2OS$.

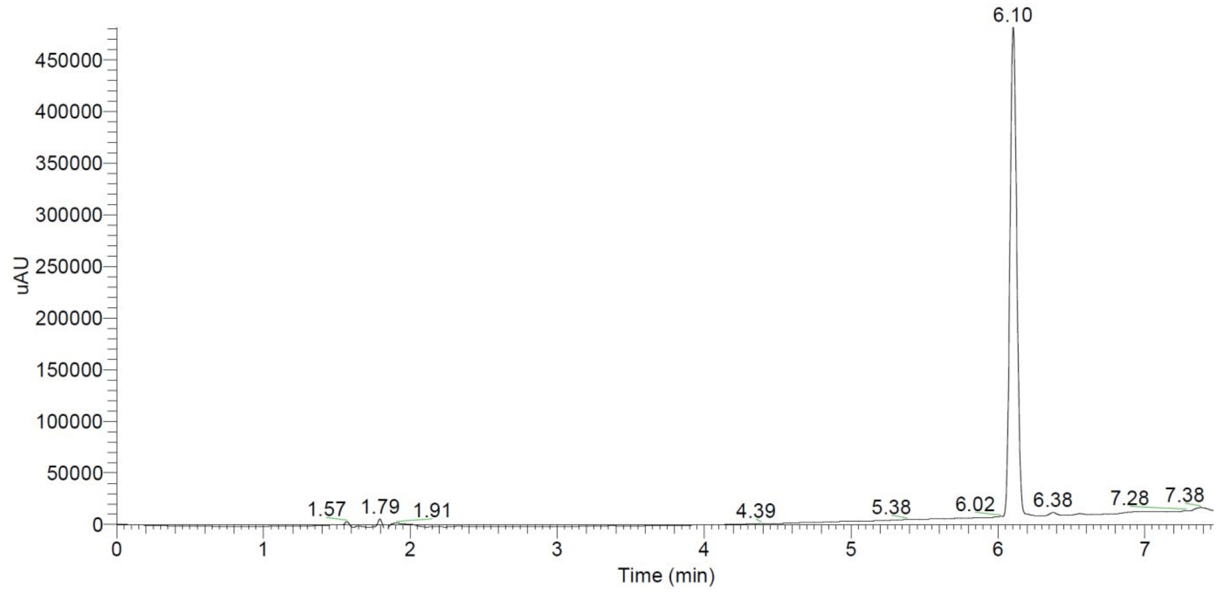

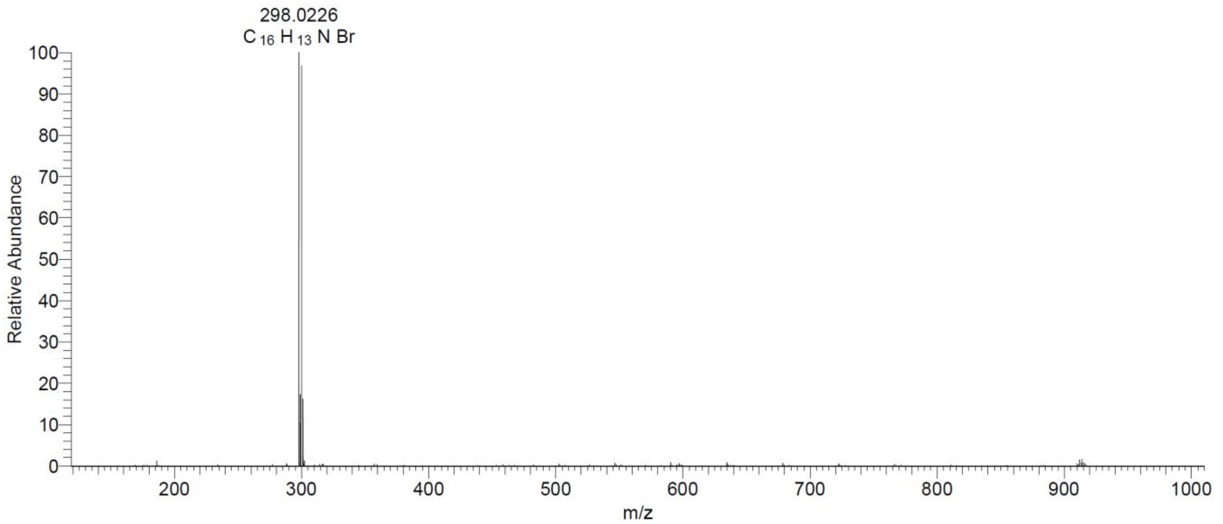

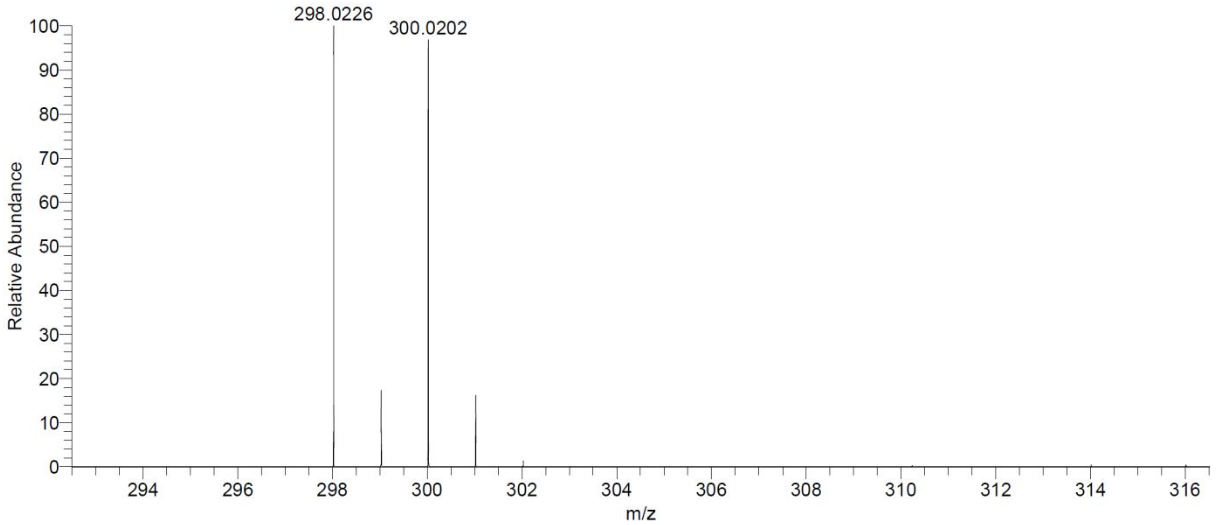

Spectrum 74: UV-trace and mass of main peak of indole **30**. Purity determined by peak area 98%.

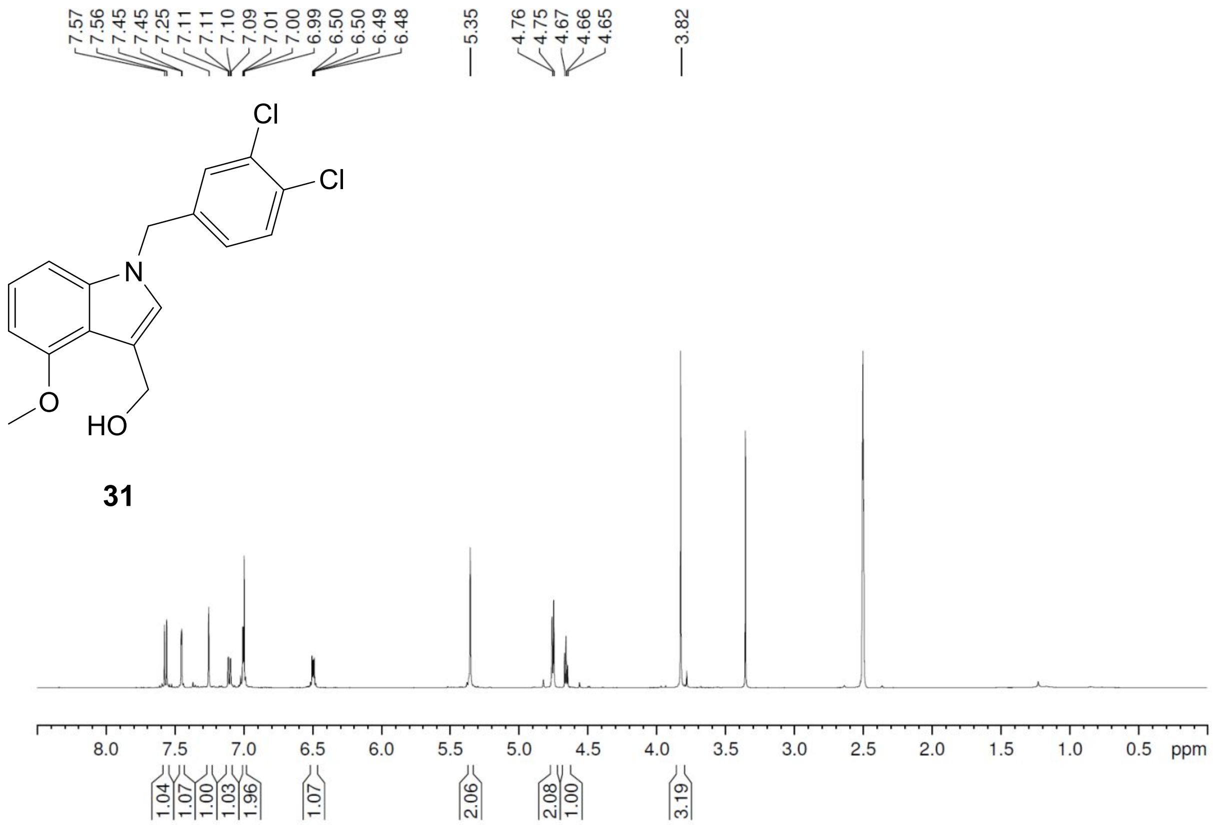

31

Spectrum 75: ^{1}H-NMR spectrum of indole **31** in $(CD_3)_2OS$.

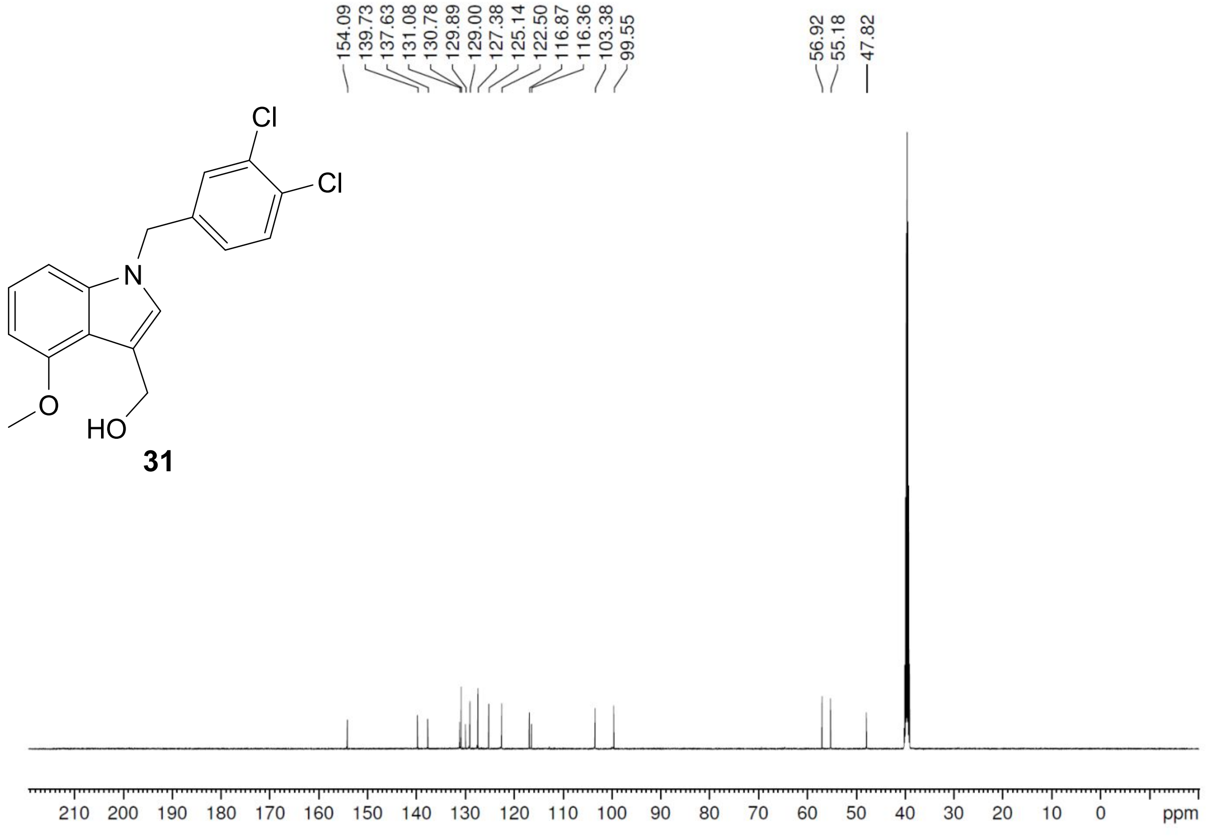

31

Spectrum 76: ^{13}C-NMR spectrum of indole **31** in $(CD_3)_2OS$.

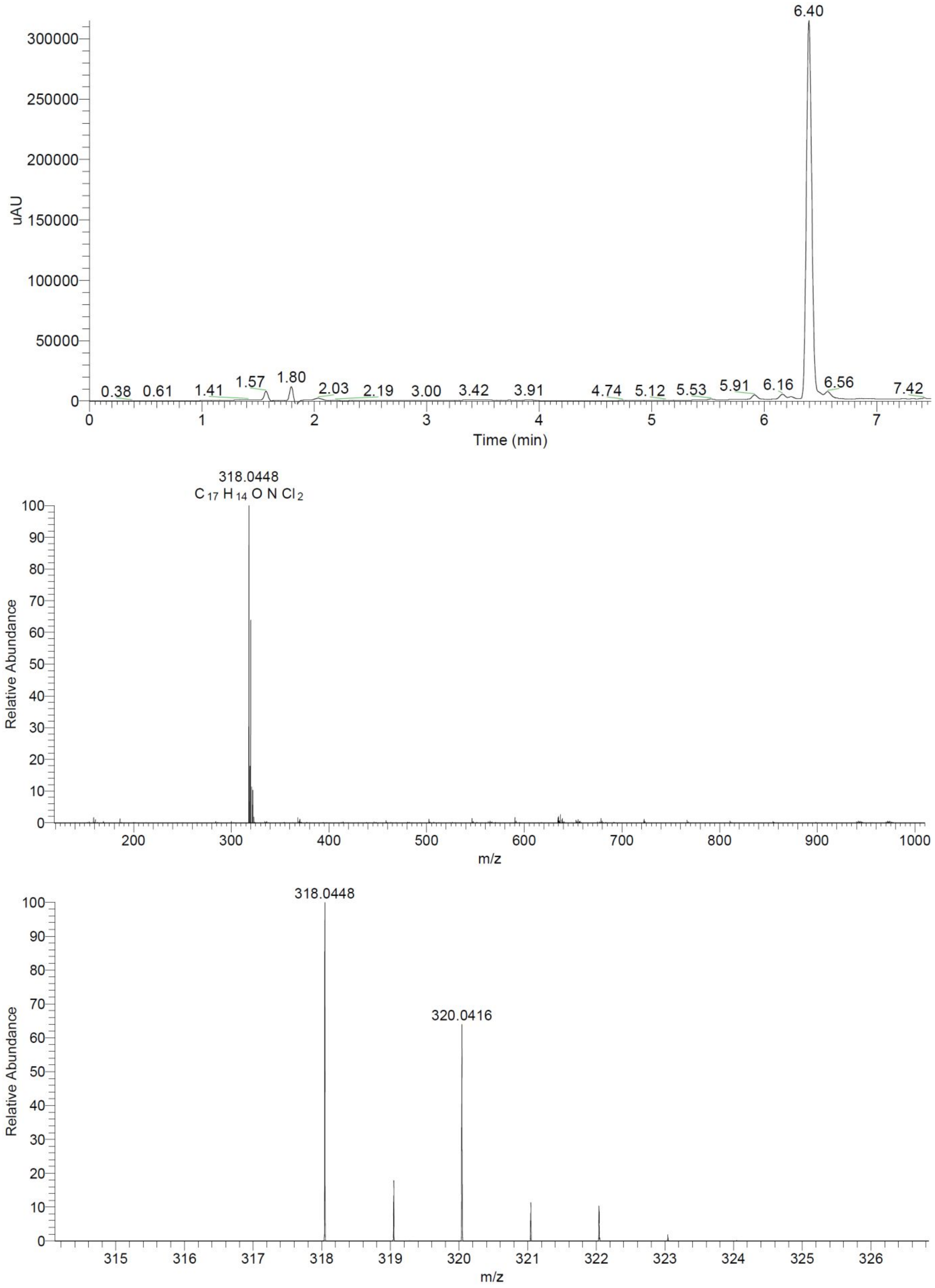

Spectrum 77: UV-trace and mass of main peak of indole **31**. Purity determined by peak area 95%.

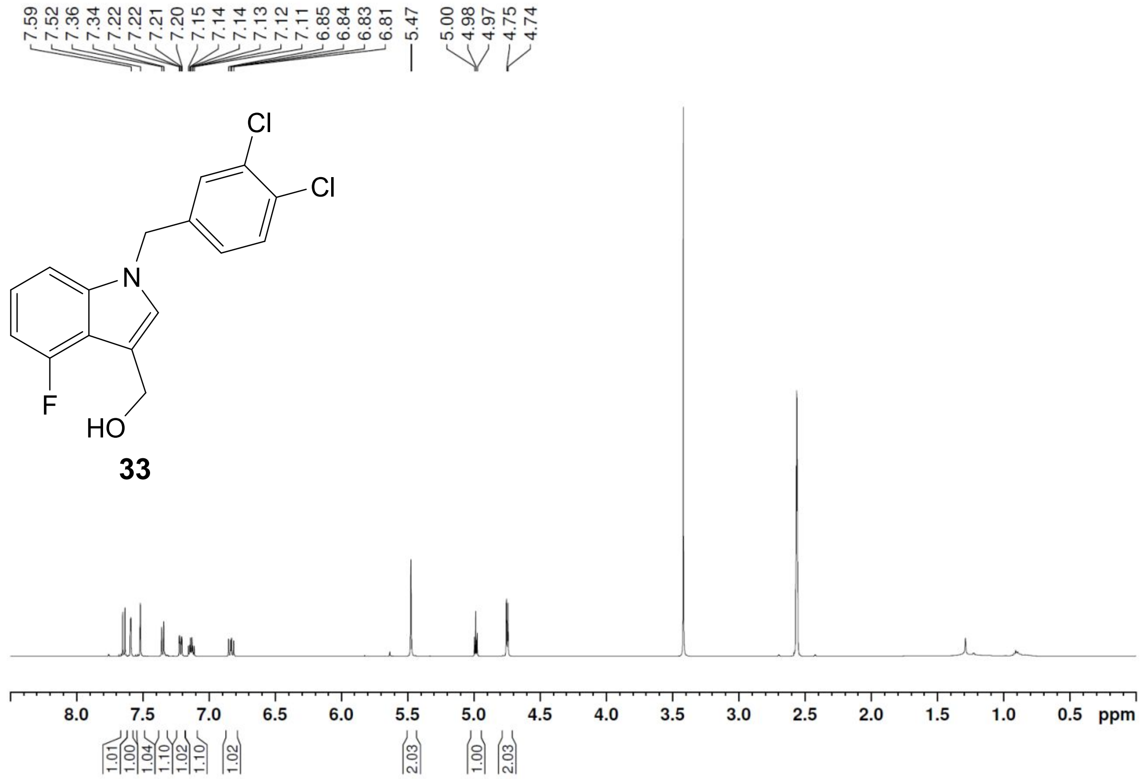

Spectrum 78: ^{1}H-NMR spectrum of indole **33** in $(CD_3)_2OS$.

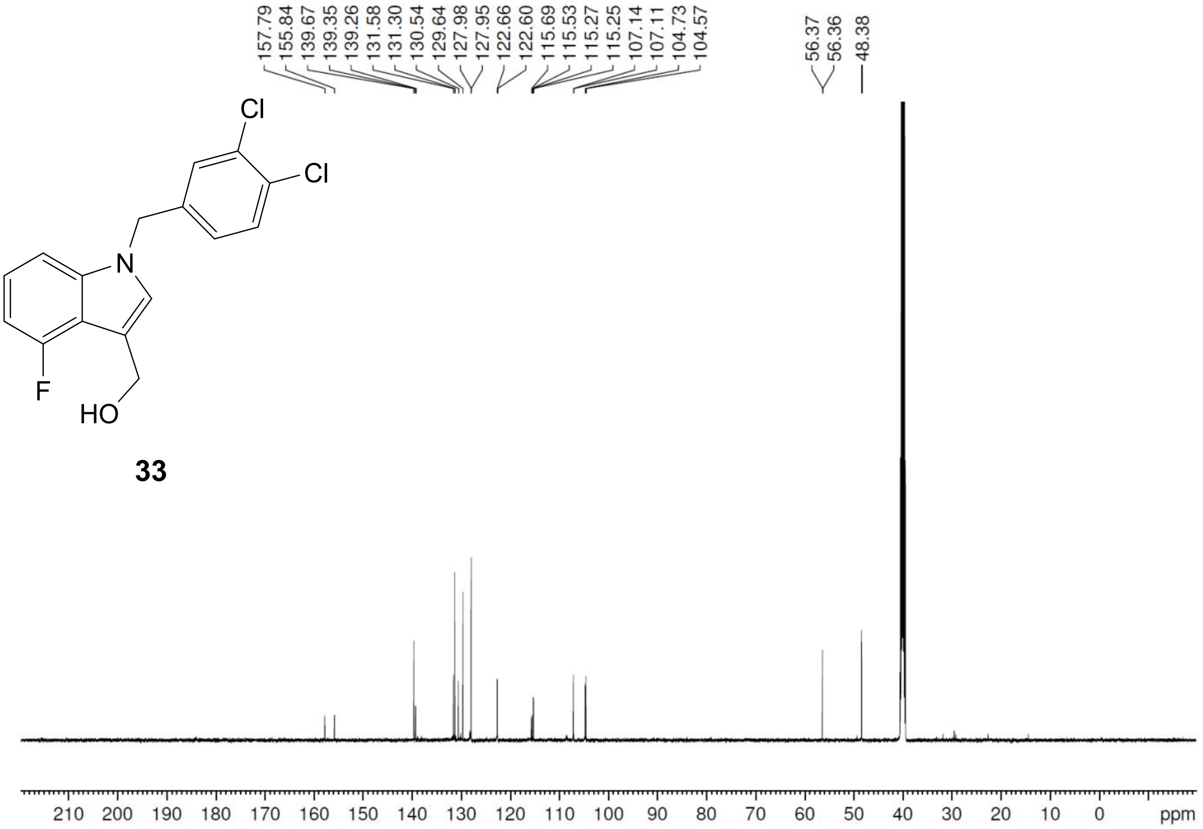

Spectrum 79: ^{13}C-NMR spectrum of indole **33** in $(CD_3)_2OS$.

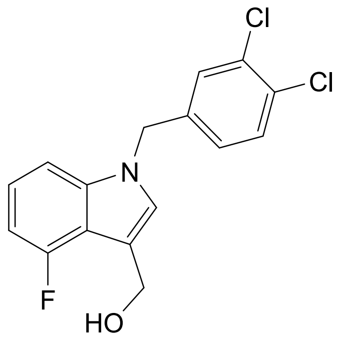

33

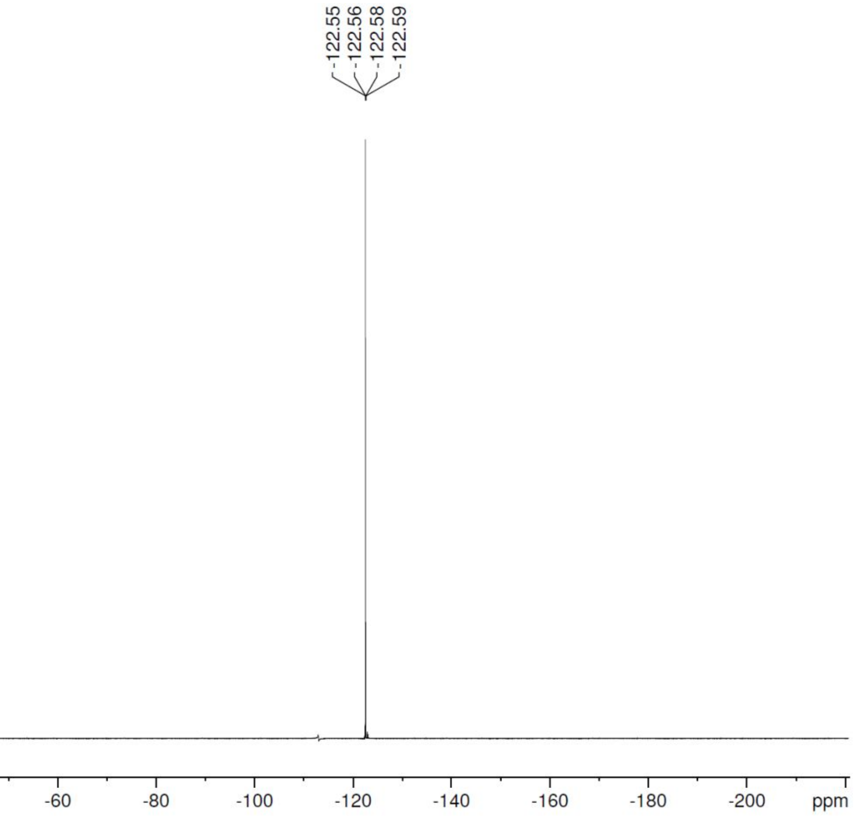

Spectrum 80: ^{19}F-NMR spectrum of indole **33** in $(CD_3)_2OS$.

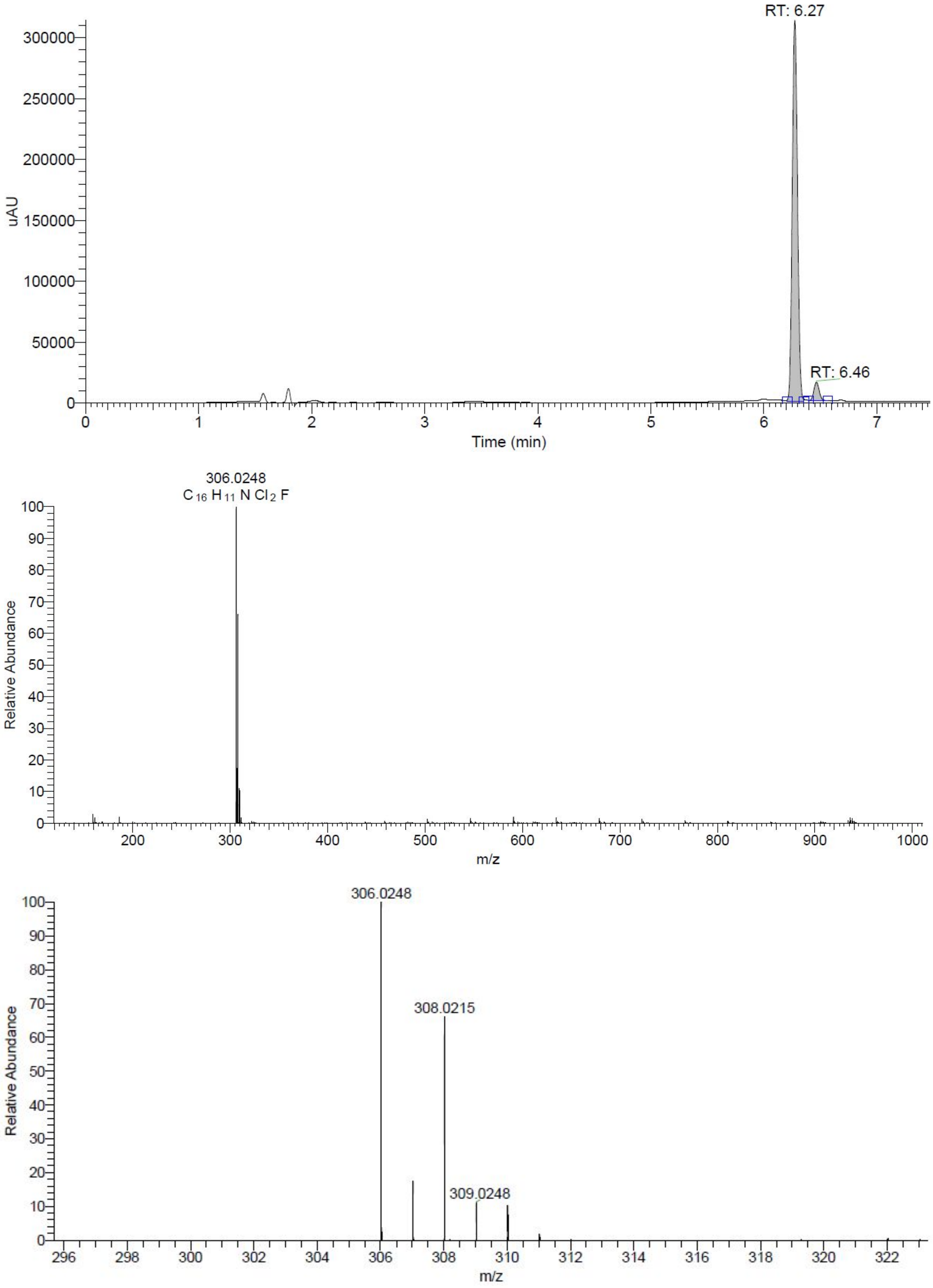

Spectrum 81: UV-trace and mass of main peak of indole **33**. Purity determined by peak area 95%.

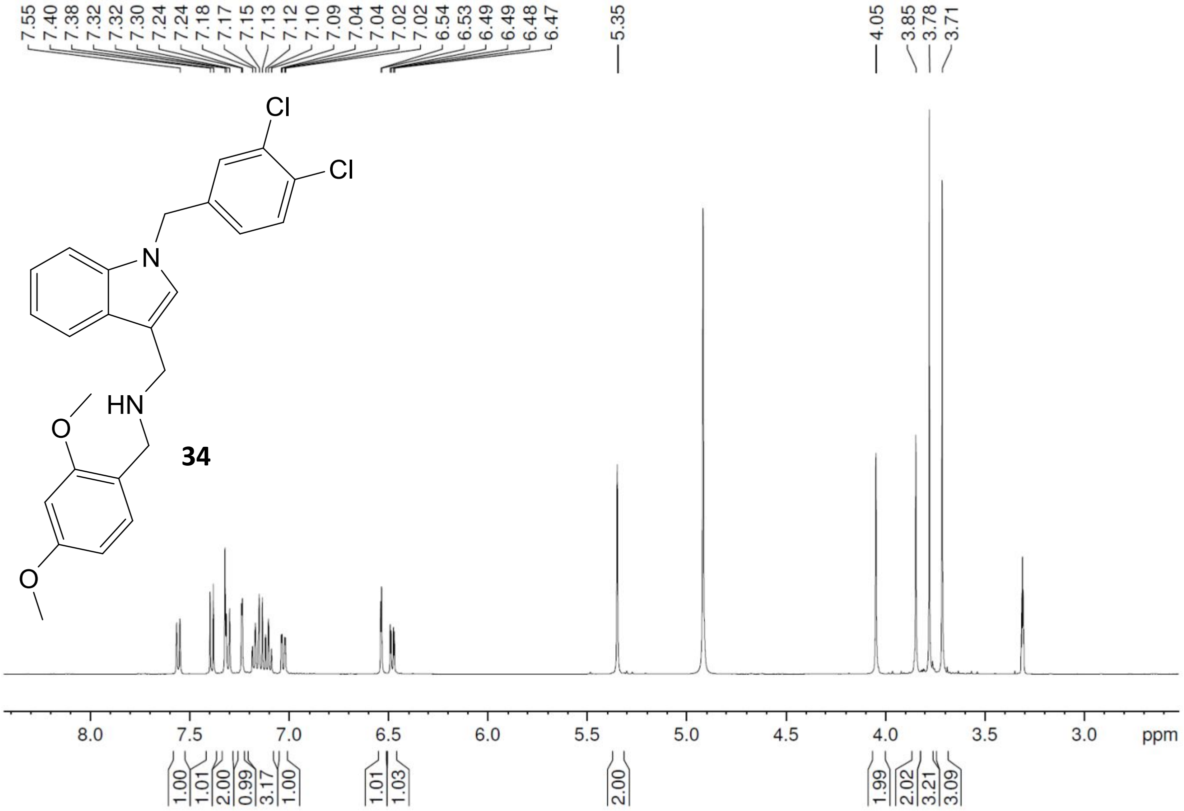

Spectrum 82: ^{1}H-NMR spectrum of indole **34** in CD_3OD.

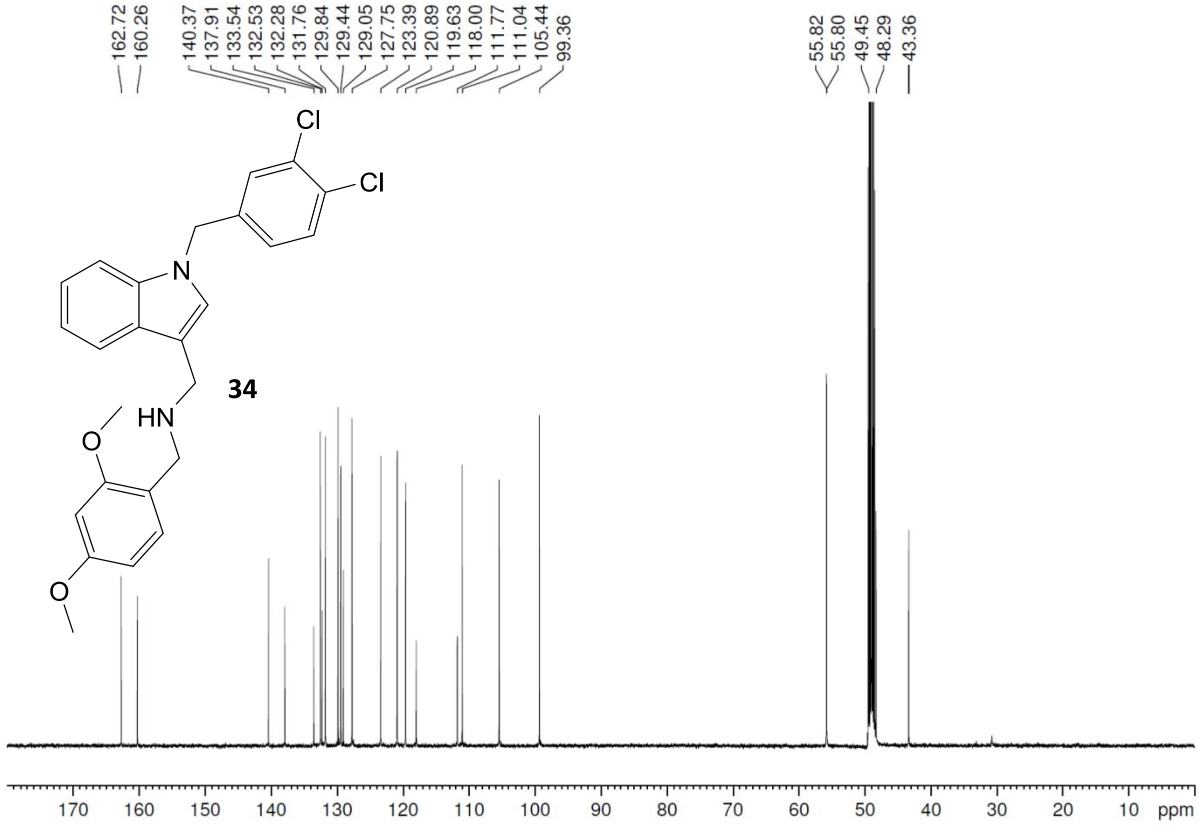

Spectrum 83: ^{13}C-NMR spectrum of indole **34** in CD_3OD.

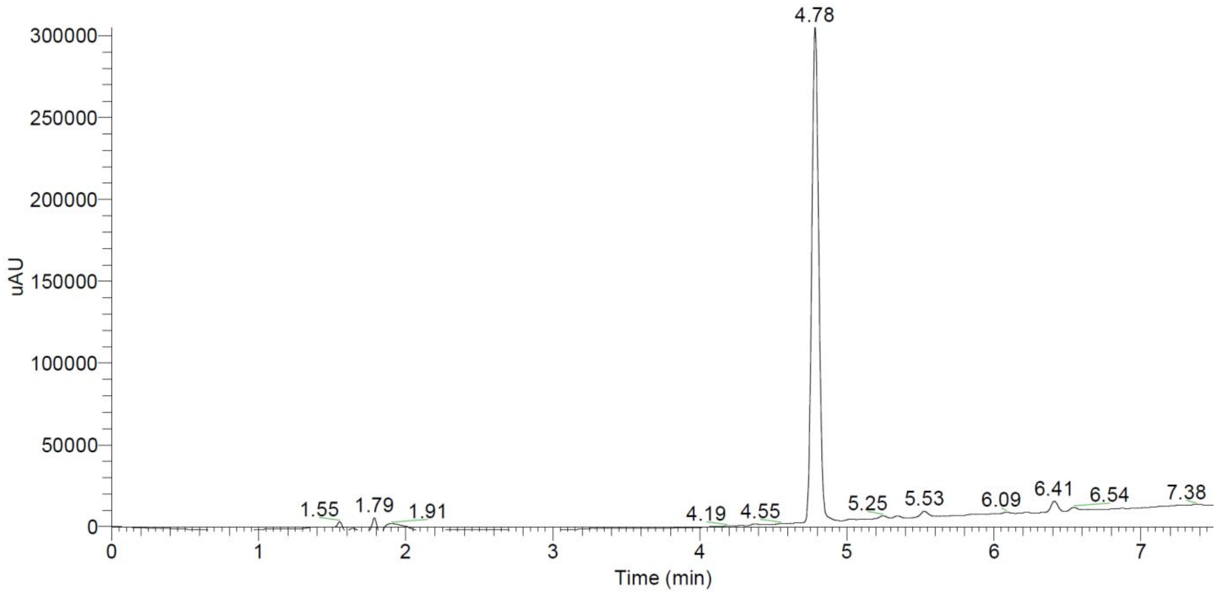

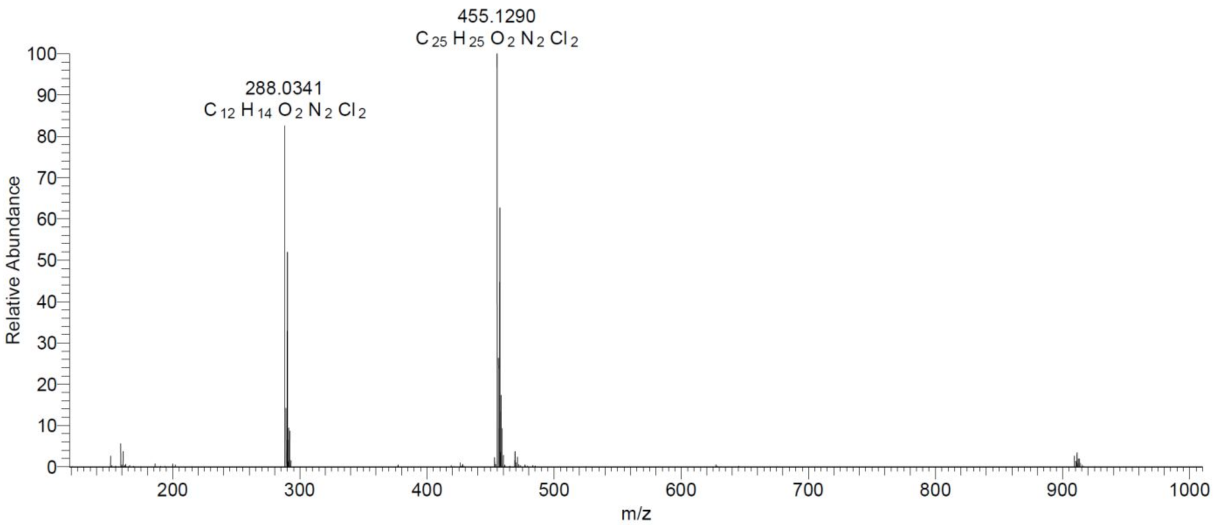

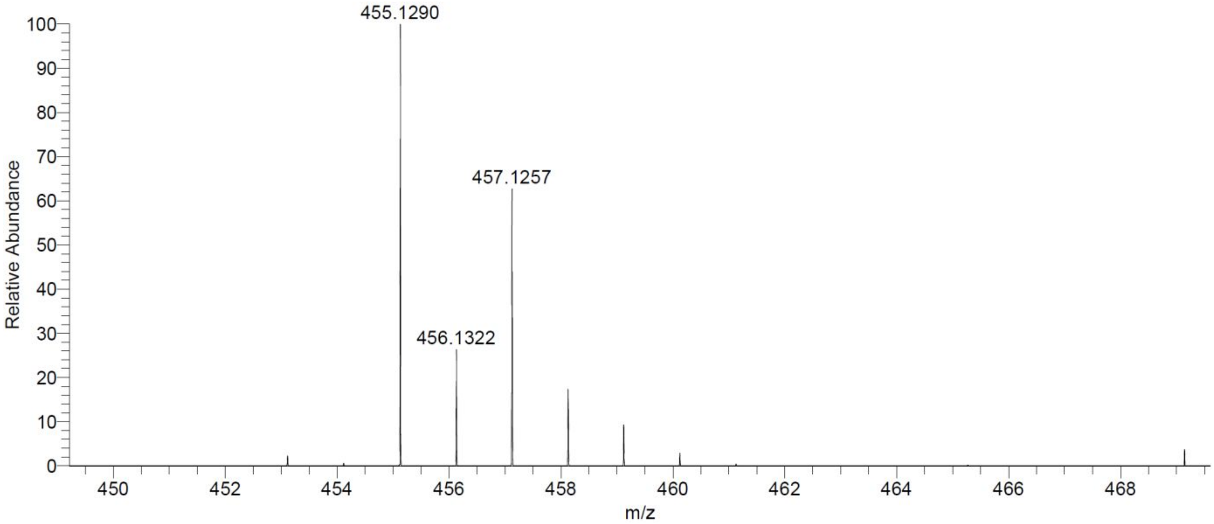

Spectrum 84: UV-trace and mass of main peak of indole **34**. Purity determined by peak area >95%.

Spectra of aminothiazoles derivatives

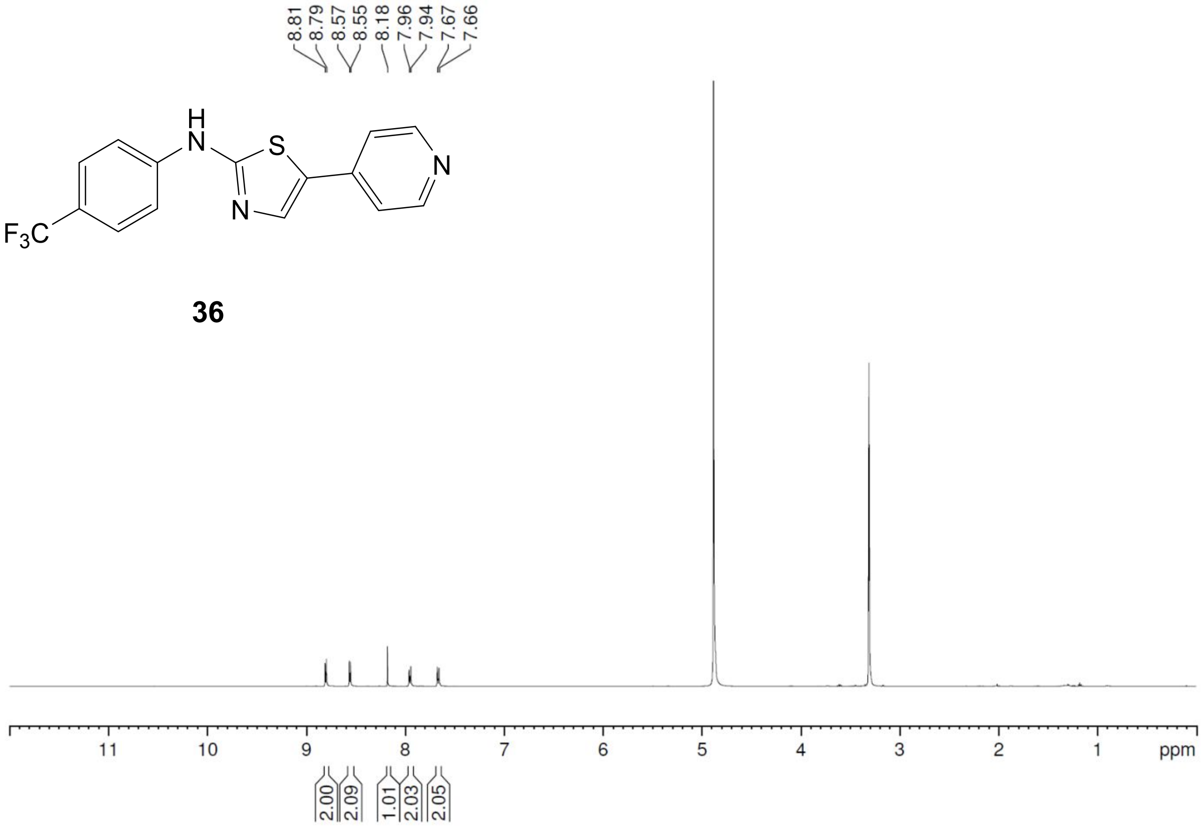

Spectrum 85: ^{1}H-NMR spectrum of aminothiazole **36** in $(CD_3)_2OS$.

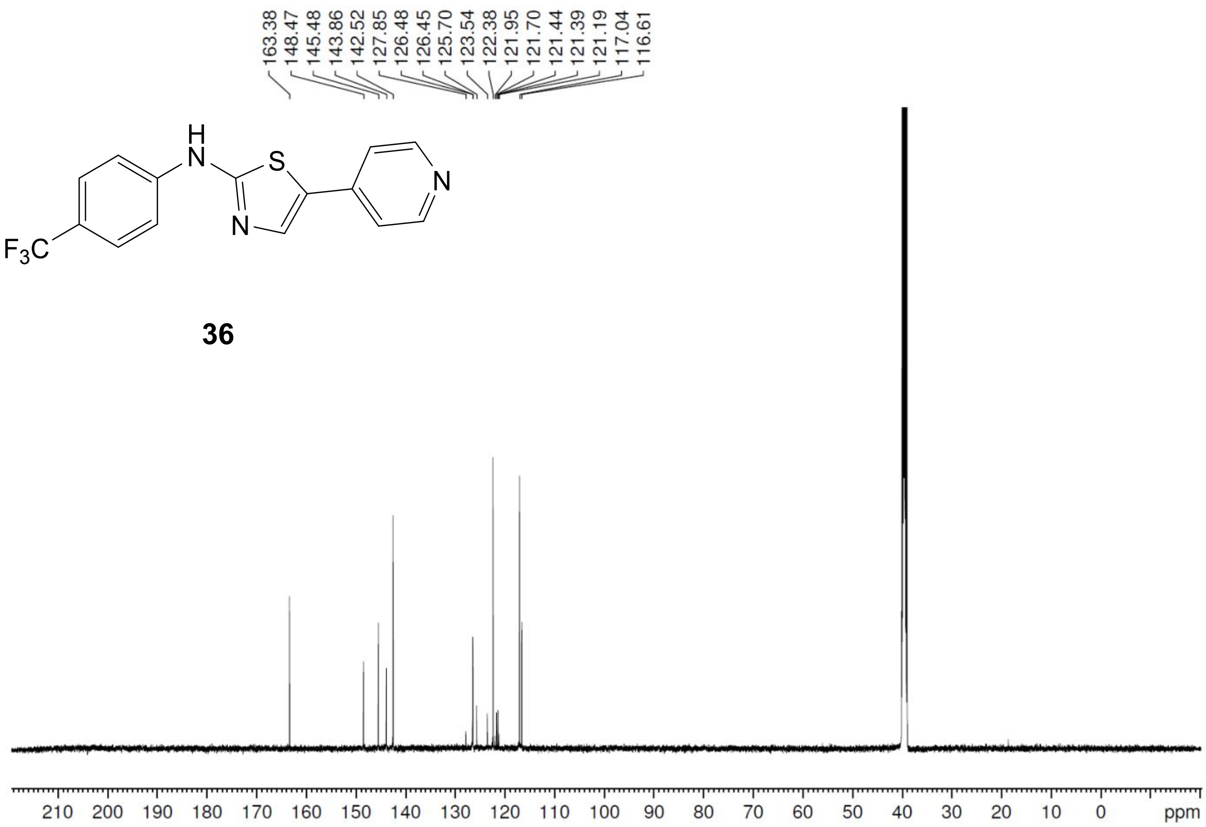

Spectrum 86: ^{13}C-NMR spectrum of aminothiazole **36** in $(CD_3)_2OS$.

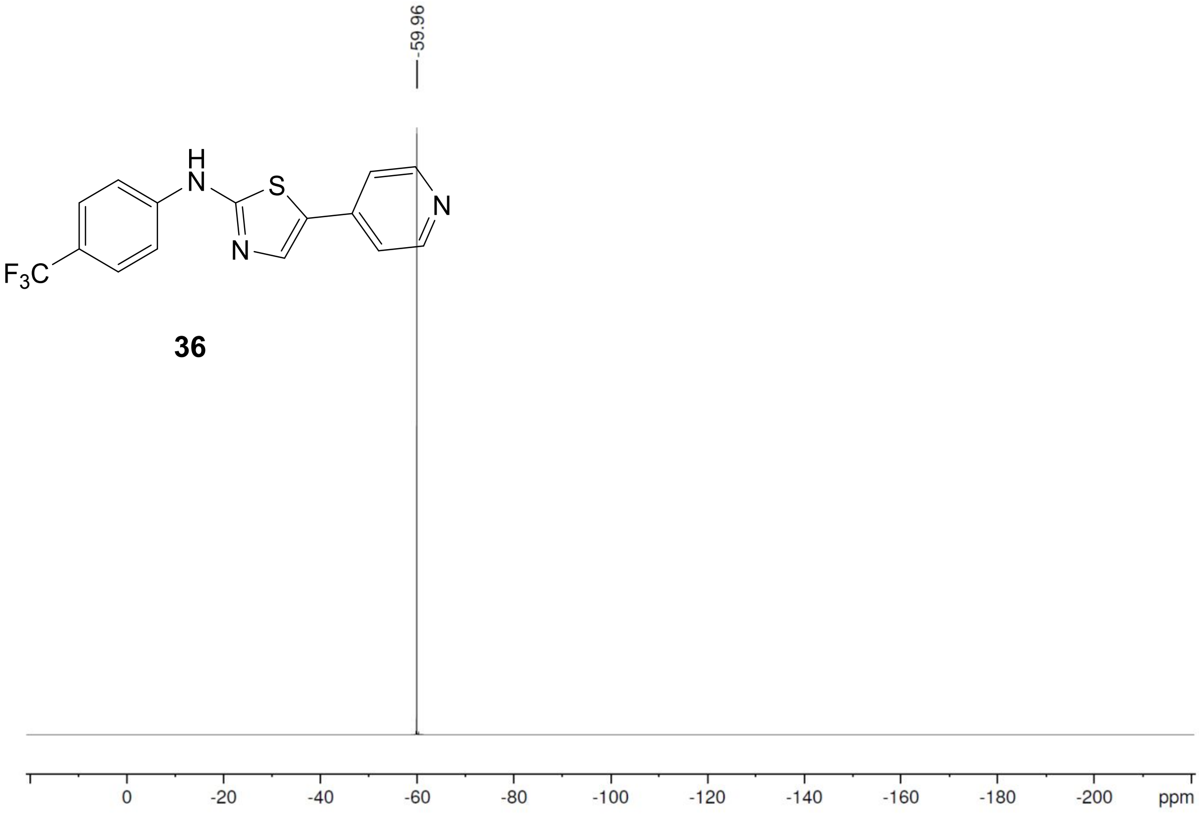

Spectrum 87: ^{19}F-NMR spectrum of aminothiazole **36** in $(CD_3)_2OS$.

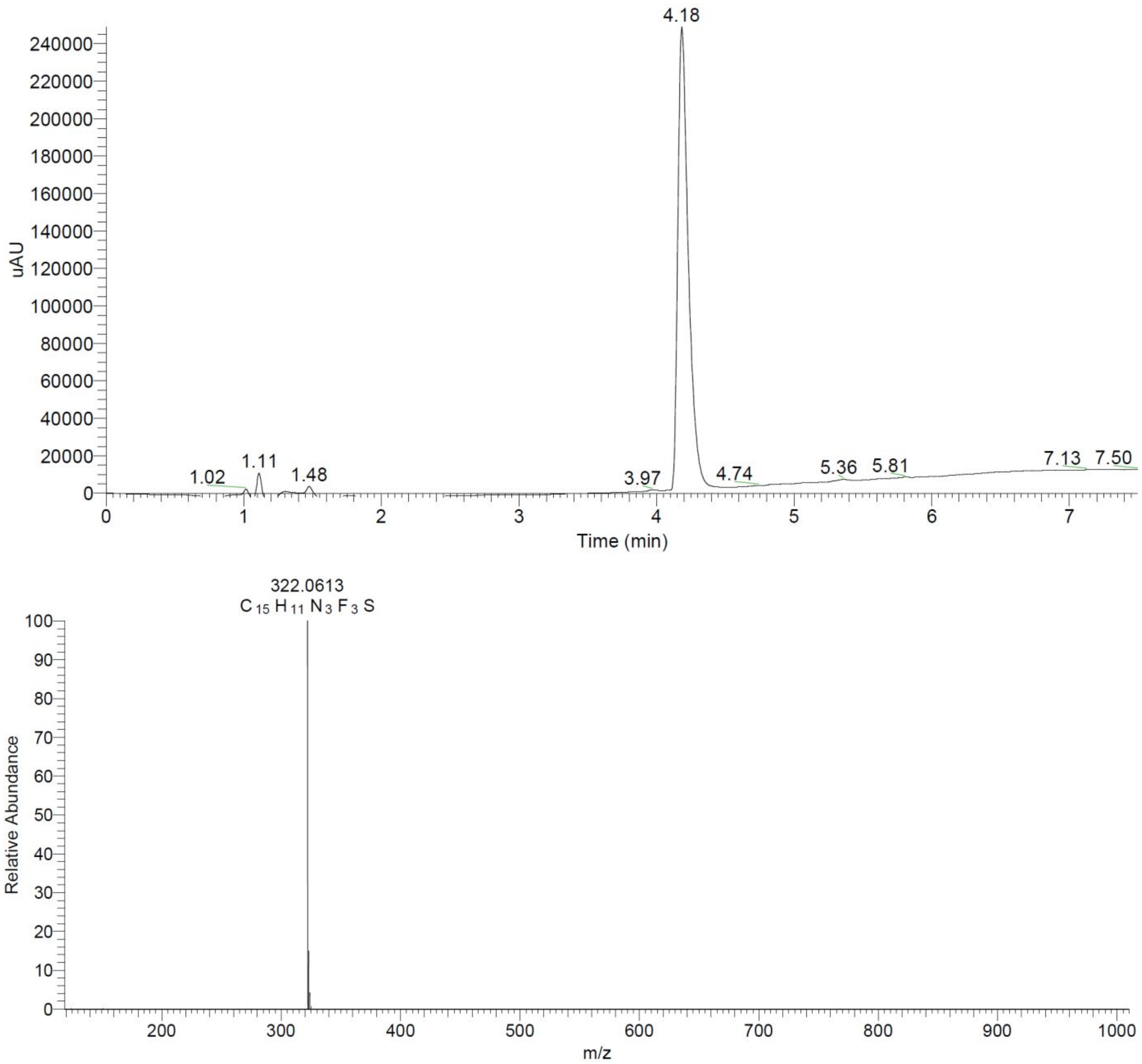

Spectrum 88: UV-trace and mass of main peak of aminothiazole **36**. Purity determined by peak area 99%.

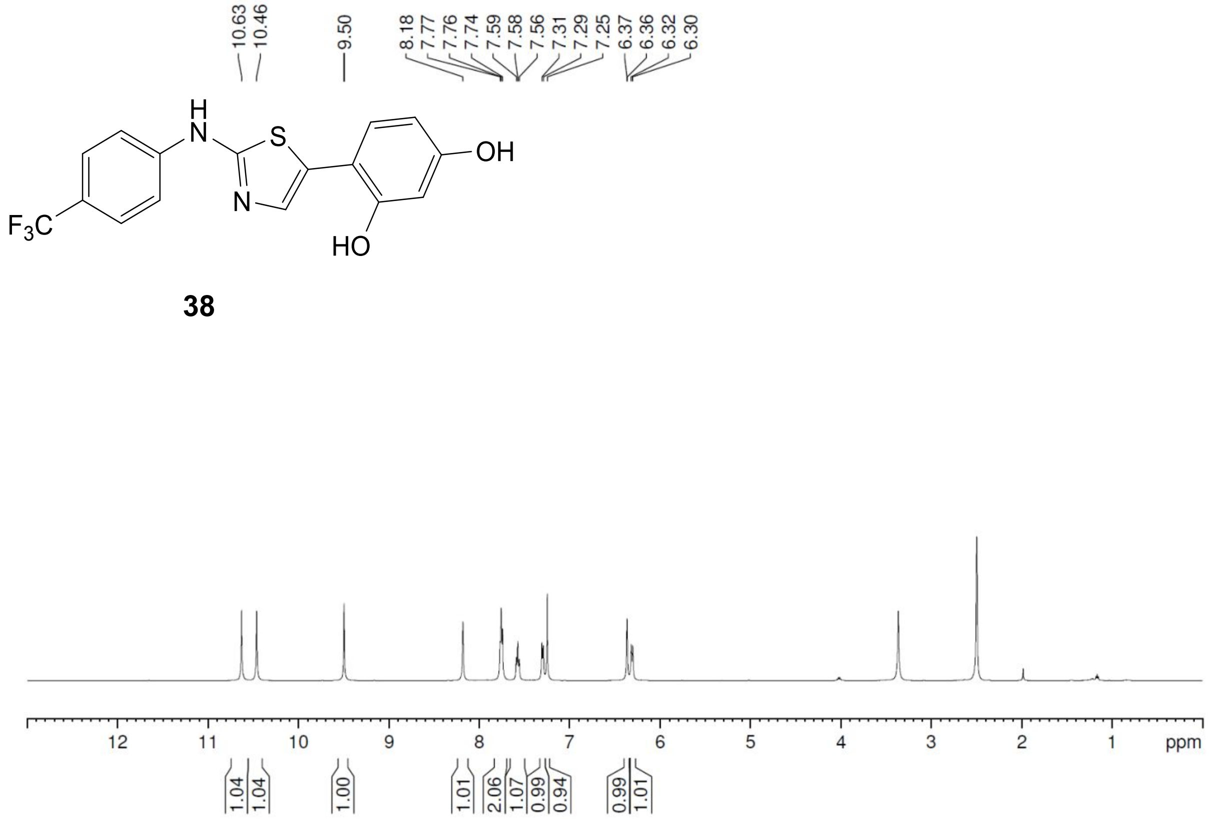

Spectrum 89: ^{1}H-NMR spectrum of aminothiazole **38** in $(CD_3)_2OS$.

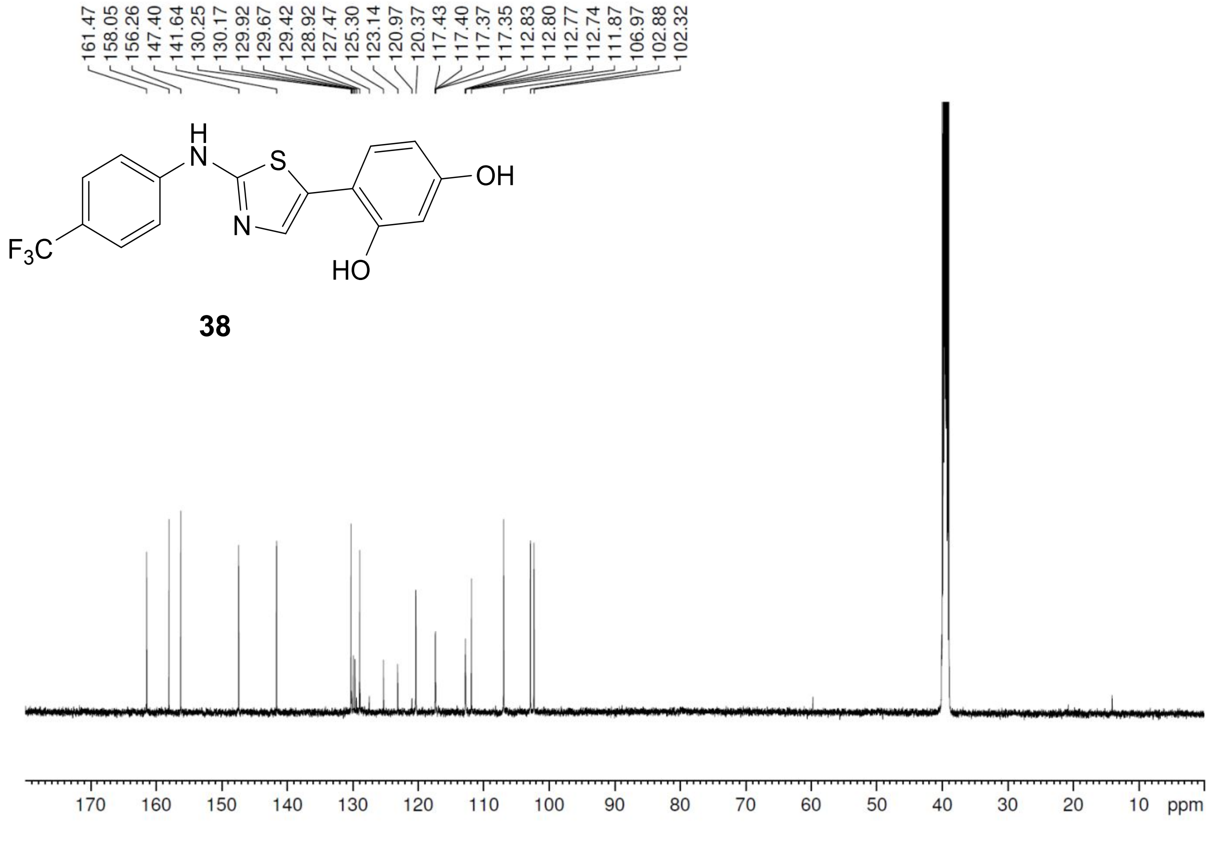

Spectrum 90: ^{13}C-NMR spectrum of aminothiazole **38** in $(CD_3)_2OS$.

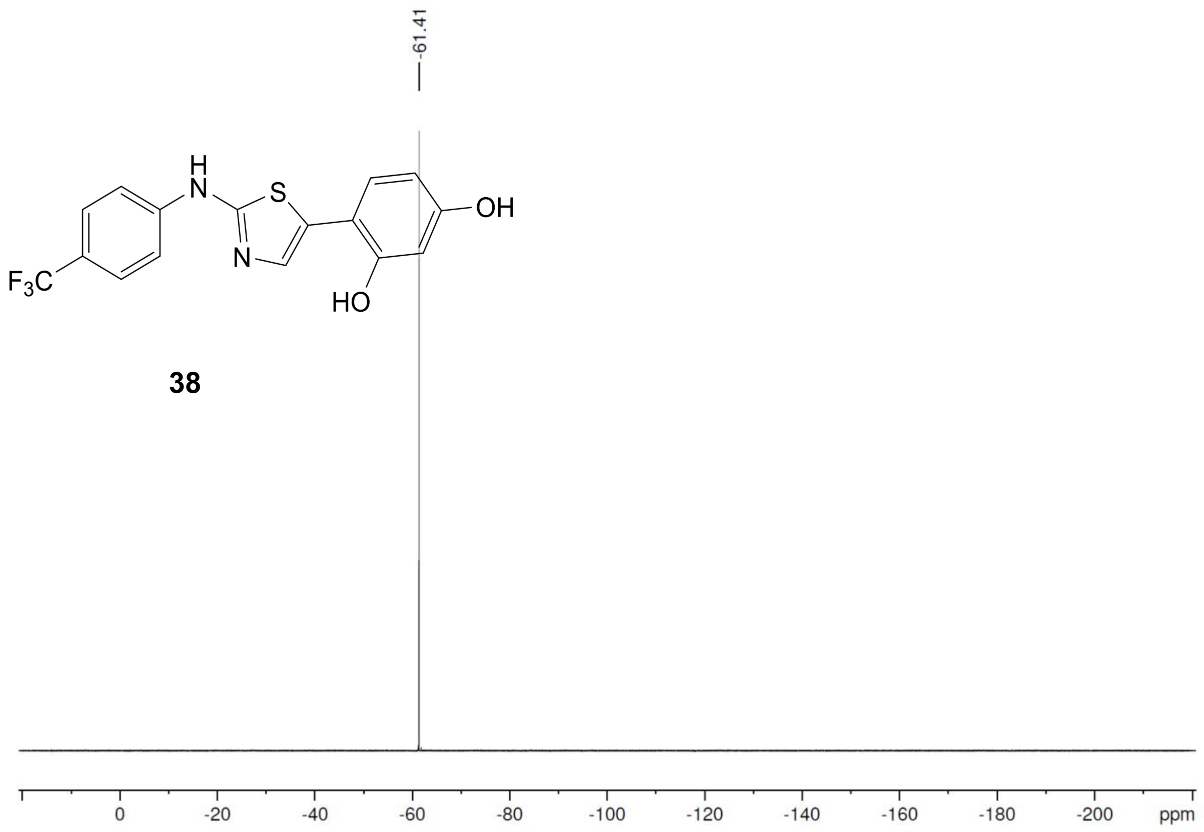

Spectrum 91: ^{19}F-NMR spectrum of aminothiazole **38** in $(CD_3)_2OS$.

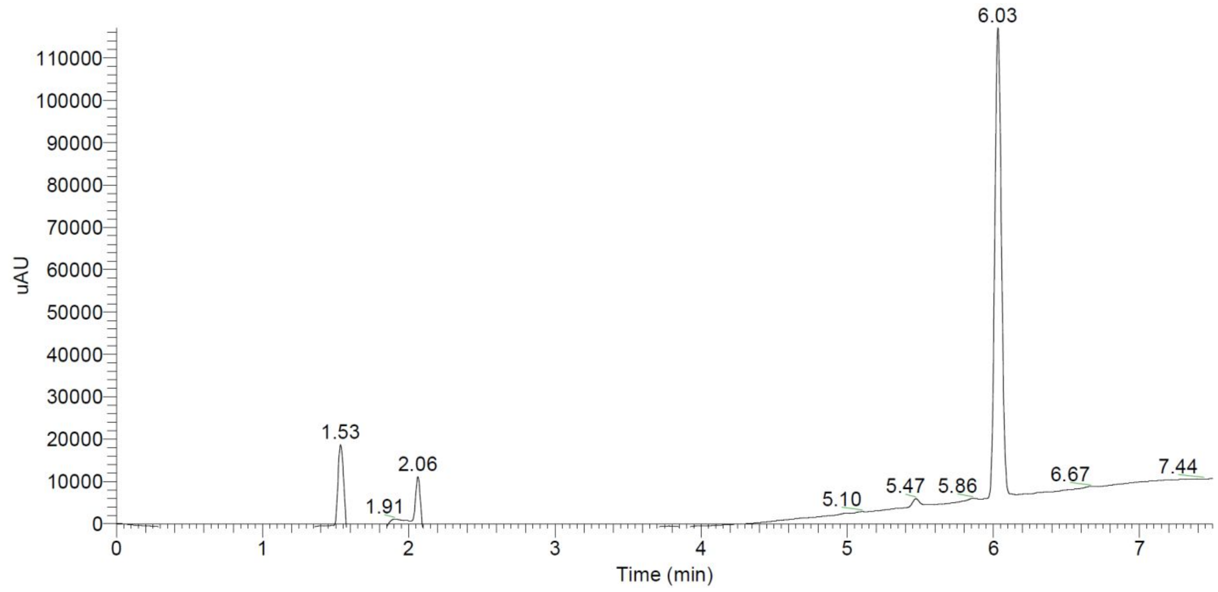

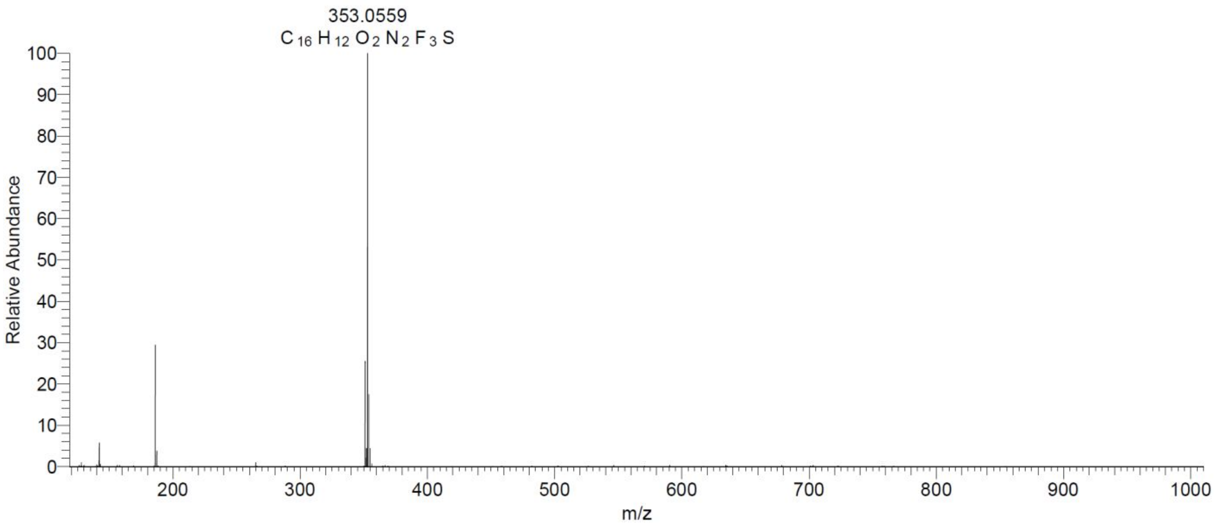

Spectrum 92: UV-trace and mass of main peak of aminothiazole **38**. Purity determined by peak area 96%.

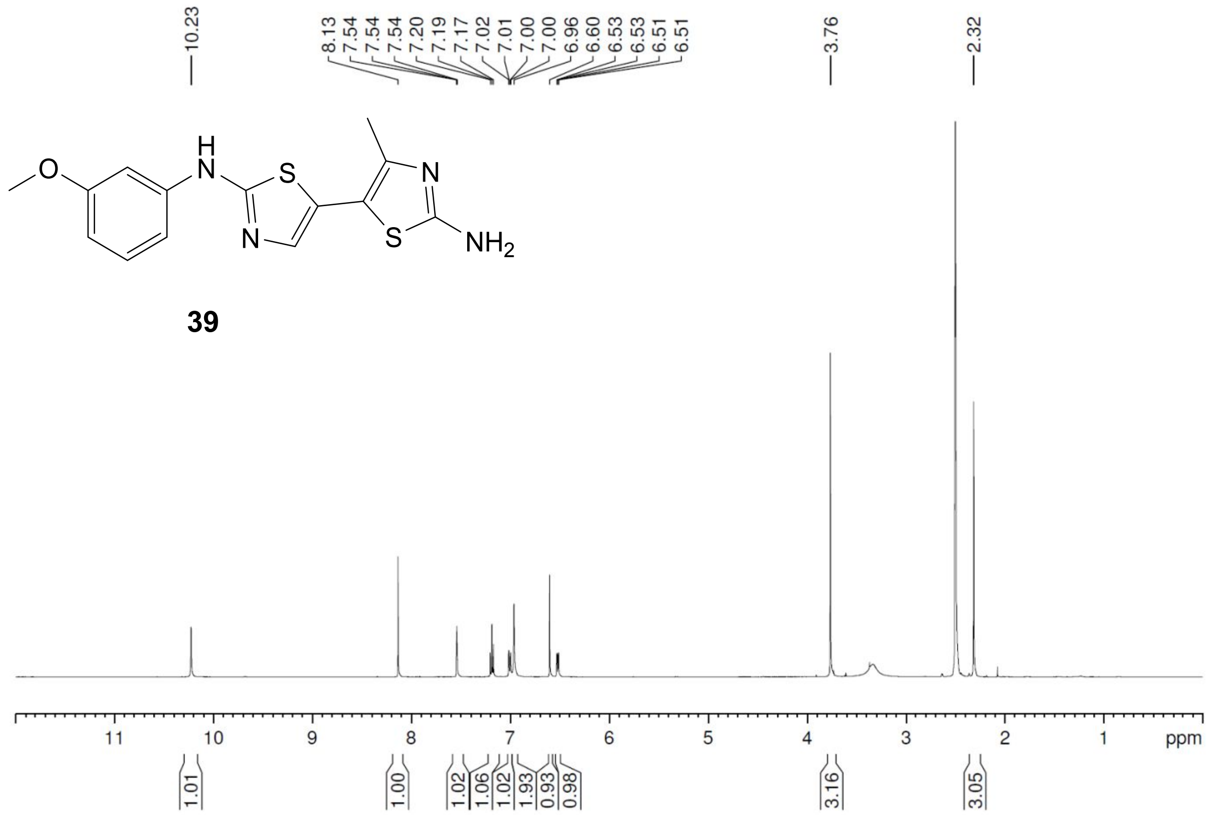

Spectrum 93: ^{1}H-NMR spectrum of aminothiazole **39** in $(CD_3)_2OS$.

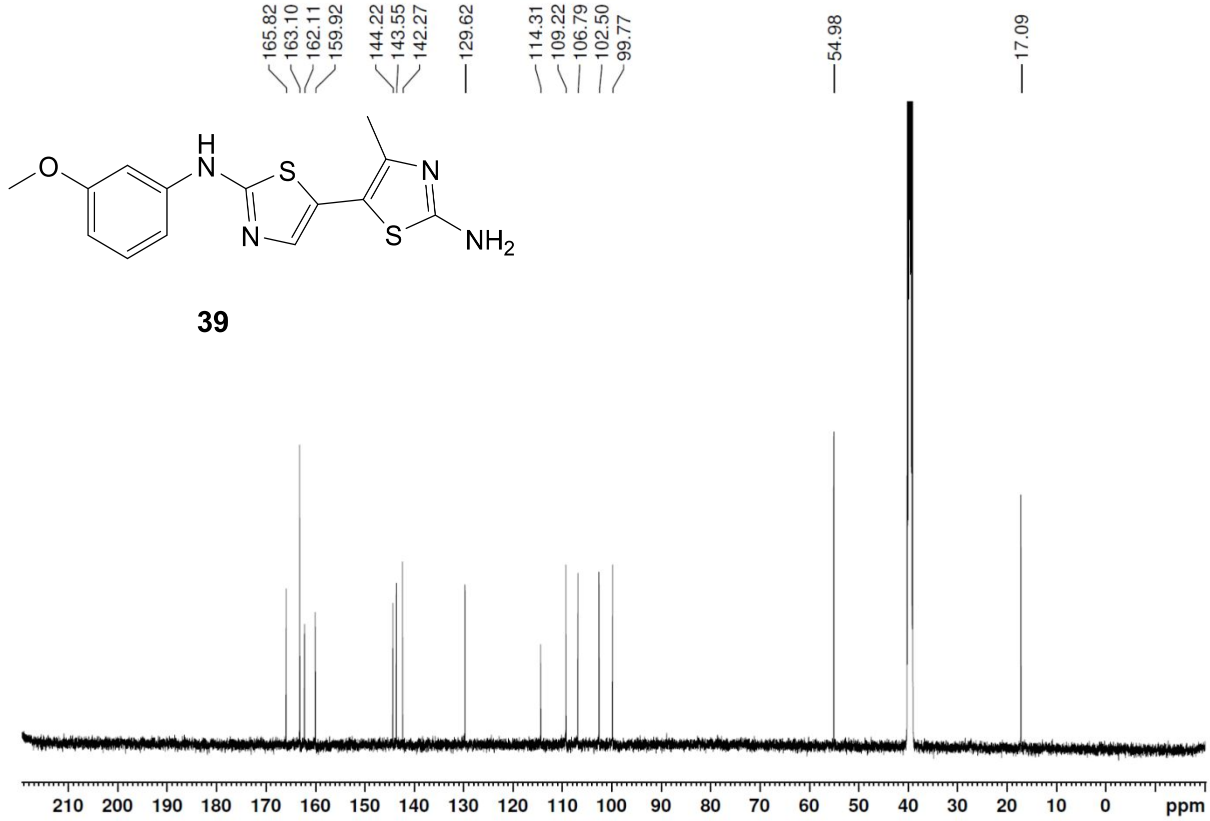

Spectrum 94: ^{13}C-NMR spectrum of aminothiazole **39** in $(CD_3)_2OS$.

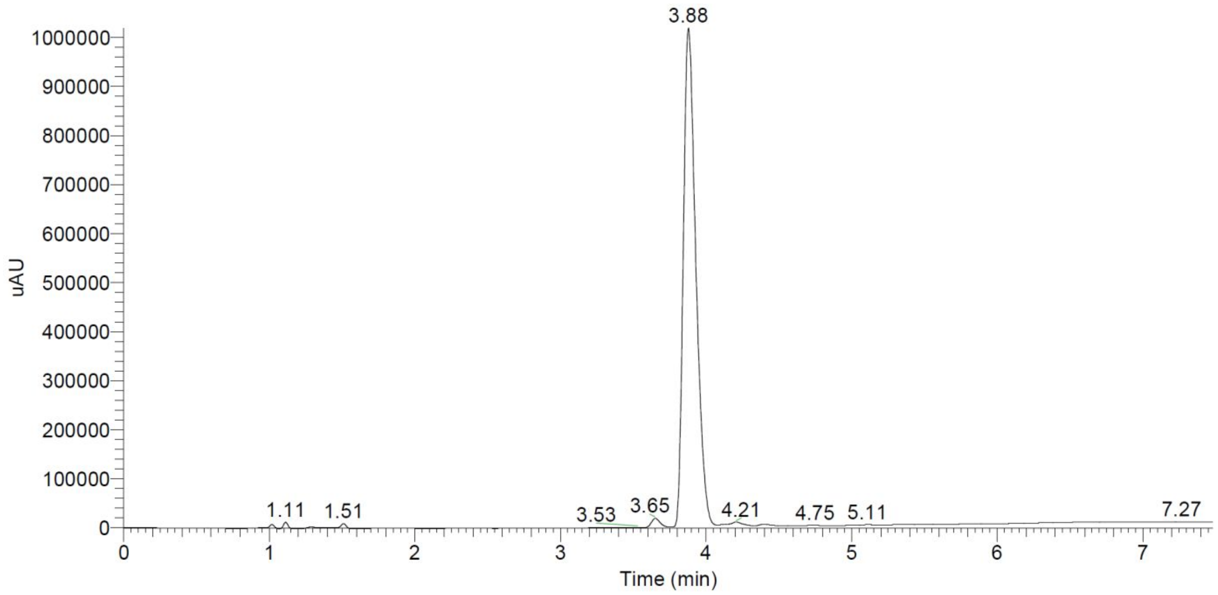

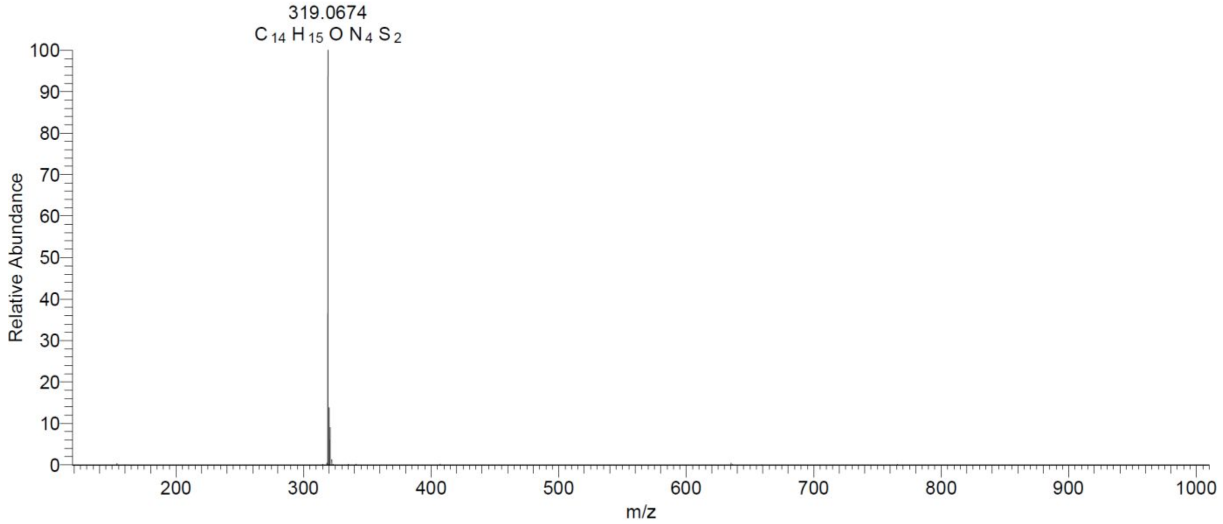

Spectrum 95: UV-trace and mass of main peak of aminothiazole **39**. Purity determined by peak area 95%.

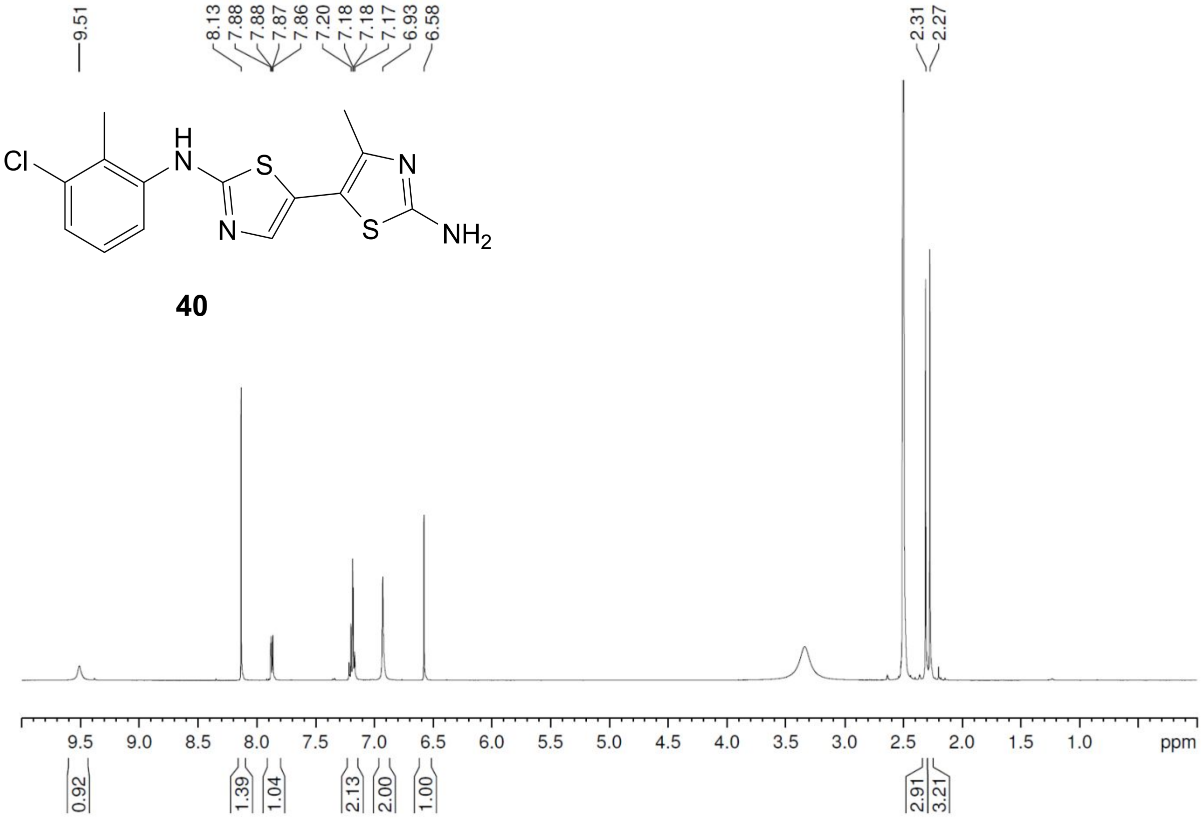

Spectrum 96: ^{1}H-NMR spectrum of aminothiazole **40** in $(CD_3)_2OS$.

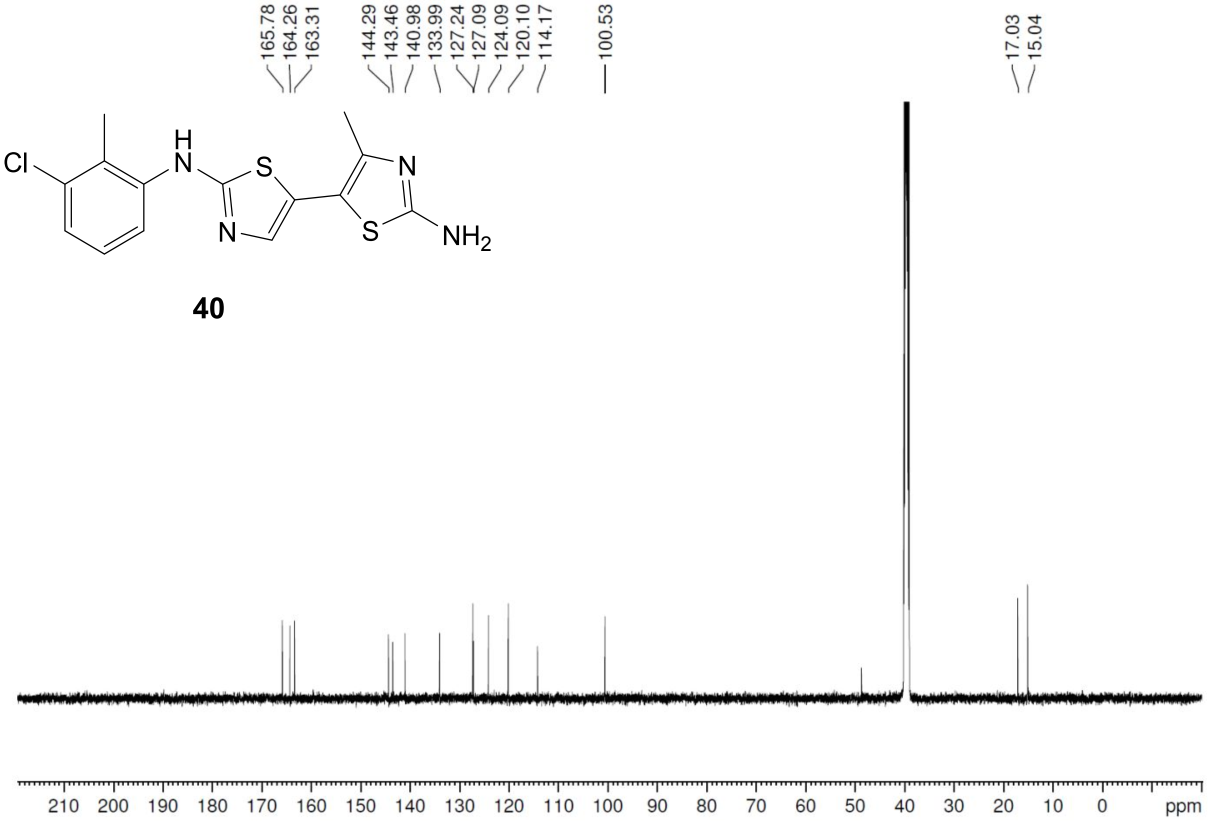

Spectrum 97: ^{13}C-NMR spectrum of aminothiazole **40** in $(CD_3)_2OS$.

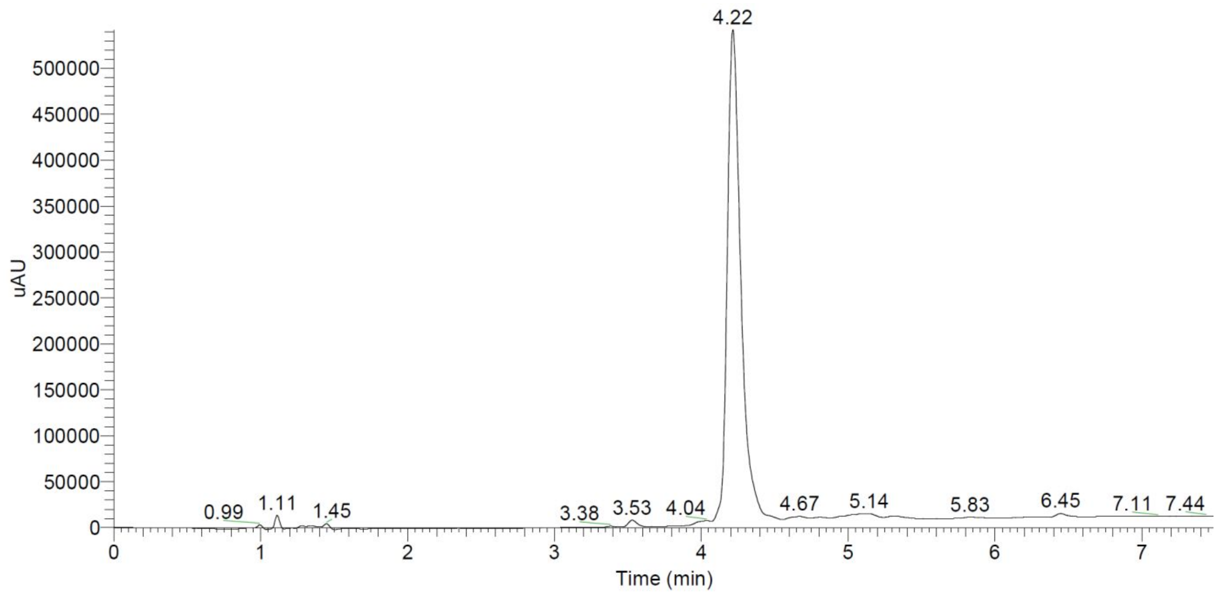

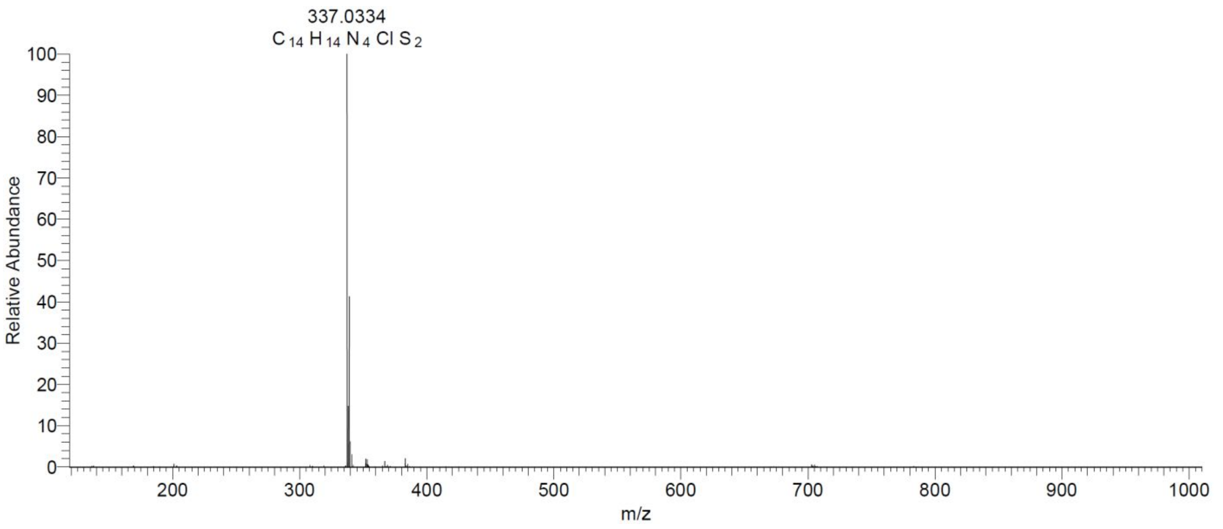

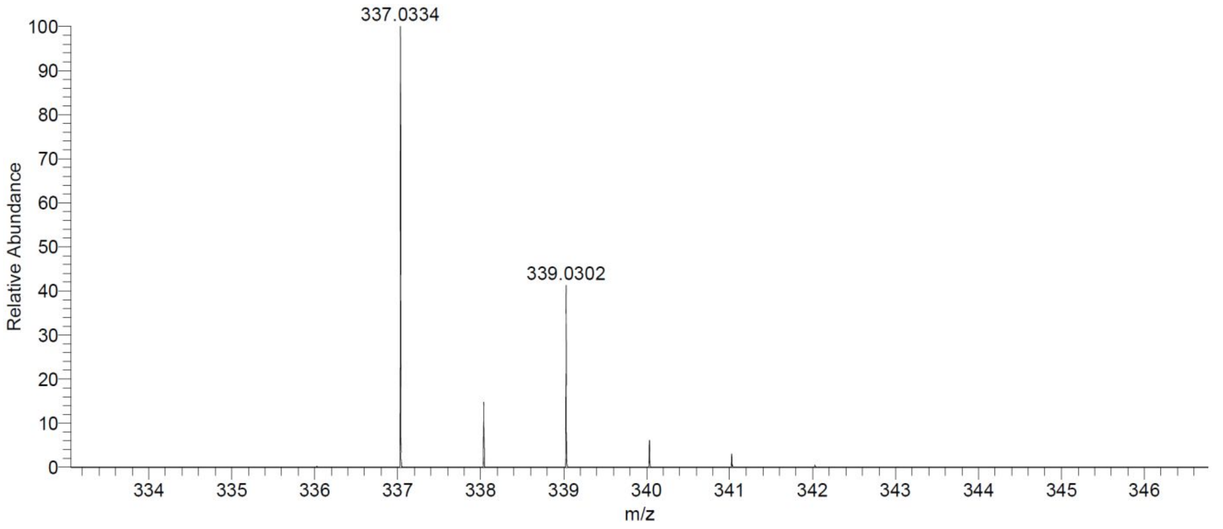

Spectrum 98: UV-trace and mass of main peak of aminothiazole **40**. Purity determined by peak area 97%.

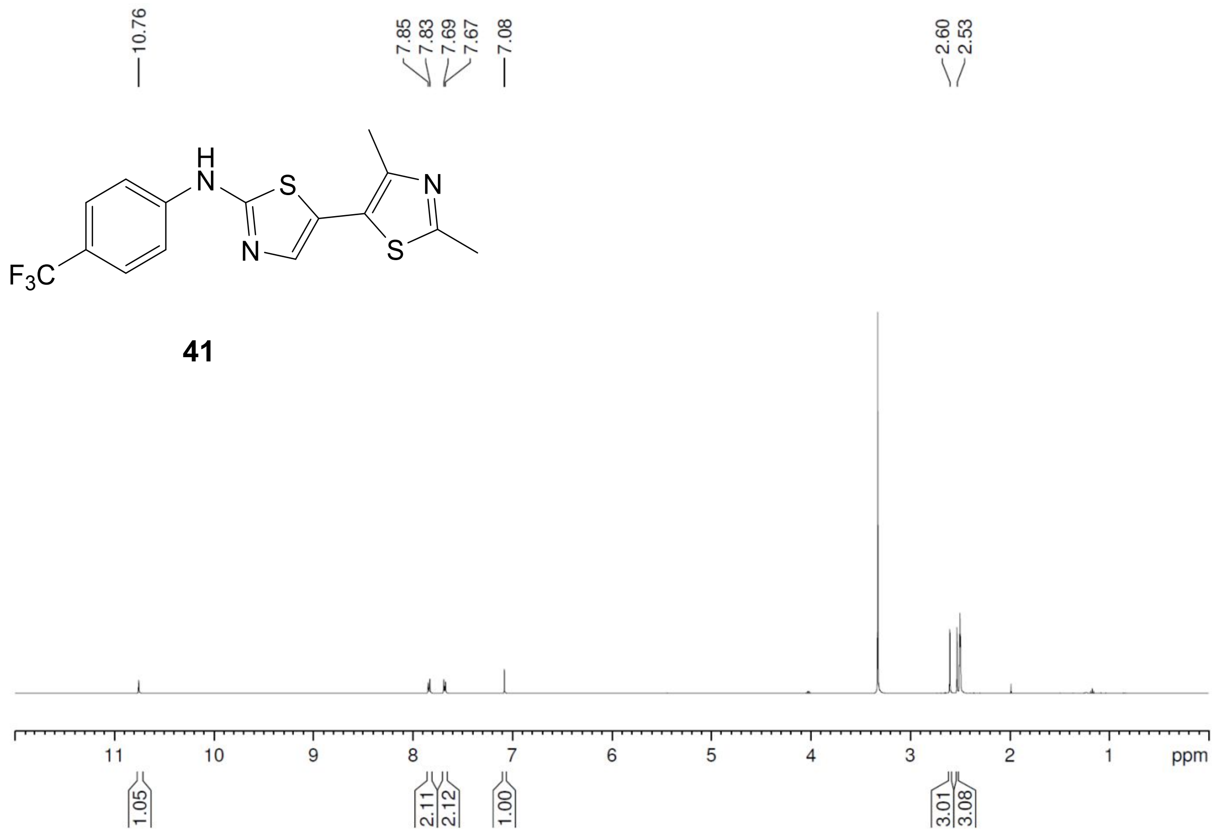

Spectrum 99: ^{1}H-NMR spectrum of aminothiazole **41** in $(CD_3)_2OS$.

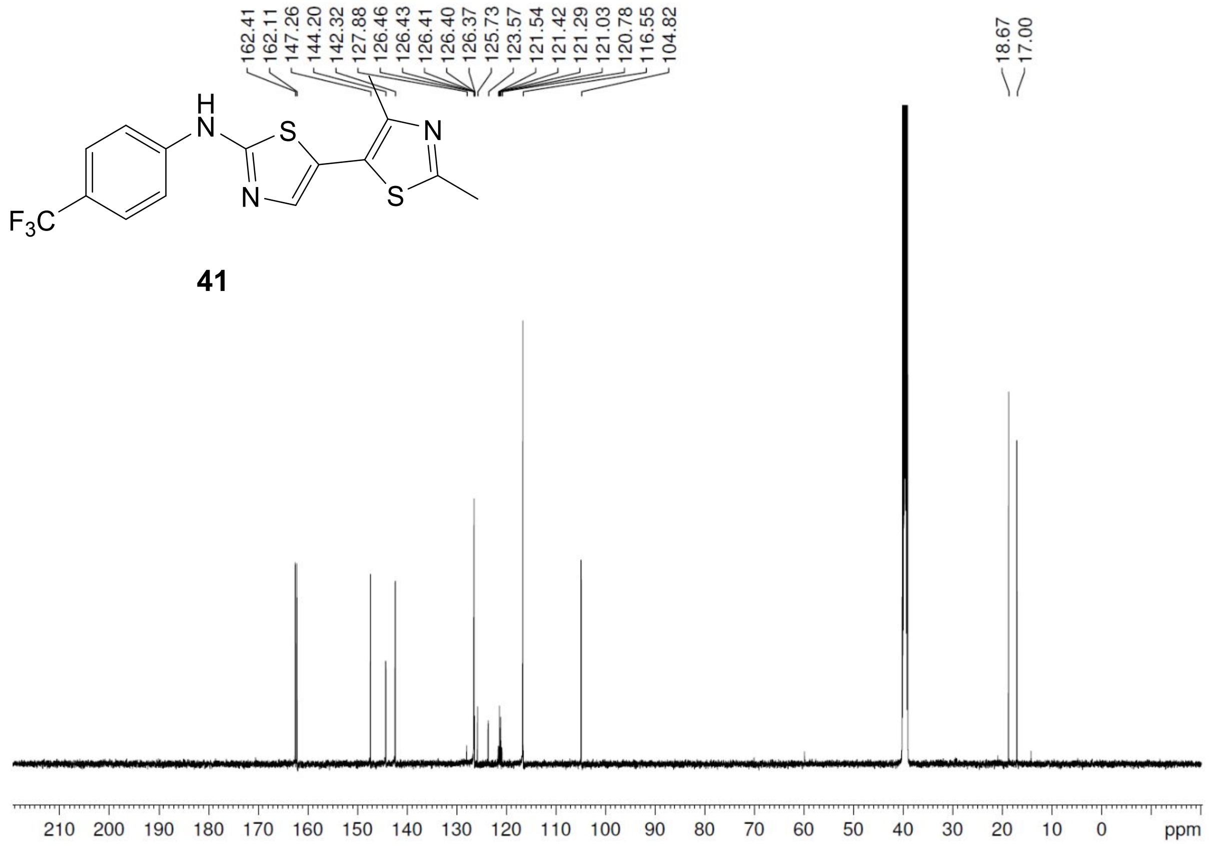

Spectrum 100: ^{13}C-NMR spectrum of aminothiazole **41** in $(CD_3)_2OS$.

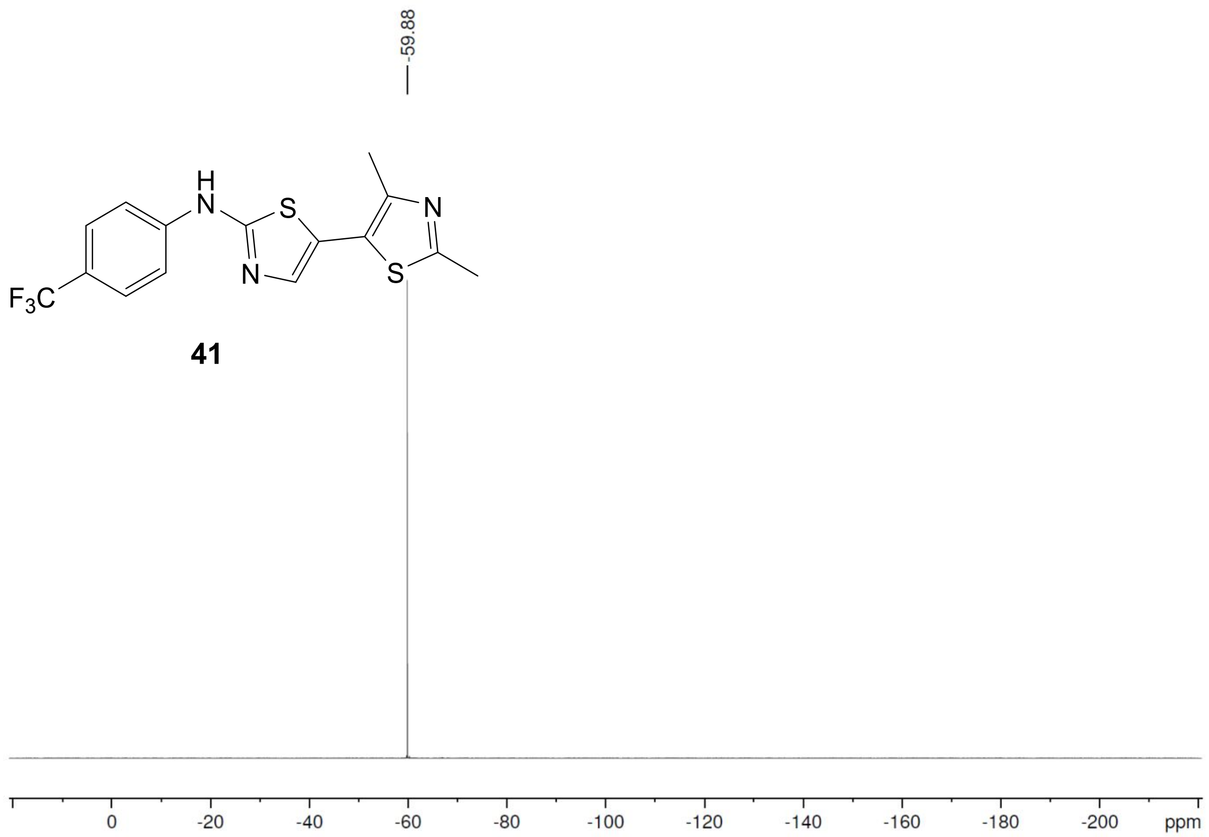

41

Spectrum 101: ^{19}F-NMR spectrum of aminothiazole **41** in $(CD_3)_2OS$.

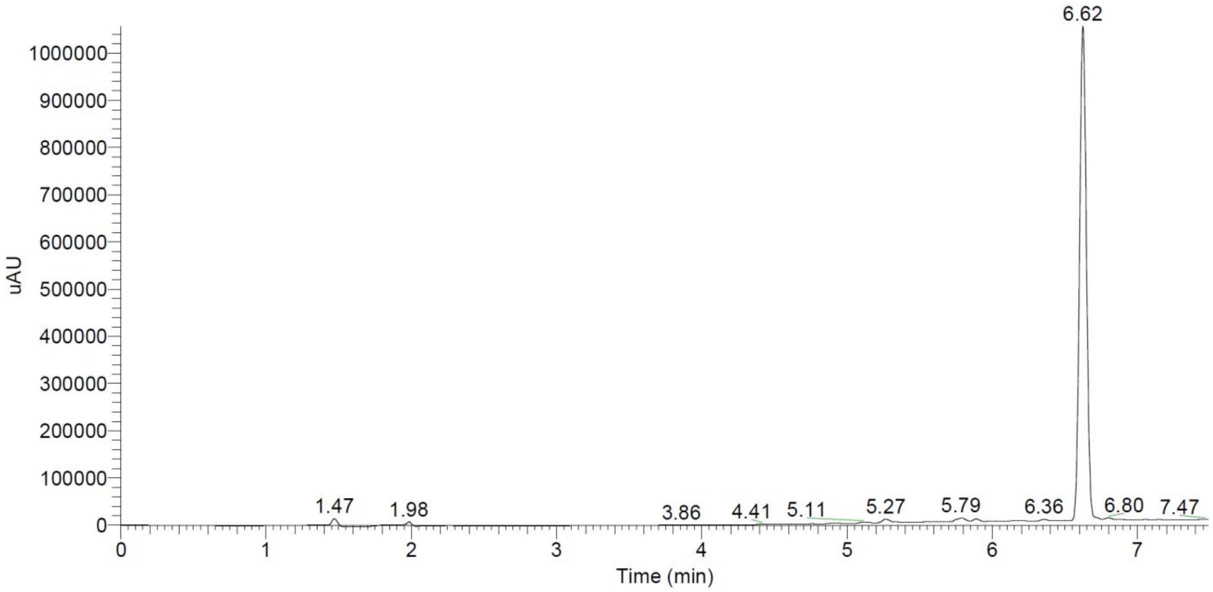

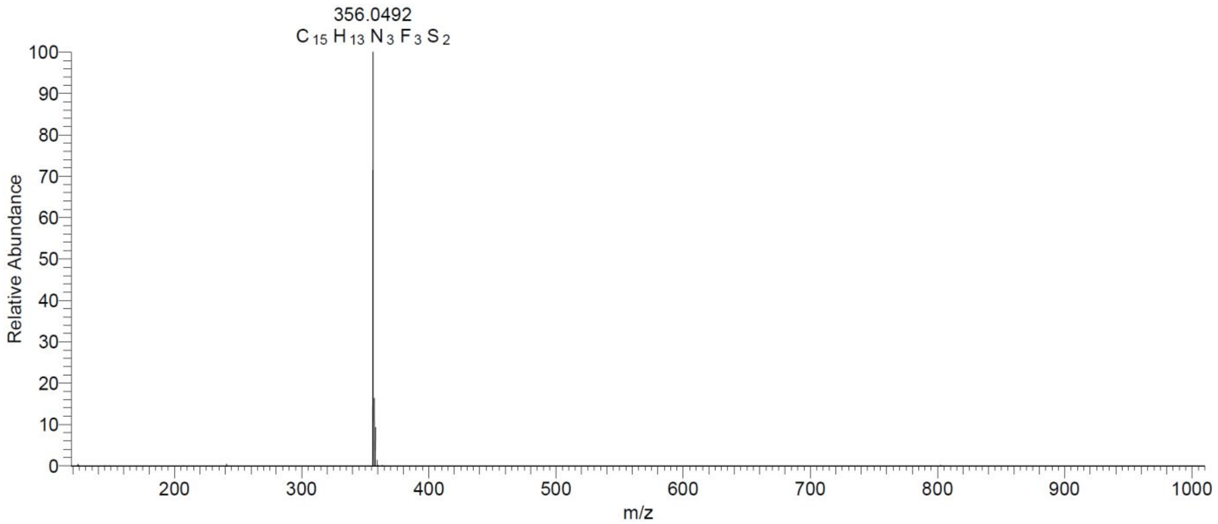

Spectrum 102: UV-trace and mass of main peak of aminothiazole **41**. Purity determined by peak area 98%.

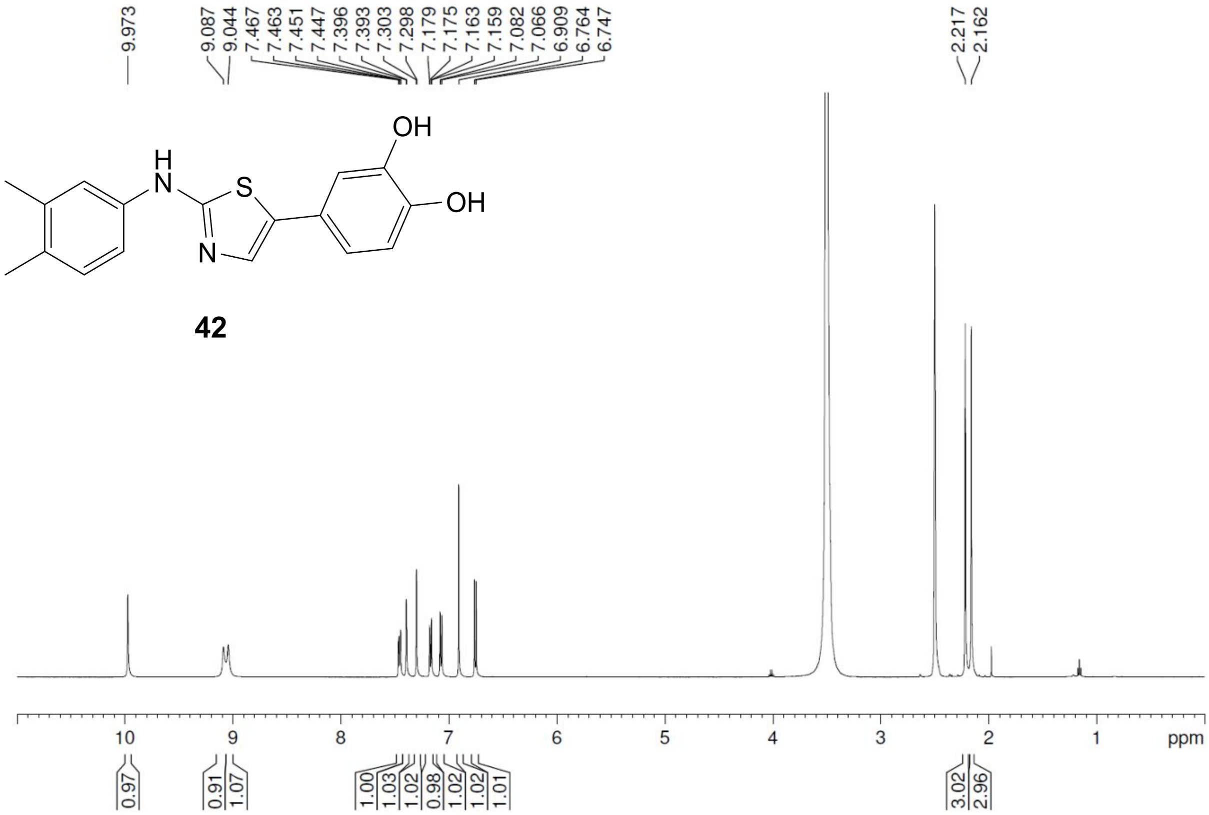

Spectrum 103: ^{1}H-NMR spectrum of aminothiazole **42** in $(CD_3)_2OS$.

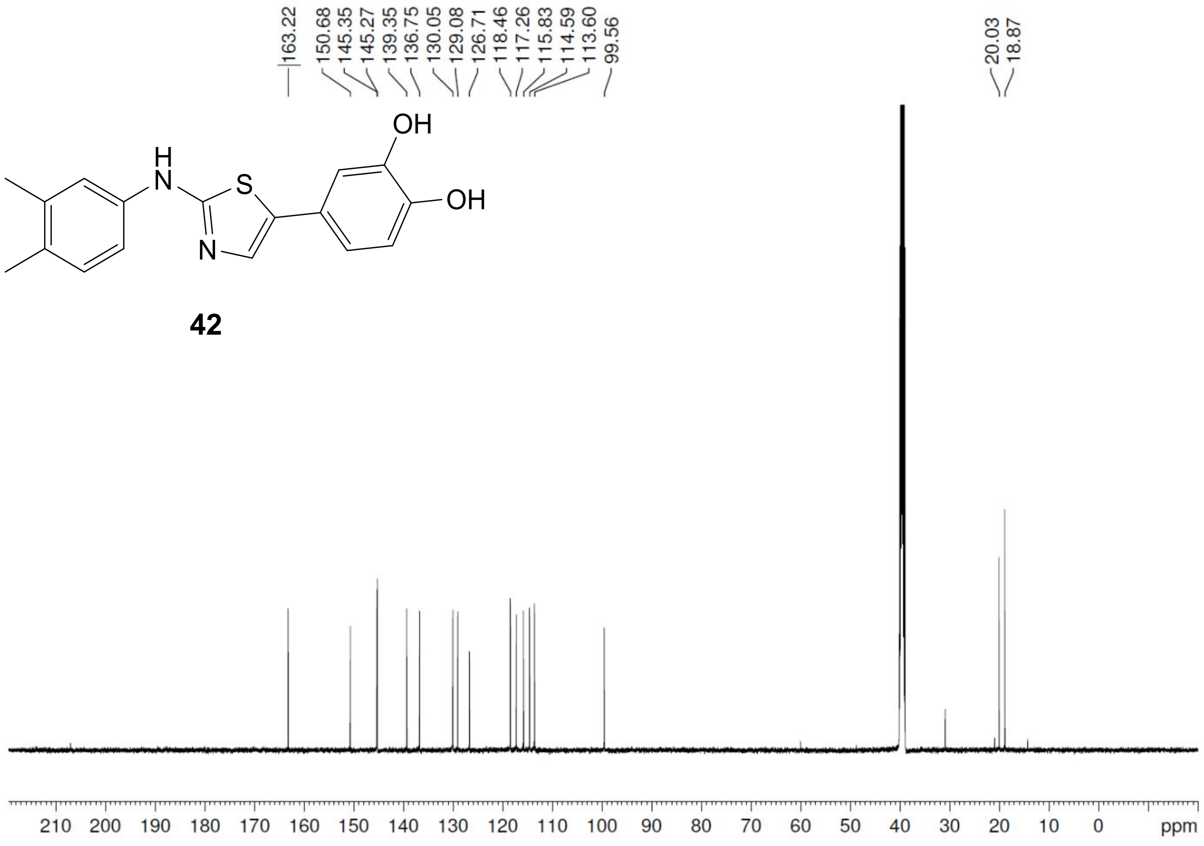

Spectrum 104: ^{13}C-NMR spectrum of aminothiazole **42** in $(CD_3)_2OS$.

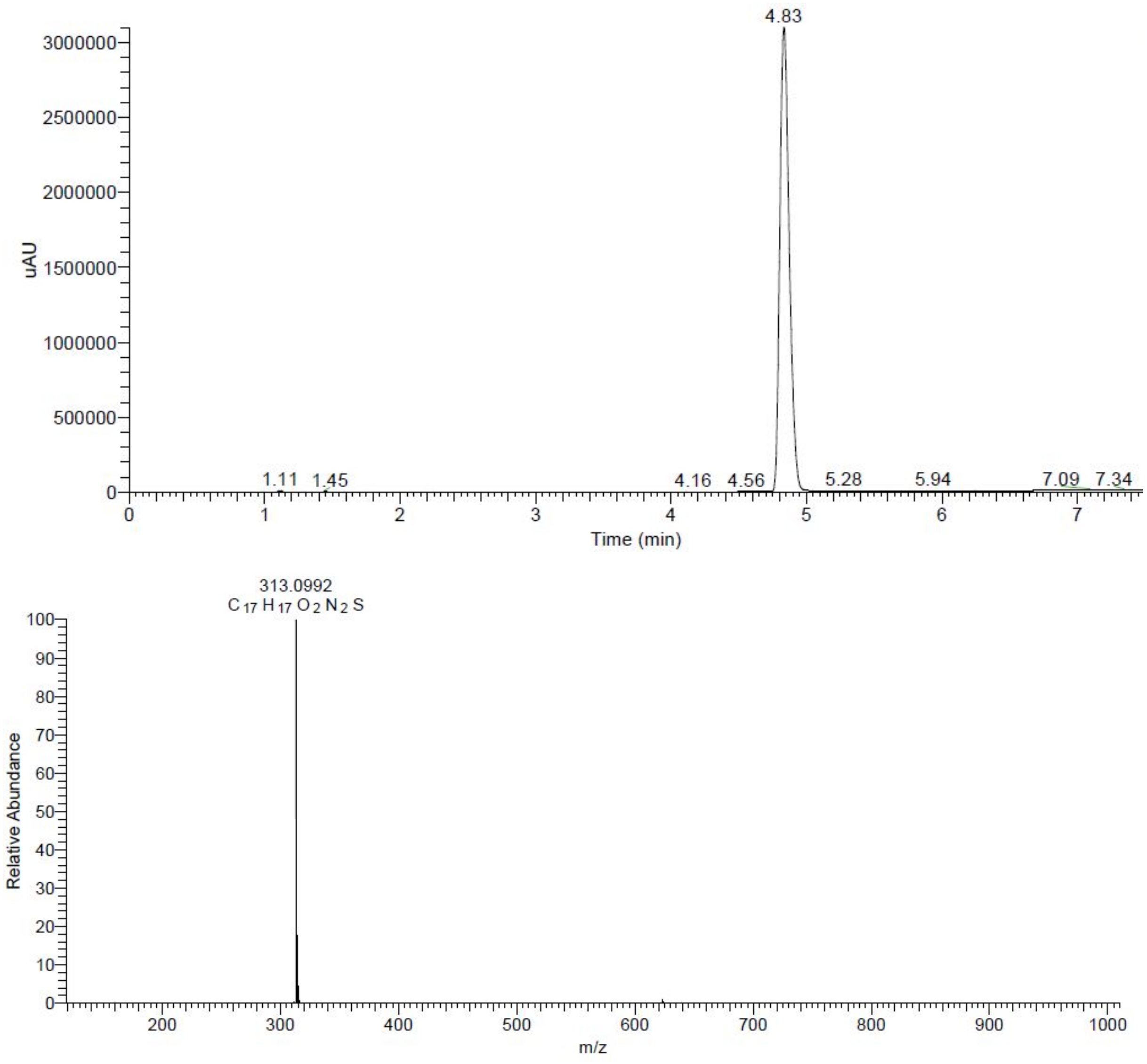

Spectrum 105: UV-trace and mass of main peak of aminothiazole **42**. Purity determined by peak area 99%.

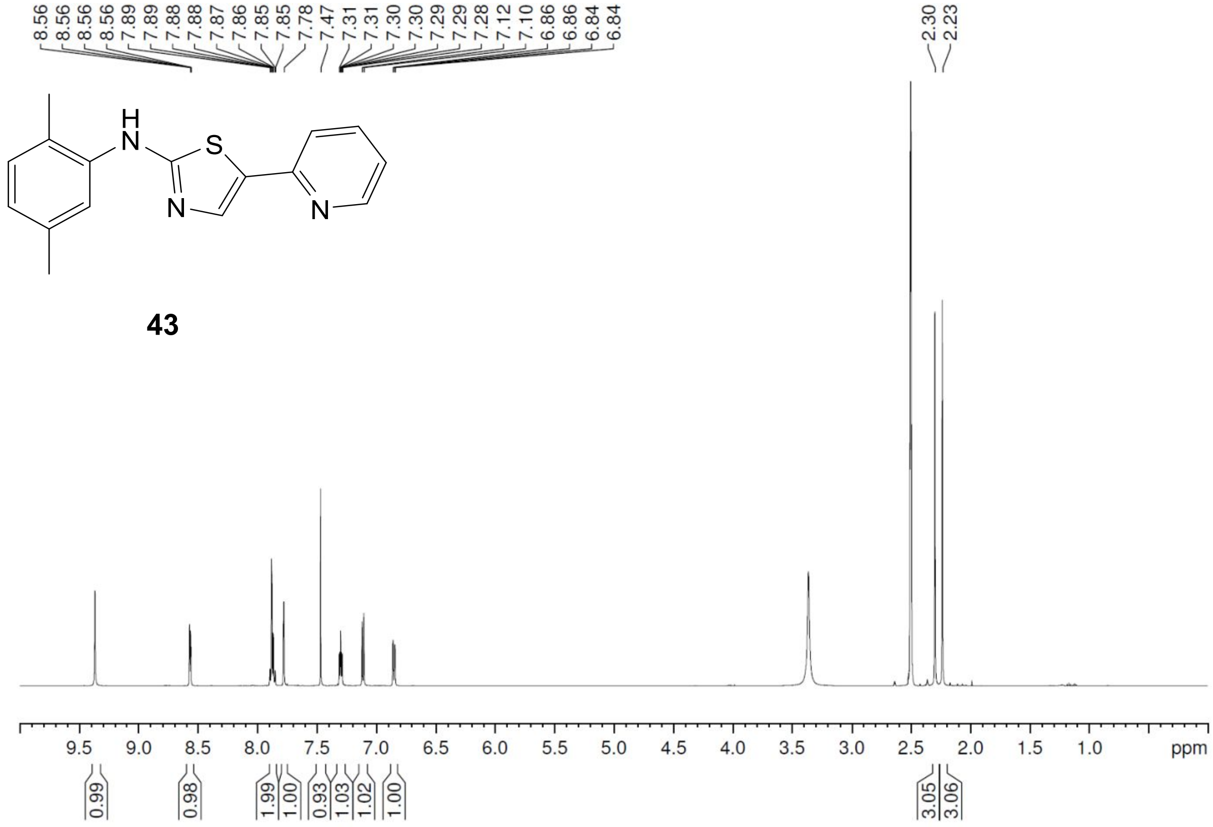

Spectrum 106: ^{1}H-NMR spectrum of aminothiazole **43** in $(CD_3)_2OS$.

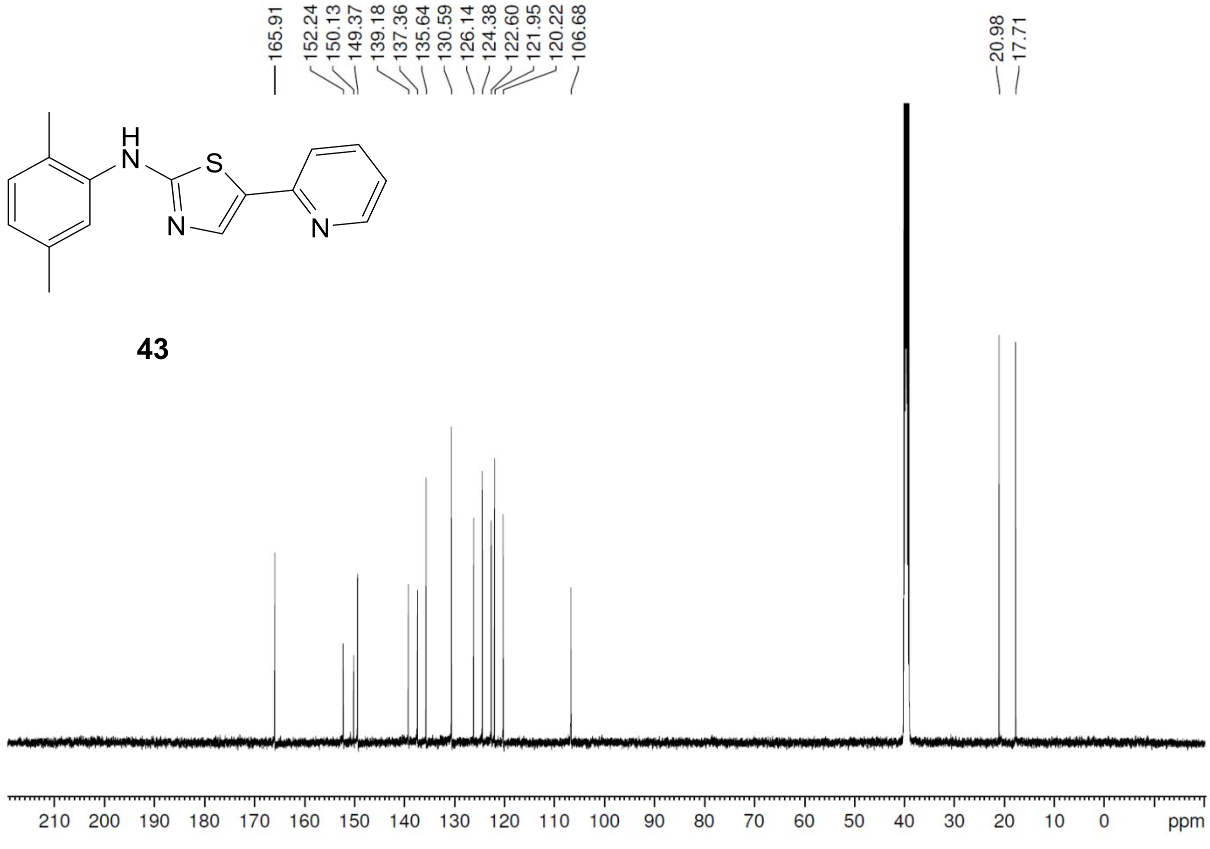

Spectrum 107: ^{13}C-NMR spectrum of aminothiazole **43** in $(CD_3)_2OS$.

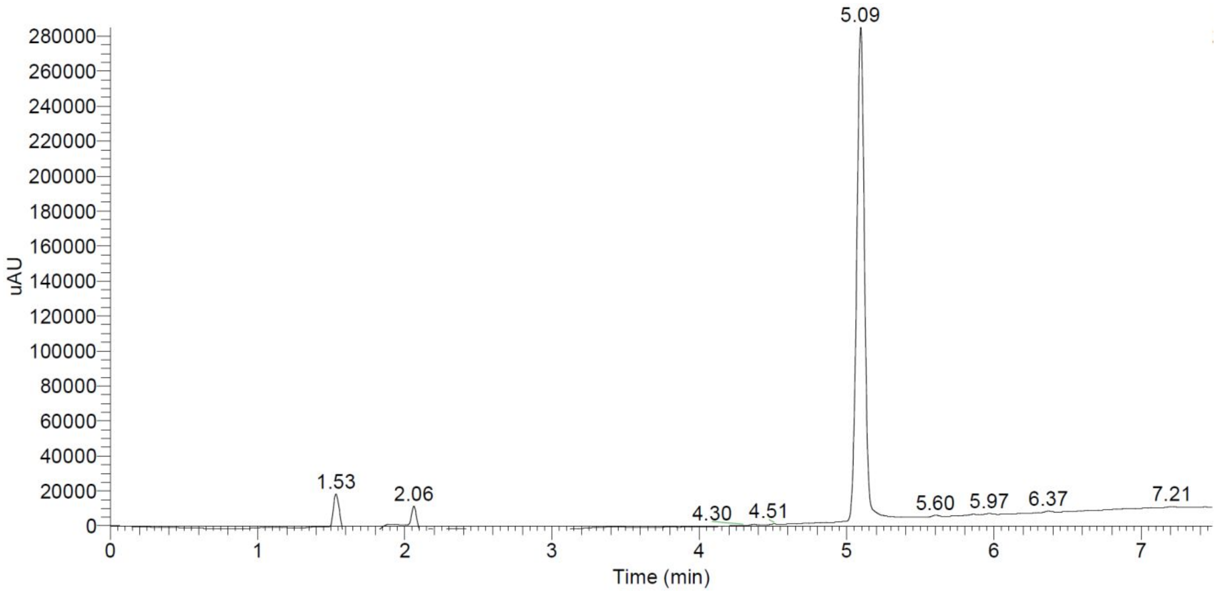

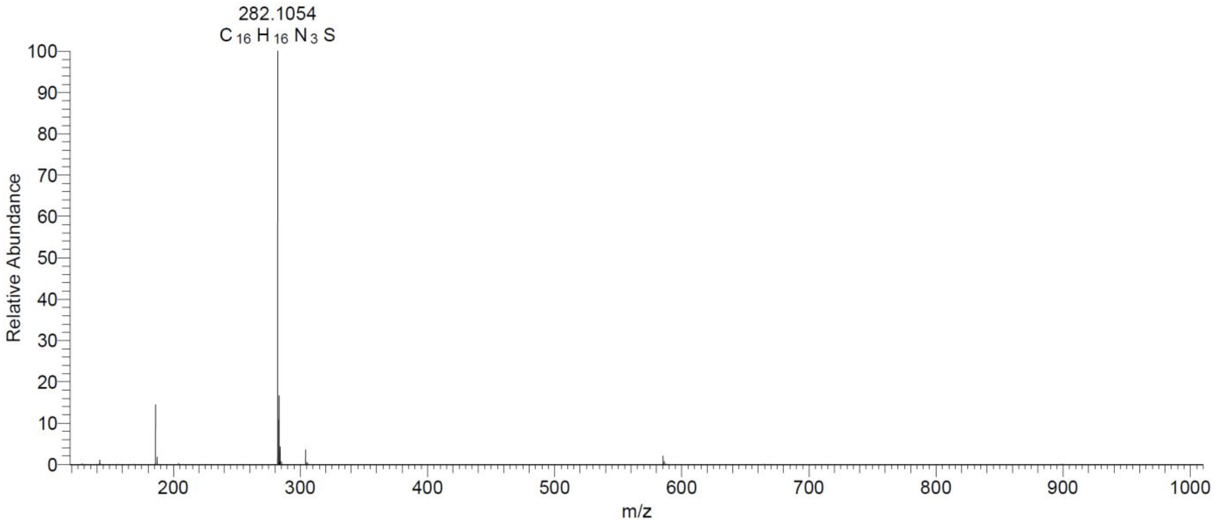

Spectrum 108: UV-trace and mass of main peak of aminothiazole **43**. Purity determined by peak area 99%.

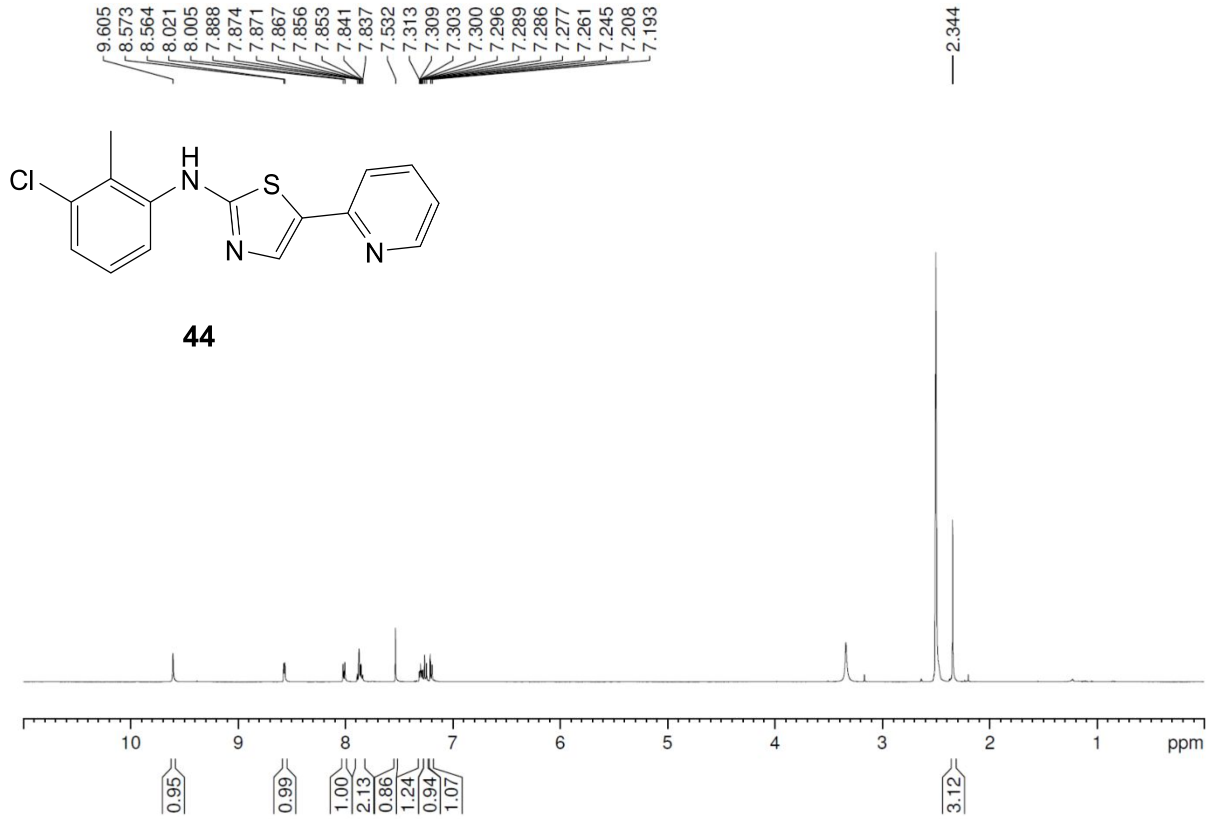

Spectrum 109: ^{1}H-NMR spectrum of aminothiazole **44** in $(CD_3)_2OS$.

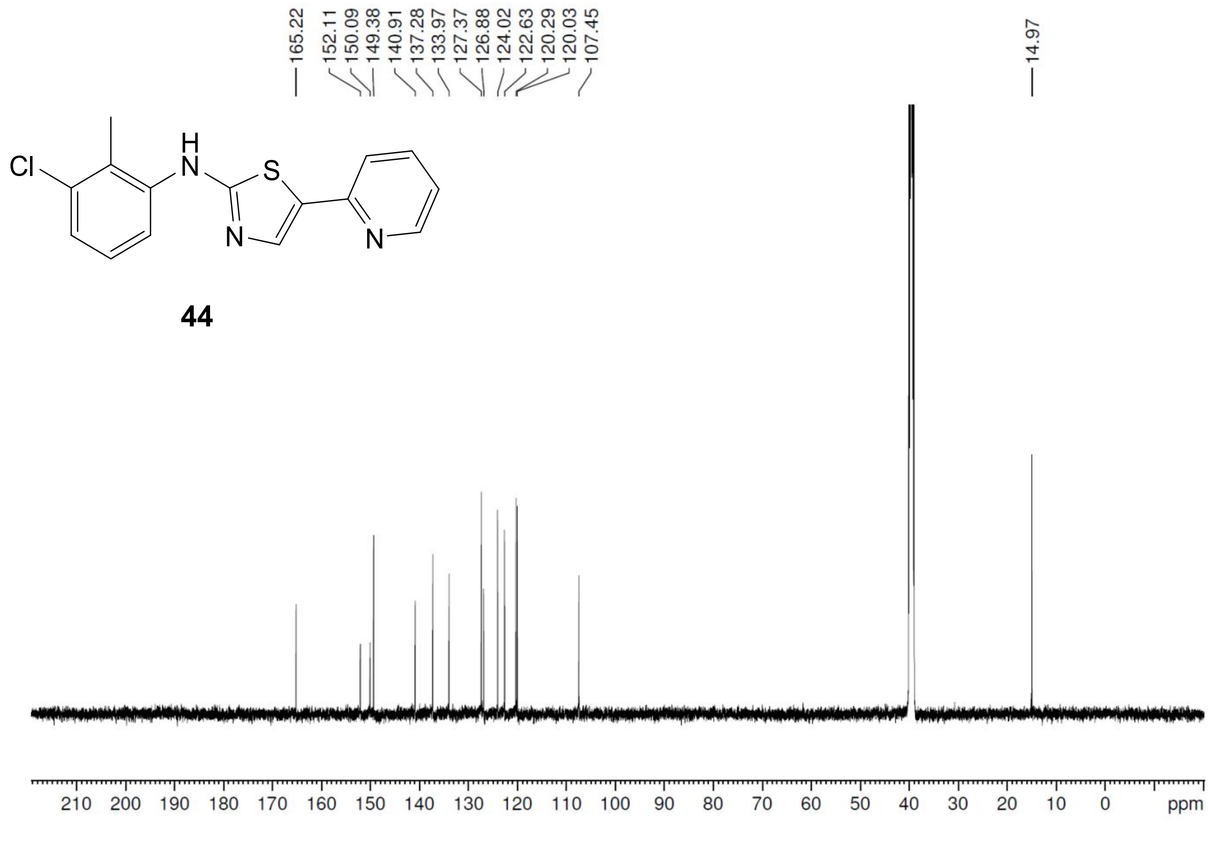

Spectrum 110: ^{13}C-NMR spectrum of aminothiazole **44** in $(CD_3)_2OS$.

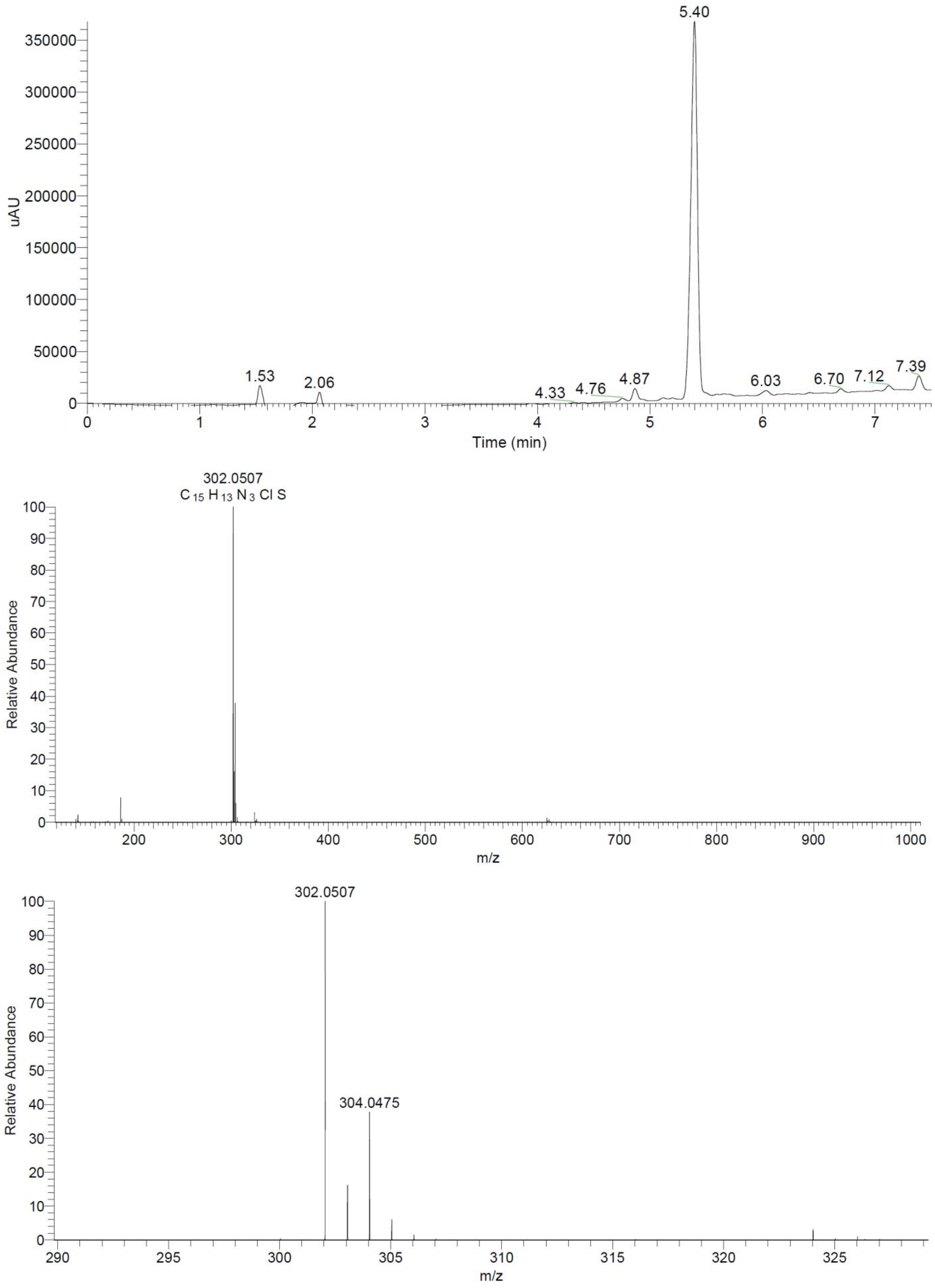

Spectrum 111: UV-trace and mass of main peak of aminothiazole **44**. Purity determined by peak area 97%.

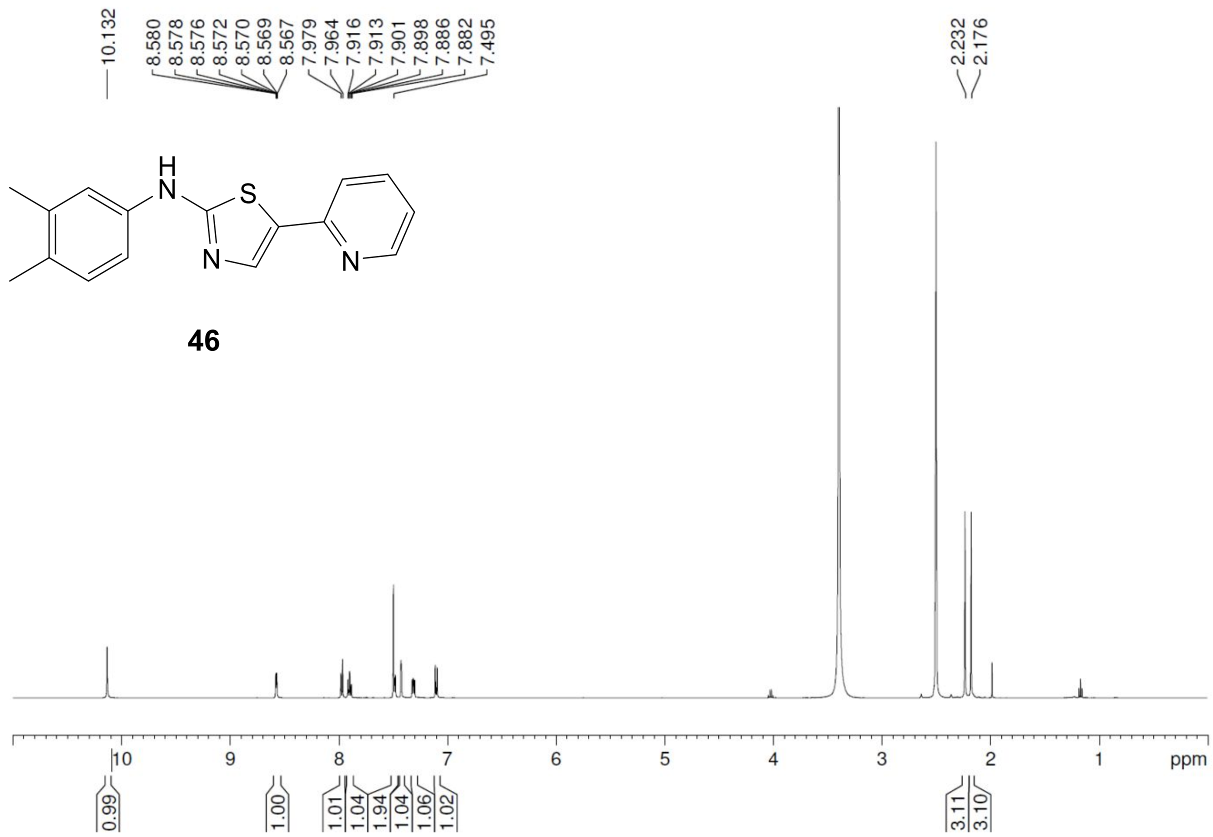

Spectrum 112: ^{1}H-NMR spectrum of aminothiazole **46** in $(CD_3)_2OS$.

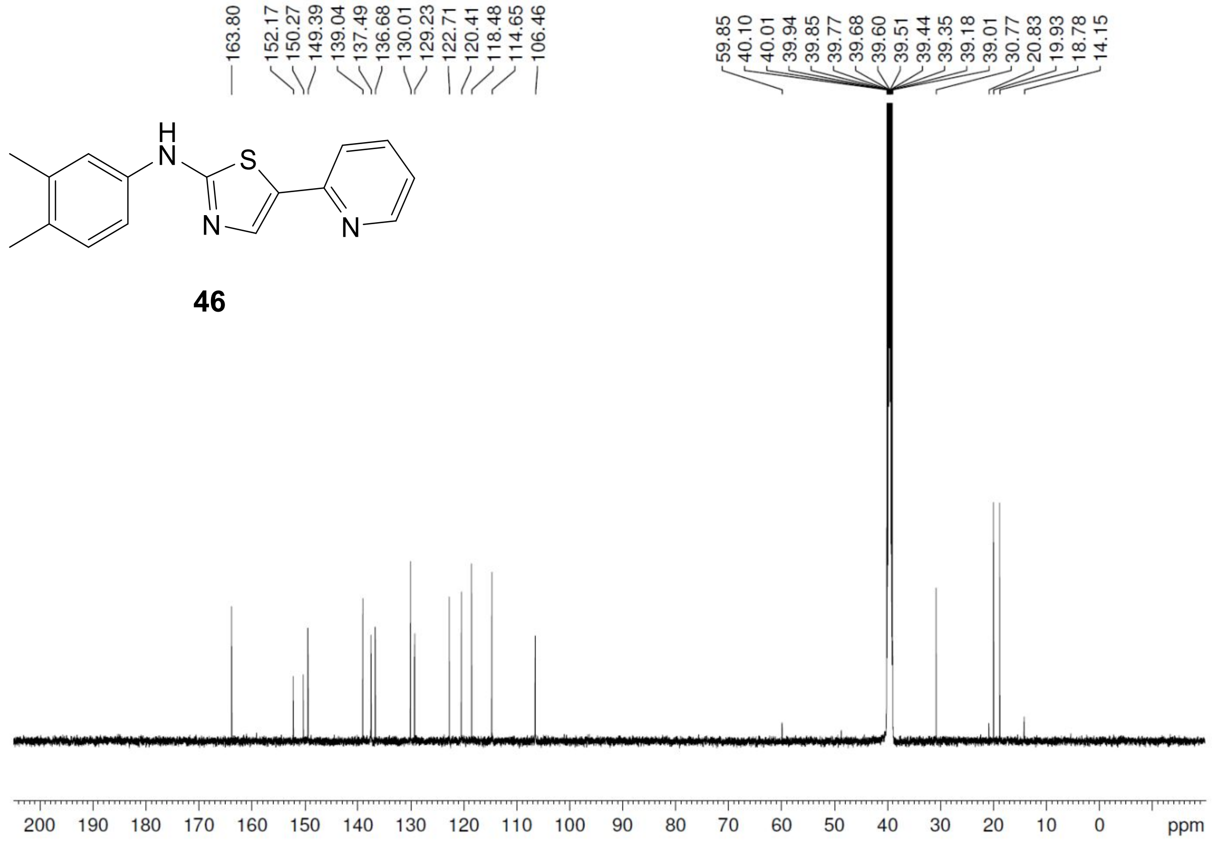

Spectrum 113: ^{13}C-NMR spectrum of aminothiazole **46** in $(CD_3)_2OS$.

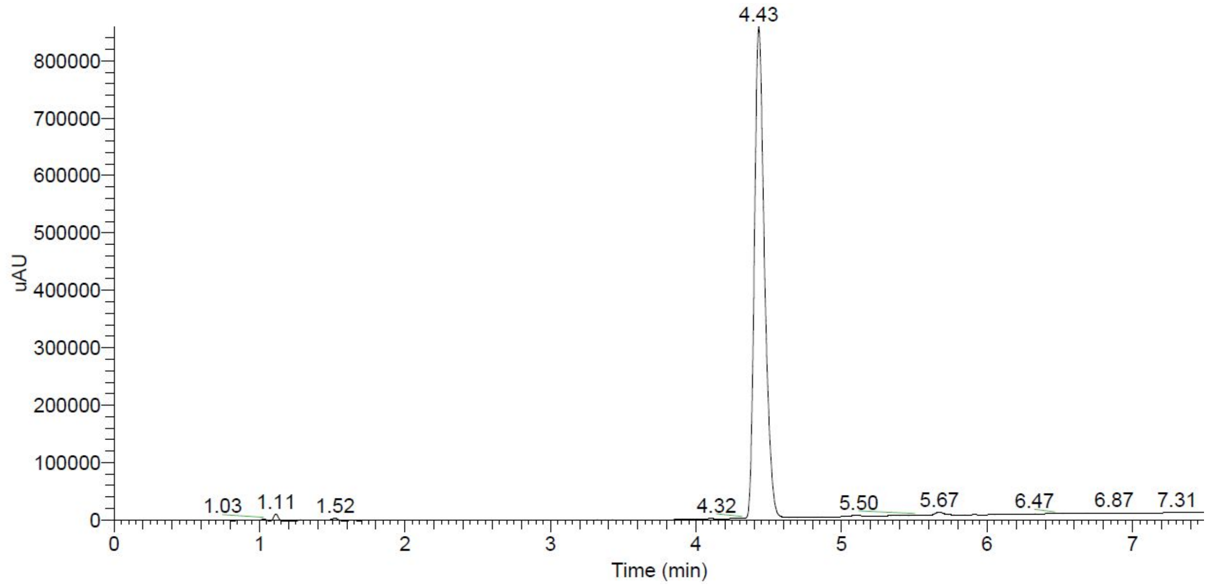

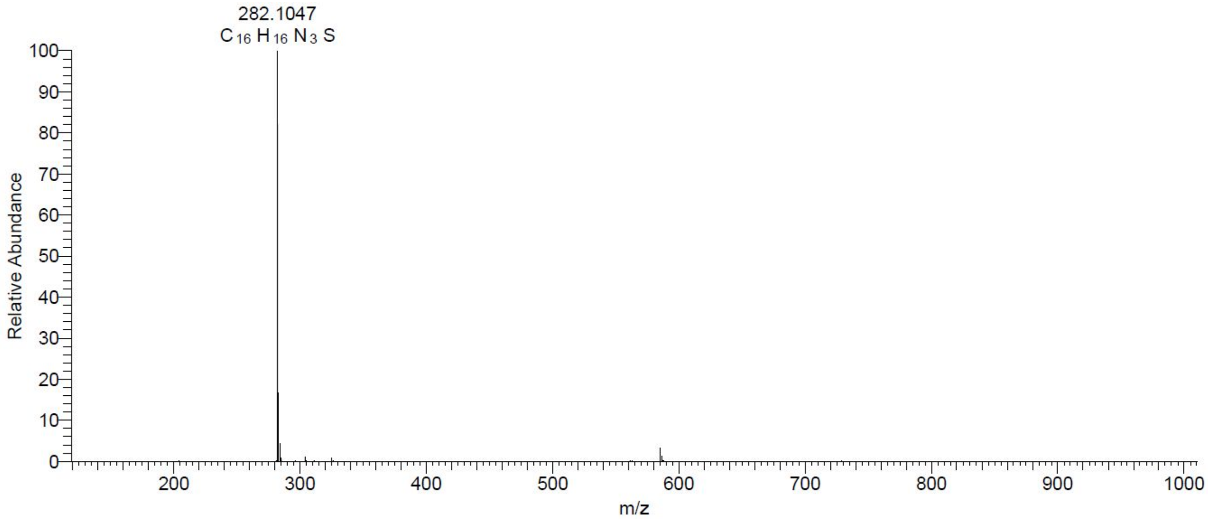

Spectrum 114: UV-trace and mass of main peak of aminothiazole **46**. Purity determined by peak area 99%.
